# Efficient synthesis of primary and secondary amides via reacting esters with alkali metal amidoboranes

**DOI:** 10.1038/s41467-021-25836-5

**Published:** 2021-10-13

**Authors:** Yu Guo, Ruo-Ya Wang, Jia-Xin Kang, Yan-Na Ma, Cong-Qiao Xu, Jun Li, Xuenian Chen

**Affiliations:** 1 Henan Key Laboratory of Boron Chemistry and Advanced Energy Materials, School of Chemistry and Chemical Engineering, Henan Normal University, Xinxiang, 453007 China; 2 Department of Chemistry, Southern University of Science and Technology, Shenzhen, 518055 China; 3 Green Catalysis Center and College of Chemistry, Zhengzhou University, Zhengzhou, 450001 China; 4 Department of Chemistry and Key Laboratory of Organic Optoelectronics & Molecular Engineering of Ministry of Education, Tsinghua University, Beijing, 100084 China

**Keywords:** Reaction mechanisms, Synthetic chemistry methodology

## Abstract

Amides are one of the most important organic compounds that are widely applied in medicine, biochemistry, and materials science. To find an efficient synthetic method of amides is a challenge for organic chemistry. We report here a facile synthesis method of primary and secondary amides through a direct amidation of esters with sodium amidoboranes (NaNHRBH_3_, R = H, Me), at room temperature without using catalysts and other reagents. This process is rapid and chemoselective, and features quantitative conversion and wide applicability for esters tolerating different functional groups. The experimental and theoretical studies reveal a reaction mechanism with nucleophilic addition followed by a swift proton transfer-induced elimination reaction.

## Abstract (web summary)

Amides are important organic compounds that are widely applied in medicine, biochemistry, and materials science. Here, the authors report a method for synthesis of primary and secondary amides through a direct amidation of esters with sodium amidoboranes (NaNHRBH_3_, R = H, Me), at room temperature without the use of catalysts.

## Introduction

**Fig. 1:**
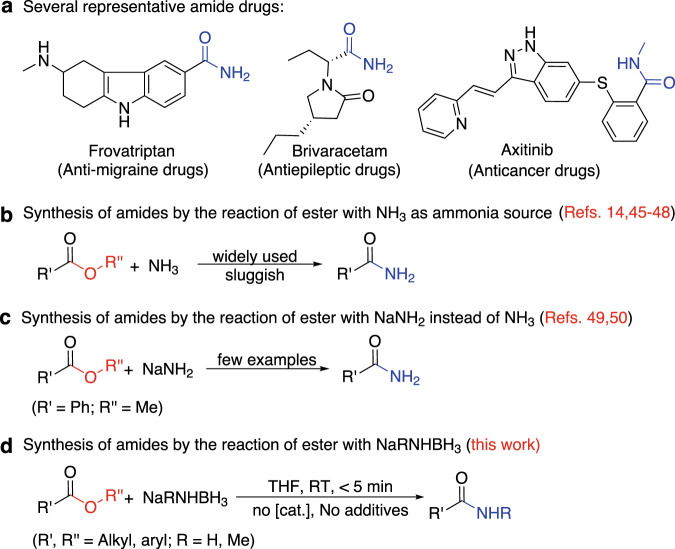
Various synthetic methods for amide bonds. **a** Several representative amide drugs. **b** Synthesis of amides by the reaction of ester with NH_3_ as ammonia source. **c** Synthesis of amides by the reaction of ester with NaNH_2_ instead of NH_3_. **d** Synthesis of amides by the reaction of ester with NaRNHBH_3_ (R = H, Me).

The formation of the amide bonds (–C(O)–NR_2_) is one of the most important organic reactions^1^ as the amide bond is a typically fundamental chemical bond^2^ that widely occurs in natural and industrial products, such as polypeptide protein, pesticide, polymer materials, and medicine^3–5^ (Fig. 1a). The most common synthetic methods currently used for the amide bonds rely almost solely on variants of the dehydration of carboxylic acid and amine, either with a catalyst^6,7^ or through the prior conversion of carboxylic acid into an activated carboxylate^8–10^ due to the requirement of harsh reaction conditions and long reaction times for the direct amidation of carboxylic acid^11–18^. Therefore, the direct amidation of esters with amines, without the conversion into carboxylic acids, is a highly desirable approach for the formation of amides^1^ as esters are common synthetic intermediates of the target compounds.

In addition to these conventional methods, alternative approaches to amide synthesis have been developed^19,20^, including different types of C-N connection manners: Staudinger ligation^21–23^, native chemical ligation^24^, chemoselective amide ligations^25^, nitroalkane-amine coupling^26^, alkyne-azide coupling^27,28^, and directly oxidative amidation with different starting materials such as alcohols^29,30^, aldehydes and ketones^31,32^ or alkynes^33^, and nitriles^34,35^ as well as ester with Mg_3_N_2_^36^. Although these contemporary methods for the formation of amide are successful and have been widely applied in amide synthesis from different types of substrates (For comparison, the main synthetic methods of amides in literature are summarized in Supplementary Table 3), the more efficient synthetic methods of amides are still considered as a challenge for organic chemistry and pharmaceutical industry^37–39^. Therefore, innovative methods for synthesis of amide functionality through mechanistically understandable approaches are needed^40,41^.

In retrospect to the direct amidation reaction, the reaction of esters with ammonia was investigated as an ammonolysis (solvolytic reaction) in 1930s^14,42–44^. Then this method was continually improved and widely used in synthetic chemistry^45^ and pharmaceutical synthesis^46,47^ (Fig. 1b) even though it is generally sluggish^48^. It is found that NaNH_2_ can be used instead of NH_3_ in some reactions to directly react with esters in liquid ammonia (Fig. 1c)^49,50^, and also the combination of NaNH_2_ as the nitrogen nucleophile and AlMe_3_ as the Lewis acid^15^ can convert esters to amides under microwave irradiation and high temperature^51^. We have recently explored the synthesis and reactivity of amine boranes (R_3_NBH_3_) and a concept of the nucleophilicity of the B-H and B-B bonding pair electrons has been proposed^42–54^. Based on this concept, we have elucidated the formation mechanisms of small borane complexes which have puzzled the community for many years^55–57^. In the light of these findings, we examined the reactions of sodium amidoborane (NaNH_2_BH_3_, NaAB) with esters with anticipation of the hydroboration of the ester substrates, similar to the literature work^58–61^, but an unconventional reaction has been observed.

In this work, we report the result that NaAB directly reacts with esters to quantitatively form the corresponding primary amides within 5 min at room temperature, without any catalyst and reagents. In addition, NaMeNHBH_3_ (NaMeAB) can also be used to synthesize *N*-Me secondary amides under the same conditions (Fig. 1d).

## Results and discussion

### Synthesis of primary amides and *N*-Me secondary amides

In our synthetic method, NaAB is soluble and stable in organic solvents^62^. Its reaction with methyl benzoate is selected as a model reaction to examine the reaction conditions and characterize the products. The reactions were carried out at room temperature and monitored by TLC or ^1^H NMR spectroscopy. The results indicate that the methyl benzoate can be consumed within 5 min when the reactant ratio of NaAB to methyl benzoate is over 2:1 (Supplementary Fig. 1). Thus, the 2.4:1 ratio was set up in all reactions to keep NaAB slightly excess. The reactions were also examined in different solvents (Table 1), which reveal that the solvents affected the reaction rate but not the isolated yield (entries 1–4), probably due to the solubility of NaAB therein (entries 3 and 4). We further scrutinized reactions with different alkyl (R”) benzoates (entries 5-9) and found that the yield did not appear to change significantly with increasing the steric hindrance of alkyl groups (entries 1 and 5-9). Consequently, a general procedure can be described as the reaction of an ester with NaAB under the optimized conditions that can be completed in less than 5 min with the ester being completely consumed (Table 1).

**Table 1:** Optimization of reaction conditions between NaAB and esters.


Entry	R”	Solvent	Time	Yield^a^
1	CH_3_	THF	5 min	91%
2	CH_3_	CH_3_CN	5 min	91%
3	CH_3_	Toluene	10 h	91%
4	CH_3_	CH_2_Cl_2_	10 h	90%
5	CH_2_CH_3_	THF	5 min	83%
6	CH_2_CH_2_CH_3_	THF	5 min	83%
7	*i*Pr	THF	5 min	91%
8	*t*Bu	THF	5 min	88%
9	Ph	THF	5 min	80%

All reactions were performed in 5 mL solvent with 0.5 mmol ester under N_2_ atmosphere and then hydrolysis.

^a^isolated yield.

**Fig. 2:**
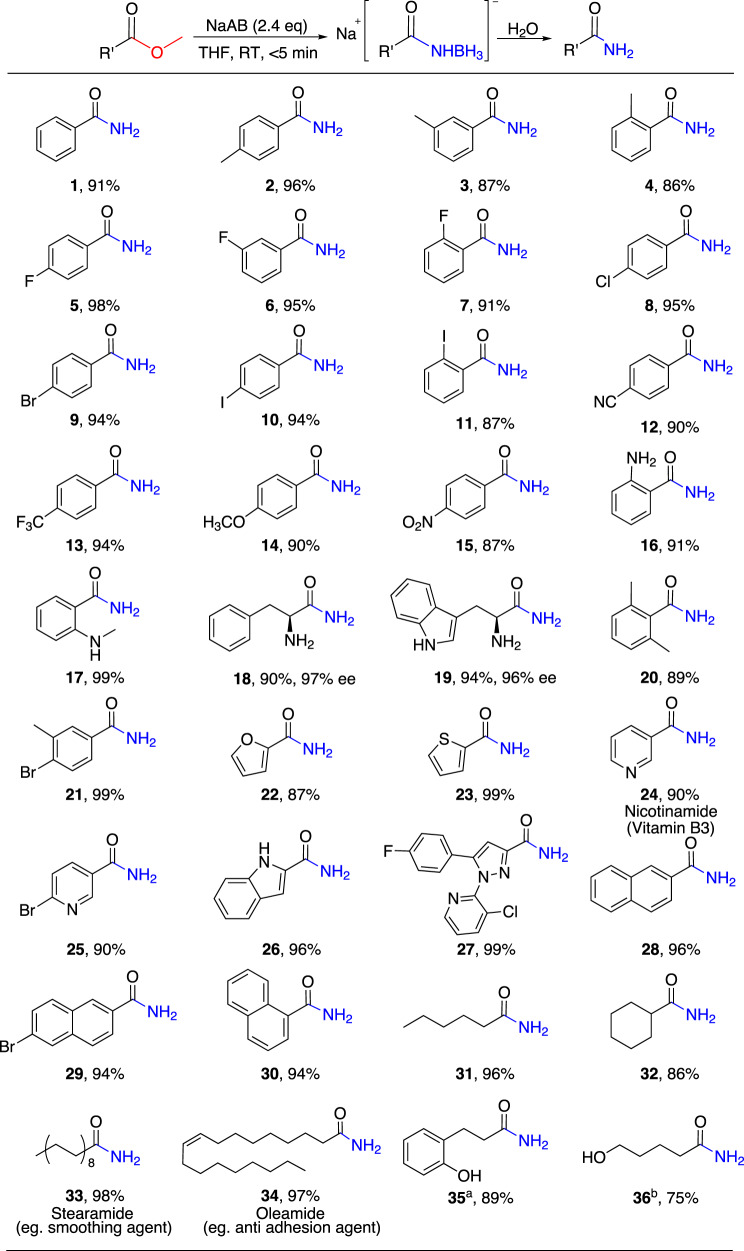
Reactions of NaAB with various esters in THF. Reaction conditions: ester (0.5 mmol), NaAB (2.4 equiv), THF (5 mL), room temperature for 5 min and then hydrolysis. ^a, b^The substrate esters are dihydrocoumarin and valerolactone, respectively.

With the optimized reaction conditions in hand, the scope of this approach has been explored (Fig. 2). Various substrate esters to form the corresponding primary amides (**1–36**) are studied. Substrate by screening shows that the isolated yield of benzamide (**1**) is 91%. The *p*-, *m*-, and *o*-methyl-substituted methyl benzoate participate in the reactions to produce the corresponding primary amides (**2**–**4**), and a good yield can be obtained, of which the yield of **2** is 96%. The substrates with electron-withdrawing groups F substituted at different positions (*p*-, *m*-, *o*-substituted) on the benzene ring provide corresponding amides (**5**-**7**) without affecting the yield of amide products. We have also investigated different methyl benzoate derivatives and found that substituents with different electronic properties on the aromatic ring have little effect on the yield of amides (**8**–**19**). Among them, the chiral compounds L-phenylalanine methyl ester and L-tryptophan methyl ester are derivatives of L-phenylalanine and L-tryptophan, respectively. The corresponding amides (**18, 19**) can be obtained with isolated yields of 90% and 94% by the reaction, and the racemization is not observed. The yields of 2,6-dimethyl-benzamide (**20**) and 4-bromo-3-methylbenzamide (**21**) are 89% and 99%, respectively, when there are two substituted methyl benzoate derivatives on the benzene ring. Moreover, different functional groups are tolerated under mild reaction conditions.

Next, we have tested the scope of the transformation by heterocycle, naphthyl, and aliphatic substrates, which behave identically to the corresponding amide derivatives (**22**–**34**). When different kinds of heterocyclic substrate esters participate in the reaction, the yield of corresponding amides (**22**–**27**) has been maintained. Furthermore, the yields of 1-naphthalene, 2-naphthalene, and 6-bromo-2-naphthamide remain unchanged (**28**–**30**). For the aliphatic amides, the long and short-chain or cycle substrate esters do not affect the yield of the corresponding amide (**31**–**34**). As expected, the product of the reaction of NaAB with a lactone is a hydroxyl substituted amide. For example, NaAB can react with dihydrocoumarin and valerolactone to produce 2-hydroxybenzenepropanamide (**35**), 5-hydroxypentanamide (**36**), respectively, with high yield. It is worth noting that the conversion rate of the ester is 100% based on the NMR (Supplementary Fig. 1) results. Therefore, the synthesis of primary amides from esters is universal through this metal amidoborane aided reaction, which can be applied to different types of aromatic and aliphatic esters to obtain high yield amides.

Especially noteworthy is the fact that this method can be applied to the synthesis of amide-bearing drug and material molecules (**24**, **33**, and **34** in Fig. 2). Vitamin B3 (Nicotinamide), a water-soluble vitamin, is synthesized in 90% yield (**24**). Stearamide (**33**) and oleamide (**34**), which are commonly used as smoothing anti-adhesion and waterproof agents and emulsifiers, can be obtained in 98% and 97% yield, respectively.

Considering that the amidoborane anion is an active reagent, especially as a reducing reagent^59–62^, the selectivity of the amidation of NaAB is investigated in the various combination of functional groups (Table 2). When a carbon-carbon double bond and an ester group coexist (entries 1-3), NaAB only reacts to the ester group but not reduces the double bond to form cinnamamide (**37–39**). As evidence of that, the yield of amide (**38**) from the reaction of methyl 3-phenylacrylate with NaAB is as high as 96%. When NaAB reacts with methyl 4-hydroxyl-benzoate (entry 4), the corresponding amide (**40**) is not formed. This is because NaAB abstracts the hydrogen from the OH group to form NH_3_BH_3_ (AB) and Na^+^(O-C_6_H_5_C(O)OCH_3_)^−^^62^. The latter precipitates from the reaction mixture and no longer reacts with NaAB (Supplementary Figs. 7-8). Hydrolysis of the formed sodium salt, methyl 4-hydroxyl-benzoate is re-formed. In addition, when the aldehyde group and the ester group coexist (entries 5 and 6), the aldehyde group can be selectively reduced by controlling the amount of NaAB. When the reaction ratios of NaAB and methyl 4-formylbenzoate are 1:1 and 3.4:1, the reaction products are methyl 4-(hydroxymethyl)benzoate (**41**) and 4-(hydroxymethyl)benzamide (**42**), respectively.

**Table 2:** The reactions of NaAB and esters with other functional groups.

Reaction conditions:entries 1–6, substrate ester (0.5 mmol), THF (5 mL), room temperature.

^a^Products after hydrolysis.

^b^isolated yield.

^c^The final product of this reaction is the product of Na^+^(O–C_6_H_5_C(O)OCH_3_)^−^ hydrolysis.

**Fig. 3:**
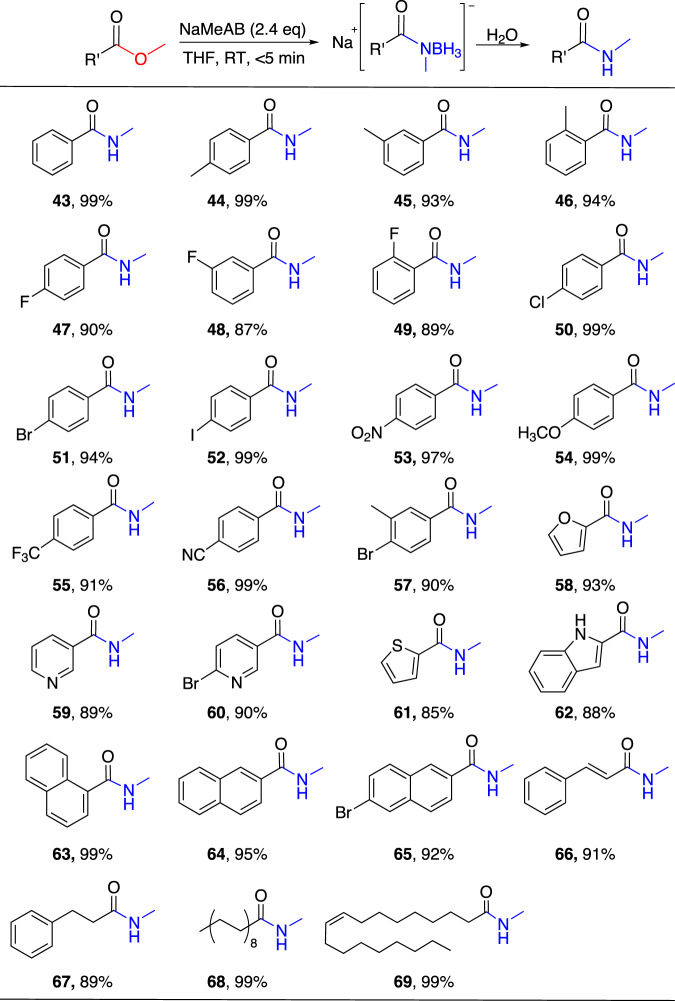
Reactions of NaMeAB with various esters in THF. Reaction conditions: ester (0.5 mmol), NaMeAB (2.4 equiv), THF (5 mL), room temperature for 5 min and then hydrolysis.

In order to explore the formation of secondary amides through this approach, we adopt NaMeAB, where a hydrogen on N in NaAB is replaced by a methyl group, to react with esters (Fig. 3). Based on the optimal reaction conditions of NaAB reacting with esters, we accordingly select the reaction conditions, as shown in Supplementary Table 2. The reaction of 2.4 equivalent of NaMeAB with methyl benzoate can be completed within 5 min in THF solvent at room temperature, and the yield of *N*-Me-benzamide (**43**) is 99%. On the benzene ring of methyl benzoate derivatives, the *o*-, *m*-, *p*-monosubstituted groups do not affect the yield of secondary amides (**44**–**56**), whether they are electron-withdrawing or electron-donating groups. Besides, the yield of 4-bromo-3-methylbenzamide (**57**) is 90%, which indicate that the substrate with two substituents on the benzene ring do not affect the yield of amide. More importantly, heterocyclic, naphthalene, and aliphatic substrate esters are also found to be suitable for reaction by NaMeAB to form corresponding secondary amides (**58**–**69**).

### Mechanism of the reactions

However, the reaction of NaMe_2_NBH_3_ with esters produced alcohols, as had been reported in 1984^58^. This reaction is completely different from the reactions of NaAB or NaMeAB with ester. Therefore, it is imperative to study the mechanism of the direct amidation reaction of ester and NaRNHBH_3_ to efficiently synthesize primary and secondary amides. In this regard, we set up the reaction of methyl benzoate with NaAB in THF-d_8_ at room temperature, and the NMR spectra of the reaction mixture are recorded in situ after 5 min (Supplementary Figs. 9–11). In the ^11^B NMR spectrum (Supplementary Fig. 9a), the quartet signal of NaAB starting material disappears, indicating that the reaction is completed. Two new quartet signals appear, assigned to the B atom of the formed NH_3_BH_3_ and Na[PhC(O)NHBH_3_], respectively, and both quartet signals display as a singlet at the corresponding chemical shift in ^11^B{^1^H} spectrum (Supplementary Fig. 9b). The ^1^H and ^1^H{^11^B} spectra (Supplementary Figs. 10-11) confirm the formation of NH_3_BH_3_ and Na[PhC(O)NHBH_3_]. The Na[PhC(O)NHBH_3_] intermediate is isolated and characterized by NMR and MS (Supplementary Figs. 2-6), which converts into the corresponding amides after hydrolysis. A singlet signal appears at δ 3.29 ppm is assigned to the H atom of the CH_3_ group in NaOCH_3_ (Supplementary Fig. 11c). Based on the characteristic signals and their related integral value in the ^1^H and ^11^B NMR spectra, the rapidly formed NH_3_BH_3_, NaOCH_3_, and Na[PhC(O)NHBH_3_] are with a 1:1:1 ratio (Supplementary Fig. 1).

In the process, a minimal of by-products are detected in the NMR spectra (Supplementary Fig. 9), which has been characterized as the [BH_3_NH_2_BH_2_NH_2_BH_3_]^–^ anion, forming from the reaction of the formed NH_3_BH_3_ with excess NaAB^63^. The observation of the minimal amount of these impurities is not unreasonable that is why 2.4 equivalent of NaAB is needed in the reaction despite two equivalents of NaAB is required as the stoichiometric value.

**Fig. 4:**
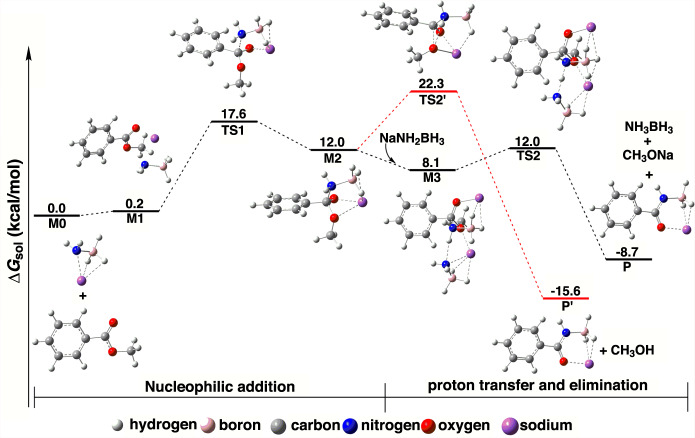
The reaction mechanism of methyl benzoate with NaAB with computed Gibbs free energy profile. White, H; orange, B; grey, C; blue, N; red, O; purple, Na.

**Fig. 5:**
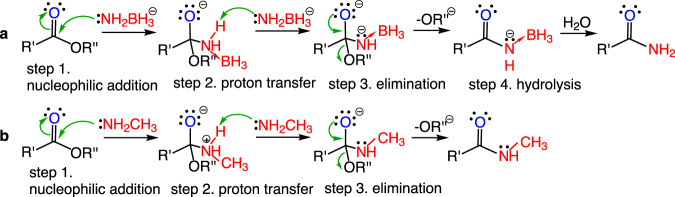
The proposed mechanism of the formation of amides by the reaction of esters with NaAB (a) and with amines (b). The both reaction including the nucleophilic addition, proton transfer, and elimination in which the molar ratio of NaAB or amine to ester (R’, R” =alkyl, aryl) is 2:1, however, the reaction with NaAB is fast while the reaction with amine is sluggish.

To provide insights into the reaction mechanism, quantum chemical studies using density functional theory (DFT) are carried out. From the experimental and theoretical data, the mechanism of the reaction is proposed as shown in Fig. 4 and Fig. 5. Firstly, as a nucleophile, lone pair electrons on the N atom of the [NH_2_BH_3_]^−^ anion in NaAB attack the C atom in the carbonyl group of methyl benzoate through TS1 (17.4 kcal/mol) to form the M2 intermediate. Then, the second [NH_2_BH_3_]^−^ anion easily abstracts the proton bonded to the N atom in M2 through TS2 (3.9 kcal/mol), leading to the formation of [PhC(O)NHBH_3_]^‒^, NH_3_BH_3_, and CH_3_O^‒^, which hydrolyze to form the final amide product and methanol. The first step is the rate determining step and the M2 intermediate has not been observed in the process as a discrete intermediate, probably because it rapidly proceeds to form [PhC(O)NHBH_3_]^‒^ with a barrier of 3.9 kcal/mol at TS2. The alternative pathway is to use the CH_3_O^‒^ group to abstract the proton bonded to the N atom in M2 through TS2’ (10.3 kcal/mol, the red line in Fig. 4) to form the CH_3_OH and [PhC(O)NHBH_3_]^‒^. But this pathway is less likely given the higher energy barrier and the fact that the reaction through TS2 can accomplish instantaneously. The influence of THF solvent on the reaction mechanism is also investigated. As shown in Supplementary Table 1, we find that the structure with three THF binding to NaAB is the most favorable, which is described as 3THF·NaAB and explored in the energy profile in Supplementary Fig. 18c. It reveals that compared with the SMD model, utilization of explicit solvation has a minor effect on the reaction mechanism.

### Features of the reactions

It is noteworthy that this synthetic method of primary and secondary amides through the reaction of esters and NaRNHBH_3_ (R = H, Me) can be carried out at room temperature without using any catalyst and reagents. Particularly, the reactions are completed within 5 min with virtually quantitative yield. Such a fast, high-yield, and chemoselective synthesis of amides is promissing. The theoretical results indicate that there are two elementary reactions in the whole procedure. The first one is a nucleophilic addition reaction, where the reactants usually cannot completely convert into the four-coordinated intermediate (M2) as an equilibrium reaction. However, the second elementary reaction involves a proton transfer-induced elimination of MOR” with a tiny energy barrier (3.9 kcal/mol). The irreversible elimination reaction in the second step instantaneously reduces the concentration of M2, thus facilitating conversion of all the reactants into the product. Therefore, this reaction can occur at a fast rate with a high yield from thermodynamic and kinetic consideration.

While the mechanism of this reaction bears similarity to that of the typical ammonolysis of esters, as shown in Fig. 5, the nucleophilicity of the [NH_2_BH_3_]^−^ anion is much stronger than that of the neutral amine molecules in ammonilysis. Although the amidoborane anion ([NH_2_BH_3_]^−^) and methylamine (NH_2_CH_3_) are isoelectonic, the [NH_2_BH_3_]^−^ anion is a much stronger proton abstractor than amines so that it rapidly abstracts proton to form NH_3_BH_3_. Thus, both the strong nucleophilicity and basicity of the [NH_2_BH_3_]^−^ anion give rise to this fast reaction with high yield. In addition, the alkali metal cation plays an important role in this reaction through activating the carbonyl group^64^. Therefore, the direct amidation of esters with alkali metal amidoboranes is an efficient way of preparing amides despite that the typical ammonolysis of esters is generally sluggish.

Furthermore, these alkali metal amidoborane aided reactions is quite different from the reactions of ester with NH_3_, NH_2_CH_3_ or NaNH_2_, as previously reported^14,45–50^. We have further investigated the properties of NH_3_, [NH_2_]^−^, and [NH_2_BH_3_]^−^ as a nucleophile because they all have lone pair electrons. The DFT results reveal that the nucleophilicity of [NH_2_BH_3_]^−^ is stronger than that of NH_3_ but weaker than that of [NH_2_]^−^ on the basis of the calculated average local ionization energy (ALIE) (Supplementary Fig. 17). Indeed, the calculated energy barriers of the nucleophilic addition are consistent with their nucleophilicity. While the reaction with NH_3_, having the highest barrier of 53.4 kcal/mol (Supplementary Fig. 18a), cannot take place at room temperature without catalyst^7^. The reaction with [NH_2_]^−^ (with a barrier of 3.1 kcal/mol, Supplementary Fig. 18b) should occur easily in the gas phase (in practice, it can only be conducted in harsh conditions in organic solvent^24^ because of the low solubility of solid NaNH_2_). Therefore, in comparison with NaNH_2_, the better solubility of NaAB in organic solvents, due to dative-bonding interaction between [NH_2_]^−^ and the BH_3_ group, plays a crucial role in these reactions. This result is consistent with the fact that esters reacting with NaAB in polar solvents (e.g., THF and CH_3_CN) can be finished within 5 min, but will take ~10 h in weak-polar solvents (e.g., toluene, and CH_2_Cl_2_, Table 1).

Another feature of these reactions is that the electronic and steric effects are not sensitive (Table 1), even though these factors often significantly influence the yield and rate of the organic reactions. This scenario is probably related to the strong nucleophilicity and basicity of the [NH_2_BH_3_]^−^ anion that strongly pulls the equilibrium shifting toward the product. In addition, the advantage of this method is that the products can be purified without using column chromatography and the amine borane main by-product could be obtained at a high yield. See supplementary materials for detailed information.

Based on these insights, we have further examined the reactions of methyl benzoate with LiNH_2_BH_3_, NaNH_2_BH_3_, and KNH_2_BH_3_ (2.4 equiv) in THF at room temperature. While using NaNH_2_BH_3_ and KNH_2_BH_3_, methyl benzoate is completely converted into benzamide in 5 min, only 64% of the ester is converted into benzamide and the rest is converted to benzyl alcohol (Supplementary Fig. 19) when using LiNH_2_BH_3_. This difference likely arises from the partially covalent nature of the Li–N bond in LiNH_2_BH_3_ structure^65^, where the tight ion-pair structure affects the nucleophilic ability of lone pair electrons on the N atom.

We have developed here an efficient synthetic method for the primary and secondary amides tolerating different functional groups through the reaction of esters and metal amidobroane at room temperature. A reaction mechanism is found to involve a nucleophilic addition and a proton transfer-induced elimination reaction. The work-up process of this synthetic method is simple, green, and efficient. This method provides a general strategy in developing chemoselective synthetic approaches of amides.

## Methods

### Computational Details

The quantum chemical studies were carried out using density functional theory (DFT). All DFT calculations were performed with Gaussian−16 package^66^ using M06 functional^67^ and 6–311 + +G** basis set^68^. The geometries of all the species were optimized. Harmonic vibrational frequencies were computed to confirm that all local minima have no imaginary frequency while all transition states (TSs) have one imaginary frequency. Besides, the connection of the TS to its corresponding reactant and product was verified via the intrinsic reaction coordinate (IRC) calculations. Single point energy calculations based on geometries obtained with the 6–311 + +G** basis set were carried out using the aug-cc-pVTZ basis sets^68^. Gibbs’s free energies were calculated by considering the aero-point energy and thermal corrections at 298.15 K and 1 atm pressure. Solvent (tetrahydrofuran) effects were taken into account via the SMD model by Truhlar and Cramer^69^. All the other computational details used the default settings.

### General method for the synthesis of primary or secondary amides

Under N_2_ atmosphere, NaRNHBH_3_ (R = H, Me) (1.20 mmol) was put into a 10 mL flask which was connected to a Schlenk line and then 5 mL of THF was added. Then 0.5 mmol of substrate ester was added. The reaction mixture was stirred at room temperature and monitored by ^1^H NMR spectroscopy or TLC. After 5 min, the substrate ester was consumed, and then the solvent was pumped out. Next, the mixed solvent of CH_2_Cl_2_ and *n*-hexane (volume ratio of 10:1) was added to extract RNH_2_BH_3_, while Na[R’CONRBH_3_] (R’ = alkyl, aryl; R = H, Me) and other boron-containing compounds are left in the residual. Then, add 15 mL water into the flask and stir for 15 min. Addition of ethyl acetate extract the product (7–8 times) and the organic phases are collected and dried. The amide products with high purity and yield were obtained.

## Supplementary information

Supplementary Information

## Data Availability

The data supporting the findings of this study are available within the paper and its supplementary information files.
