# Supplementary material for: Efficient synthesis of primary and secondary amides via reacting esters with alkali metal amidoboranes
Source: Nat Commun. 2021 Oct 13;12:5964. doi: 10.1038/s41467-021-25836-5 (PMC8514480; doi:10.1038/s41467-021-25836-5)
Supplement: Supplementary file 1 — Supplementary Information [file 41467_2021_25836_MOESM1_ESM.pdf]

Supplementary Information for

Efficient synthesis of primary and secondary amides via reacting esters with alkali metal amidoboranes

Yu Guo, Ruo-Ya Wang, Jia-Xin Kang, Yan-Na Ma, Cong-Qiao Xu, Jun Li, Xuenian Chen

Correspondence to: xnchen@htu.edu.cn, junli@tsinghua.edu.cn

Table of Contents

1. Supplementary Methods **S3**

2. Supplementary notes **S6**

Supplementary note 1 .... **S6**

Supplementary note 2 .... **S8**

Supplementary note 3 .... **S16**

3. Supplementary Figures **S26**

4. Supplementary References **S127**

1. Supplementary Methods

Materials: Organic solvents were purified by rigorous degassing with nitrogen before passing through a PureSolv solvent purification system. Unless otherwise stated, all esters are purchased from Macklin or Aladdin and used without further purification. $MRNHBH_3$ (M = Li, Na, K; R = H, Me) is made from MH and RNH_2BH_3 by the literature method[1-2].

General experimental details: ^1H, $^1H\{^{11}B\}$, ^{13}C, ^{19}F, ^{11}B and $^{11}B\{^1H\}$ NMR spectra were recorded on a Bruker AVANCE 600 MHz spectrometer. ^1H NMR spectra were internally referenced to the residual solvent signal (e.g. CD_3CN = 1.94 ppm, DMSO-d_6 = 2.50 ppm). ^{13}C NMR spectra were internally referenced to the residual solvent signal (e.g. CD_3CN = 118.26 ppm, DMSO-d_6 = 39.52 ppm). The ^{11}B NMR spectra were obtained at 193 MHz and externally referenced to $BF_3{\cdot}OEt_2$ in C_6D_6 (δ = 0.00 ppm). High-resolution mass spectra (HRMS) were obtained via an electrospray ionization (ESI) mode using a MicrOTOF mass spectrometer. The enantiomeric excess (ee) of the products were determined by high-performance liquid chromatography (HPLC) with a chiral stationary phase in comparison with the authentic racemate sample. The chiral stationary phases Chiralcel OD-XF133 used in this study were purchased from Daicel Chirsal Technologies. Optical rotations were reported as follows: [α] D T = (c: g/100 mL, in $CDCl_3$). X-ray diffraction (XRD) data were collected with a Rigaku D/max 2500 diffractometer using the Cu/Ka radiation (λ = 0.1542 nm, 40 kV, 100 mA).

All operations are carried out on the Schlenk line or in a glove box filled with high-purity nitrogen. Analytical thin layer chromatography (TLC) was conducted with silica gel plate. Visualization of developed plates was performed under UV light (254 nm) or iodine vapors. A general synthetic procedure as below (substrate esters take methyl benzoate as an example):

A 10 mL flask containing 0.0636g (1.20 mmol) NaAB was connected to a Schlenk line and then 5 mL of THF and 0.5 mmol of methyl benzoate were successively added. The reaction mixture was stirred at room temperature and monitored by ^1H NMR spectroscopy or TLC. The reaction is completed with the benzoate was consumed by 5 minutes. The main products after reaction $Na[PhC(O)NHBH_3]$, NH_3BH_3, and a small amount of $NaBH_3NH_2BH_2NH_2BH_3$ can be separated according to their different solubility. Firstly, THF was pumped out from the reaction mixture for the cyclically used to produce a solid. NH_3BH_3 was extracted from the "solid mixture" with a mix-solvent of 5 mL CH_2Cl_2 and 0.5 mL *n*-hexane at a volume ratio of 10:1 for cyclical utilization. Then, a mix-solvent of water and ethyl acetate at 1:1 ratio was added to extracted $NaBH_3NH_2BH_2NH_2BH_3$ (water phase) and benzamide (ethyl acetate phase after $Na[PhC(O)NHBH_3]$ hydrolysis). Collect the organic phase and spin dry to obtain pure benzamide. In the same treatment way, the secondary amides are obtained.

The solvent effect of THF on the reaction mechanism: To investigate the solvent effect of THF on the reaction mechanism, the maximum number of THF molecules binding to NaAB is studied. As shown in Supplementary Table 1, we have found that the structure with three THF binding to NaAB is the most favorable, which is described as 3THF·NaAB and explored for reaction mechanism. As the system is very huge by considering three THF molecules, the structures of M3 and TS2 were optimized using M06 functional and the smaller 6-31+G* basis sets. Then single point energy calculations were performed with aug-cc-pVTZ basis sets and used to generate the free energy profile (Supplementary Figure 18c), which is the same as all other calculations. Compared with the energy profile using SMD model to simulate the solvent effect,

the energy of TS2' has been increased by 10.1 kcal/mol, the first step barrier is reduced by 11.3 kcal/mol. So it's more reasonable to consider about solvent coordination.

Supplementary Table 1. Binding energy of nTHF (n = 1-5) on NaAB.

nTHF·NaAB	ΔG_{sol} (kcal/mol)
1THF·NaAB	-8.6
2THF·NaAB	-14.1
3THF·NaAB	-16.8
4THF·NaAB	-15.4
5THF·NaAB	-14.9

Optimization of reaction conditions: On the basis of optimizing the reaction conditions of NaAB and esters, we investigated the reactions of different benzoate esters with NaMeAB (Supplementary Table 2). The reaction showed that different substrate esters did not affect the reaction time and separation yield (entries 1-5).

Supplementary Table 2. Optimization of reaction conditions between NaMeAB and esters.

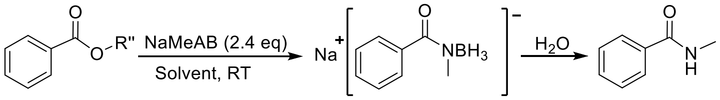

Entry	R''	Solvent	Time	Yield[a]
1	CH_3	THF	5 min	99%
2	CH_2CH_3	THF	5 min	96%
3	$CH_2CH_2CH_3$	THF	5 min	96%
4	*i*Pr	THF	5 min	92%
5	Ph	THF	5 min	99%

All reactions were performed in 5 mL solvent with 0.5 mmol ester under N_2 atmosphere and then hydrolysis. [a]isolated yield.

2. Supplementary notes

Supplementary note 1

Review of main synthetic methods of amides. The main synthetic methods of amides in the literature are summarized in Supplementary Table 3.

Supplementary Table 3. Synthetic methods of amide

Refs.	Reaction equations	Condition and yileds
3	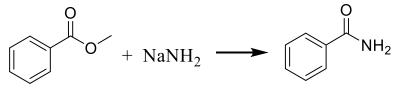	150 W, 150 °C, 200 psi, THF, 45 min, 80-89%
4	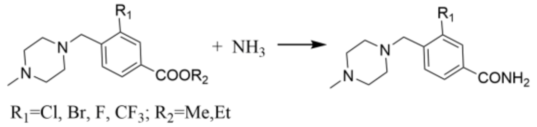	150 °C, 50 atm, 18 h, 80-87%
5-6	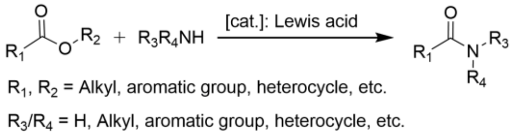	eg. a)Zr(Ot-Bu)$_4$:HOAt = 1:1, toluene, 60 or 100 °C, 1-48 h, 80-99%<br>b) La(OTf)$_3$, rt-70 °C, 1-48 h,79->99%
7-9	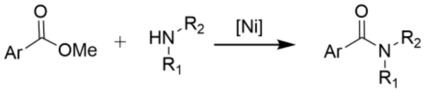	eg. a) Ni(cod)$_2$, Privileged NHC ligands, t-BuOK, toluene, 140 °C, 16 h, 22-90%<br>b) Ni(cod)$_2$, SIPr, or Al(O^{t}Bu$_3$)$_3$, toluene, 60 °C 15-89%<br>c) Ni(cod)$_2$, IPr, PhMe, 140 °C, 16 h, 38-94%
10-11	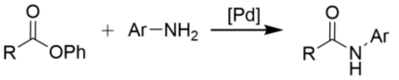	eg. a) Pd-PEPPSI, K_2CO_3, DME, 110 °C, 16 h, 50-97%<br>b) Pd(Ipr)(allyl)Cl, K_2CO_3, H_2O, PhMe, 110 °C,16 h, 55-97%
12	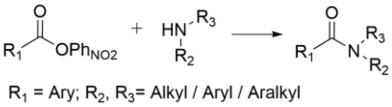	K_2CO_3, THF, 55 °C, 3 h, 32-93%
13	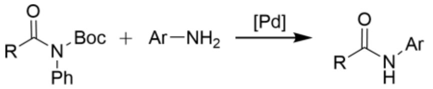	eg. Pd(iPr)(cinnamyl)Cl, K_2CO_3, DME, 110 °C,15 h, 74-98%
14-15	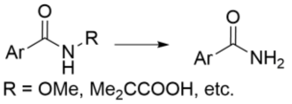	eg. a) R = OMe; S_8 (0.3 equiv.), DABCO (2 equiv.), DMSO, 80 °C, 70-94%<br>b) R = Me$_2$CCOOH; CuCl$_2$ (0.5 mmol), DMSO, 120 °C, 11 h, 84-96%
16-18	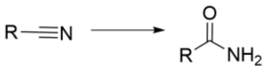	eg. a) KO^{t}Bu(3 equiv.), ^{t}BuOH, RT, 4-36 h, 55->99%<br>b) phosphate buffer pH 7.0 (3.75 mL), DMSO (1.25 mL), NHase (2.0 µL, PE), 17 h, 30 °C, Average conversion 99.9%<br>c) RhCl(PPh$_3$)$_3$ (1 mol%), MeCH=N-OH, toluene, 110 °C, 75-99%
19	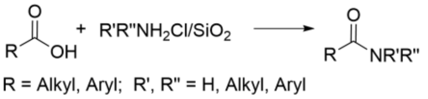	TsCl and TEA, solvent-free conditions, rt, rapid, 70-90%

20	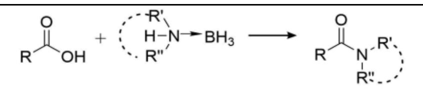 R = H, Alkyl, Ar; R' = H, Alkyl, Ar; R'' = H, Alkyl	xylenes, reflux, 12 h, 50-99%
21-22	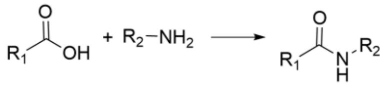	eg. a) $TiCl_4$ or $ZrCl_4$ (20 mol%), 500 mg MS (4A˚), THF/toluene, 100-120 ºC, 24 h, 42-99%<br>b) R_1 = alkyl, aryl; R_2 = -$CONH_2$; imidazole, 300 W, 90-360 sec, 47-88%
23-25	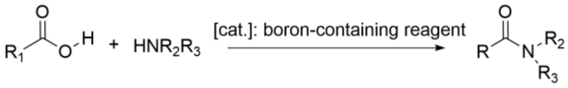 [cat.]:boric acid, boric anhydride, borane and other boron-containing reagents etc.	eg. a) A biphenylbased diboronic acid anhydride, toluene, 60 or 110 ºC， 2-24 h， 67->99%<br>b) Tetrakis(dimethylamido)diboron or tetrahydroxydiboron (1-2 mol%), toluene, azeotropic reflux in air, 7 h, 31 (or 8%)-98%<br>c) 2,4-$(CF_3)_2$-$C_6H_3B(OH)_2$ (10 mol%), CH_2Cl_2, 85-110 ºC, 12-36 h, 90-99%
26-27	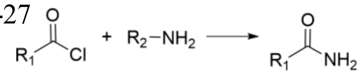 R_1 = Aryl. Hetero aryl, aliphatic; R_2 = Aryl. Hetero aryl, aliphatic, H,etc.	eg. a) R_2 ≠ H; 100-120 ºC, 5-6 h, 67-94%<br>b) R_1 = Ph, R_2 = H; poly(4-vinylpyridine) (6 equiv), PhCOCl, K_2CO_3, 50 ºC, 0.5 h, 95%
28	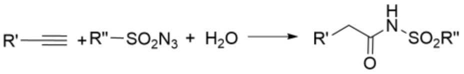	CuI (cat), Et_3N, $CHCl_3$, 25 ºC, 12 h, 74-97%
29	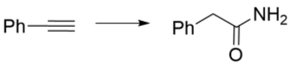	[Mn(2,6-Cl_2TPP)Cl], Oxone/NH_4HCO_3, CH_3CN/H_2O, rt, 1 h, 100% conv. 80% yield
30-31	RCH_2OH + R−NH_2 ⟶ RNHCOR<br>R = alkyl, aryl	eg. a) a ruthenium complex based on a dearomatized PNN-type ligand (PNN:2-(di-tertbutyllphosphinomethyl)-6-(diethylaminomethyl)pyridine) 0.1 mol%, toluene, reflux, 7-12 h, 58-99%<br>b) 5% Ru(COD)Cl_2, 5% D, 20% KOtBu, 5% $PCyp_3$·HBF_4, tolune, 110 ºC, 24 h, 21-100%
32-33	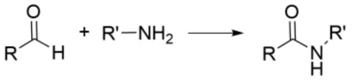	eg. a) CuI, $AgIO_3$, $CaCO_3$, T-HYDRO, MeCN, 40 ºC, 6 h, 39-91%<br>b) Oxone, $MgSO_4$, ball milling (30 Hz), rt, 90 min, 38-78%

Supplementary note 2

Characterization data of products:

Benzamide (1)[34]

^{1}H NMR (600 MHz, CD_3CN) δ 7.84 (d, *J* = 7.2 Hz, 2H), 7.54 (t, *J* = 7.4 Hz, 1H), 7.46 (t, *J* = 7.7 Hz, 2H), 6.86 (s, 1H), 6.26 (s, 1H); ^{13}C NMR (151 MHz, CD_3CN) δ 169.8, 134.9, 132.5, 129.3, 128.3.

4-methylbenzamide (2)[34]

^{1}H NMR (600 MHz, CD_3CN) δ 7.71 (d, *J* = 7.7 Hz, 2H), 7.27 (d, *J* = 7.5 Hz, 2H), 6.69 (s, 1H), 5.95 (s, 1H), 2.38 (s, 3H); ^{13}C NMR (151 MHz, CD_3CN) δ 169.5, 143.1, 132.1, 129.9, 128.4, 21.4.

3-methylbenzamide (3)[34]

^{1}H NMR (600 MHz, CD_3CN) δ 7.66 (s, 1H), 7.61 (d, *J* = 7.2 Hz, 1H), 7.38-7.30 (m, 2H), 6.79 (s, 1H), 6.12 (s, 1H), 2.38 (s, 3H); ^{13}C NMR (151 MHz, CD_3CN) δ 169.9, 139.2, 134.9, 133.1, 129.2, 128.9, 125.4, 21.3.

2-methylbenzamide (4)[34]

^{1}H NMR (600 MHz, CD_3CN) δ 7.40 (s, 1H), 7.33 (s, 1H), 7.24 (s, 1H), 7.22 (s, 1H), 6.45 (s, 1H), 6.12 (s, 1H), 2.41 (s, 3H); ^{13}C NMR (151 MHz, CD_3CN) δ 172.5, 137.1, 136.7, 131.7, 130.6,127.9, 126.4, 19.9.

4-fluorobenzamide (5)[34]

^{1}H NMR (600 MHz, CD_3CN) δ 7.91-7.81 (m, 2H), 7.18 (t, *J* = 8.7 Hz, 2H), 6.73 (s, 1H), 6.01 (s, 1H); ^{13}C NMR (151 MHz, CD_3CN) δ 168.5, 165.6 (d, *J* = 249.0 Hz), 131.3 (d, *J* = 2.9 Hz), 131.0 (d, *J* = 9.1 Hz), 116.1 (d, *J* = 22.0 Hz); ^{19}F NMR (565MHz, CD_3CN) δ -109.6.

3-fluorobenzamide (6)[34]

^{1}H NMR (600 MHz, CD_3CN) δ 7.64 (d, *J* = 7.6 Hz, 1H), 7.56 (d, *J* = 9.8 Hz, 1H), 7.48 (dd, *J* = 14.2, 7.0 Hz, 1H), 7.29 (t, *J* = 8.4 Hz, 1H), 6.81 (s, 1H), 6.17 (s, 1H); ^{13}C NMR (151 MHz, CD_3CN) δ 168.3, 163.5 (d, *J* = 244.5 Hz), 137.4 (d, *J* = 6.0 Hz), 131.4 (d, *J* = 9.1 Hz), 124.2 (d, *J* = 3.0 Hz), 119.2 (d, *J* = 22.6 Hz), 115.3 (d, *J* = 22.6 Hz); ^{19}F NMR (565MHz, CD_3CN) δ -114.3.

2-fluorobenzamide (7)[34]

^{1}H NMR (600 MHz, $CDCl_3$) δ 8.12 (td, *J* = 7.9, 1.3 Hz, 1H), 7.50 (td, *J* = 7.3, 1.5 Hz, 1H), 7.28 (d, *J* = 7.5 Hz, 1H), 7.14 (dd, *J* = 11.9, 8.4 Hz, 1H), 6.70 (s, 1H), 6.11 (s, 1H); ^{13}C NMR (151 MHz, $CDCl_3$) δ 165.0, 161.1 (d, *J* = 248.2 Hz), 134.1 (d, *J* = 9.1 Hz), 132.4 (d, *J* = 1.5 Hz), 125.0 (d, *J* = 3.0 Hz), 120.2 (d, *J* = 12.1 Hz), 116.2 (d, *J* = 24.2 Hz); ^{19}F NMR (565MHz, $CDCl_3$) δ -112.8.

4-chlorobenzamide (8)[35]

^{1}H NMR (600 MHz, DMSO-d_6) δ 8.03 (s, 1H), 7.88 (d, *J* = 8.5 Hz, 2H), 7.52 (d, *J* = 8.4 Hz, 2H), 7.45 (s, 1H); ^{13}C NMR (151 MHz, DMSO-d_6) δ 166.8, 136.1, 133.0, 129.4, 128.3.

4-bromobenzamide (9)[36]

^{1}H NMR (600 MHz, DMSO-d_6) δ 8.04 (s, 1H), 7.81 (d, *J* = 8.0 Hz, 2H), 7.66 (d, *J* = 7.9 Hz, 2H), 7.46 (s, 1H); ^{13}C NMR (151 MHz, DMSO-d_6) δ 167.2, 133.7, 131.5, 129.9, 125.3.

4-iodobenzamide (10)[34]
^{1}H NMR (600 MHz, DMSO-d_6) δ 8.02 (s, 1H), 7.83 (d, *J* = 8.2 Hz, 2H), 7.65 (d, *J* = 8.0 Hz, 2H), 7.43 (s, 1H); ^{13}C NMR (151 MHz, DMSO-d_6) δ 167.2, 137.1, 133.7, 129.5, 98.9.

2-iodobenzamide (11)[34]
^{1}H NMR (600 MHz, CD_3CN) δ 7.90 (d, *J* = 8.0 Hz, 1H), 7.46-7.36 (m, 2H), 7.15 (s, 1H), 6.46 (s, 1H), 6.14 (s, 1H); ^{13}C NMR (151 MHz, CD_3CN) δ 171.8, 143.3, 140.6, 131.8, 129.1, 128.8, 92.6.

4-cyanobenzamide (12)[34]
^{1}H NMR (600 MHz, DMSO-d_6) δ 8.21 (s, 1H), 8.01 (d, *J* = 8.3 Hz, 2H), 7.93 (d, *J* = 8.4 Hz, 2H), 7.67 (s, 1H); ^{13}C NMR (151 MHz, DMSO-d_6) δ 166.6, 138.2, 132.3, 128.2, 118.4, 113.6.

4-trifluoromethylbenzamide (13)[35]
^{1}H NMR (600 MHz, DMSO-d_6) δ 8.20 (s, 1H), 8.07 (d, *J* = 8.0 Hz, 2H), 7.82 (d, *J* = 8.1 Hz, 2H), 7.63 (s, 1H); ^{13}C NMR (151 MHz, DMSO-d_6) δ 166.7, 138.1, 131.2 (qd, *J* = 32.1, 3.5 Hz), 128.3, 125.2 (q, *J* = 3.6 Hz), 124.0 (q, *J* = 271.8 Hz); ^{19}F NMR (565MHz, DMSO-d_6) δ -61.3.

4-methoxybenzamide (14)[34]

^{1}H NMR (600 MHz, CD_3CN) δ 7.71 (d, *J* = 8.5 Hz, 2H), 6.89 (d, *J* = 8.7 Hz, 2H), 6.57 (s, 1H), 5.84 (s, 1H), 3.75 (s, 3H); ^{13}C NMR (151 MHz, CD_3CN) δ 169.4, 163.4, 130.1, 126.9, 114.4, 56.0.

4-nitrobenzamide (15)[34]
^{1}H NMR (600 MHz, DMSO-d_6) δ 8.30 (d, *J* = 8.8 Hz, 2H), 8.27 (s, 1H), 8.09 (d, *J* = 8.8 Hz, 2H), 7.72 (s, 1H); ^{13}C NMR (151 MHz, DMSO-d_6) δ 166.3, 149.1, 140.0, 128.9, 123.5.

2-aminobenzamide (16)[37]
^{1}H NMR (600 MHz, CD_3CN) δ 7.43 (dd, *J* = 7.9, 1.3 Hz, 1H), 7.19 (ddd, *J* = 8.4, 7.2, 1.5 Hz, 1H), 6.70 (dd, *J* = 8.2, 0.9 Hz, 1H), 6.63-6.51 (m, 1H), 5.94 (s, 2H); ^{13}C NMR (151 MHz, CD_3CN) δ 172.4, 151.0, 133.4, 129.4, 117.7, 116.4, 114.8.

2-(methylamino)benzamide (17)[38]
^{1}H NMR (600 MHz, CD_3CN) δ 7.79 (s, 1H), 7.46 (dd, *J* = 7.9, 1.5 Hz, 1H), 7.37-7.29 (m, 1H), 6.68 (d, *J* = 8.4 Hz, 1H), 6.60-6.52 (m, 1H), 2.83 (d, *J* = 5.1 Hz, 3H); ^{13}C NMR (151 MHz, CD_3CN) δ 172.9, 152.1, 134.0, 129.6, 114.9, 114.4, 111.8, 29.8.

L-phenylalaninamide (18)[39]
^{1}H NMR (600 MHz, CD_3CN) δ 7.33-7.23 (m, 5H), 6.86 (s, 1H), 5.91 (s, 1H), 3.48 (dd, *J* = 8.7, 4.7 Hz, 1H), 3.06 (dd, *J* = 13.6, 4.7 Hz, 1H), 2.68 (dd, *J* = 13.6, 8.7 Hz, 1H); ^{13}C NMR (151 MHz, CD_3CN) δ 178.3, 139.4, 130.2, 129.2, 127.2, 57.1, 41.7; Chiral HPLC (Chiralcel OD-XF133, 20% *i*-PrOH in hexanes, 0.6 mL/min, 214 nm, t_1 = 12.3 min, t_2 = 15.3 min) 97% ee.

L-tryptophanamide (19)[39]

^{1}H NMR (600 MHz, CD_3CN) δ 9.19 (s, 1H), 7.61 (d, *J* = 7.9 Hz, 1H), 7.39 (d, *J* = 8.1 Hz, 1H), 7.15-7.11 (m, 2H), 7.07-7.02 (m, 1H), 6.91 (s, 1H), 5.75 (s, 1H), 3.55 (dd, *J* = 8.6, 4.4 Hz, 1H), 3.20 (dd, *J* = 14.4, 4.4 Hz, 1H), 2.84 (dd, *J* = 14.4, 8.6 Hz, 1H); ^{13}C NMR (151 MHz, CD_3CN) δ 178.5, 137.5, 128.5, 124.6, 122.4, 119.8, 119.6, 112.24, 112.22, 56.4, 31.7; Chiral HPLC (Chiralcel OD-XF133, 20% *i*-PrOH in hexanes, 0.6 mL/min, 214 nm, t_1 = 25.9 min, t_2 = 38.1 min) 96% ee.

2,6-dimethylbenzamide (20)[40]

^{1}H NMR (600 MHz, CD_3CN) δ 7.15 (t, *J* = 7.6 Hz, 1H), 7.04 (d, *J* = 7.7 Hz, 2H), 6.40 (s, 1H), 6.24 (s, 1H), 2.29 (s, 6H); ^{13}C NMR (151 MHz, CD_3CN) δ 172.7, 139.0, 134.5, 129.2, 128.1, 19.3.

4-bromo-3-methylbenzamide (21)[41]

^{1}H NMR (600 MHz, CD_3CN) δ 7.75 (s, 1H), 7.63 (d, *J* = 8.3 Hz, 1H), 7.51 (d, *J* = 8.0 Hz, 1H), 6.73 (s, 1H), 6.01 (s, 1H); ^{13}C NMR (151 MHz, CD_3CN) δ 168.7, 139.1, 134.3, 133.3, 130.9, 128.9, 127.4, 23.0.

2-furancarboxamide (22)[34]

^{1}H NMR (600 MHz, CD_3CN) δ 7.59 (s, 1H), 7.05 (s, 1H), 6.66 (s, 1H), 6.55 (s, 1H), 6.08 (s, 1H); ^{13}C NMR (151 MHz, CD_3CN) δ 160.6, 148.9, 145.8, 114.9, 112.9.

2-thiophenecarboxamide (23)[36]

^{1}H NMR (600 MHz, CD_3CN) δ 7.63-7.54 (m, 2H), 7.11 (dd, *J* = 5.0, 3.8 Hz, 1H), 6.70 (s, 1H), 6.02 (s, 1H); ^{13}C NMR (151 MHz, CD_3CN) δ 164.2, 140.2, 131.9, 129.7, 128.9.

Nicotinamide (24)[42]

^{1}H NMR (600 MHz, CD_3CN) δ 8.98 (d, *J* = 1.8 Hz, 1H), 8.70 (dd, *J* = 4.8, 1.6 Hz, 1H), 8.19-8.04 (m, 1H), 7.43 (ddd, *J* = 7.9, 4.8, 0.7 Hz, 1H), 6.84 (s, 1H), 6.13 (s, 1H). ^{13}C NMR (151 MHz, CD_3CN) δ 168.2, 153.1, 149.5, 136.1, 130.4, 124.3.

2-bromopyridine-4-formamide (25)[43]

^{1}H NMR (600 MHz, CD_3CN) δ 8.47 (d, *J* = 5.0 Hz, 1H), 7.88 (s, 1H), 7.67 (d, *J* = 5.0 Hz, 1H), 6.92 (s, 1H), 6.29 (s, 1H); ^{13}C NMR (151 MHz, CD_3CN) δ 166.4, 151.9, 145.1, 143.1, 127.0, 121.9.

Indole-2-carboxylic acid formamide (26)[44]

^{1}H NMR (600 MHz, DMSO-d_6) δ 11.52 (s, 1H), 7.59 (d, *J* = 7.9 Hz, 1H), 7.42 (d, *J* = 8.2 Hz, 1H), 7.35 (s, 1H), 7.17 (t, *J* = 7.6 Hz, 1H), 7.12 (s, 1H), 7.02 (t, *J* = 7.5 Hz, 1H); ^{13}C NMR (151 MHz, DMSO-d_6) δ 162.8, 136.5, 131.8, 127.2, 123.3, 121.5, 119.6, 112.3, 103.1.

1H-pyrazole-3-carboxylic acid, 5-(4-fluorophenyl)-1-(6-Cl-2- pyridinyl)-acetamide (27)

^{1}H NMR (600 MHz, CD_3CN) δ 8.51 (dd, *J* = 4.6, 1.4 Hz, 1H), 8.00 (dd, *J* = 8.1, 1.4 Hz, 1H), 7.55 (dd, *J* = 8.1, 4.7 Hz, 1H), 7.12 (s, 4H), 7.01 (s, 1H), 6.88 (s, 1H), 6.00 (s, 1H); ^{13}C NMR (151 MHz, CD_3CN) δ 163.9, 149.9, 148.7 (d, *J* = 2.9 Hz), 147.7, 147.5, 140.9, 140.2, 130.3, 130.2, 128.8, 127.8, 127.0, 106.9; ^{19}F NMR (565MHz, CD_3CN) δ -100.0.

2-naphthalamide (28)[34]

^{1}H NMR (600 MHz, DMSO-d_6) δ 8.49 (s, 1H), 8.16 (s, 1H), 8.00 (d, *J* = 7.7 Hz, 1H), 7.96 (d, *J* = 8.7 Hz, 3H), 7.59 (m, 2H), 7.48 (s, 1H); ^{13}C NMR (151 MHz, DMSO-d_6) δ 168.2, 134.3, 132.2, 131.7, 128.9, 127.9, 127.9, 127.7, 126.7, 124.5.

6-bromo-2-naphthamide (29)[45]

^{1}H NMR (600 MHz, DMSO-d_6) δ 8.51 (s, 1H), 8.25 (s, 1H), 8.17 (s, 1H), 8.01 (d, *J* = 8.6 Hz, 1H), 7.96 (dd, *J* = 8.3, 5.8 Hz, 2H), 7.69 (dd, *J* = 8.7, 1.8 Hz, 1H), 7.54 (s, 1H); ^{13}C NMR (151 MHz, DMSO-d_6) δ 167.7, 135.3, 132.2, 131.1, 130.7, 129.7, 129.6, 127.9, 127.1, 125.6, 120.9.

1-naphthalenecarboxamide (30)[34]

^{1}H NMR (600 MHz, DMSO-d_6) δ 8.31 (d, *J* = 7.8 Hz, 1H), 8.05-7.91 (m, 3H), 7.64 (dd, *J* = 7.0, 1.7 Hz, 1H), 7.55 (ddd, *J* = 18.5, 14.3, 6.8 Hz, 4H); ^{13}C NMR (151 MHz, DMSO-d_6) δ 170.6, 134.6, 133.2, 129.8, 129.7, 128.2, 126.6, 126.1, 125.6, 125.1, 124.9.

Formamide caproate (31)[46]

^{1}H NMR (600 MHz, CD_3CN) δ 6.13 (s, 1H), 5.75 (s, 1H), 2.11 (t, *J* = 7.5 Hz, 2H), 1.62-1.47 (m, 2H), 1.38-1.19 (m, 4H), 0.89 (t, *J* = 6.9 Hz, 3H); ^{13}C NMR (151 MHz, CD_3CN) δ 176.3, 36.1, 32.1, 26.0, 23.0, 14.2.

1-cyclohexylformamide (32)[34]

^{1}H NMR (600 MHz, DMSO-d_6) δ 7.13 (s, 1H), 6.61 (s, 1H), 2.05 (t, *J* = 11.0 Hz, 1H), 1.68 (d, *J* = 10.2 Hz, 4H), 1.60 (d, *J* = 10.8 Hz, 1H), 1.28 (dd, *J* = 22.6, 11.5 Hz, 2H), 1.17 (dt, *J* = 30.2, 11.9 Hz, 3H); ^{13}C NMR (151 MHz, DMSO-d_6) δ 177.3, 43.7, 29.2, 25.5, 25.3.

Octadecanamide (33)[35]

^{1}H NMR (600 MHz, CD_2Cl_2) δ 5.41 (s, 1H), 5.30 (s, 1H), 2.17 (t, *J* = 7.6 Hz, 2H), 1.63-1.53 (m, 2H), 1.28 (d, *J* = 21.5 Hz, 28H), 0.88 (t, *J* = 6.9 Hz, 3H); ^{13}C NMR (151 MHz, CD_2Cl_2) δ 175.6, 36.3, 32.5, 30.3, 30.2, 30.2, 30.2, 30.1, 29.9, 29.8, 26.1, 23.3, 14.4.

Oleamide (34)[47]

^{1}H NMR (600 MHz, DMSO-d_6) δ 7.19 (s, 1H), 6.65 (s, 1H), 5.32 (s, 2H), 1.99 (dt, *J* = 12.4, 7.0 Hz, 6H), 1.46 (s, 2H), 1.24 (s, 20H), 0.85 (s, 3H); ^{13}C NMR (151 MHz, DMSO-d_6) δ 174.2, 129.5, 35.1, 31.3, 29.2, 29.1, 28.9, 28.8, 28.7, 28.6, 26.6, 25.1, 22.1, 13.9.

2-hydroxybenzenepropanamide (35)[48]

^{1}H NMR (600 MHz, CD_3CN) δ 7.14-7.02 (m, 2H), 6.80 (dd, *J* = 11.0, 4.4 Hz, 2H), 6.37 (s, 1H), 5.97 (s, 1H), 2.81 (t, *J* = 6.7 Hz, 2H), 2.55 (t, *J* = 6.8 Hz, 2H); ^{13}C NMR (151 MHz, CD_3CN) δ 177.4, 155.9, 131.3, 129.1, 128.5, 120.9, 117.3, 36.4, 25.6.

5-hydroxypentanamide (36)[49]

^{1}H NMR (600 MHz, CD_3CN) δ 6.05 (s, 1H), 5.53 (s, 1H), 3.48 (t, *J* = 8.5 Hz, 2H), 2.14 (t, *J* = 7.5 Hz, 2H), 1.58 (dd, *J* = 18.7, 11.1 Hz, 2H), 1.48 (dq, *J* = 9.5, 6.5 Hz, 2H); ^{13}C NMR (151 MHz, CD_3CN) δ 176.1, 62.1, 35.7, 32.9, 22.5.

Phenylpropanamide (37)[50]

^{1}H NMR (600 MHz, CD_3CN) δ 7.38-7.10 (m, *J* = 14.7, 7.4 Hz, 5H), 6.09 (s, 1H), 5.65(s, 1H), 2.87 (t, *J* = 7.7 Hz, 2H), 2.44 (t, *J* = 7.8 Hz, 2H); ^{13}C NMR (151 MHz, CD_3CN) δ 174.9, 142.5, 129.3, 129.2, 126.9, 37.6, 32.0.

Cinnamamide (38, 39)[34]

^{1}H NMR (600 MHz, CD_3CN) δ 7.58 (d, *J* = 6.6 Hz, 2H), 7.52 (d, *J* = 15.8 Hz, 1H), 7.45-7.34 (m, 3H), 6.61 (d, *J* = 15.8 Hz, 1H), 6.36 (s, 1H), 5.94 (s, 1H); ^{13}C NMR (151 MHz, CD_3CN) δ 168.1, 141.3, 136.0, 130.6, 129.8, 128.7, 122.1.

4-hydroxymethyl benzoate (40)[51]
^{1}H NMR (600 MHz, CD_3CN) δ 7.41 (d, *J* = 8.8 Hz, 2H), 6.00 (d, *J* = 8.7 Hz, 2H), 3.61 (s, 3H), 2.50 (s, 1H); ^{13}C NMR (151 MHz, CD_3CN) δ 167.4, 162.1, 132.5, 122.8, 116.1, 52.3.

Methyl 4-(hydroxymethyl)benzoate (41)[52]
^{1}H NMR (600 MHz, CD_3CN) δ 7.96 (d, *J* = 8.2 Hz, 2H), 7.44 (d, *J* = 8.1 Hz, 2H), 4.65 (s, 2H), 3.86 (s, 3H); ^{13}C NMR (151 MHz, CD_3CN) δ 167.6, 148.5, 130.2, 127.3, 64.1, 52.5.

4-(hydroxymethyl)benzamide (42)[53]
^{1}H NMR (600 MHz, CD_3CN) δ 7.79 (d, *J* = 8.2 Hz, 2H), 7.41 (d, *J* = 8.1 Hz, 2H), 6.72 (s, 1H), 5.95 (s, 1H), 4.63 (s, 2H); ^{13}C NMR (151 MHz, CD_3CN) δ 169.6, 146.9, 133.5, 128.4, 127.3, 64.1.

***N*-methylbenzamide (43)**[54]
^{1}H NMR (600 MHz, CD_3CN) δ 7.77 (d, *J* = 7.7 Hz, 2H), 7.51 (s, 1H), 7.44 (s, 2H), 7.01 (s, 1H), 2.86 (d, *J* = 4.7 Hz, 3H); ^{13}C NMR (151 MHz, CD_3CN) δ 168.3, 135.9, 132.0, 129.4, 127.8, 26.6.

***N*-methyl-p-toluamide (or N,4-dimethylbenzamide 44)**[55]
^{1}H NMR (600 MHz, $CDCl_3$) δ 7.65 (d, *J* = 8.1 Hz, 2H), 7.20 (d, *J* = 7.8 Hz, 2H), 6.29 (s, 1H), 2.98 (d, *J* = 4.8 Hz, 3H), 2.37 (s, 3H); ^{13}C NMR (151 MHz, $CDCl_3$) δ 168.4, 141.8, 131.9, 129.3, 126.9, 26.9, 21.5.

***N*-methyl-3-methylbenzamide (45)**[54]
^{1}H NMR (600 MHz, $CDCl_3$) δ 7.58 (s, 1H), 7.54-7.50 (m, 1H), 7.28 (d, *J* = 4.6 Hz, 2H), 6.31 (s, 1H), 2.99 (d, *J* = 4.8 Hz, 3H), 2.37 (s, 3H); ^{13}C NMR (151 MHz, $CDCl_3$) δ 168.7, 138.5, 134.7, 132.2, 128.5, 127.7, 123.9, 26.9, 21.4.

***N*-methyl-2-methylbenzamide (46)**[56]
^{1}H NMR (600 MHz, $CDCl_3$) δ 7.36-7.27 (m, 2H), 7.23-7.16 (m, 2H), 5.79 (s, 1H), 2.99 (d, *J* = 4.9 Hz, 3H), 2.44 (s, 3H); ^{13}C NMR (151 MHz, $CDCl_3$) δ 171.0, 136.6, 136.1, 131.1, 129.9, 126.8, 125.8, 26.7, 19.8.

***N*-methyl-4-fluorobenzamide (47)**[54]
^{1}H NMR (600 MHz, $CDCl_3$) δ 7.85-7.67 (m, 2H), 7.04 (t, *J* = 8.0 Hz, 2H), 6.68 (s, 1H), 3.07-

2.83 (m, 3H); ^{13}C NMR (151 MHz, $CDCl_3$) δ 167.5, 164.7 (d, *J* = 251.6 Hz), 130.8 (d, *J* = 3.0 Hz), 129.3 (d, *J* = 8.9 Hz), 115.6 (d, *J* = 21.8 Hz), 26.9; ^{19}F NMR (565MHz, $CDCl_3$) δ -108.6.

3-fluoro-*N*-methylbenzamide (48)[54]
^{1}H NMR (600 MHz, $CDCl_3$) δ 7.56-7.44 (m, 2H), 7.43-7.31 (m, 1H), 7.21-7.09 (m, 1H), 6.30 (s, 1H), 3.00 (d, *J* = 4.8 Hz, 3H); ^{13}C NMR (151 MHz, $CDCl_3$) δ 167.2 (d, *J* = 2.2 Hz), 162.9 (d, *J* = 247.6 Hz), 137.0 (d, *J* = 6.7 Hz), 130.4 (d, *J* = 8.0 Hz), 122.4 (d, *J* = 3.0 Hz), 118.5 (d, *J* = 21.3 Hz), 114.4 (d, *J* = 22.9 Hz), 27.1; ^{19}F NMR (565MHz, $CDCl_3$) δ -111.9.

2-fluoro-*N*-methylbenzamide (49)[57]
^{1}H NMR (600 MHz, $CDCl_3$) δ 8.09 (td, *J* = 7.9, 1.8 Hz, 1H), 7.45 (tdd, *J* = 7.3, 5.3, 1.8 Hz, 1H), 7.26-7.20 (m, 1H), 7.10 (dd, *J* = 12.1, 8.3 Hz, 1H), 6.77 (s, 1H), 3.02 (dd, *J* = 4.8, 0.8 Hz, 3H); ^{13}C NMR (151 MHz, $CDCl_3$) δ 164.1 (d, *J* = 3.1 Hz), 160.7 (d, *J* = 246.9 Hz), 133.3 (d, *J* = 9.3 Hz), 132.1 (d, *J* = 2.2 Hz), 124.9 (d, *J* = 3.2 Hz), 121.1 (d, *J* = 11.7 Hz), 116.1 (d, *J* = 24.9 Hz), 26.9; ^{19}F NMR (565MHz, $CDCl_3$) δ -114.0.

4-chloro-*N*-methylbenzamide (50)[35]
^{1}H NMR (600 MHz, $CDCl_3$) δ 7.69 (d, *J* = 8.1 Hz, 2H), 7.36 (d, *J* = 8.1 Hz, 2H), 6.52 (s, 1H), 2.97 (s, 3H); ^{13}C NMR (151 MHz, $CDCl_3$) δ 167.4, 137.7, 133.1, 128.9, 128.4, 27.0.

4-bromo-*N*-methylbenzamide (51)[56]
^{1}H NMR (600 MHz, $CDCl_3$) δ 7.62 (d, *J* = 7.9 Hz, 2H), 7.53 (d, *J* = 7.8 Hz, 2H), 6.39 (s, 1H), 2.98 (s, 3H); ^{13}C NMR (151 MHz, $CDCl_3$) δ 167.5, 133.5, 131.9, 128.6, 126.1, 27.0.

4-iodo-*N*-methylbenzamide (52)[58]
^{1}H NMR (600 MHz, $CDCl_3$) δ 7.75 (d, *J* = 8.4 Hz, 2H), 7.47 (d, *J* = 8.4 Hz, 2H), 6.32 (s, 1H), 2.98 (d, *J* = 4.9 Hz, 3H); ^{13}C NMR (151 MHz, $CDCl_3$) δ 167.6, 137.9, 134.1, 128.6, 98.4, 27.0.

***N*-methyl-4-nitrobenzamide (53)**[59]
^{1}H NMR (600 MHz, CD_3CN) δ 8.30-8.22 (m, 2H), 7.99-7.91 (m, 2H), 7.19 (s, 1H), 2.89 (d, *J* = 4.7 Hz, 3H); ^{13}C NMR (151 MHz, CD_3CN) δ 166.6, 150.4, 141.5, 129.2, 124.5, 26.8.

4-methoxy-*N*-methylbenzamide (54)[56]
^{1}H NMR (600 MHz, $CDCl_3$) δ 7.73 (d, *J* = 8.0 Hz, 2H), 6.86 (d, *J* = 8.1 Hz, 2H), 6.50 (s, 1H), 3.80 (s, 3H), 2.94 (s, 3H); ^{13}C NMR (151 MHz, $CDCl_3$) δ 168.0, 162.1, 128.8, 127.0, 113.7, 55.4, 26.9.

***N*-methyl-4-(trifluoromethyl)benzamide (55)**[56]
^{1}H NMR (600 MHz, $CDCl_3$) δ 7.86 (d, *J* = 7.7 Hz, 2H), 7.66 (d, *J* = 7.7 Hz, 2H), 6.53 (s, 1H), 3.01 (s, 3H); ^{13}C NMR (151 MHz, $CDCl_3$) δ 167.2, 138.0, 133.2 (q, *J* = 32.8 Hz), 127.5, 125.7 (q, *J* = 3.7 Hz), 123.8 (q, *J* = 271.8 Hz), 27.1; ^{19}F NMR (565MHz, $CDCl_3$) δ -63.0.

4-cyano-*N*-methylbenzamide (56)[60]
^{1}H NMR (600 MHz, CD_3CN) δ 7.88 (d, *J* = 8.4 Hz, 2H), 7.80 (d, *J* = 8.5 Hz, 2H), 7.15 (s, 1H), 2.87 (d, *J* = 4.7 Hz, 3H); ^{13}C NMR (151 MHz, CD_3CN) δ 166.9, 139.8, 133.4, 128.6, 119.1, 115.2, 26.7.

4-bromo-*N*,3-dimethylbenzamide (57)[61]
^{1}H NMR (600 MHz, $CDCl_3$) δ 7.63 (s, 1H), 7.53 (d, *J* = 8.1 Hz, 1H), 7.39 (d, *J* = 8.0 Hz, 1H), 6.44 (s, 1H), 2.97 (s, 3H), 2.39 (s, 3H); ^{13}C NMR (151 MHz, $CDCl_3$) δ 167.7, 138.5, 133.8, 132.6, 129.5, 128.5, 125.6, 27.0, 23.0.

***N*-methyl-2-furancarboxamide (58)**[56]
^{1}H NMR (600 MHz, $CDCl_3$) δ 7.41 (d, *J* = 1.0 Hz, 1H), 7.09 (d, *J* = 3.1 Hz, 1H), 6.47 (dd, *J* = 3.5, 1.7 Hz, 1H), 6.41 (s, 1H), 2.97 (d, *J* = 5.0 Hz, 3H); ^{13}C NMR (151 MHz, $CDCl_3$) δ 159.2, 148.2, 143.9, 114.0, 112.2, 26.0.

***N*-methylnicotinamide (59)**[62]
^{1}H NMR (600 MHz, $CDCl_3$) δ 8.95 (d, *J* = 1.9 Hz, 1H), 8.68 (dd, *J* = 4.8, 1.6 Hz, 1H), 8.12 (dt, *J* = 7.9, 1.9 Hz, 1H), 7.37 (dd, *J* = 7.9, 4.8 Hz, 1H), 6.69 (s, 1H), 3.01 (d, *J* = 4.8 Hz, 3H); ^{13}C NMR (151 MHz, $CDCl_3$) δ 166.5, 152.1, 147.8, 135.4, 130.5, 123.7, 27.0.

6-bromo-*N*-methylnicotinamide (60)[63]
^{1}H NMR (600 MHz, $CDCl_3$) δ 8.69 (d, *J* = 1.7 Hz, 1H), 7.97 (dd, *J* = 8.2, 2.4 Hz, 1H), 7.57 (d, *J* = 8.2 Hz, 1H), 6.27 (s, 1H), 3.03 (d, *J* = 4.8 Hz, 3H); ^{13}C NMR (151 MHz, $CDCl_3$) δ 165.4, 148.2, 145.2, 137.7, 129.7, 128.4, 27.1.

***N*-methyl-2-thiophenecarboxamide (61)**[54]
^{1}H NMR (600 MHz, $CDCl_3$) δ 7.49 (d, *J* = 4.5 Hz, 1H), 7.45 (d, *J* = 5.0 Hz, 1H), 7.10-7.02 (m, 1H), 6.09 (s, 1H), 2.99 (d, *J* = 4.9 Hz, 3H); ^{13}C NMR (151 MHz, $CDCl_3$) δ 162.8, 139.0, 129.8, 128.1, 127.7, 26.9.

***N*-methyl-1H-indole-2-carboxamide (62)**[64]
^{1}H NMR (600 MHz, $CDCl_3$) δ 9.90 (s, 1H), 8.10 (d, *J* = 7.9 Hz, 1H), 7.90 (d, *J* = 8.1 Hz, 1H), 7.73 (dd, *J* = 16.0, 8.6 Hz, 1H), 7.60 (t, *J* = 7.4 Hz, 1H), 6.65 (s, 1H), 3.52 (d, *J* = 4.7 Hz, 3H); ^{13}C NMR (151 MHz, $CDCl_3$) δ 162.4, 136.3, 130.9, 127.8, 124.6, 122.0, 120.8, 112.1, 101.8, 26.6.

***N*-methyl-1-naphthamide (63)**[54]
^{1}H NMR (600 MHz, $CDCl_3$) δ 8.29 (d, *J* = 8.3 Hz, 1H), 7.91 (d, *J* = 8.3 Hz, 1H), 7.86 (d, *J* = 7.6 Hz, 1H), 7.59 (dd, *J* = 7.0, 1.0 Hz, 1H), 7.57-7.50 (m, 2H), 7.44 (dd, *J* = 8.1, 7.1 Hz, 1H), 6.02 (s, 1H), 3.09 (d, *J* = 4.9 Hz, 1H); ^{13}C NMR (151 MHz, $CDCl_3$) δ 170.4, 134.7, 133.8, 130.7, 130.3, 128.4, 127.2, 126.6, 125.6, 125.0, 124.9, 27.0.

***N*-methyl-2-naphthamide (64)**[59]
^{1}H NMR (600 MHz, $CDCl_3$) δ 8.27 (s, 1H), 7.90-7.79 (m, 4H), 7.53 (dt, *J* = 14.5, 6.9 Hz, 2H), 6.51 (s, 1H), 3.06 (s, 3H); ^{13}C NMR (151 MHz, $CDCl_3$) δ 168.5, 134.8 , 132.7, 131.9, 129.0, 128.5, 127.8, 127.7, 127.4, 126.8, 123.7, 27.1.

6-bromo-*N*-methyl-2-naphthamide (65)[65]
^{1}H NMR (600 MHz, $CDCl_3$) δ 8.25 (s, 1H), 8.04 (s, 1H), 7.81 (ddd, *J* = 19.8, 9.8, 5.2 Hz, 3H), 7.61 (dd, *J* = 8.7, 1.8 Hz, 1H), 6.28 (s, 1H), 3.08 (d, *J* = 4.8 Hz, 3H); ^{13}C NMR (151 MHz,

$CDCl_3$) δ 168.0, 135.8, 132.4, 131.2, 130.6, 130.4, 130.0, 127.7, 127.4, 124.8, 122.0, 27.2.

***N*-methyl-3-phenylacrylamide (66)**[66]
^{1}H NMR (600 MHz, $CDCl_3$) δ 7.61 (d, *J* = 15.6 Hz, 1H), 7.50-7.45 (m, 2H), 7.36-7.30 (m, 3H), 6.43 (d, *J* = 15.6 Hz, 1H), 6.03 (d, *J* = 43.8 Hz, 1H), 2.92 (t, *J* = 11.4 Hz, 3H); ^{13}C NMR (151 MHz, $CDCl_3$) δ 166.9, 140.9, 134.9, 129.7, 128.9, 127.9, 120.7, 26.6.

***N*-methyl-3-phenylpropanamide (67)**[59]
^{1}H NMR (600 MHz, $CDCl_3$) δ 7.27 (dd, *J* = 15.7, 7.6 Hz, 2H), 7.20 (dd, *J* = 15.1, 8.1 Hz, 3H), 5.43 (s, *J* = 91.2 Hz, 1H), 2.95 (t, *J* = 6.0 Hz, 2H), 2.76 (d, *J* = 4.8 Hz, 3H), 2.46 (t, *J* = 6.0 Hz, 2H); ^{13}C NMR (151 MHz, $CDCl_3$) δ 173.0, 141.0, 128.6, 128.4, 126.3, 38.5, 31.9, 26.4.

***N*-methyloctadecanamide (68)**[67]
^{1}H NMR (600 MHz, $CDCl_3$) δ 5.42 (s, 1H), 2.80 (d, *J* = 4.7 Hz, 3H), 2.15 (t, *J* = 13.5 Hz, 2H), 1.61 (dd, *J* = 14.2, 7.1 Hz, 2H), 1.33-1.19 (m, 28H), 0.88 (t, *J* = 7.0 Hz, 3H); ^{13}C NMR (151 MHz, $CDCl_3$) δ 174.0, 36.9, 32.1, 29.84, 29.82, 29.80, 29.76, 29.6, 29.51, 29.49, 26.4, 25.9, 22.8, 14.3.

***N*-methyloleamide (69)**[68]
^{1}H NMR (600 MHz, $CDCl_3$) δ 5.47 (s, 1H), 5.40-5.10 (m, 2H), 2.91-2.75 (m, 3H), 2.20-2.09 (m, 2H), 2.00 (s, 4H), 1.65-1.58 (m, 2H), 1.28 (d, *J* = 18.3 Hz, 20H), 0.87 (t, *J* = 6.9 Hz, 3H); ^{13}C NMR (151 MHz, $CDCl_3$) δ 174.4, 130.02, 129.76, 36.7, 34.6, 31.9, 29.8, 29.7, 29.5, 29.34, 29.32, 29.30, 29.2, 27.24, 27.20, 26.3, 25.9, 22.7, 14.1.

Supplementary note 3
Coordinates:

$PhCOOCH_3$

C	-2.11784400	-1.36912100	-0.00000800
C	-0.74552500	-1.17362300	-0.00000600
C	-0.22859100	0.12035900	0.00000000
C	-1.09310500	1.21272000	0.00000200
C	-2.46369500	1.01384100	-0.00000100
C	-2.97655300	-0.27739700	-0.00000600
H	-2.52038500	-2.37719200	-0.00001200
H	-0.06749900	-2.01968400	-0.00000700
H	-0.66771500	2.21124100	0.00000600
H	-3.13547400	1.86639100	0.00000100
H	-4.05113800	-0.43374200	-0.00000800
C	1.22948400	0.39360200	0.00000200
O	1.71924100	1.49305400	0.00000800
O	1.95867800	-0.73347400	0.00000100
C	3.36928100	-0.55013500	0.00000500
H	3.68756000	0.00205100	0.88787700
H	3.80303000	-1.54923700	0.00000400
H	3.68756500	0.00205400	-0.88786400

$NaNH_2BH_3$

H	-1.26066300	-1.15467700	0.80346200
N	-0.77712600	-0.76358700	-0.00000900
B	-0.88170300	0.82150100	0.00000100
H	-0.26087700	1.21621400	-1.00344600
H	-0.26075000	1.21619800	1.00338400
H	-1.26078500	-1.15468800	-0.80339800
H	-1.99844500	1.28815600	0.00008400
Na	1.35362900	-0.01578100	-0.00000300

M1

C	-3.01792000	0.26042500	-1.27754200
C	-1.76310700	0.73799900	-0.93607700
C	-1.16039600	0.31888500	0.24822000
C	-1.82213600	-0.57581400	1.08583900
C	-3.07982600	-1.04503200	0.74545700
C	-3.67652400	-0.62897300	-0.43809600
H	-3.48431600	0.58044300	-2.20369100
H	-1.23893500	1.42916800	-1.58644700
H	-1.34046400	-0.88716300	2.00731900
H	-3.59734200	-1.73711200	1.40200800
H	-4.66044800	-1.00036100	-0.70790800
C	0.19047800	0.77768400	0.63489500
O	0.83539300	0.29401300	1.54845000
O	0.62409500	1.78578600	-0.10371100
C	1.96119200	2.23745900	0.13384500
H	2.67880600	1.46075300	-0.15337700
H	2.08971000	3.11771000	-0.49351000
H	2.09393500	2.49849100	1.18603600
H	1.60790600	-0.24004600	-1.84098600
N	1.86442900	-1.08566500	-1.33710000
B	3.43822800	-1.20459900	-1.20511600
H	3.67058900	-2.27714000	-0.62080500
H	3.81349400	-0.28772000	-0.45295000
H	1.50172900	-1.83806400	-1.91627400
H	4.06888800	-1.16256700	-2.24007400
Na	2.28071200	-1.37890400	0.93352500

TS1

C	-2.87546700	0.05204100	-1.19897200
C	-1.53137300	0.34827900	-1.03885800
C	-0.91029100	0.12846000	0.18949000
C	-1.64587800	-0.38983900	1.25254500
C	-2.98921800	-0.68798300	1.08817200
C	-3.60569300	-0.46715500	-0.13682800
H	-3.35627500	0.22248300	-2.15719900
H	-0.94958300	0.74153800	-1.86706200
H	-1.14506800	-0.55199400	2.20169700
H	-3.55939500	-1.09313600	1.91833700
H	-4.65808000	-0.70118400	-0.26544000
C	0.52197100	0.43374200	0.40981100
O	1.06951900	0.36635500	1.49814500
O	1.12322300	1.21193400	-0.58480900
C	0.93472200	2.60168600	-0.35345200
H	1.33449600	2.90042100	0.62334800
H	1.46761800	3.13102000	-1.14415800
H	-0.12884700	2.86879500	-0.39764000
H	0.82007300	-1.28365100	-1.63392800
N	1.18942900	-1.47276600	-0.70490600
B	2.63422600	-2.09682200	-0.80354400
H	3.03245300	-2.31530000	0.34880800
H	3.37283000	-1.25959200	-1.34162700
H	0.54326500	-2.13961100	-0.28785700
H	2.68063100	-3.12906400	-1.43605600
Na	3.10128900	-0.12172200	0.52842700

M2

C	-2.97395400	-0.02807000	-1.13579800
C	-1.58829900	0.03497800	-1.11693500
C	-0.89167700	-0.08114200	0.08438200
C	-1.60134000	-0.25261600	1.26539900
C	-2.98882900	-0.31858400	1.24868100
C	-3.67752600	-0.20849900	0.04899300
H	-3.50835000	0.06966200	-2.07628100
H	-1.04175700	0.20305400	-2.04279900
H	-1.03537300	-0.32523700	2.18921100
H	-3.53545400	-0.45339400	2.17758600
H	-4.76224100	-0.25835700	0.03527100
C	0.63620400	-0.04671700	0.15012300
O	1.15264000	0.01932700	1.31415900
O	1.13932900	0.97340000	-0.78458800
C	0.75990300	2.27764300	-0.42102500
H	0.97251200	2.47709600	0.63932000
H	1.33012700	2.97302300	-1.04271200
H	-0.31079200	2.45780900	-0.59054800
H	0.74174200	-1.27523600	-1.59043300
N	1.12090300	-1.33302600	-0.64559300
B	2.69112300	-1.71895200	-0.74043800
H	3.08840100	-1.93299700	0.39108800
H	3.27596500	-0.79169400	-1.27405800
H	0.63780100	-2.10789300	-0.19106600
H	2.76583300	-2.71682700	-1.41080800
Na	3.24518200	0.30937800	0.72982500

TS2’

C	-2.82786800	-0.38416400	-1.14334700
C	-1.45722100	-0.55944400	-1.04127600
C	-0.82104800	-0.43180000	0.19325600

C	-1.57959200	-0.11915300	1.32013400
C	-2.94910100	0.05874500	1.21639500
C	-3.57622700	-0.07488300	-0.01541000
H	-3.31290200	-0.48040300	-2.10950100
H	-0.88709300	-0.76433000	-1.94279800
H	-1.07109400	-0.02112200	2.27370300
H	-3.53153500	0.30137800	2.09969300
H	-4.64959900	0.06516000	-0.09820300
C	0.64057200	-0.57828100	0.37847100
O	1.19329500	-0.47192000	1.45149600
O	1.21188300	1.30349000	-0.63466200
C	0.26741700	2.27505600	-0.85206700
H	-0.35662900	2.48415700	0.04362900
H	0.71801200	3.24284700	-1.14286200
H	-0.44538900	2.02105100	-1.66302600
H	1.38122900	-0.45732100	-1.45974600
N	1.37983900	-1.21027100	-0.75567500
B	2.92207300	-1.71715900	-0.52653900
H	2.95114700	-2.40406000	0.45923200
H	3.62888600	-0.72534500	-0.43995200
H	0.83952700	-1.99915600	-1.10892900
H	3.19211400	-2.30864300	-1.54308000
Na	2.89555200	0.93949600	0.69842300

M3

C	-2.91845900	1.43529200	0.11353300
C	-1.61478500	0.97216300	0.21987700
C	-1.32512800	-0.37024800	-0.02229800
C	-2.35570700	-1.23740400	-0.36207900
C	-3.65986400	-0.77250900	-0.47396000
C	-3.94323800	0.56520600	-0.23832800
H	-3.13539100	2.48068700	0.31216700
H	-0.82122600	1.65753400	0.51080300
H	-2.11134500	-2.28320700	-0.52129700
H	-4.45880400	-1.45880900	-0.73975800
H	-4.96266100	0.93029800	-0.32148800
C	0.10014600	-0.92402300	0.05100200
O	0.20043500	-2.20268500	0.09107900
O	0.80389700	-0.23426200	1.15226700
C	0.33210100	-0.62200500	2.42112900
H	0.36680100	-1.71163800	2.53729200
H	0.97409700	-0.15641200	3.17375000
H	-0.69993600	-0.28543800	2.58745300
H	0.82932100	0.73608600	-1.14036500
N	0.87272100	-0.31438700	-1.15874000
B	2.40080200	-0.75915800	-1.39825500
H	2.42496100	-1.80068000	-2.03643400
H	2.94553800	-0.90723600	-0.30919900
H	0.32527600	-0.59210700	-1.97265600
H	2.94596400	0.11489100	-2.02527200
Na	2.07914300	-3.13636100	-0.29267300
H	0.11989600	2.84156700	-1.25803800
N	1.09816900	2.64146200	-1.05785000
B	1.57822900	3.62094200	0.10248800
H	2.81033400	3.50054700	0.19428800
H	1.06821200	3.22060100	1.15844000
H	1.57998500	2.88877600	-1.92004100
H	1.30581100	4.78829100	-0.07266700
Na	2.38237800	1.37768300	0.62339900

TS2

C	-3.02958400	1.21737900	0.24892400
C	-1.69273400	0.86099200	0.35644700
C	-1.26976500	-0.41479200	-0.01373000
C	-2.20751000	-1.32583700	-0.48289500
C	-3.54536000	-0.96866100	-0.59836300
C	-3.95958300	0.30440800	-0.23409200
H	-3.34846200	2.21135300	0.54972700
H	-0.97280100	1.57612400	0.74863300
H	-1.86347400	-2.32354900	-0.73822900
H	-4.26886500	-1.69045200	-0.96703500
H	-5.00557200	0.58440500	-0.31847000
C	0.20206600	-0.84360600	0.05741500
O	0.39577300	-2.11937900	0.01773200
O	0.78566700	-0.20479800	1.27220700
C	0.32976500	-0.79382200	2.46416700
H	0.49557800	-1.87738200	2.45601600
H	0.88363900	-0.34751900	3.29524000
H	-0.74208400	-0.60822500	2.62117400
H	0.77920700	1.14120000	-1.04188400
N	0.94412100	-0.07212400	-1.03308100
B	2.49457800	-0.36745100	-1.26215700
H	2.66261500	-1.27493900	-2.06903300
H	3.02049800	-0.66897300	-0.19067500
H	0.45574000	-0.34260900	-1.88623500
H	3.01601600	0.64024200	-1.69258800
Na	2.29224800	-2.88006800	-0.57166000
H	-0.29998700	2.73908900	-1.27818300
N	0.69435200	2.54899100	-1.16817500
B	1.29283300	3.51052800	-0.04587200
H	2.52157500	3.39326400	-0.08089100
H	0.86791000	3.12232200	1.04514900
H	1.11171600	2.78511400	-2.06606700
H	0.99800800	4.67046300	-0.21283700
Na	2.33203200	1.38858400	0.81423900

P

C	2.87849600	1.10052000	-0.25365300
C	1.49276400	1.16171200	-0.25135700
C	0.73362900	0.01981000	-0.00204800
C	1.38865300	-1.18795100	0.22612700
C	2.77315900	-1.24857200	0.23628500
C	3.52200100	-0.10355600	-0.00261700
H	3.45789700	1.99530500	-0.45956500
H	1.00538900	2.10640500	-0.47764800
H	0.78642700	-2.07482700	0.39399700
H	3.27225400	-2.19387900	0.42719400
H	4.60680500	-0.15064900	-0.00105400
C	-0.76420700	0.02613200	0.00668800
O	-1.36558300	-1.04902200	-0.20767100
N	-1.39408900	1.16666600	0.25134800
B	-2.94164600	1.38850700	0.28309800
H	-3.44411300	0.67858900	1.16279200
H	-3.41909900	1.09024300	-0.81771300
H	-0.80007900	1.95048800	0.47997600
H	-3.17232100	2.54536100	0.52161400
Na	-3.54987400	-1.02478500	-0.22724500

CH_3OH

O	0.04616000	0.65584800	0.00000000
C	0.04616000	0.65584800	0.00000000
H	-0.43784400	1.07716400	0.89189000
H	1.08912300	0.97863100	0.00000000
H	-0.43784400	1.07716400	-0.89189000

H	-0.85967700	-1.06358900	0.00000000

CH_3ONa

O	0.00000000	0.00000000	-0.28942800
C	0.00000000	0.00000000	-1.64592300
H	0.00000000	1.02026900	-2.09126800
H	0.88357900	-0.51013500	-2.09126800
H	-0.88357900	-0.51013500	-2.09126800
Na	0.00000000	0.00000000	1.67861400

NH_3BH_3

H	0.00000000	0.94900900	1.09489600
N	0.00000000	0.00000000	0.72939400
B	0.00000000	0.00000000	-0.93405000
H	0.00000000	-1.16766400	-1.24006600
H	1.01122700	0.58383200	-1.24006600
H	-0.82186600	-0.47450500	1.09489600
H	-1.01122700	0.58383200	-1.24006600
H	0.82186600	-0.47450500	1.09489600

Supplementary Figure 18a

NH_3

H	0.00000000	0.94513300	-0.25706600
H	0.81850900	-0.47256600	-0.25706600
N	0.00000000	0.00000000	0.11017100
H	-0.81850900	-0.47256600	-0.25706600

TS

C	2.27026500	1.33968500	-0.12612000
C	0.92340700	1.08506500	-0.32854200
C	0.42243000	-0.20234500	-0.14709200
C	1.27702000	-1.22447000	0.25058000
C	2.62501500	-0.96586500	0.45621200
C	3.12405200	0.31395800	0.26350800
H	2.65514700	2.34618800	-0.25804400
H	0.24735900	1.89795700	-0.57647000
H	0.86454800	-2.21769100	0.39748800
H	3.28755200	-1.76757200	0.76790600
H	4.17829900	0.51738800	0.42493600
C	-1.00579300	-0.56315000	-0.38204500
O	-1.52440200	-1.60651400	-0.06908600
O	-1.90288800	0.91452200	0.39863300
C	-2.73192600	0.55193700	1.45064700
H	-2.34764900	0.92855700	2.40956600
H	-3.74915200	0.94821400	1.31784500
H	-2.81369400	-0.54718300	1.52019400
H	-2.13362300	0.79636000	-0.96281900
H	-0.91215100	0.52152800	-2.32013400
N	-1.58822100	0.09920700	-1.68872700
H	-2.16758100	-0.59115700	-2.15865500

P

C	1.44940200	-1.68610200	0.57984700
C	0.41645100	-0.76033400	0.61324300
C	0.61818000	0.52509100	0.11008000
C	1.85418400	0.86644500	-0.43522600
C	2.88610600	-0.05663200	-0.45525800
C	2.68475000	-1.33382900	0.05379900
H	1.28490400	-2.68974000	0.95902100
H	-0.55967000	-1.06480000	0.98539700
H	1.98314400	1.86593200	-0.83841600
H	3.85005800	0.21723600	-0.87236200
H	3.49212000	-2.05959200	0.03382100
C	-0.44409700	1.57171800	0.10683400
O	-0.42664100	2.52134100	-0.64292000
O	-2.89502500	-1.17752200	0.24952600
C	-2.66662200	-1.54705200	-1.08859500
H	-2.85839900	-2.61911800	-1.17353600
H	-3.33575700	-1.02673000	-1.78699900
H	-1.62784300	-1.36186800	-1.40118200
H	-2.69628400	-0.23709900	0.34323400
H	-1.29011900	0.87376400	1.85538900
N	-1.48465500	1.38187500	1.00241400
H	-2.06635500	2.20251200	1.11754500

Supplementary Figure 18b

$NaNH_2$

H	0.00000000	0.80107600	-1.71842800
H	0.00000000	-0.80107600	-1.71842800
N	0.00000000	0.00000000	-1.09102300
Na	0.00000000	0.00000000	1.00672900

M1

C	-2.63894100	-1.00716500	0.77186500
C	-1.28558400	-1.14673200	0.51792300
C	-0.64085100	-0.24810200	-0.32965000
C	-1.36486900	0.78354000	-0.92003200
C	-2.72228100	0.92030500	-0.66610900
C	-3.35978200	0.02609900	0.18158100
H	-3.13739200	-1.70273100	1.43993400
H	-0.70595500	-1.93135800	0.99084600
H	-0.85517200	1.46167700	-1.59847100
H	-3.28438500	1.72255300	-1.13439000
H	-4.42122900	0.13236000	0.38461200
C	0.80905700	-0.34830200	-0.61543300
O	1.44177200	0.52570300	-1.19341000
O	1.30538500	-1.54934900	-0.34814400
C	2.72035600	-1.64154000	-0.32656700
H	3.09349300	-1.01041200	0.48846500
H	2.95550000	-2.68769300	-0.13419300
H	3.15252200	-1.32483500	-1.27907600
H	0.45998100	0.62933500	2.30538600
H	1.99848800	0.30006200	2.56093800
N	1.37178300	0.67513800	1.84772900
Na	1.82379900	2.16778400	0.33571100

TS1

C	2.73286300	1.03855900	0.59915700
C	1.36557200	1.16774600	0.42728400
C	0.64942300	0.18266600	-0.25102200
C	1.32445400	-0.91988300	-0.76439400
C	2.69537400	-1.05013500	-0.58789200
C	3.40278400	-0.07251000	0.09592800
H	3.28400100	1.80769200	1.13215000
H	0.83306400	2.02203700	0.83062100
H	0.76310100	-1.66319800	-1.32294600
H	3.21424000	-1.91357400	-0.99393300
H	4.47529000	-0.17098600	0.23438700
C	-0.81868100	0.27637100	-0.46147000
O	-1.46148000	-0.56208200	-1.10003100
O	-1.27239700	1.53185300	-0.28891100
C	-2.66765800	1.69712400	-0.41223200
H	-3.18392900	1.12919000	0.37174500

H	-2.86582000	2.76124900	-0.28222300
H	-3.02492800	1.36599600	-1.39142600
H	-0.33848400	-0.62829000	2.15986400
H	-1.71421000	0.17442900	2.26706400
N	-1.24506700	-0.53360100	1.70006900
Na	-2.08733000	-2.07519400	0.39396300

M2

C	-2.89478500	-1.00822900	0.46519700
C	-1.50923100	-1.06842900	0.44097700
C	-0.76336500	0.00063700	-0.05142700
C	-1.42784000	1.12504800	-0.52256400
C	-2.81569500	1.18962300	-0.49541100
C	-3.55351800	0.12455300	0.00113400
H	-3.46633900	-1.85155600	0.84316600
H	-0.99734300	-1.96263400	0.78561900
H	-0.82699200	1.93421100	-0.92690800
H	-3.32414200	2.07335200	-0.87146100
H	-4.63859900	0.17115500	0.01972000
C	0.76470800	-0.00016000	-0.04643100
O	1.32491300	0.93792000	-0.74380400
O	1.12396400	-1.36558200	-0.42717500
C	2.42851500	-1.52392000	-0.87831600
H	3.17810000	-1.41891400	-0.06707600
H	2.52274700	-2.54236000	-1.26477900
H	2.67133500	-0.81679800	-1.68295100
H	0.63289200	0.74890400	1.89465300
H	1.06929800	-0.80952200	1.88103800
N	1.24936000	0.08432300	1.42681400
Na	3.04270700	1.29731400	0.48092400

M3

C	3.31998100	-1.35782300	-0.25574700
C	1.93848800	-1.23750700	-0.27860600
C	1.33195600	-0.01254200	-0.00816300
C	2.13152700	1.09316000	0.26945600
C	3.51130300	0.97129200	0.30548000
C	4.10834600	-0.25509000	0.04333500
H	3.78285100	-2.31401800	-0.47908600
H	1.33649200	-2.10295800	-0.54210900
H	1.64601400	2.04558900	0.45533900
H	4.12564400	1.83670400	0.53436100
H	5.18970800	-0.35033800	0.06502300
C	-0.15169800	0.17684100	-0.02686400
O	-0.61568300	1.31630100	-0.20640800
O	-3.48694700	-0.29670600	0.06295400
C	-4.38567600	-1.31669600	0.21589700
H	-5.05000400	-1.19100400	1.09527100
H	-5.05980400	-1.44517300	-0.65558700
H	-3.89719900	-2.30620800	0.35640100
H	-0.51007500	-1.77320900	0.39090500
N	-0.92839200	-0.88714100	0.15710500
H	-2.00175600	-0.76051000	0.14089000
Na	-2.82431400	1.64041200	-0.26384000

TS2

C	3.28656200	-1.36873200	-0.25239100
C	1.90583900	-1.23798700	-0.27154000
C	1.30753800	-0.00783500	-0.00682300
C	2.11726000	1.09274100	0.26189500
C	3.49648600	0.96133300	0.29483100
C	4.08446900	-0.27045200	0.03782200
H	3.74148400	-2.32992000	-0.47120600
H	1.29620900	-2.10104800	-0.52554700
H	1.63877200	2.04970400	0.44269000
H	4.11760300	1.82384600	0.51696200
H	5.16523900	-0.37347700	0.05679900
C	-0.17785700	0.19042800	-0.02174700
O	-0.62265800	1.34468900	-0.19650000
O	-3.42714100	-0.31089700	0.06136300
C	-4.28049700	-1.37827100	0.20555700
H	-4.94770400	-1.28407200	1.08303400
H	-4.93921200	-1.52793000	-0.67065900
H	-3.73698000	-2.33603800	0.34102300
H	-0.54187700	-1.74377100	0.36890500
N	-0.97089300	-0.85566400	0.15779700
H	-2.11209500	-0.69763200	0.13191500
Na	-2.81141800	1.66839200	-0.25300200

P

C	3.13812400	-1.52731700	0.13298300
C	1.78375400	-1.24361900	0.23305100
C	1.31125700	0.05395200	0.04673500
C	2.22557900	1.06025800	-0.25667800
C	3.58106800	0.78324600	-0.34649500
C	4.04182000	-0.51225000	-0.15060300
H	3.48916200	-2.54558600	0.27234400
H	1.08776300	-2.05330300	0.43677100
H	1.84105700	2.06109100	-0.42398100
H	4.28287700	1.58009500	-0.57522400
H	5.10260800	-0.73259600	-0.22600200
C	-0.14875200	0.41515800	0.14337800
O	-0.49678200	1.51647800	-0.36419200
O	-3.62908800	-0.41622300	-0.00304600
C	-4.35899700	-1.40648200	-0.67704000
H	-4.51265200	-2.29706900	-0.05381600
H	-5.34202800	-1.00017500	-0.92903500
H	-3.86972700	-1.71554000	-1.60987200
H	-0.55606900	-1.17084200	1.18967700
N	-1.01591100	-0.37888600	0.75261800
H	-2.70542300	-0.71152600	0.21621300
Na	-2.55839500	1.51799400	0.42004700

Supplementary Figure 18c

3THF • NaAB

N	-0.07723300	2.13314900	2.03604800
H	0.65666400	2.74607200	2.38175000
H	-0.93129800	2.54611000	2.40253900
B	0.11226300	0.66527000	2.58675200
H	1.14585400	0.20300300	2.07332200
H	0.18003200	0.55935500	3.79871800
H	-0.84562300	-0.00519800	2.16883100
Na	-0.04356700	0.89786700	0.02563100
C	-0.99187400	-2.14264300	0.06129400
O	0.07222500	-1.44465300	-0.59431500
C	1.32449500	-2.04104600	-0.23569900
C	0.99351900	-3.27867200	0.58078500
C	-0.34125400	-2.89158200	1.20362300
H	-1.73018900	-1.40150400	0.38950700
H	-1.46836600	-2.83039400	-0.65580700
H	1.88474300	-2.26256800	-1.15260600
H	1.89176900	-1.31308100	0.36161900
H	0.87336000	-4.15207700	-0.07213800
H	1.76751800	-3.50691500	1.31726400

H	-0.93108900	-3.74807700	1.53952100
H	-0.19029200	-2.20652100	2.04568300
C	3.02974300	1.77746900	0.17023600
O	2.13317900	1.24310800	-0.79807700
C	2.93616600	0.63401200	-1.79803800
C	4.19548600	0.11509000	-1.08859600
C	4.08931900	0.70391500	0.32656800
H	3.45916100	2.71949300	-0.20836300
H	2.45687500	1.97582100	1.07950500
H	2.33772200	-0.14953500	-2.27310800
H	3.19400600	1.38168500	-2.56339200
H	4.22978100	-0.97770500	-1.06791900
H	5.09740700	0.45828800	-1.60342000
H	3.73558400	-0.04674500	1.04107200
H	5.03796700	1.09965300	0.69719300
C	-3.18378600	1.19075100	0.47708800
O	-2.30632300	0.91221100	-0.62258600
C	-3.04080900	0.33236000	-1.70106800
C	-4.50420200	0.38992800	-1.29341900
C	-4.40800200	0.33632400	0.22593000
H	-2.64700700	0.95274000	1.40230500
H	-3.43540300	2.26151100	0.47438900
H	-2.82517000	0.88681200	-2.62087800
H	-2.70036600	-0.70413400	-1.83992300
H	-4.95240900	1.33997900	-1.60770000
H	-5.09213700	-0.42184700	-1.72815600
H	-5.29826400	0.71176600	0.73562400
H	-4.22773000	-0.69169100	0.56412500

M1

N	-1.59724400	-0.10897800	2.65737200
H	-1.74314700	-0.73969400	3.44226400
B	-0.14900900	0.43841600	2.63182400
H	-0.08820000	1.32392900	1.75637800
H	0.26549500	0.93362600	3.67780800
H	0.62928900	-0.49168200	2.33454000
Na	0.68039100	0.01961400	0.15436800
C	4.01320500	-0.09307600	0.39928800
O	2.88676600	0.72495000	0.65944800
C	3.08895400	1.23885200	1.97647800
C	3.74239000	0.10408400	2.76958700
C	4.19973200	-0.88619300	1.68343200
H	3.79628700	-0.69284100	-0.48983100
H	4.89152500	0.54519700	0.19340400
H	3.75323200	2.11702500	1.90878900
H	2.11715300	1.54374200	2.37419600
H	4.57974400	0.47177200	3.36928400
H	3.01564500	-0.35242000	3.44603700
H	5.22669800	-1.23588600	1.81870700
H	3.54330300	-1.76410000	1.67094500
C	0.13121900	3.03692500	-0.93957700
O	0.54781600	1.77582900	-1.45034500
C	1.80592400	1.99789300	-2.08277400
C	2.48773900	3.13819100	-1.31398800
C	1.39560800	3.64000900	-0.35785200
H	-0.26951800	3.65224400	-1.76328400
H	-0.66078700	2.85900800	-0.20575400
H	2.36002900	1.05393700	-2.05350500
H	1.63423100	2.27117800	-3.13506800
H	3.35979000	2.77593800	-0.76263100
H	2.81810400	3.92456300	-1.99925000
H	1.54960900	3.24241800	0.65078900
H	1.34992300	4.72978200	-0.29049000
C	1.69191900	-2.73491800	-0.76113800
O	1.62543000	-1.47166600	-1.42837000
C	0.83486200	-1.58910900	-2.61922600
C	0.18897100	-2.96107800	-2.56057100
C	1.22038600	-3.76046800	-1.77345800
H	2.72160300	-2.89160800	-0.42000400
H	1.02964800	-2.71152800	0.11967800
H	0.10902800	-0.76801600	-2.63711400
H	1.50075900	-1.48888000	-3.48855600
H	-0.75062000	-2.90123200	-2.00180600
H	-0.01439900	-3.37053400	-3.55297400
H	0.81122500	-4.65754600	-1.30163600
H	2.05033100	-4.06443000	-2.42282400
C	-5.31755200	1.08476100	0.50761800
C	-4.48024800	0.00382200	0.72807700
C	-3.24123900	-0.05679600	0.09450900
C	-2.85149600	0.96789100	-0.76309900
C	-3.69151500	2.04989800	-0.98031900
C	-4.92470600	2.10979100	-0.34558600
H	-6.28057800	1.13324400	1.00657200
H	-4.76855300	-0.79318300	1.40457700
H	-1.88484500	0.90586900	-1.25748900
H	-3.38387800	2.84850400	-1.64929500
H	-5.58228600	2.95746600	-0.51399000
C	-2.30766300	-1.18480400	0.30738500
O	-1.19948900	-1.24566600	-0.19421100
O	-2.85928400	-2.21423600	0.94842000
C	-1.95140300	-3.22966800	1.34465800
H	-1.17065500	-2.79492800	1.97818300
H	-2.53537700	-3.96025000	1.90362800
H	-1.48956600	-3.70706500	0.47390400
H	-2.26922400	0.64166300	2.79685600

TS1

N	-1.62780700	-0.30538400	2.38883900
H	-1.79526000	-1.06952800	3.04026900
B	-0.17518200	0.26144000	2.51303300
H	-0.07811800	1.22003900	1.73982000
H	0.08404700	0.64026700	3.64295700
H	0.61770600	-0.63114900	2.20274200
Na	0.79251800	0.02092900	0.02945600
C	4.01633600	-0.39819600	0.40574900
O	3.02258000	0.59184600	0.62303500
C	3.16803000	0.99434300	1.98650400
C	3.62769300	-0.25134700	2.75441100
C	3.93948300	-1.26983300	1.64646100
H	3.78292900	-0.90519000	-0.53502300
H	5.00584900	0.08269200	0.31848600
H	3.91785400	1.79992300	2.03710200
H	2.20254900	1.38007300	2.32636300
H	4.51131600	-0.03233500	3.36091700
H	2.83885300	-0.60968100	3.42000200
H	4.85614400	-1.83771600	1.82566300
H	3.11325100	-1.98346400	1.54257200
C	0.23814300	3.16407500	-0.68746300
O	0.66031000	1.95354200	-1.30444200
C	1.90896100	2.23702200	-1.92975100
C	2.59172600	3.31079900	-1.07200300
C	1.50418200	3.72651600	-0.07007000
H	-0.17617700	3.84125300	-1.45323100
H	-0.54416800	2.91895200	0.03668900

H	2.46989500	1.29809000	-1.98776100
H	1.72416200	2.59818400	-2.95265600
H	3.46931100	2.90814000	-0.56047100
H	2.91525200	4.15339100	-1.69018200
H	1.66939000	3.25048100	0.90246200
H	1.45338300	4.80672600	0.08696700
C	1.52207700	-2.77991600	-1.01556500
O	1.59614800	-1.47642700	-1.60555900
C	0.72258800	-1.40840900	-2.74250300
C	-0.12181500	-2.66784700	-2.69741400
C	0.83486200	-3.65752200	-2.04313300
H	2.53933400	-3.10323300	-0.76634400
H	0.92688100	-2.71860700	-0.09030800
H	0.12974000	-0.48836700	-2.67180900
H	1.33894300	-1.35975500	-3.65183700
H	-0.99071800	-2.49934500	-2.05215300
H	-0.46055300	-2.97968500	-3.68838200
H	0.33225000	-4.51569100	-1.58947500
H	1.56246900	-4.03372700	-2.77290900
C	-5.28256700	1.12689400	0.48224800
C	-4.41832800	0.08918700	0.79743700
C	-3.13810300	0.05226200	0.24989100
C	-2.74020800	1.06013800	-0.62068600
C	-3.60388500	2.10179600	-0.93150500
C	-4.87687200	2.13861000	-0.38019400
H	-6.28054500	1.14705600	0.91037200
H	-4.73001300	-0.70419300	1.46949300
H	-1.74830800	1.01293000	-1.06276700
H	-3.28212600	2.88464700	-1.61317900
H	-5.55488800	2.95114400	-0.62404700
C	-2.15693600	-1.03561400	0.57599300
O	-1.10358400	-1.15804000	-0.06457800
O	-2.81590000	-2.15930500	1.02319300
C	-1.98516300	-3.28125300	1.20901100
H	-1.15843700	-3.05642100	1.89676100
H	-2.61204800	-4.06784200	1.63207300
H	-1.55423800	-3.62367300	0.26096700
H	-2.32072200	0.40271500	2.62110300

M2

N	-1.60115900	-0.63836000	2.29610700
H	-1.47439200	-1.54562000	2.74347300
B	-0.26417500	0.21804600	2.49709000
H	-0.31282200	1.19565300	1.76235000
H	-0.19383700	0.55411500	3.65897200
H	0.67026100	-0.51706600	2.22008200
Na	0.77900800	-0.01374400	-0.00999100
C	3.94261900	-0.53076400	0.46647000
O	3.01578600	0.52993100	0.65995000
C	3.15381600	0.92960500	2.02446000
C	3.56348900	-0.32650000	2.80587600
C	3.77994400	-1.38127400	1.71172400
H	3.68770300	-1.02810400	-0.47366000
H	4.96485200	-0.12181200	0.39488200
H	3.92311700	1.71473100	2.08814700
H	2.19524700	1.34457400	2.34983300
H	4.48102100	-0.14813200	3.37454100
H	2.78197400	-0.62519700	3.50855100
H	4.63735700	-2.03199000	1.90163800
H	2.88893900	-2.01270000	1.60812100
C	0.24023200	3.11275000	-0.75112600
O	0.73435600	1.92318800	-1.35730900
C	2.02524100	2.24302100	-1.87101900
C	2.60779500	3.32128300	-0.94691300
C	1.44042800	3.67357200	-0.01391200
H	-0.11396100	3.80415700	-1.53406200
H	-0.60399100	2.84122600	-0.11075200
H	2.61339400	1.31962300	-1.88829600
H	1.91939100	2.61154000	-2.90218100
H	3.46709500	2.94179900	-0.38897500
H	2.93618000	4.19041200	-1.52459200
H	1.54248600	3.15998400	0.94884800
H	1.35419100	4.74482100	0.18355500
C	1.36795500	-2.84002900	-1.06440100
O	1.60380900	-1.53255600	-1.61239800
C	0.79799600	-1.34719800	-2.78449800
C	-0.22964800	-2.46088500	-2.75996300
C	0.56355000	-3.58906200	-2.11080400
H	2.33832600	-3.29937600	-0.84213600
H	0.78701400	-2.73051000	-0.13524100
H	0.35745700	-0.34275000	-2.74490200
H	1.44619300	-1.40693300	-3.67160300
H	-1.06447900	-2.17095600	-2.11194100
H	-0.60202400	-2.70868300	-3.75708700
H	-0.06817400	-4.36345600	-1.66783900
H	1.22912200	-4.06547600	-2.84166400
C	-4.94494600	1.55227300	0.63306500
C	-4.16185400	0.47727900	1.02606600
C	-2.96907000	0.18921200	0.36649900
C	-2.58718300	0.98339600	-0.70532500
C	-3.36956200	2.06037600	-1.10380500
C	-4.54807000	2.35139300	-0.43226700
H	-5.87461700	1.76348300	1.15359200
H	-4.49702400	-0.16536100	1.83642200
H	-1.67123700	0.73590000	-1.23679000
H	-3.05725600	2.67194700	-1.94656800
H	-5.16171500	3.19221200	-0.74201500
C	-2.08016800	-0.98582400	0.77371400
O	-1.07068800	-1.23840100	0.04198300
O	-2.98850200	-2.08114800	0.99889700
C	-2.39361700	-3.34578400	0.90969600
H	-1.58513700	-3.49122300	1.64658800
H	-3.17910900	-4.07878900	1.10824600
H	-1.96649200	-3.52735100	-0.08415700
H	-2.38691600	-0.20436600	2.77812200

TS2’

N	0.86565100	0.05282500	-1.90839400
H	1.12480200	-0.98716800	-2.03377400
B	-0.71602100	0.32208600	-2.07836600
H	-0.98295200	1.39155700	-1.55486200
H	-0.92964900	0.34713700	-3.26756800
H	-1.30022900	-0.60445800	-1.55152100
Na	-1.54466300	0.25002800	0.60051200
C	-0.28681400	-1.78741000	2.54606700
O	-1.29760300	-1.86433700	1.53256500
C	-0.98397100	-2.94176900	0.62515800
C	0.13929700	-3.72041800	1.27753500
C	0.87010600	-2.61847300	2.03337400
H	-0.02601700	-0.73161700	2.70047100
H	-0.69480900	-2.18730700	3.48659200
H	-1.89727400	-3.52756800	0.46756200
H	-0.63902100	-2.51965800	-0.32883400
H	-0.25458200	-4.47528400	1.97046000

H	0.76943900	-4.21112000	0.53268000
H	1.51132400	-2.98794500	2.83804900
H	1.46543200	-2.03916000	1.31681800
C	-4.17140400	0.63034700	-1.31226700
O	-3.79489500	0.13901000	-0.02642200
C	-4.36190500	-1.16201200	0.08524100
C	-4.14043400	-1.78678600	-1.28008400
C	-4.27618700	-0.59103800	-2.23420800
H	-5.13583200	1.15296100	-1.22461800
H	-3.40804700	1.34851000	-1.63007800
H	-3.85786400	-1.67771000	0.90847100
H	-5.43553700	-1.07545800	0.31998500
H	-3.12844100	-2.20140000	-1.33755100
H	-4.85022000	-2.58998400	-1.49374800
H	-3.48019500	-0.59057700	-2.98239300
H	-5.23570000	-0.59832400	-2.75935800
C	-0.14687200	3.00976600	1.66118000
O	-1.41651300	2.37790800	1.54174900
C	-2.25787300	3.33812900	0.91513300
C	-1.38431100	4.03399100	-0.13045600
C	0.05287000	3.73699200	0.33841200
H	-0.17511000	3.71457200	2.50782500
H	0.59787200	2.23474100	1.85851800
H	-3.12291200	2.80776500	0.50411700
H	-2.61833500	4.05049000	1.67367500
H	-1.55746500	3.61287900	-1.12502600
H	-1.59829600	5.10511100	-0.17714500
H	0.55781200	3.07765300	-0.37542700
H	0.66027300	4.63819800	0.45305000
C	5.13159900	0.63803500	-1.46894400
C	3.77272700	0.40663500	-1.61760900
C	2.92771000	0.42145200	-0.50790300
C	3.47656200	0.64976600	0.75509500
C	4.83533100	0.86910500	0.90350000
C	5.66647700	0.87121200	-0.20991200
H	5.77784700	0.62209400	-2.34089400
H	3.38838600	0.18096800	-2.60803500
H	2.81441300	0.64084800	1.61596400
H	5.25066200	1.04004700	1.89196300
H	6.73189900	1.04509700	-0.09497400
C	1.46769700	0.19182900	-0.57998900
O	0.74513100	0.29819900	0.38948500
O	1.74181600	-2.03421600	-1.02726000
C	2.87741500	-2.78608500	-0.95292900
H	3.06376600	-3.19371600	0.06585300
H	2.85757900	-3.66364700	-1.63055000
H	3.80429300	-2.22672200	-1.22160600
H	1.39292100	0.59556900	-2.58845400

P’

N	-1.12380800	0.16316500	1.80089400
H	-0.31122300	-2.07959100	1.65102600
B	0.41263500	0.23493100	2.10615600
H	0.87226100	1.27678700	1.64501500
H	0.56639500	0.19707800	3.30478800
H	0.98877300	-0.71995000	1.57880100
Na	1.31362100	0.36279300	-0.49523800
C	0.58128500	-1.71044500	-2.64989100
O	1.64073300	-1.63509600	-1.69294800
C	1.72029100	-2.87854600	-0.97307400
C	0.62960500	-3.77679300	-1.53501100
C	-0.35587300	-2.76818700	-2.11215000
H	0.12046400	-0.71910700	-2.73109300
H	0.99213700	-1.99173800	-3.63198400
H	2.72815000	-3.29410600	-1.09639900
H	1.54324100	-2.67372600	0.09012600
H	1.02464000	-4.42226800	-2.32901800
H	0.19413700	-4.40433900	-0.75438900
H	-1.01052100	-3.18723600	-2.88060700
H	-0.97037500	-2.33212900	-1.31619600
C	3.88234100	1.14435600	1.43610000
O	3.58167400	0.65583200	0.13193800
C	4.37918200	-0.51260000	-0.01700200
C	4.26240100	-1.23500100	1.31924200
C	4.00798000	-0.09419100	2.32264900
H	4.82820500	1.70824600	1.39372400
H	3.07592900	1.81837200	1.73848800
H	3.99798700	-1.07215300	-0.87599500
H	5.42096200	-0.21575300	-0.22121600
H	3.41035700	-1.92241100	1.30536300
H	5.15798200	-1.81804400	1.54945100
H	3.07941900	-0.26345000	2.87437200
H	4.81925300	0.01629200	3.04713600
C	-0.19606900	2.96767300	-1.63769900
O	1.13259000	2.47467400	-1.50969200
C	1.85199700	3.51673500	-0.86488800
C	0.90480900	4.05764500	0.20599200
C	-0.49108200	3.65128900	-0.30752100
H	-0.23345100	3.68301100	-2.47531000
H	-0.84879000	2.11802400	-1.85219000
H	2.78413400	3.09193200	-0.48135700
H	2.10161200	4.29350900	-1.60558200
H	1.10496200	3.58548700	1.17263200
H	1.01325200	5.13852100	0.32964400
H	-0.96036100	2.94282100	0.38290300
H	-1.16650300	4.50239200	-0.42657900
C	-5.40577700	0.05606200	1.33092300
C	-4.03393200	-0.03713400	1.50833800
C	-3.16034300	0.25328500	0.46067800
C	-3.69295400	0.62551600	-0.77151600
C	-5.06410300	0.72891200	-0.94888300
C	-5.92442500	0.44524800	0.10296800
H	-6.07350300	-0.18119500	2.15347000
H	-3.65190300	-0.36682500	2.47103800
H	-3.00566400	0.82569700	-1.58743000
H	-5.46451200	1.02800200	-1.91288500
H	-6.99883100	0.52075200	-0.03451000
C	-1.66565500	0.17931900	0.58490300
O	-0.98078700	0.14699000	-0.45308600
O	-0.51264200	-2.98890400	1.38552200
C	-1.90189900	-3.15353800	1.30392300
H	-2.09644100	-4.18217500	0.98498500
H	-2.40353200	-2.99986800	2.27093800
H	-2.36634000	-2.47850000	0.56779300
H	-1.76786500	0.28449900	2.56928400

M3

N	0.01250000	-0.81239200	1.00394900
H	-0.49293900	-1.54071800	0.49224000
B	1.27174000	-1.46471700	1.75158300
H	1.98173000	-0.55092200	2.15103400
H	0.84599200	-2.12854300	2.67909600
H	1.88073400	-2.19683100	0.96884000
Na	3.29546700	-0.67525000	-0.02072900

C	4.40424100	-1.71051800	-2.84676400
O	4.13913100	-2.28849300	-1.57116800
C	3.09626100	-3.23056800	-1.81536800
C	2.12703600	-2.50522200	-2.74567800
C	3.03005200	-1.49570300	-3.48432100
H	4.97830600	-0.79141200	-2.68221400
H	5.01613000	-2.41679300	-3.43579100
H	3.52993300	-4.12948100	-2.28966800
H	2.66882500	-3.51408000	-0.84487100
H	1.60528100	-3.19667400	-3.42008100
H	1.38583100	-1.95268800	-2.15067200
H	3.05843400	-1.65410100	-4.56945400
H	2.66999000	-0.47424000	-3.29963700
C	4.69645400	0.47865900	2.61639800
O	5.02579600	-0.44813300	1.57077200
C	5.37085100	-1.70283800	2.15824400
C	4.53281100	-1.78244800	3.41865100
C	4.57137200	-0.33403700	3.90900000
H	5.48594700	1.24456900	2.67552400
H	3.75170600	0.97156500	2.34040400
H	5.15961300	-2.48374800	1.41545600
H	6.45196400	-1.72006200	2.38442200
H	3.50404900	-2.07661300	3.17093100
H	4.93292700	-2.49322800	4.15145200
H	3.67317200	-0.06189500	4.47463800
H	5.44348000	-0.16745200	4.55653400
C	3.63111000	1.91925000	-2.12989400
O	4.32426100	1.09183000	-1.19612600
C	5.53389100	1.76912800	-0.89192800
C	5.11168200	3.22116600	-0.72064200
C	3.95761800	3.36708300	-1.73115400
H	4.00563900	1.69393100	-3.14572000
H	2.56847500	1.64673800	-2.06592100
H	5.96361200	1.29592800	-0.00102300
H	6.24119800	1.65358500	-1.73591700
H	4.75134800	3.38039100	0.30563800
H	5.93281800	3.92583800	-0.89752100
H	3.08948700	3.86259300	-1.27901000
H	4.25453700	3.95834500	-2.60623500
C	-0.56995600	3.37614900	2.07297400
C	-0.67505200	2.16518000	1.39601700
C	0.42795000	1.61220300	0.74111200
C	1.63889700	2.29900100	0.78694900
C	1.75342000	3.50877000	1.47023400
C	0.64701100	4.05476800	2.11428200
H	-1.44492400	3.78802700	2.57732800
H	-1.62897800	1.63728300	1.38941500
H	2.50079400	1.87423700	0.27081700
H	2.71293500	4.02858400	1.49971400
H	0.73215700	5.00086400	2.64792600
C	0.32934500	0.27669200	-0.02077200
O	1.32581500	-0.01441300	-0.78308200
O	-0.96618400	0.32420900	-0.77309900
C	-0.82208500	1.01229100	-1.99123500
H	-0.14625500	0.47906400	-2.67807400
H	-1.82147500	1.09752500	-2.43835400
H	-0.41734300	2.02869800	-1.83409000
H	-0.65297800	-0.48972500	1.76832200
N	-1.80717500	-0.32084600	3.14952700
H	-1.57063500	-1.09935700	3.76323600
H	-1.72440600	0.51568500	3.72501400
B	-3.21647400	-0.48369500	2.52297100
H	-3.19039500	-1.49025700	1.78714900
H	-4.15794700	-0.62125600	3.31033200
H	-3.45463600	0.52454200	1.82336500
Na	-3.18926900	-0.43605600	-0.21614600
C	-6.25481600	0.37286200	0.51943800
O	-5.59746500	-0.75246200	-0.07873300
C	-6.02110200	-1.95459600	0.57488800
C	-7.30474200	-1.59459800	1.29856500
C	-6.99640200	-0.16527300	1.73191300
H	-5.49665000	1.12400700	0.77999300
H	-6.94448500	0.80593300	-0.22632400
H	-6.13283400	-2.73308100	-0.19156200
H	-5.24410800	-2.25916500	1.29659300
H	-8.16106400	-1.61898300	0.60775300
H	-7.51728000	-2.26801200	2.13701900
H	-7.88787600	0.42428300	1.97717100
H	-6.32369500	-0.17228200	2.60179700
C	-1.34665900	-2.92887000	-1.63910200
O	-2.51400800	-2.62635100	-0.87636200
C	-2.77496200	-3.77640200	-0.07668600
C	-1.40580600	-4.21014800	0.41952800
C	-0.47758500	-3.83249300	-0.74917400
H	-1.64689700	-3.44691900	-2.56631200
H	-0.87399300	-1.97276200	-1.90241300
H	-3.46448600	-3.47777600	0.72139800
H	-3.25030200	-4.55372900	-0.70296700
H	-1.14275300	-3.64633000	1.32645700
H	-1.36664700	-5.27644600	0.67098900
H	0.41985800	-3.31137700	-0.38787000
H	-0.13629400	-4.71554700	-1.30555900
C	-3.58087000	2.81488900	-0.57848200
O	-3.90071300	1.58300000	-1.23612900
C	-4.68555400	1.83422700	-2.40158000
C	-4.64209500	3.33952200	-2.60916000
C	-4.52908700	3.83944800	-1.17257700
H	-3.69395300	2.66222900	0.50460300
H	-2.52562800	3.06664900	-0.77974400
H	-4.26376600	1.26116900	-3.23939500
H	-5.71213800	1.47356700	-2.22219200
H	-3.74322600	3.62107400	-3.17825100
H	-5.51886500	3.72004000	-3.14558000
H	-4.15265600	4.86521000	-1.08936100
H	-5.50860400	3.79047800	-0.67296500

TS2

N	-0.04638500	-0.77846300	0.94913300
H	-0.52871400	-1.50257700	0.41164300
B	1.17085900	-1.43944400	1.71134700
H	1.88029700	-0.54295400	2.16630500
H	0.72869600	-2.12788800	2.62576400
H	1.83856700	-2.18038500	0.97273500
Na	3.23738200	-0.69044100	-0.01762000
C	4.39894200	-1.67824500	-2.84966100
O	4.12055100	-2.27725100	-1.58691400
C	3.07426900	-3.20833300	-1.85457000
C	2.11529400	-2.45955600	-2.77646400
C	3.03212200	-1.45223800	-3.50055300
H	4.97108600	-0.76206900	-2.66307700
H	5.01748300	-2.37476500	-3.44364700
H	3.50519500	-4.10125600	-2.34324400
H	2.64004100	-3.50648800	-0.89146100
H	1.58543500	-3.13538100	-3.46051700

H	1.38205500	-1.90272500	-2.17475700
H	3.07203300	-1.60666600	-4.58605400
H	2.67436100	-0.43011700	-3.31564500
C	4.63226100	0.44197500	2.63498100
O	4.97233300	-0.47833300	1.58732500
C	5.28497800	-1.74463000	2.16749900
C	4.42615800	-1.82055500	3.41397900
C	4.49197000	-0.37903300	3.92007200
H	5.42135800	1.20724800	2.70726000
H	3.68990600	0.93635400	2.35354000
H	5.07096900	-2.51483100	1.41448000
H	6.36210700	-1.78437100	2.41017800
H	3.39461800	-2.08584900	3.14505400
H	4.79851300	-2.54900700	4.14422400
H	3.59917600	-0.09437300	4.48833700
H	5.36770100	-0.23613100	4.56879400
C	3.64800400	1.91447100	-2.07932100
O	4.33424100	1.07847500	-1.14745800
C	5.54565600	1.74737800	-0.83739800
C	5.13169500	3.20154400	-0.65983900
C	3.97705300	3.35813100	-1.66826800
H	4.02921800	1.69613500	-3.09450100
H	2.58446300	1.64256700	-2.02160200
H	5.97094900	1.26764600	0.05192800
H	6.25473900	1.63292400	-1.68043400
H	4.77335900	3.35929800	0.36742400
H	5.95684600	3.90221300	-0.83474200
H	3.10974600	3.85029200	-1.21068200
H	4.27373800	3.95724700	-2.53819900
C	-0.58408400	3.41598200	2.03420100
C	-0.69306800	2.21119500	1.34613500
C	0.41444000	1.62579500	0.72755400
C	1.63764400	2.28667300	0.81948800
C	1.76000700	3.48844600	1.51598000
C	0.64817700	4.06192800	2.12575900
H	-1.46754000	3.85156400	2.50361200
H	-1.66144100	1.71343800	1.29031400
H	2.50535300	1.84794200	0.32514700
H	2.73183900	3.98199800	1.58082200
H	0.73938900	5.00291100	2.66737900
C	0.30605800	0.28700300	-0.03897100
O	1.31141700	0.02682600	-0.81581700
O	-0.98424300	0.38858300	-0.82557100
C	-0.80621800	1.09214800	-2.02591300
H	-0.13035100	0.55855500	-2.71345100
H	-1.79600700	1.20665100	-2.49083000
H	-0.38192700	2.09805500	-1.84830200
H	-0.85286500	-0.47209500	1.97414800
N	-1.60546800	-0.36654500	2.99156800
H	-1.32398600	-1.12706500	3.60964600
H	-1.39692900	0.49778600	3.49000100
B	-3.11204900	-0.48506700	2.54462000
H	-3.19693200	-1.50400900	1.85075900
H	-3.89472100	-0.56014500	3.48117600
H	-3.37954000	0.51592200	1.86675400
Na	-3.13836300	-0.43403500	-0.23005400
C	-6.17347400	0.35681100	0.61693900
O	-5.53185300	-0.76520800	-0.00396000
C	-5.98718300	-1.97560600	0.61076200
C	-7.24978800	-1.60336800	1.36529000
C	-6.89750800	-0.19514800	1.83263800
H	-5.40743300	1.10162100	0.87083200
H	-6.87464500	0.80312200	-0.11013500
H	-6.13087200	-2.72444100	-0.17921300
H	-5.21221200	-2.33029100	1.31112400
H	-8.11700500	-1.58375700	0.68835500
H	-7.46871000	-2.29593800	2.18620400
H	-7.76801900	0.40799100	2.11642500
H	-6.20635700	-0.24021200	2.68705100
C	-1.34141800	-2.89783600	-1.66187400
O	-2.51788300	-2.62919700	-0.89554300
C	-2.75042500	-3.78859800	-0.10200900
C	-1.37138000	-4.19789400	0.38578500
C	-0.45949400	-3.80565000	-0.78967800
H	-1.63358600	-3.39921100	-2.60056000
H	-0.88256400	-1.92870800	-1.90170100
H	-3.44534800	-3.51132700	0.69953000
H	-3.21296000	-4.57263900	-0.72982700
H	-1.10613300	-3.62540200	1.28660900
H	-1.31248900	-5.26301600	0.63891600
H	0.43487500	-3.27993500	-0.42937100
H	-0.12093200	-4.68249200	-1.35771400
C	-3.60504700	2.83496500	-0.60801500
O	-3.91330000	1.57640800	-1.21673100
C	-4.70718600	1.77528600	-2.38670800
C	-4.68949100	3.27314300	-2.64560700
C	-4.57475800	3.82349500	-1.22780200
H	-3.70686100	2.72193500	0.48126700
H	-2.55583900	3.09493200	-0.82762200
H	-4.27956500	1.18094000	-3.20630000
H	-5.72717000	1.40470100	-2.19009800
H	-3.79920100	3.54936000	-3.23029800
H	-5.57597500	3.62113700	-3.18806700
H	-4.21516500	4.85759200	-1.18212400
H	-5.54972100	3.77478600	-0.71901500

P

N	-0.78958000	-0.85194500	1.99034100
H	-1.78320300	-0.87492900	2.21295000
B	-0.11638600	-2.24911600	1.67837500
H	0.80813500	-2.07170800	0.89371900
H	0.28098700	-2.81895300	2.67875500
H	-0.98625700	-2.92361800	1.11966000
Na	-0.77025900	-1.09631400	-0.59204600
C	-3.86363900	-0.12637200	-0.94463200
O	-3.11330300	-1.28623800	-0.59446300
C	-3.81338400	-1.87176800	0.50875200
C	-4.43825500	-0.70273000	1.29017000
C	-4.17943100	0.51847400	0.39260200
H	-3.24726300	0.47717500	-1.62056500
H	-4.78205900	-0.43489400	-1.47711600
H	-4.58826000	-2.54719500	0.11098600
H	-3.08850800	-2.47184800	1.07216700
H	-5.51073200	-0.87335300	1.44728400
H	-3.98610500	-0.56843000	2.28100300
H	-5.03078300	1.20791400	0.34640900
H	-3.30372300	1.07332000	0.76120700
C	2.38605100	-0.73721300	-1.39610400
O	1.26872700	-1.55404600	-1.78814900
C	1.63756000	-2.94133600	-1.71513700
C	2.95388500	-2.98346600	-0.96008700
C	3.58989200	-1.66101600	-1.37407900
H	2.48055900	0.08685500	-2.11690400
H	2.18599300	-0.31495600	-0.39776700

H	0.83233900	-3.48971400	-1.20659900
H	1.73939300	-3.32868900	-2.74170000
H	2.76350500	-2.99328700	0.12229500
H	3.55810800	-3.86098300	-1.21793400
H	4.36457100	-1.31207700	-0.68130000
H	4.03571900	-1.74222700	-2.37640800
C	-1.26120900	2.35049600	-1.00062600
O	-0.81757000	1.08288100	-1.50890100
C	-0.13102000	1.36163700	-2.72513900
C	0.70087600	2.58701200	-2.39686100
C	-0.25804500	3.39737300	-1.51448500
H	-2.27307100	2.55212100	-1.39334600
H	-1.31863500	2.26347100	0.09203300
H	0.43645400	0.46586500	-3.00398800
H	-0.86645400	1.57988500	-3.52218300
H	1.58926800	2.28614900	-1.82248000
H	1.03533500	3.13250300	-3.28706600
H	0.26807000	3.90448600	-0.69636400
H	-0.77226100	4.17259300	-2.09673800
C	3.42658100	0.17876000	2.24489000
C	2.08150900	-0.16089300	2.36708300
C	1.09102000	0.71555900	1.91324000
C	1.47214100	1.94401500	1.36766800
C	2.81484200	2.27430700	1.22147900
C	3.79788600	1.38730700	1.65818700
H	4.18995700	-0.50630900	2.61268300
H	1.79680500	-1.10862800	2.81974700
H	0.68830300	2.64148900	1.07619100
H	3.09535700	3.23162700	0.78147800
H	4.85170000	1.64530300	1.55730000
C	-0.37816400	0.43094700	2.03693600
O	-1.16985100	1.38529500	2.14372500

CH_3ONa • 3THF

O	1.95459300	0.91425700	-1.29504000
C	3.18468800	1.44420500	-1.46088800
H	3.29940200	2.07009900	-2.37630200
H	3.50924200	2.11361900	-0.61965600
H	4.00271100	0.68172100	-1.53509100
Na	0.14529100	-0.01407500	-0.90748400
C	-2.19332700	-1.49161100	0.46461500
O	-2.07752200	-0.67377400	-0.71176200
C	-3.36833700	-0.43275800	-1.27121000
C	-4.36141800	-0.87831900	-0.21442300
C	-3.60946700	-2.03009700	0.44059500
H	-1.41217400	-2.25732700	0.42496500
H	-2.01311500	-0.85914900	1.34681000
H	-3.44581700	0.62948200	-1.53069600
H	-3.47756800	-1.02106200	-2.19351300
H	-4.53249900	-0.07563100	0.51353000
H	-5.32672000	-1.16452500	-0.63798700
H	-3.97883900	-2.28461500	1.43646400
H	-3.66841800	-2.92755900	-0.18650300
C	0.76845400	2.18139800	1.21745700
O	-0.35412300	1.29204900	1.11531700
C	-1.47687200	2.13210900	0.91579400
C	-1.00296500	3.13347200	-0.12176900
C	0.46746800	3.34958000	0.27129600
H	0.85793000	2.51159200	2.26317500
H	1.65348600	1.62180400	0.90338900
H	-2.31791700	1.50631100	0.60076900
H	-1.74256800	2.63472800	1.86157300
H	-1.06889200	2.68554500	-1.12232400
H	-1.59315400	4.05369300	-0.13076800
H	1.12265700	3.29709600	-0.60160900
H	0.61251400	4.31195300	0.77103300
C	2.28647900	-2.20190200	-0.78553200
O	1.10664200	-1.97536700	0.01576300
C	1.49782200	-1.62223600	1.34691900
C	2.96358500	-1.24962800	1.25516500
C	3.44853300	-2.22291200	0.18914400
H	2.15172200	-3.14007800	-1.33359900
H	2.38648800	-1.35572100	-1.48164600
H	0.85440100	-0.80433900	1.69493400
H	1.34604500	-2.48968700	2.00873600
H	3.06265800	-0.22272800	0.88374700
H	3.48481100	-1.34490300	2.21119500
H	4.38488100	-1.91627300	-0.28272500
H	3.58596500	-3.22433800	0.61721500

3. Supplementary Figures

The amidation of methyl benzoate with NaAB was used as a model reaction. The reaction of different ratio of NaAB to methyl benzoate in THF-d_8 was monitored by $^1H\{^{11}B\}$ NMR spectra at room temperature. The results indicated that the methyl benzoate could not be consumed completely at a 1:1 ratio (a) but the methyl benzoate nearly consumed at the 2.4:1 ratio (b).

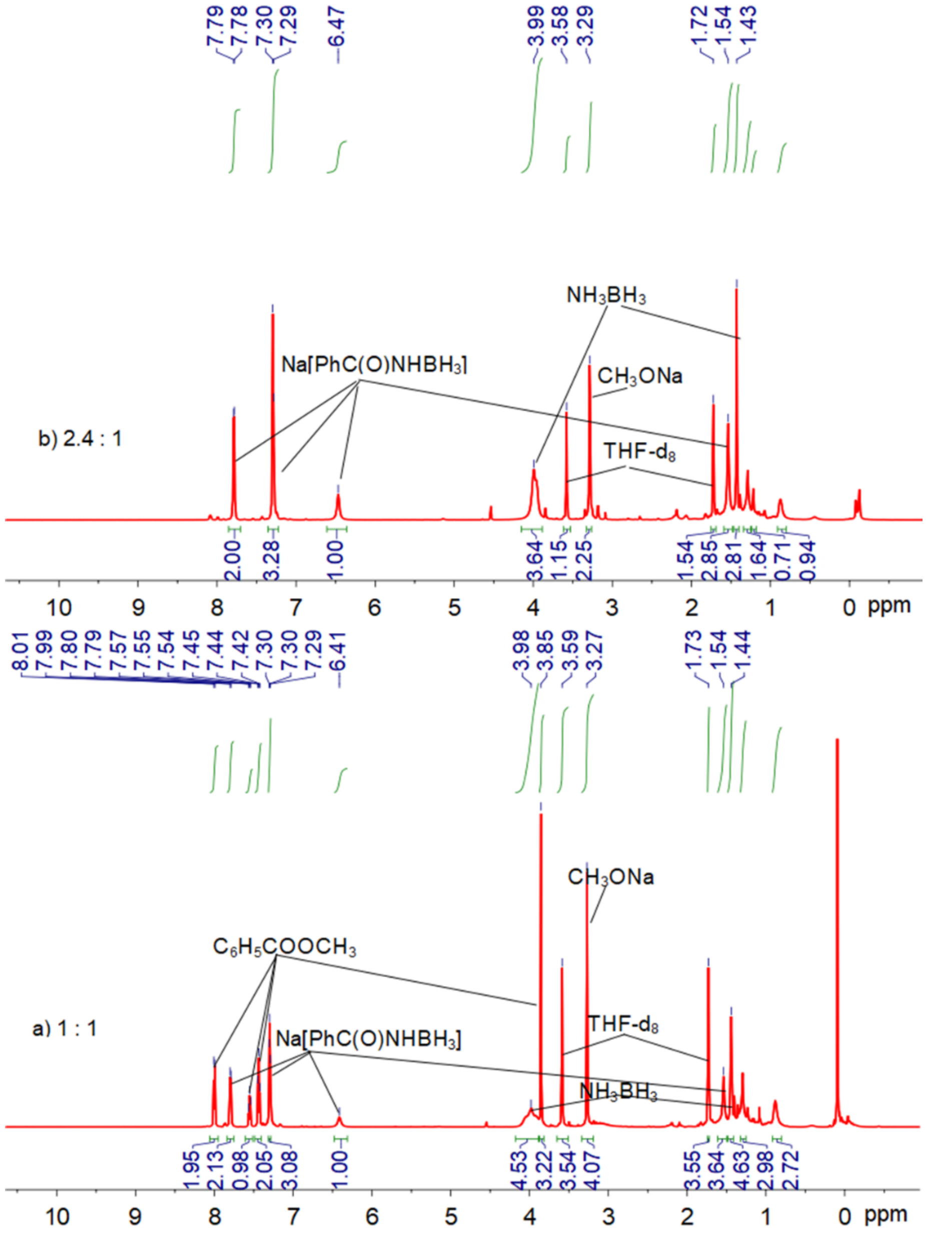

Supplementary Figure 1. $^1H\{^{11}B\}$ NMR spectra of the reaction of NaAB with methyl benzoate in THF-d_8 at different ratios. a) 1:1, b) 2.4:1.

Characterization of the $Na[PhC(O)NHBH_3]$ intermediate (Supplementary Figures 2-6)

In order to characterize the product of the reaction of methyl benzoate and NaAB in THF, the forming complex is isolated before hydrolysis. After reaction completion, removal of solvent afforded a white powder from which NH_3BH_3 was extracted by a mixed solvent of CH_2Cl_2 and n-hexane (16:1). After removing NH_3BH_3, relatively pure $Na[PhC(O)NHBH_3]$ (white solid) can be obtained, which was dissolved in CH_3CN to run the ESI-MS (Supplementary Figure 2). A peak of 120.0450 has the maximum abundance in the electrospray ionization mass spectra (ESI-MS) is related to the formula weight of $Na[PhC(O)NHBH_3]$ (except for the BH_3 free ligand fragment generated due to electrospray ionization, Supplementary Figure 2)[69].

^{11}B, $^{11}B\{^1H\}$, ^1H, $^1H\{^{11}B\}$, and ^{13}C NMR (Supplementary Figures 3-6). A quartet signal at δ -25.05 ppm ($J_{B\text{-}H}$ = 87.30 Hz) in ^{11}B NMR (CD_3CN) (top in Supplementary Figure 3), corresponding to a singlet signal at the same chemical shift in $^{11}B\{^1H\}$ NMR (bottom in Supplementary Figure 3) with hydrogen decoupling, is assigned to the B atom of the BH_3 group in $Na[PhC(O)NHBH_3]$. A multiplet at δ 7.37 and 7.73 ppm in $^1H\{^{11}B\}$ NMR (Supplementary Figure 4) corresponds to the H atoms of phenyl group, and a broad signal at δ 6.27 ppm is ascribed to the H atom in the NH group. A singlet at δ 1.44 ppm, corresponding to a quartet signal in ^1H NMR spectra with the coupling constant of 85.20 Hz (Supplementary Figure 5), is attributed to the H atoms in the BH_3 group. The integral ratios of the all H atoms in each functional group are consistent with the theoretical value on the basis of the formula of $Na[PhC(O)NHBH_3]$.

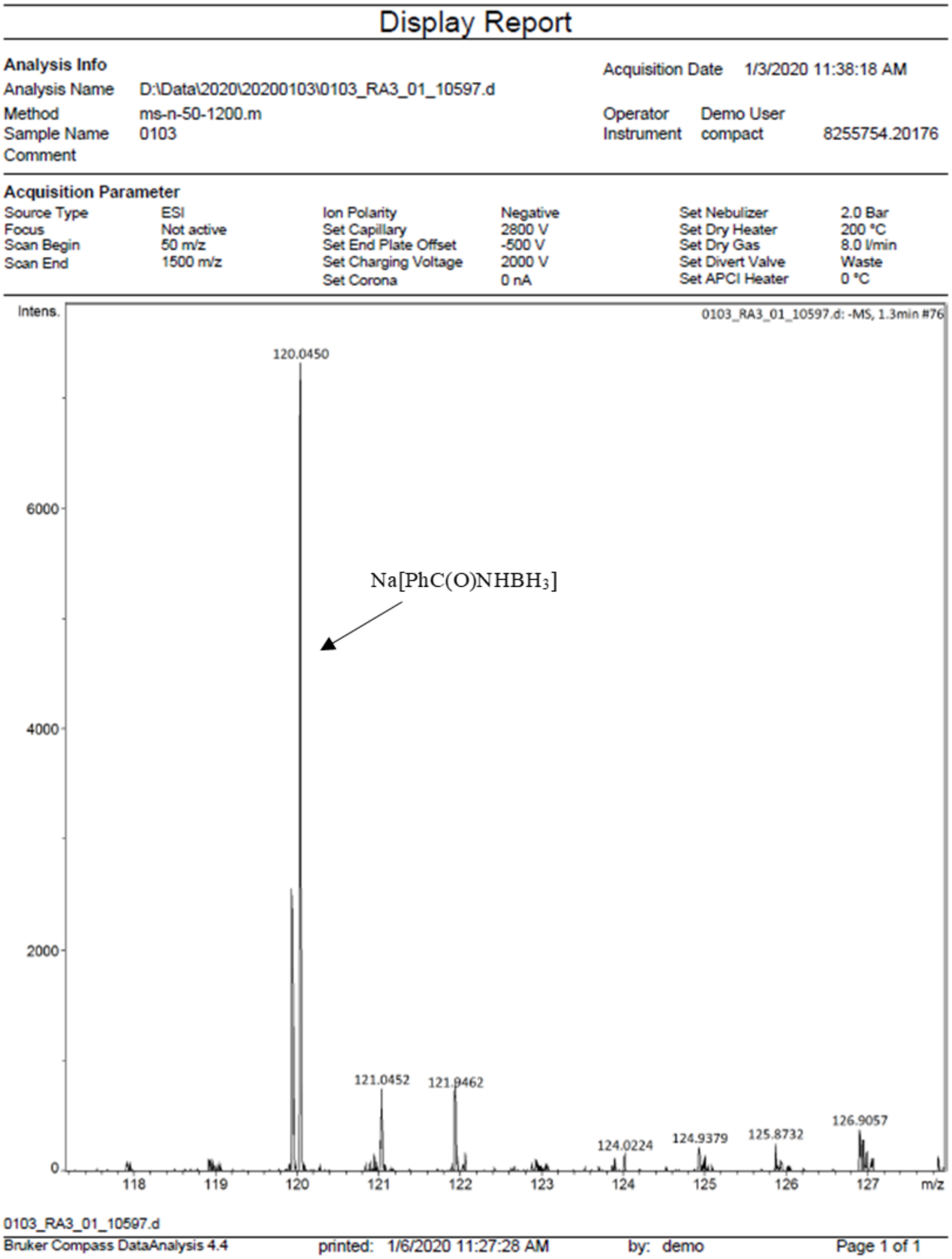

Supplementary Figure 2. ESI-MS spectrum of Na[PhC(O)NHBH_3] in anhydrous CH_3CN. m/z calculated for C_7H_6NO [M] 120.0444, found 120.0450.

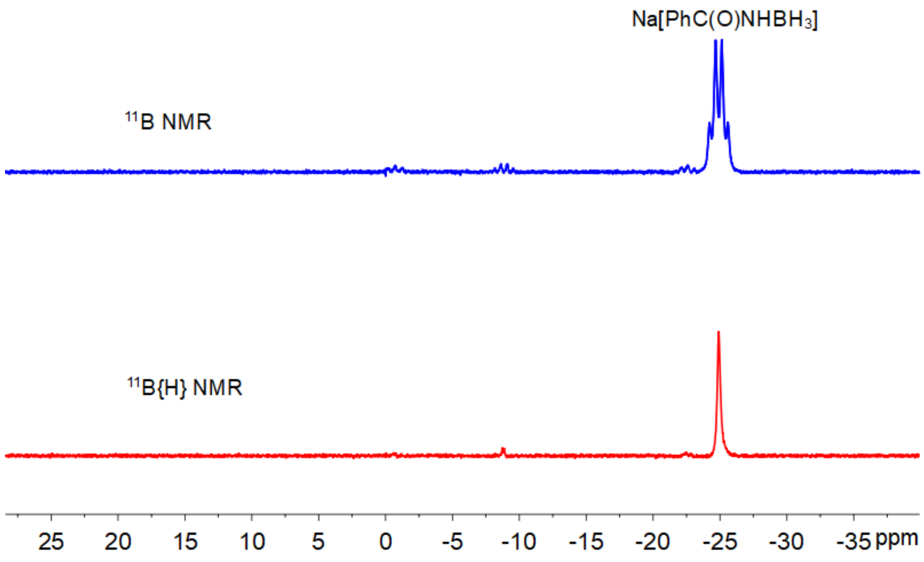

Supplementary Figure 3. ^{11}B and ^{11}B{^{1}H} NMR spectra of the isolated Na[PhC(O)NHBH_3] in CD_3CN.
Na[PhC(O)NHBH_3], ^{11}B NMR (193 MHz) δ -25.05 ppm (q, J_{B-H} = 87.30 Hz).

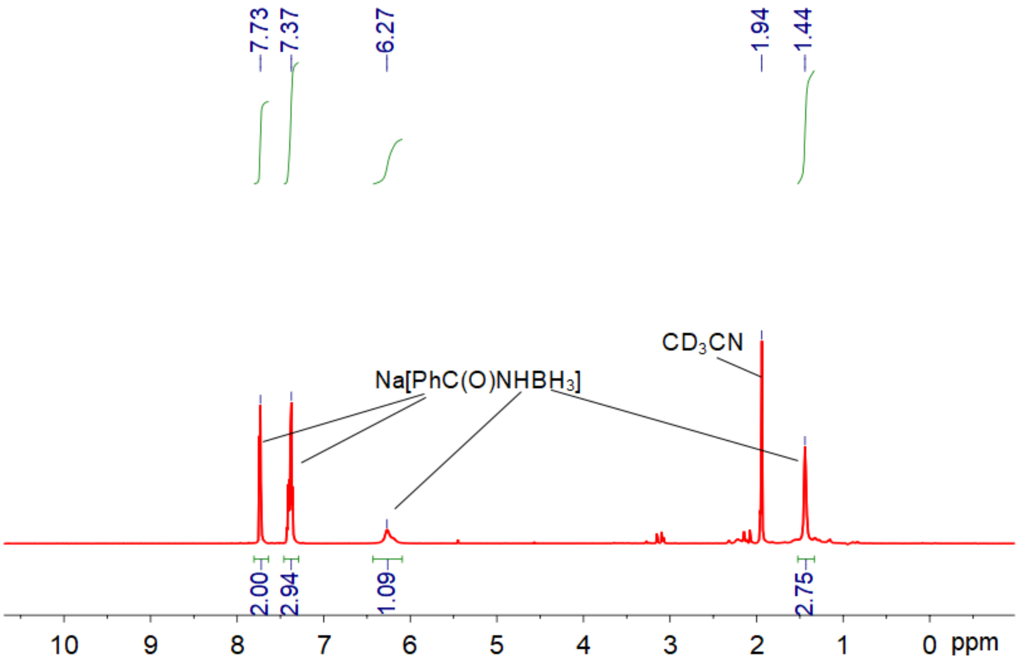

Supplementary Figure 4. ^{1}H{^{11}B} NMR spectrum of the isolated Na[PhC(O)NHBH_3] in CD_3CN. ^{1}H{^{11}B} NMR (600 MHz, CD_3CN) δ 7.73 (d, *J* = 8.2 Hz, 2H), 7.46-7.29 (m, 3H), 6.27 (s, 1H), 1.44 (s, BH, 3H).

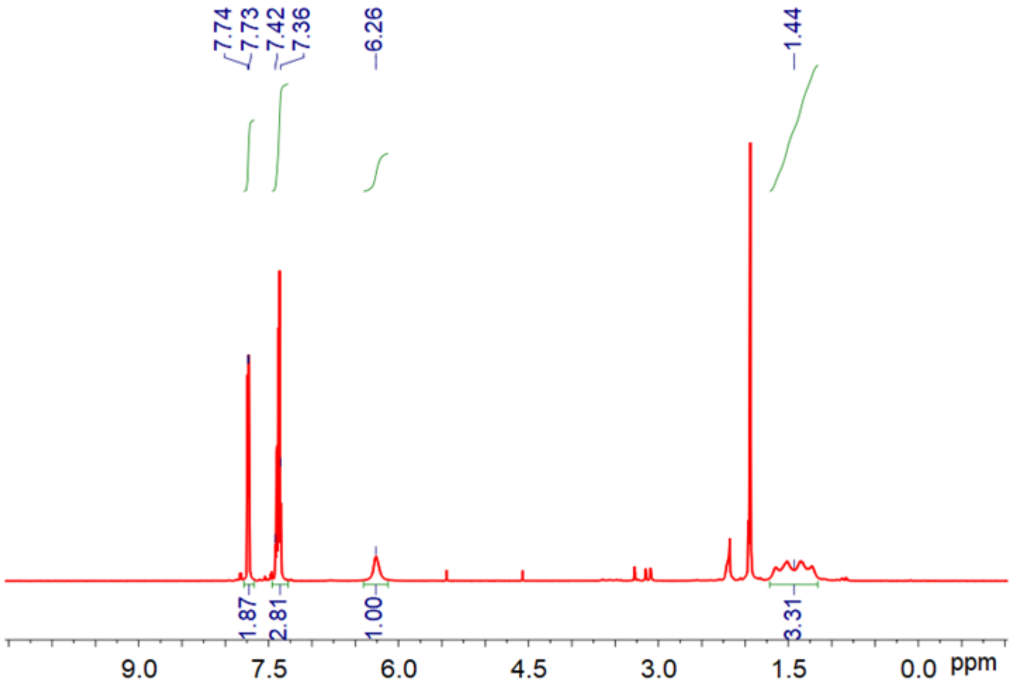

Supplementary Figure 5. ^{1}H NMR spectrum of the isolated $Na[PhC(O)NHBH_3]$ in CD_3CN. ^{1}H NMR (600 MHz, CD_3CN) δ 7.74 (d, *J* = 8.2 Hz, 2H), 7.46-7.28 (m, 3H), 6.26 (s, 1H), 1.44 (q, BH, 3H).

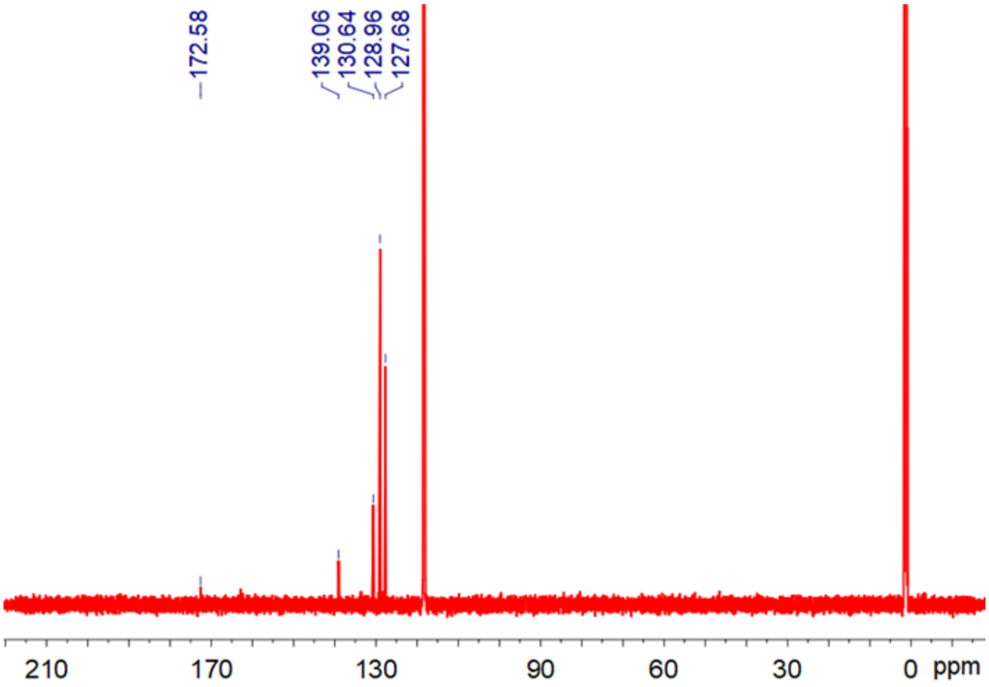

Supplementary Figure 6. ^{13}C NMR spectrum of the isolated $Na[PhC(O)NHBH_3]$ in CD_3CN. ^{13}C NMR (151 MHz, CD_3CN) δ 172.6, 139.1, 130.6, 129.0, 127.7.

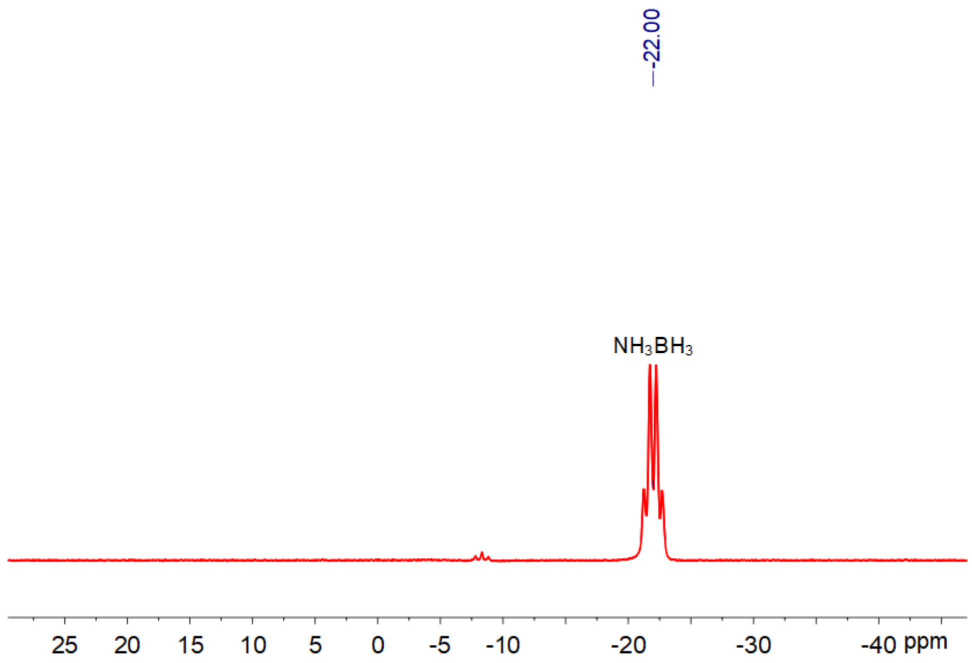

Supplementary Figure 7. ^{11}B NMR of the reaction mixture of methyl 4-hydroxyl-benzoate and NaAB in THF.

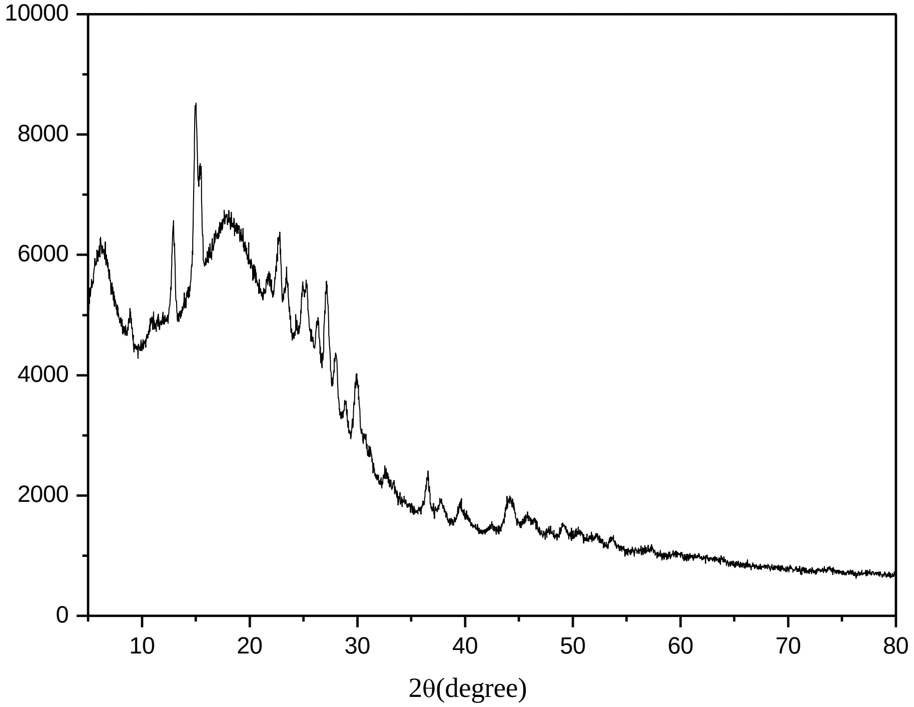

Supplementary Figure 8. XRD pattern of the solid product in the reaction of methyl 4-hydroxyl-benzoate and NaAB.

In order to study the mechanism, the reaction of methyl benzoate with NaAB was carried out in THF-d_8 at room temperature and the reaction mixture was recorded in situ by NMR spectroscopy after 5 min (Supplementary Figures 9-11).

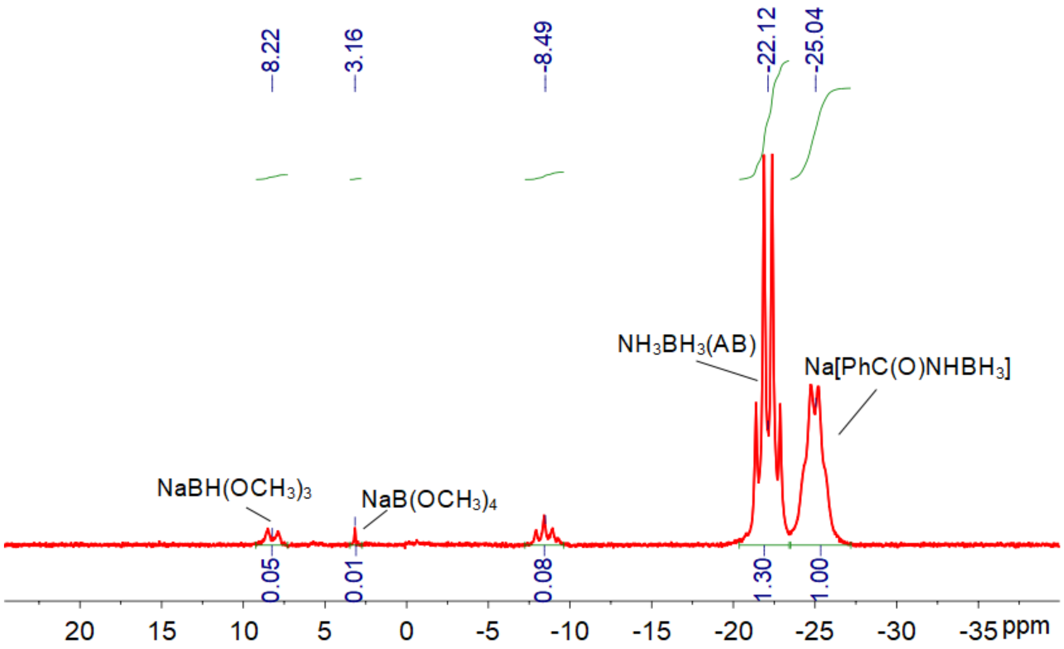

Supplementary Figure 9a. ^{11}B NMR spectrum of the reaction of methyl benzoate with NaAB (2.4 equiv) in THF-d_8 at room temperature after 5 min.

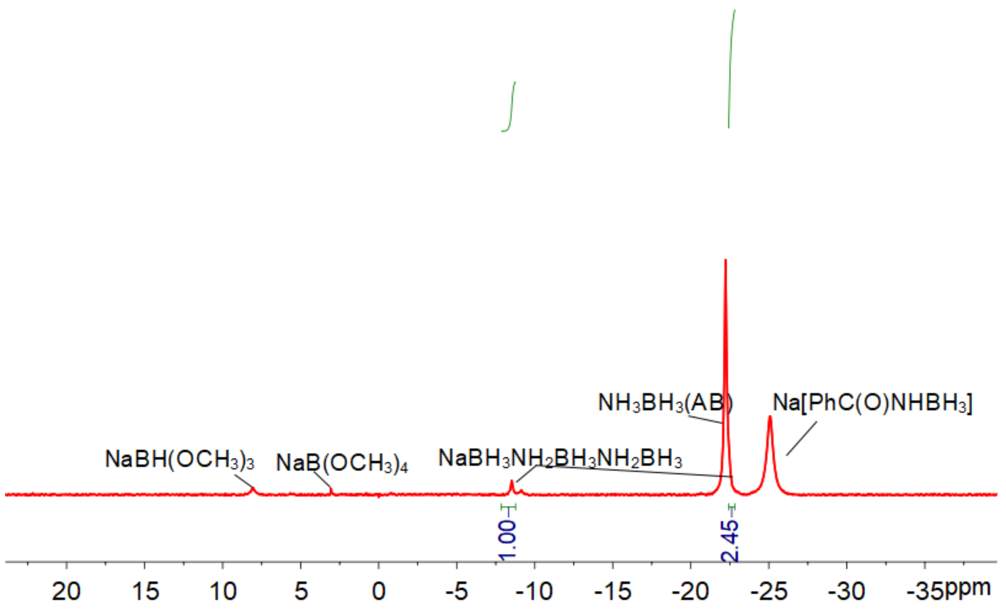

Supplementary Figure 9b. $^{11}B\{^{1}H\}$ NMR spectrum of the reaction of methyl benzoate with NaAB (2.4 equiv) in THF-d_8 at room temperature after 5 min.

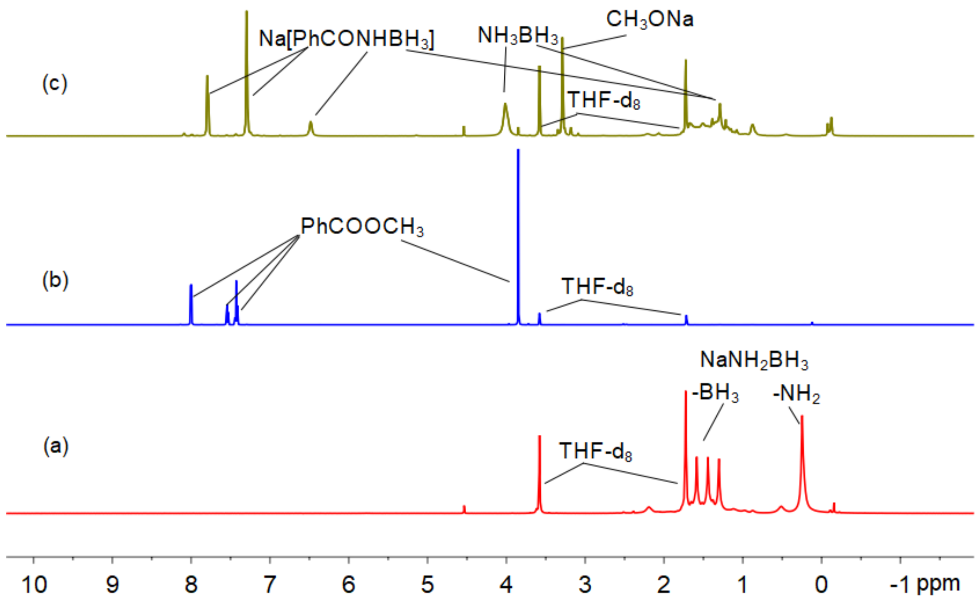

Supplementary Figure 10. ^{1}H NMR spectra of the reaction mixture (a) in comparison with the starting materials of NaAB (b) and methyl benzoate (c) The reaction mixture was taken from the reaction of methyl benzoate with NaAB (2.4 equiv) in THF-d_8 at room temperature after 5 min.

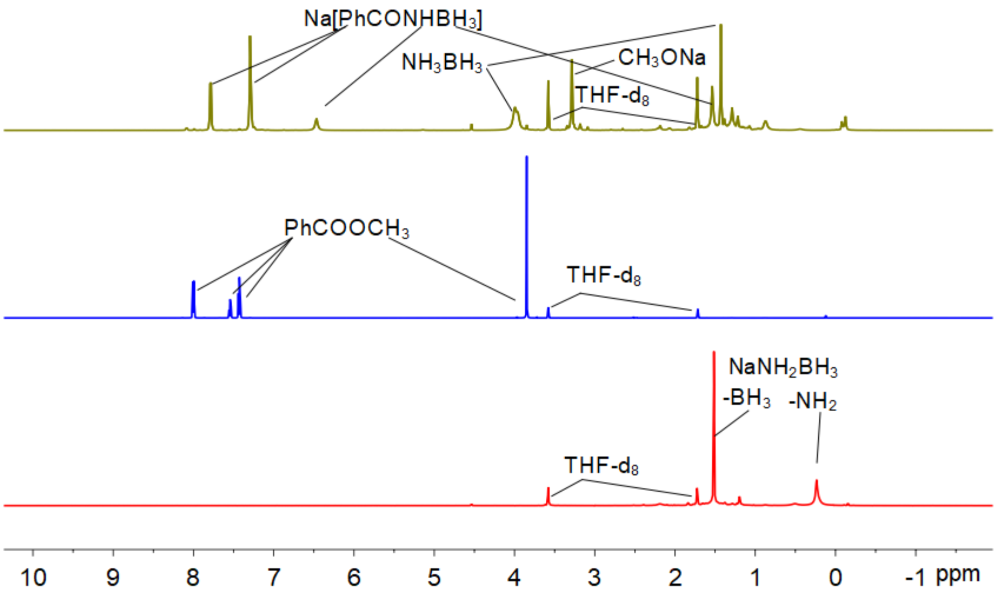

Supplementary Figure 11. $^{1}H\{^{11}B\}$ NMR spectra of the reaction mixture (a) in comparison with the starting materials of NaAB (b) and methyl benzoate (c) The reaction mixture was taken from the reaction of methyl benzoate with NaAB (2.4 equiv) in THF-d_8 at room temperature after 5 min.

Characterization of the Na[PhC(O)NMeBH$_3$] intermediate (Supplementary Figures 12-15)

In order to characterize the product of the reaction of methyl benzoate and NaMeAB in THF, the forming complex is isolated before hydrolysis. After reaction completion, removal of solvent affords a white powder from which $MeNH_2BH_3$ was extracted by a mixture of CH_2Cl_2 and n-hexane (10:1). By removing $MeNH_2BH_3$ from the reaction system, relatively pure Na[PhC(O)NMeBH$_3$] (white solid) can be obtained, which was dissolved in THF-d$_8$ to measure ^{11}B, ^{11}B{^{1}H}, ^{1}H, ^{1}H{^{11}B}, and ^{13}C NMR (Supplementary Figures 12-15).

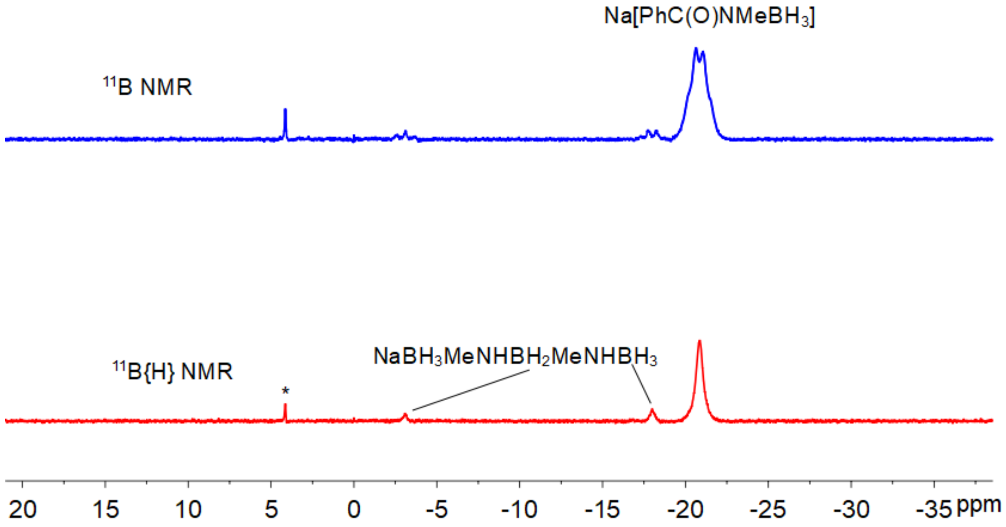

Supplementary Figure 12. ^{11}B and ^{11}B{^{1}H} NMR spectra of the isolated Na[PhC(O)NMeBH$_3$] in THF-d$_8$.

Na[PhC(O)NMeBH$_3$], ^{11}B NMR (193 MHz) δ -20.84 ppm (q, $J_{B\text{-}H}$ = 91.35 Hz). (*: Unknown species)

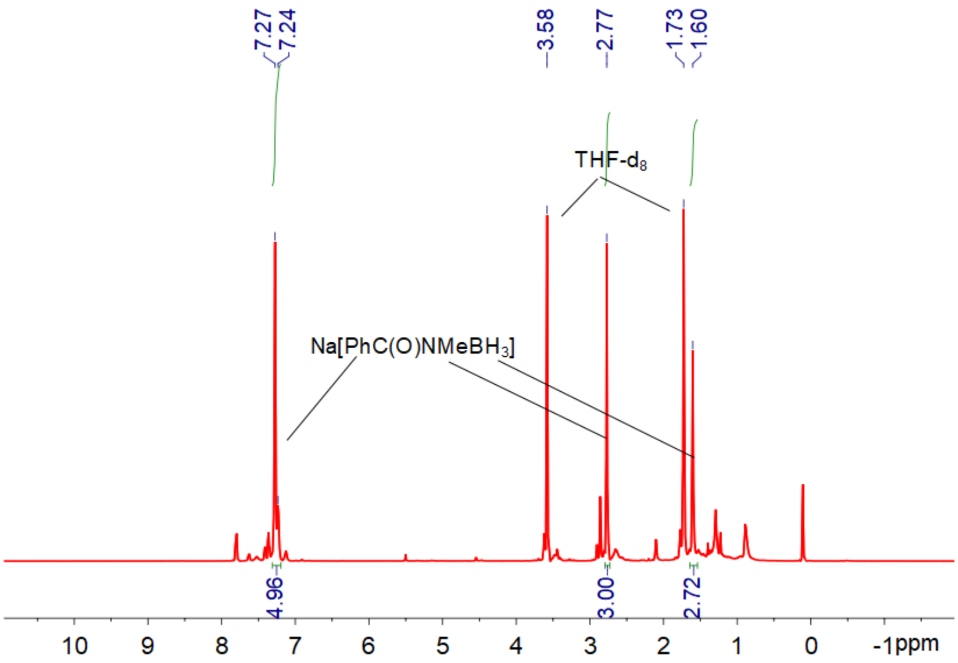

Supplementary Figure 13. ^{1}H{^{11}B} NMR spectrum of the isolated Na[PhC(O)NMeBH$_3$] in THF-d$_8$.

^{1}H{^{11}B} NMR (600 MHz, THF-d$_8$) δ 7.30-7.24 (m, 5H), 2.77 (s, 3H), 1.60 (s, BH, 3H).

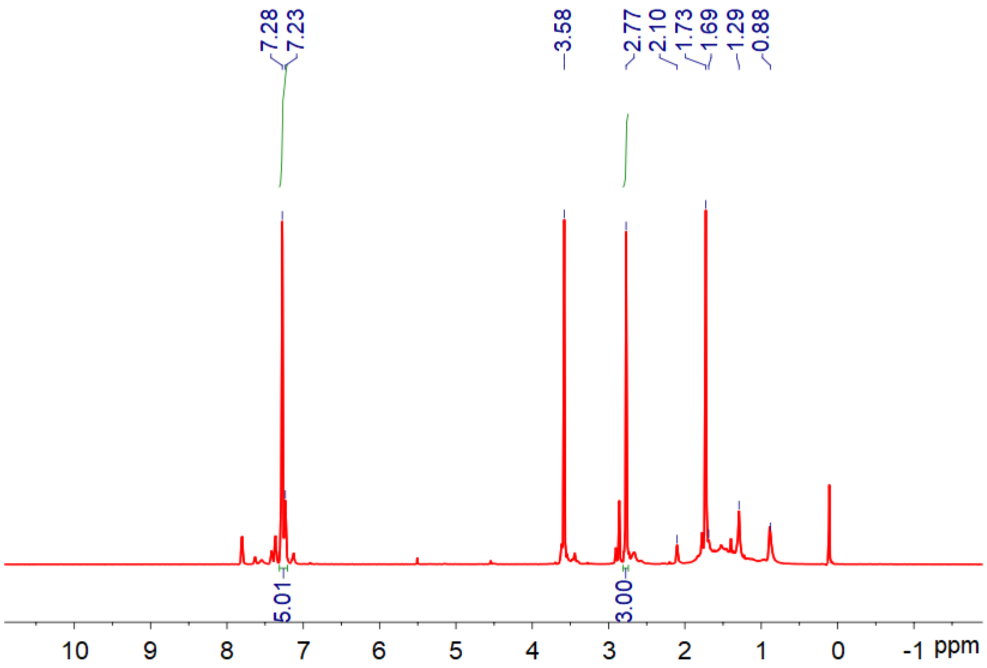

Supplementary Figure 14. ^{1}H NMR spectrum of the isolated $Na[PhC(O)NMeBH_3]$ in THF-d_8. ^{1}H NMR (600 MHz, THF-d_8) δ 7.30-7.24 (m, 5H), 2.77 (s, 3H), 1.60 (q, BH, 3H).

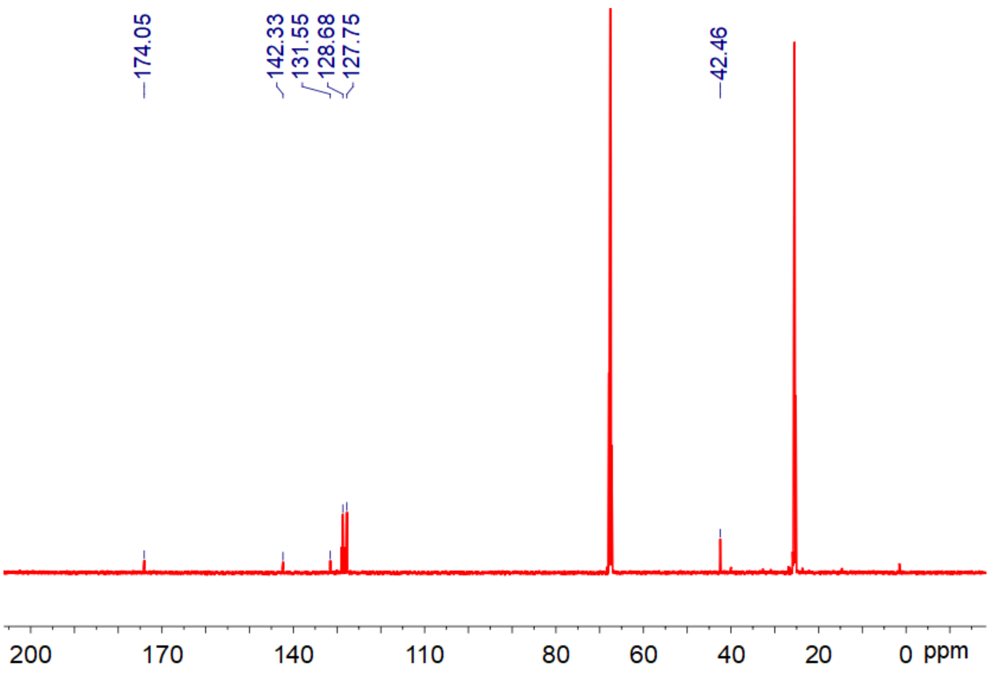

Supplementary Figure 15. ^{13}C NMR spectrum of the isolated Na[PhC(O)NMeBH$_3$] in THF-d$_8$. ^{13}C NMR (151 MHz, THF-d$_8$) δ 174.0, 142.3, 131.5, 128.7, 127.7, 42.5.

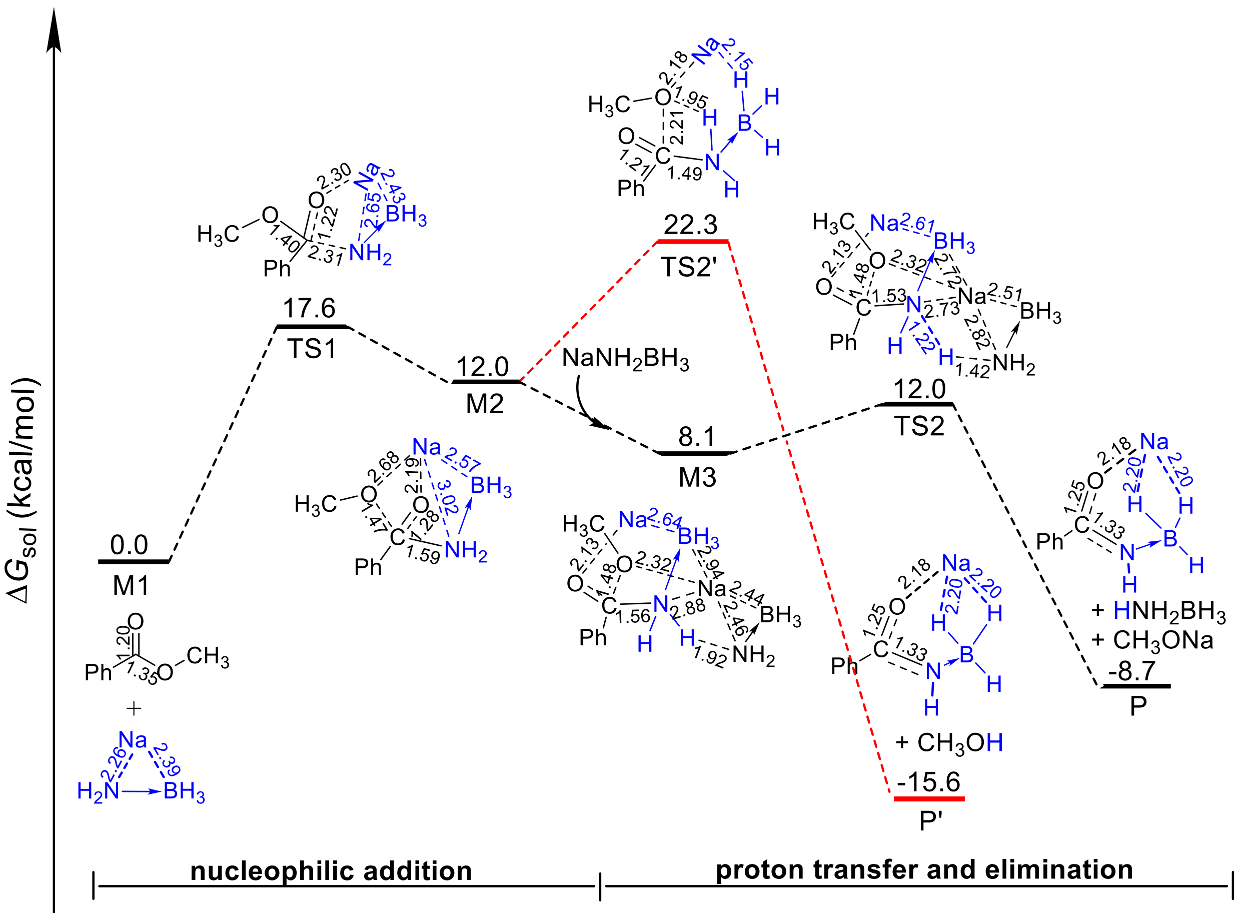

Supplementary Figure 16. Computed Gibbs free energy profile for the reaction of methyl benzoate with NaAB.

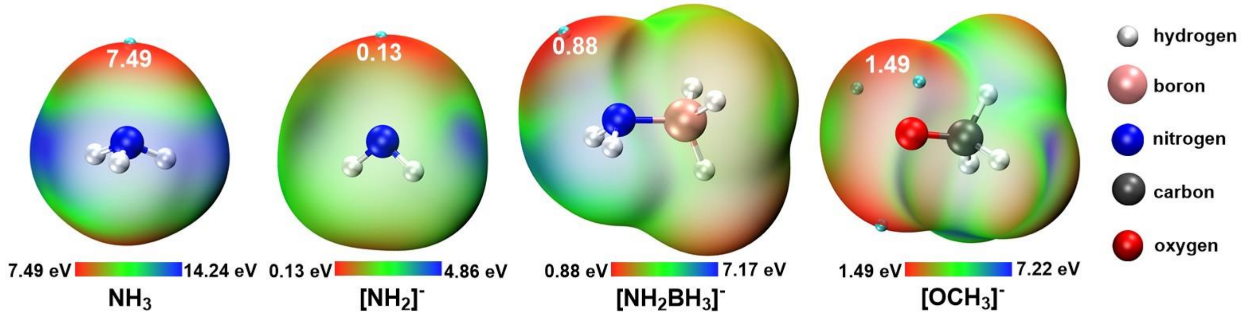

Supplementary Figure 17. The isosurfaces of calculated average local ionization energy (ALIE) of NH_3, $[NH_2]^-$, $[NH_2BH_3]^-$, and $[OCH_3]^-$ (ρ = 0.005 a.u.). Cyan spheres indicate the position of minima of ALIE on this surface.

The average local ionization energy (ALIE, $\bar{I}(\mathbf{r})$) is rigorously defined within the framework of self-consistent-filed molecular orbital (SCF-MO) theory, as given by eq. (1):

$$\bar{I}(\mathbf{r}) = -\sum_{i=1}^{HOMO} \frac{\varepsilon_i \rho_i(\mathbf{r})}{\rho(\mathbf{r})} \qquad (1)$$

$\rho_i(\mathbf{r})$ is the electronic density of ith molecular orbital at the point $\mathbf{r}$, ε_i is the eigenvalue of the orbital energy, and $\rho(\mathbf{r})$ is the total electron density. The smaller the ALIE minima is, the more nucleophilicity the specie is. The ALIE analysis is performed by using Multiwfn software package[70].

By calculating the ALIE, the $[NH_2]^-$ anion is the most reactive because of its least ALIE minimum (0.13 eV). The reactivity of $[NH_2BH_3]^-$ anion is the second corresponding to its minimum of 0.88 eV. The ALIE minimum of NH_3 is 7.49 eV. Theoretically, while the $NaNH_2$ molecule may react very fast, the low solubility of $NaNH_2$ solid in THF solvent makes this reaction hard to occur. The $[NH_2BH_3]^-$ anion is more favorable to combine with a cation compared with the $[OCH_3]^-$ anion. As shown in Supplementary Figure 17, the $[OCH_3]^-$ anion has three minima near the O atom (1.49 eV). Therefore, from M2 (In the text of Figure 4), the H(N) is more favorable to react with another $NaNH_2BH_3$ to form NH_3BH_3 rather than with the $[OCH_3]^-$ anion to form CH_3OH.

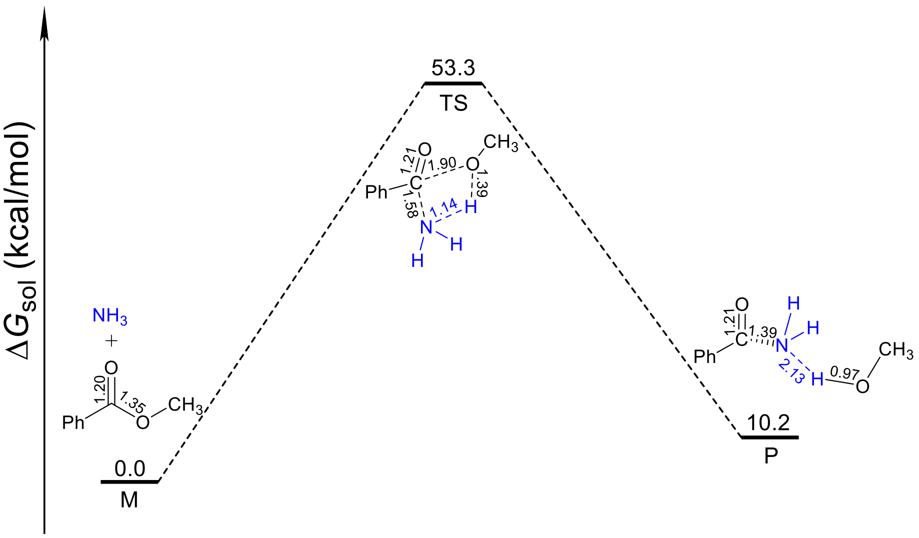

Supplementary Figure 18a. Energy profile for the reaction of methyl benzoate with NH_3 calculated.

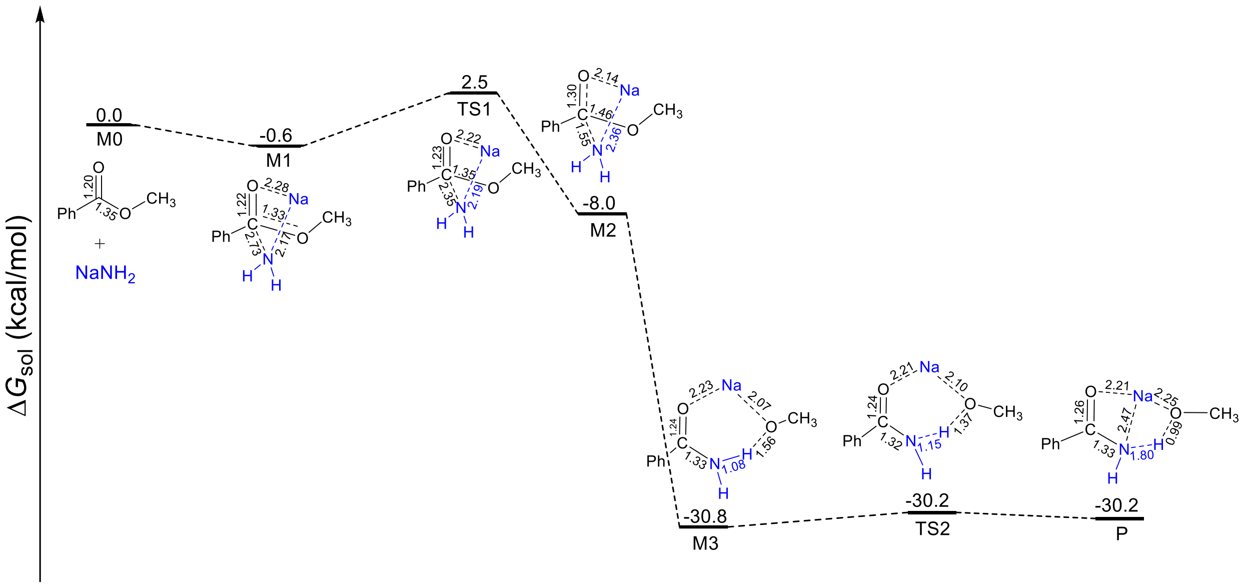

Supplementary Figure 18b. Energy profile for the reaction of methyl benzoate with $NaNH_2$ calculated.

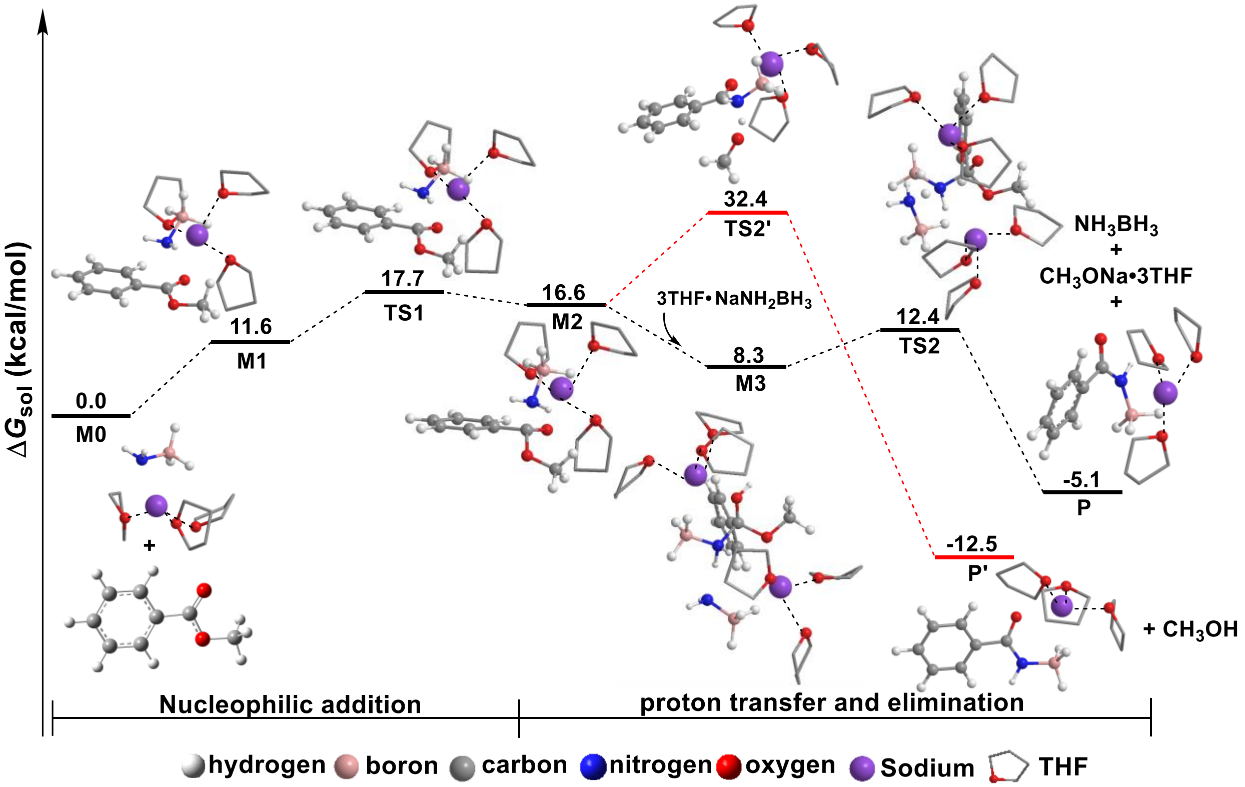

Supplementary Figure 18c. Computed Gibbs free energy profile for the reaction of methyl benzoate with 3THF·NaAB.

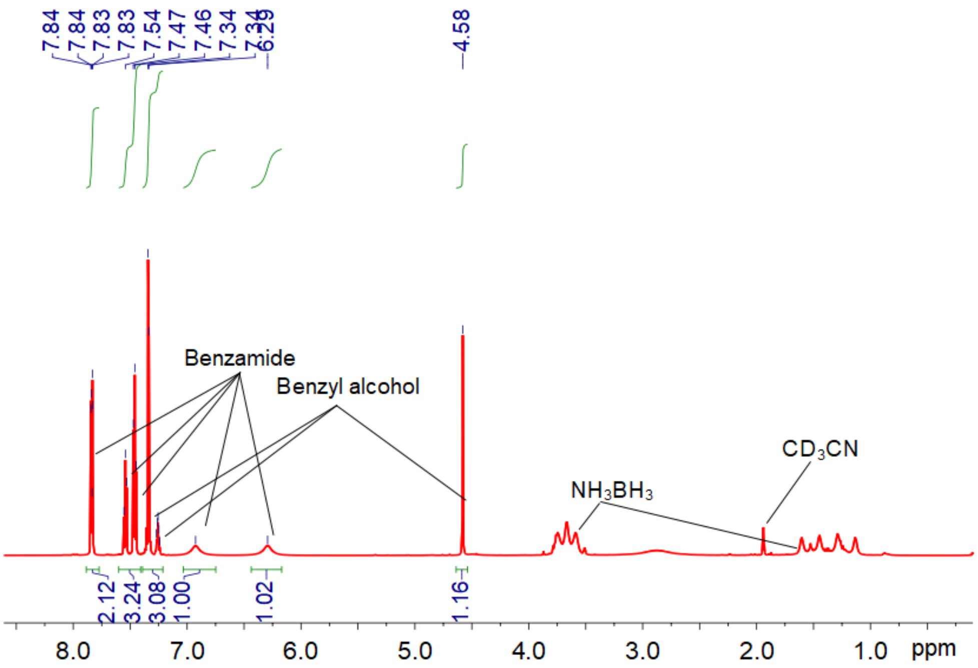

Supplementary Figure 19. In the reaction of $LiNH_2BH_3$ with methyl benzoate, the ester was converted into benzamide and benzyl alcohol.

Benzamide : ^{1}H NMR (600 MHz, CD_3CN) δ 7.84 (d, *J* = 7.2 Hz, 2H), 7.54 (t, *J* = 7.4 Hz, 1H), 7.46 (t, *J* = 7.7 Hz, 2H), 6.93 (s, 1H), 6.29 (s, 1H). Benzyl alcohol : ^{1}H NMR (600 MHz, CD_3CN) δ 7.34 (d, *J* = 4.5 Hz, 4H), 7.26 (dt, *J* = 8.7, 4.5 Hz, 1H), 4.58 (s, 2H).

NMR Spectra and Chiral HPLC Assays of Compounds:

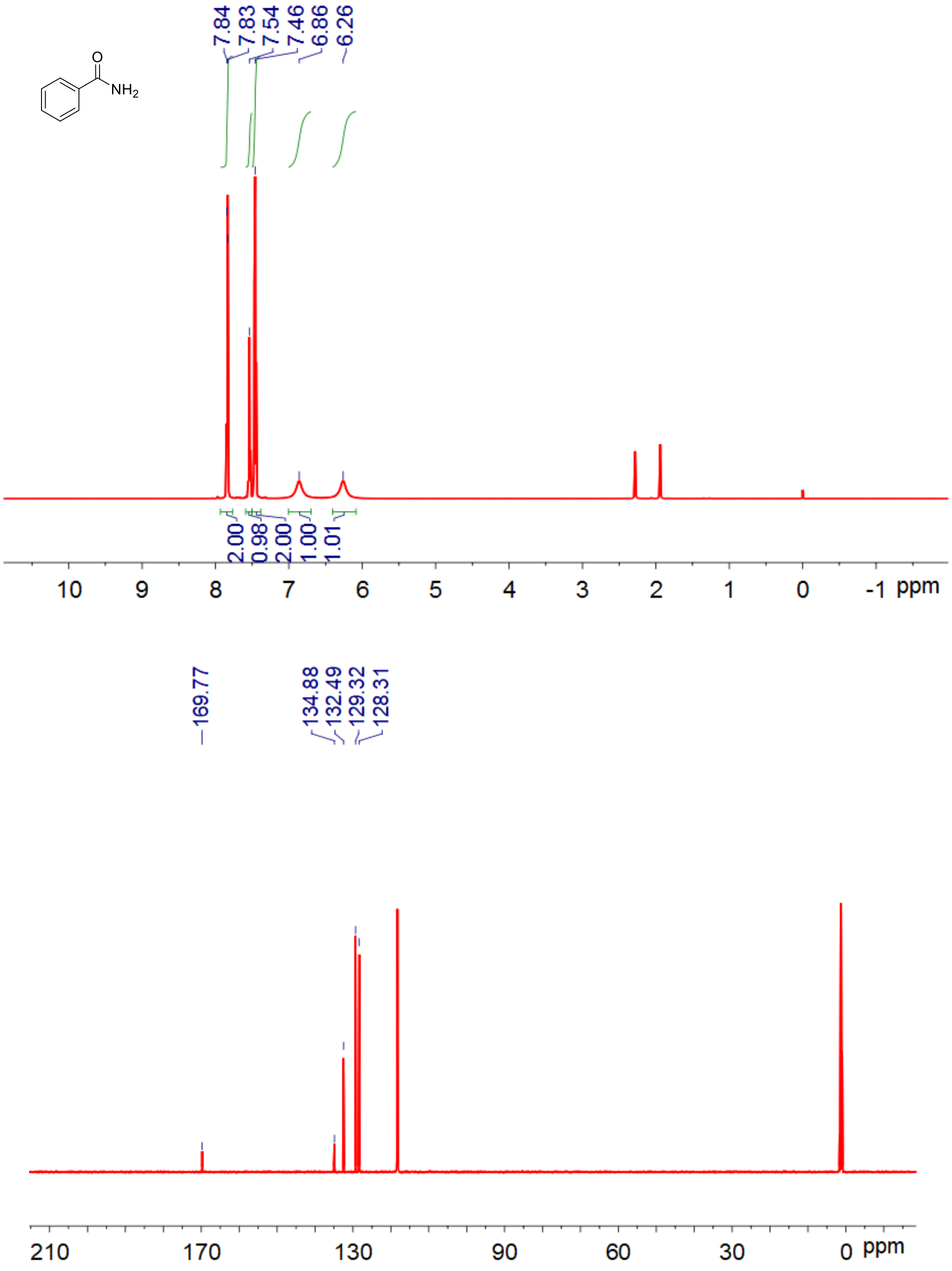

Supplementary Figure 20. ^{1}H NMR and ^{13}C NMR spectrum of benzamide (**1**).

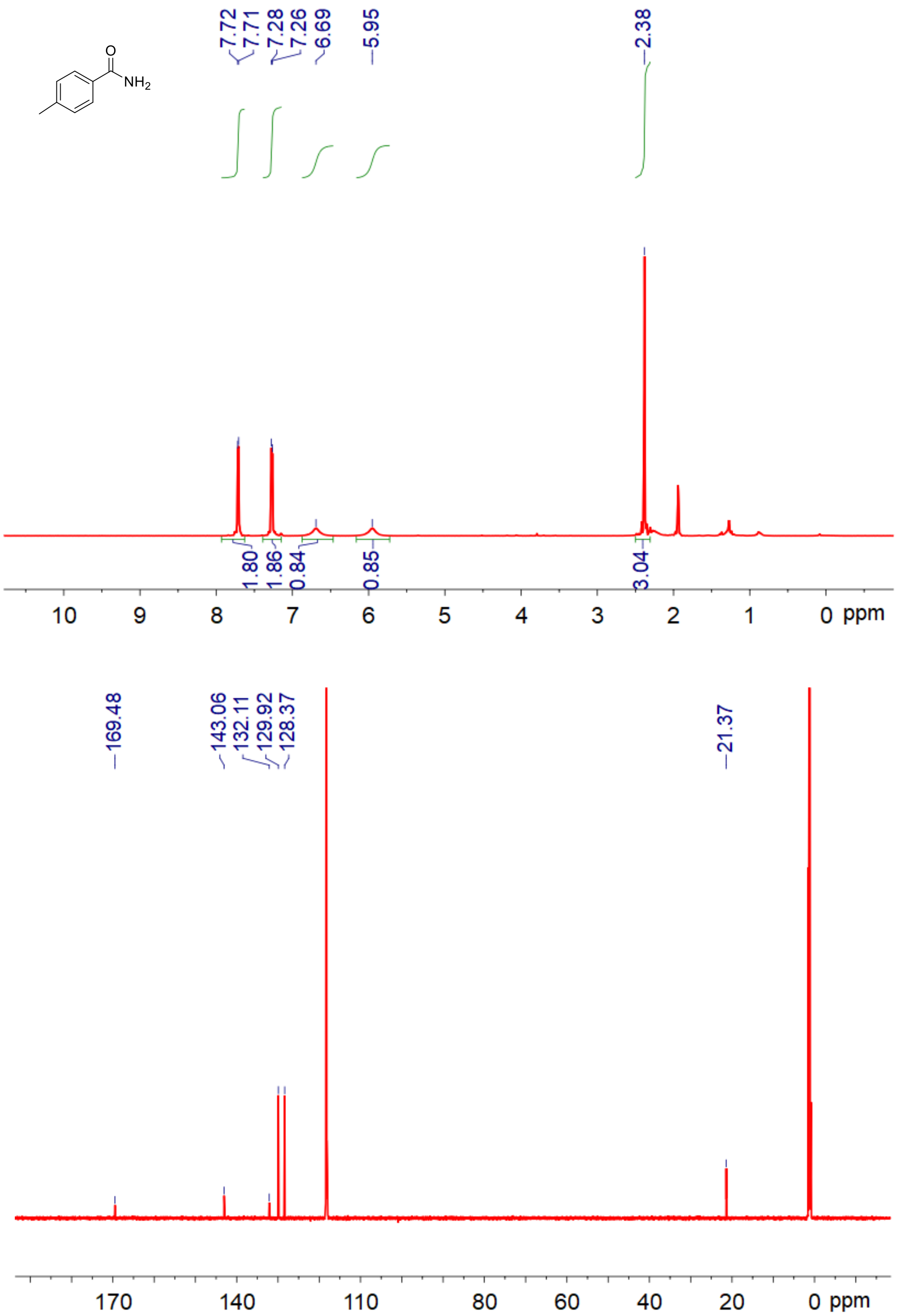

Supplementary Figure 21. ^{1}H NMR and ^{13}C NMR spectrum of 4-methylbenzamide (**2**).

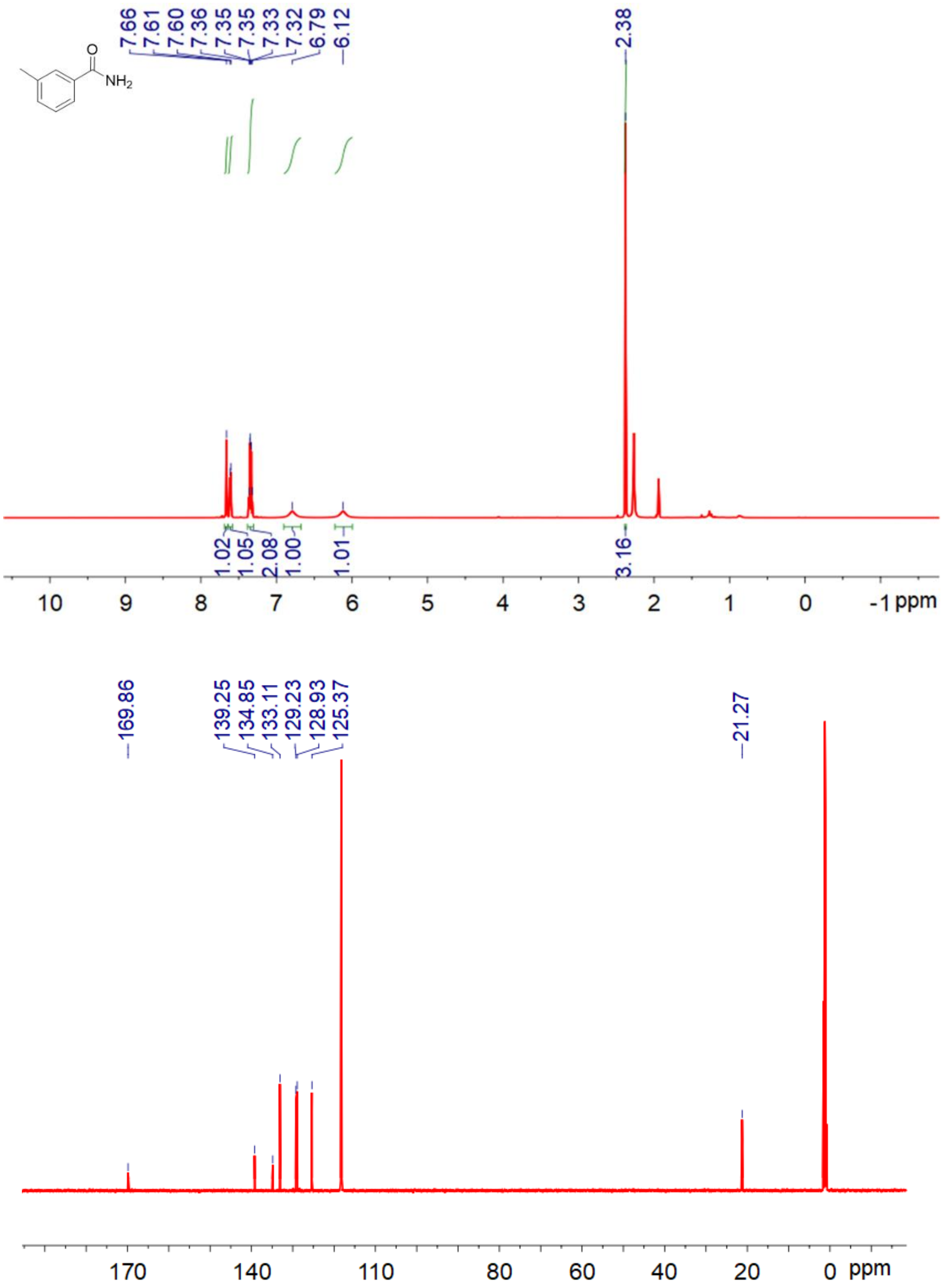

Supplementary Figure 22. ^{1}H NMR and ^{13}C NMR spectrum of 3-methylbenzamide (**3**).

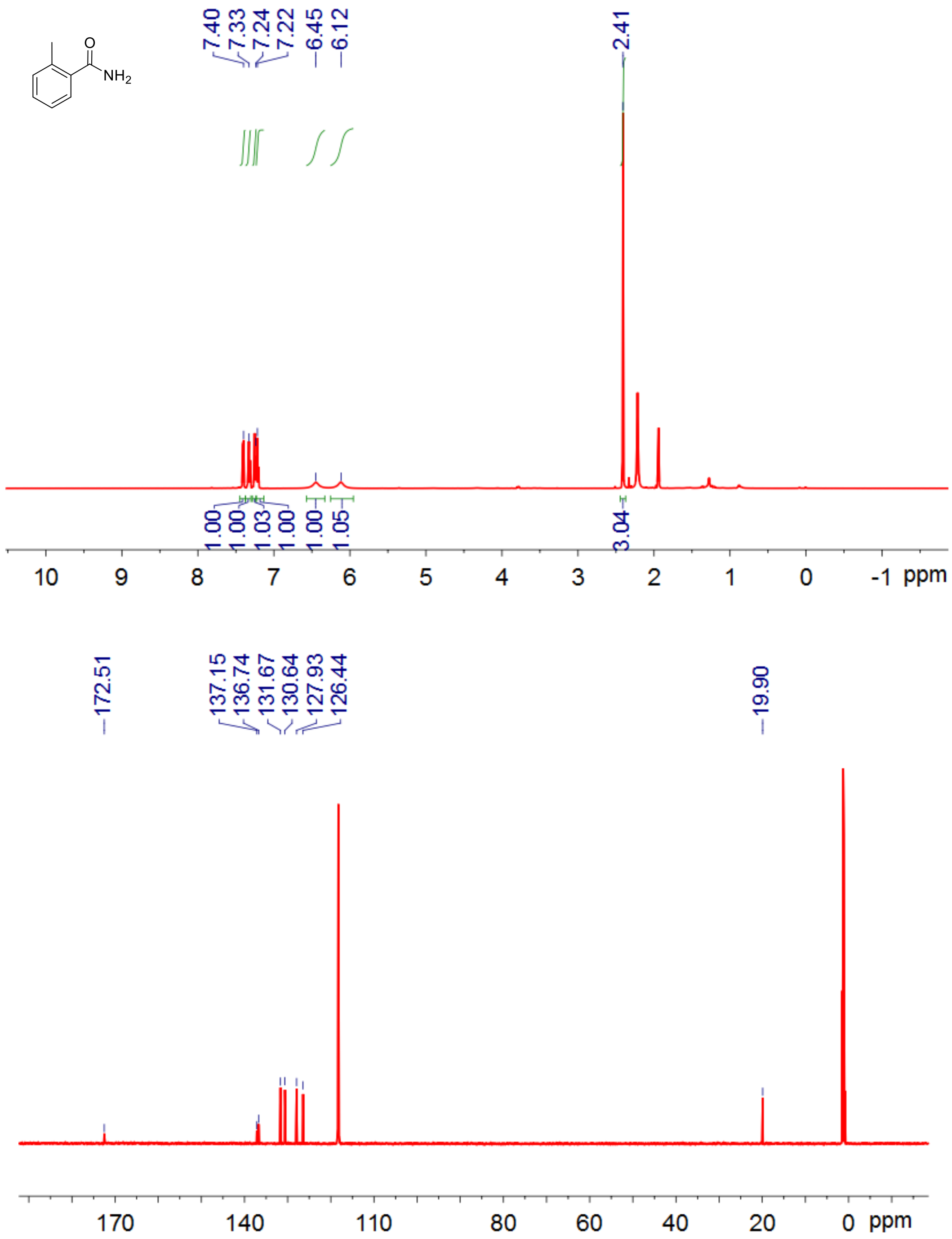

Supplementary Figure 23. ^{1}H NMR and ^{13}C NMR spectrum of 2-methylbenzamide (**4**).

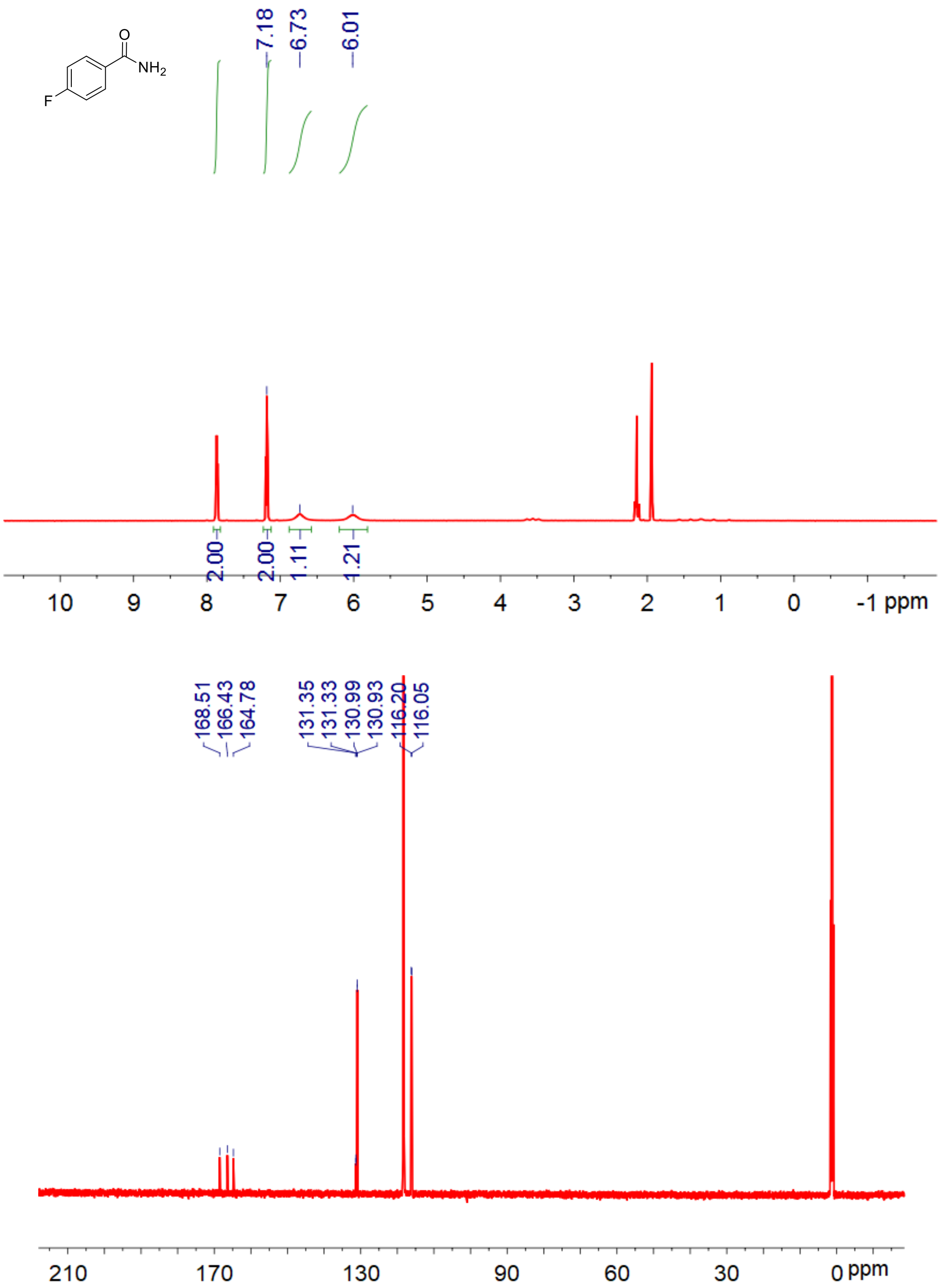

Supplementary Figure 24. ^{1}H NMR and ^{13}C NMR spectrum of 4-fluorobenzamide (**5**).

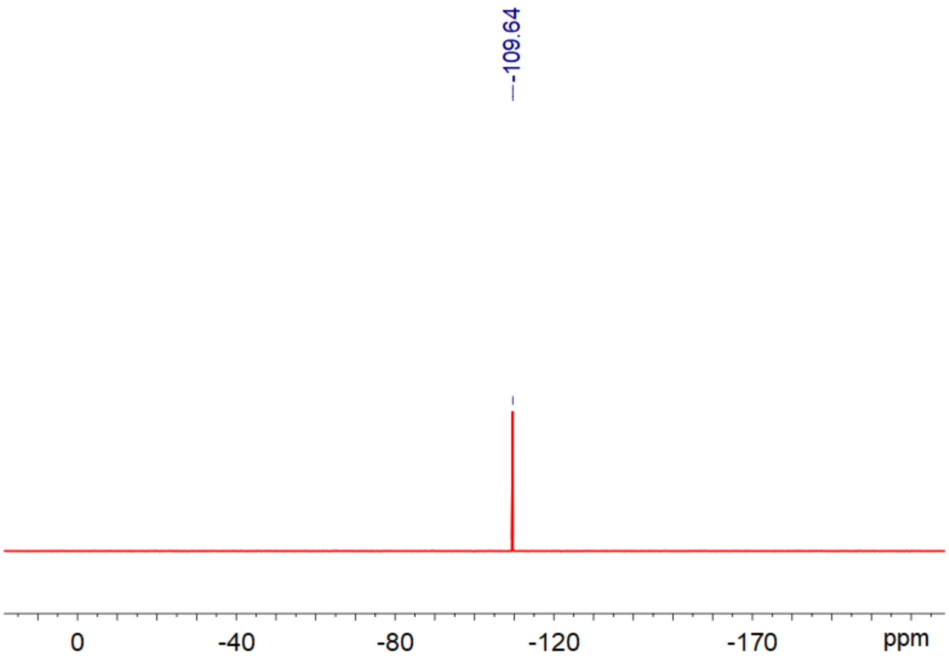

Supplementary Figure 25. ^{19}F NMR spectrum of 4-fluorobenzamide (**5**).

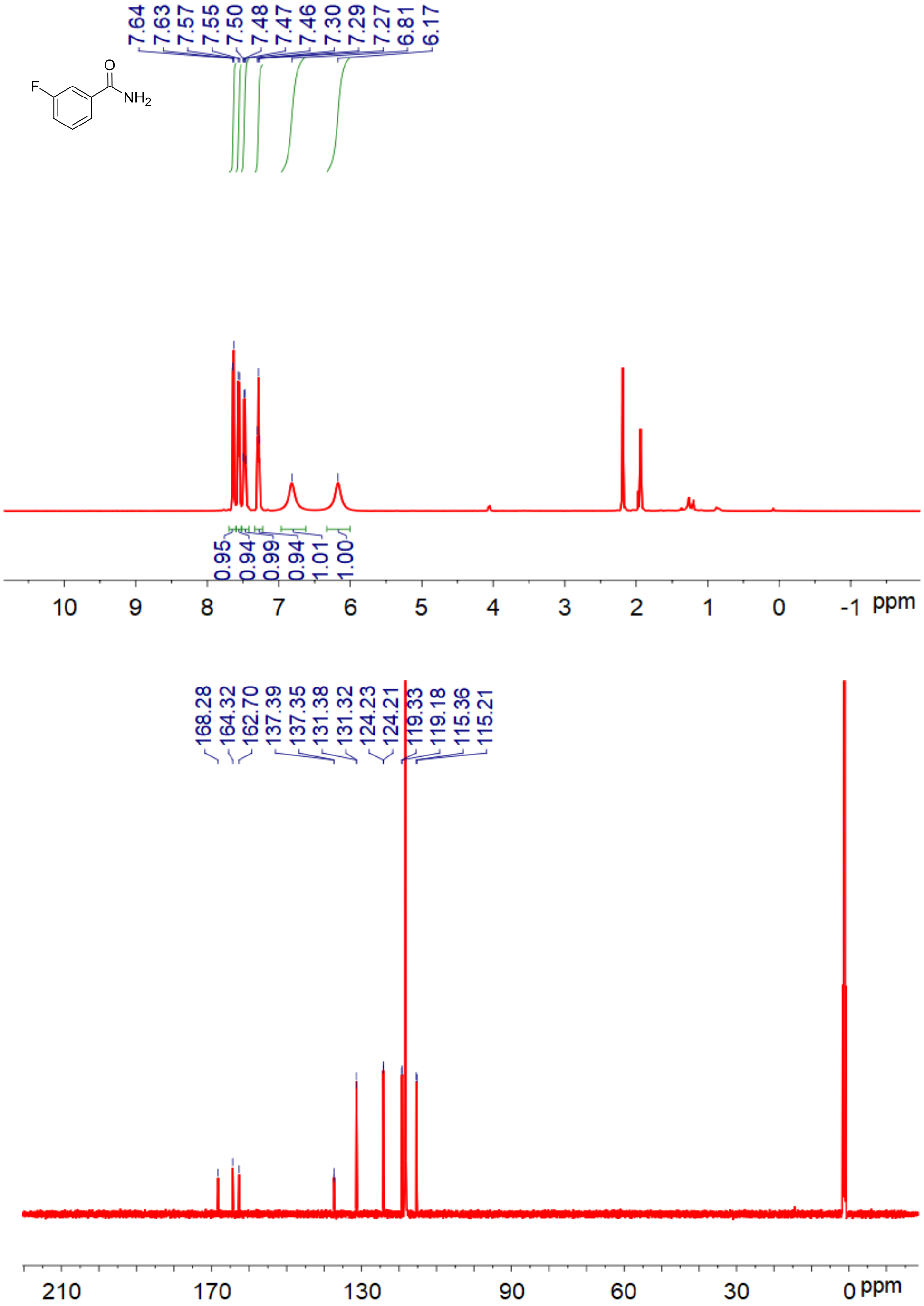

Supplementary Figure 26. ^{1}H NMR and ^{13}C NMR spectrum of 3-fluorobenzamide (**6**).

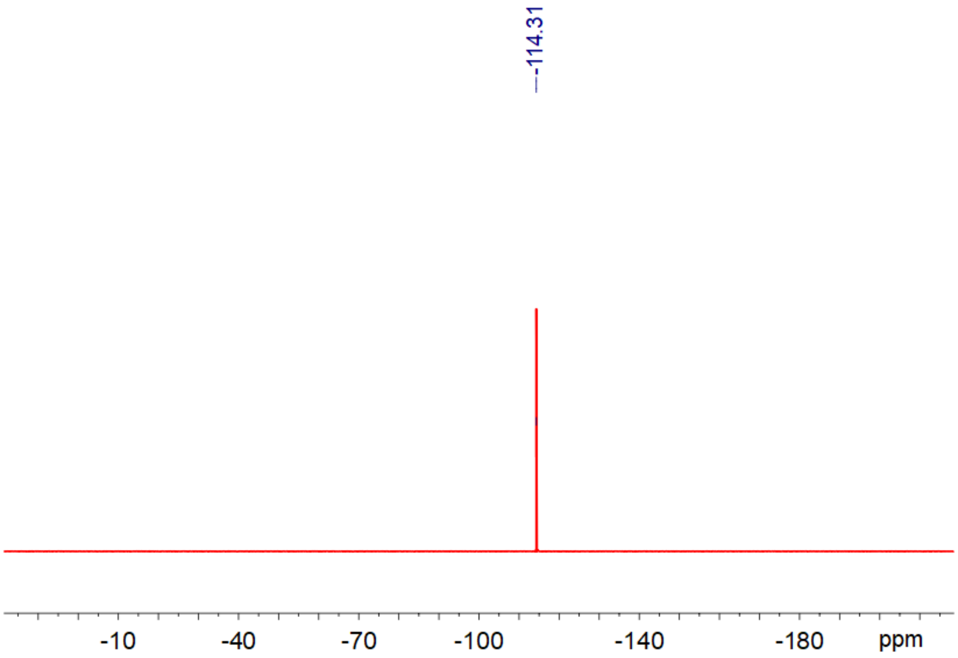

Supplementary Figure 27. ^{19}F NMR spectrum of 3-fluorobenzamide (**6**).

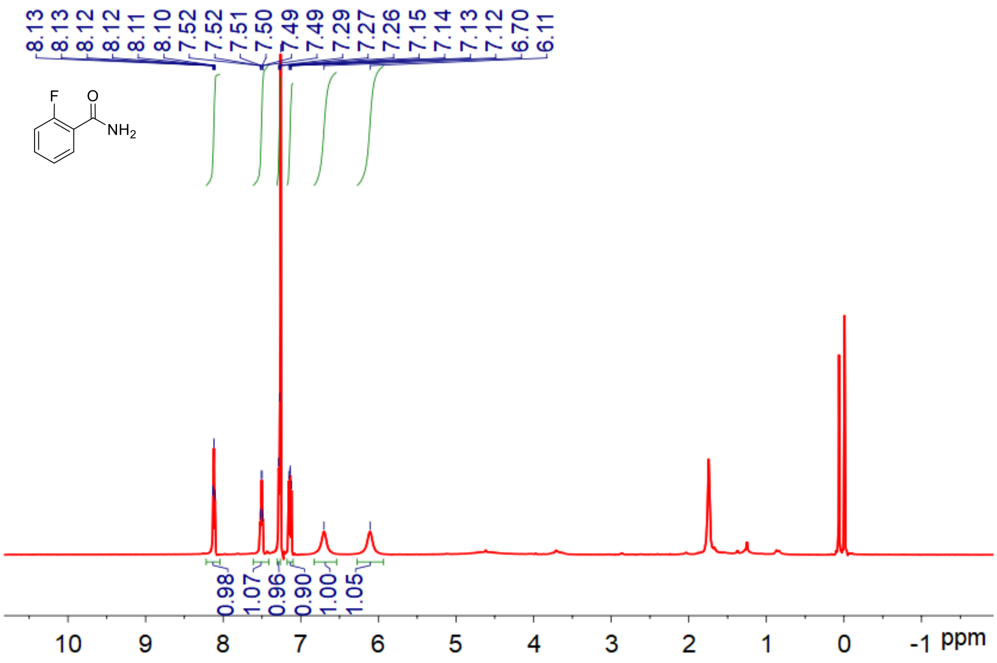

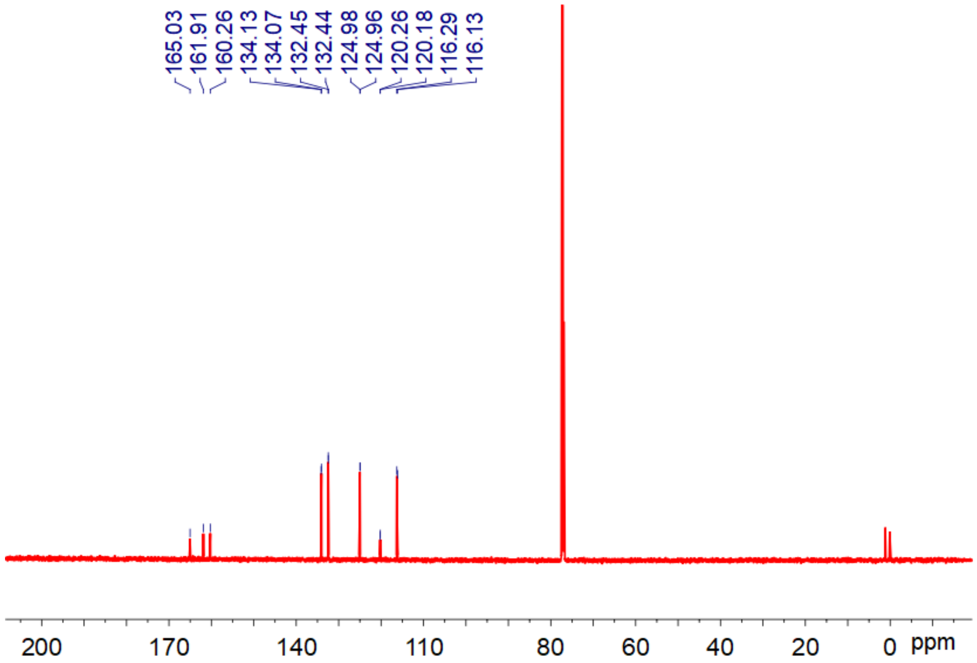

Supplementary Figure 28. ^{1}H NMR and ^{13}C NMR spectrum of 2-fluorobenzamide (**7**).

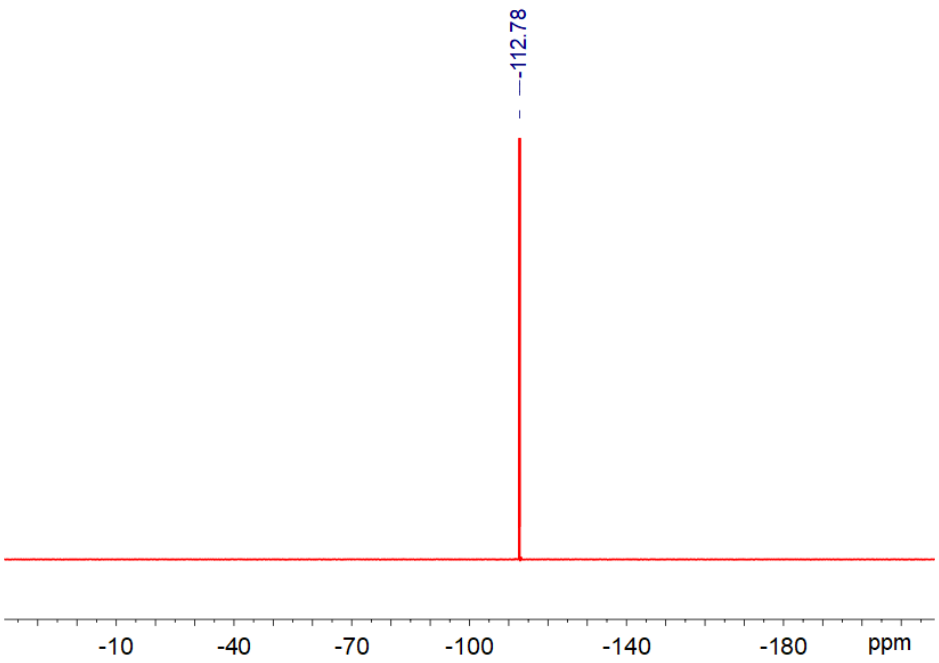

Supplementary Figure 29. ^{19}F NMR spectrum of 2-fluorobenzamide (**7**).

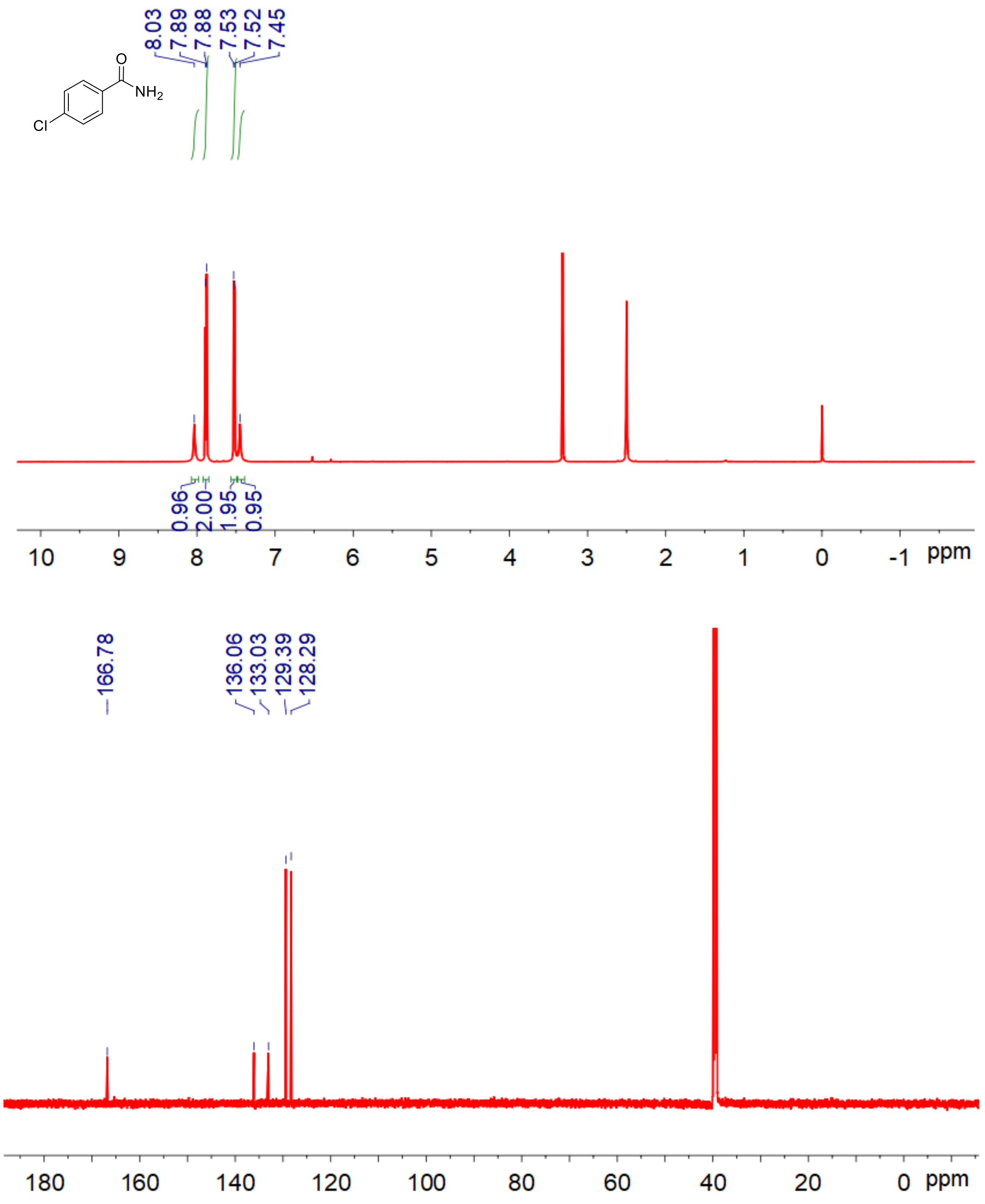

Supplementary Figure 30. ^{1}H NMR and ^{13}C NMR spectrum of 4-chlorobenzamide (**8**).

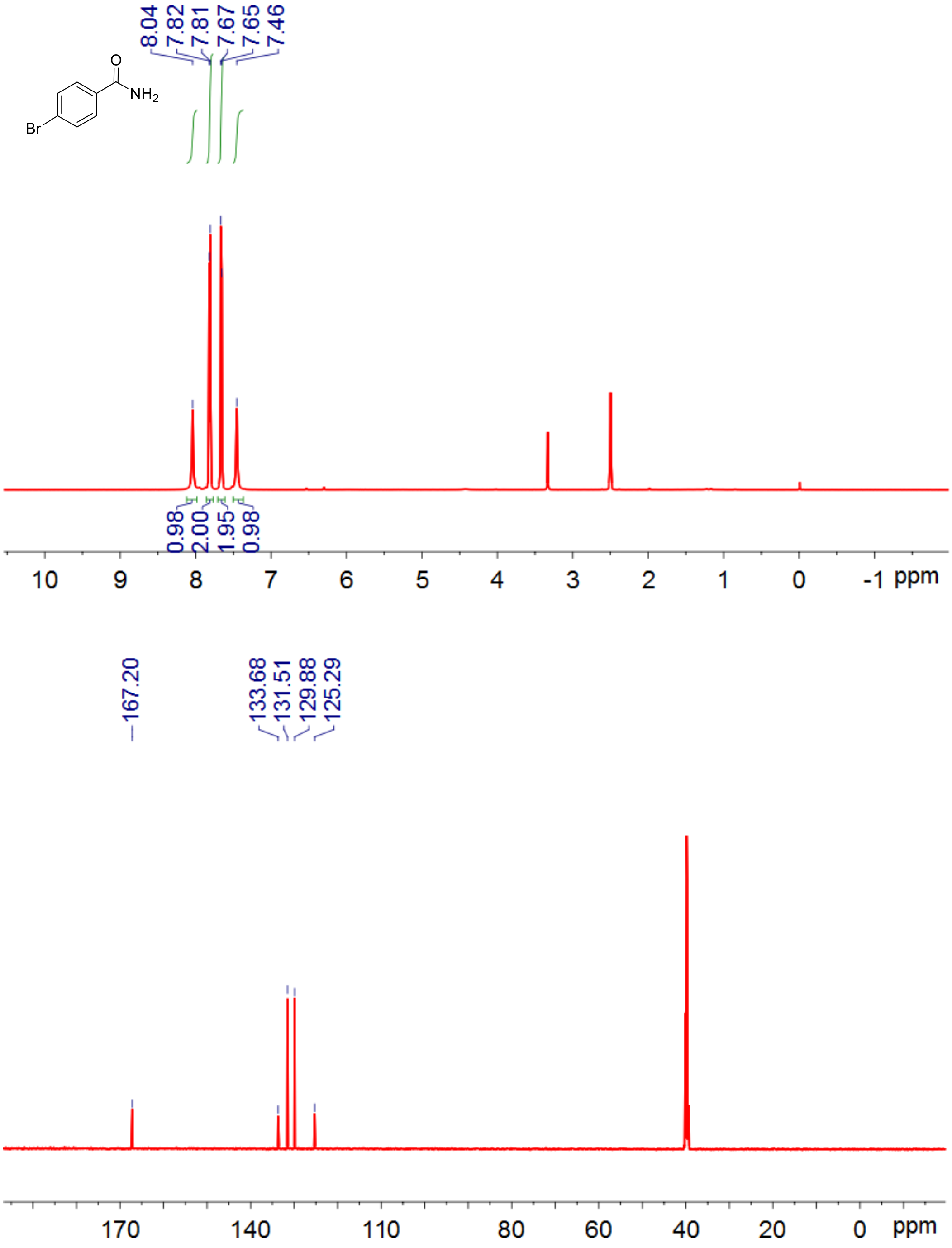

Supplementary Figure 31. ^{1}H NMR and ^{13}C NMR spectrum of 4-bromobenzamide (**9**).

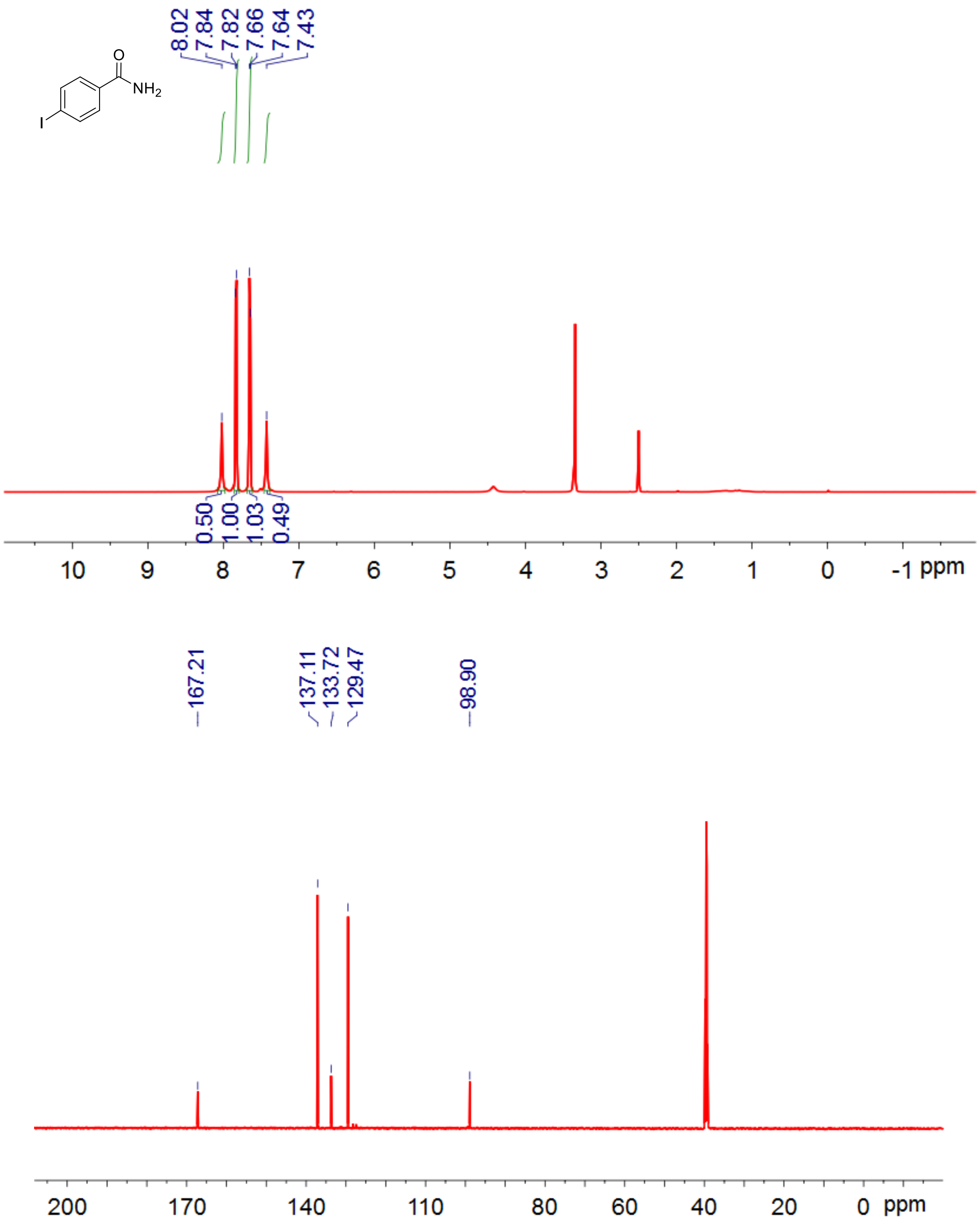

Supplementary Figure 32. ^{1}H NMR and ^{13}C NMR spectrum of 4-iodobenzamide (**10**).

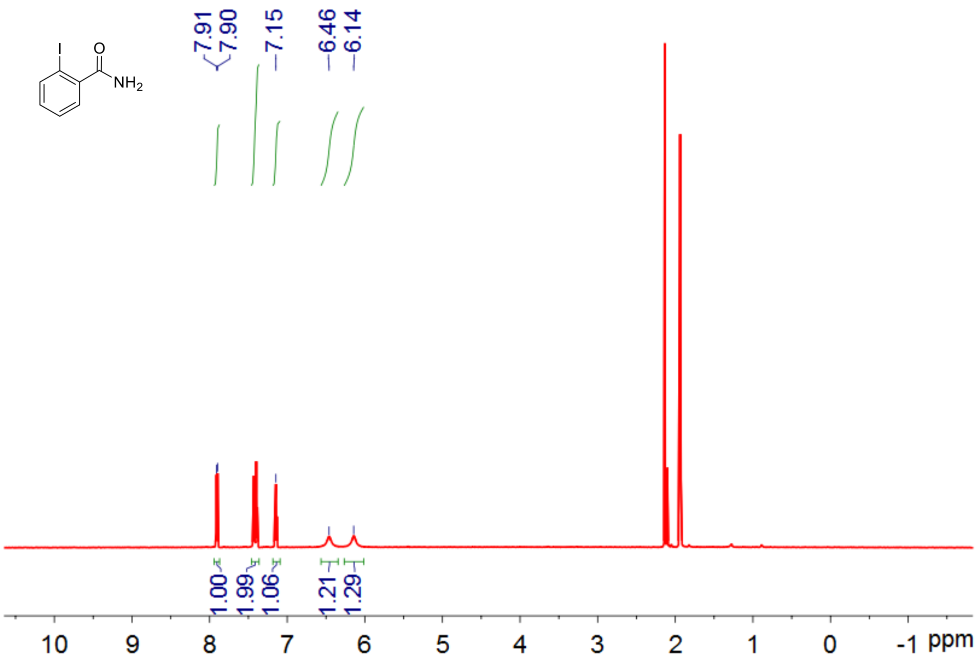

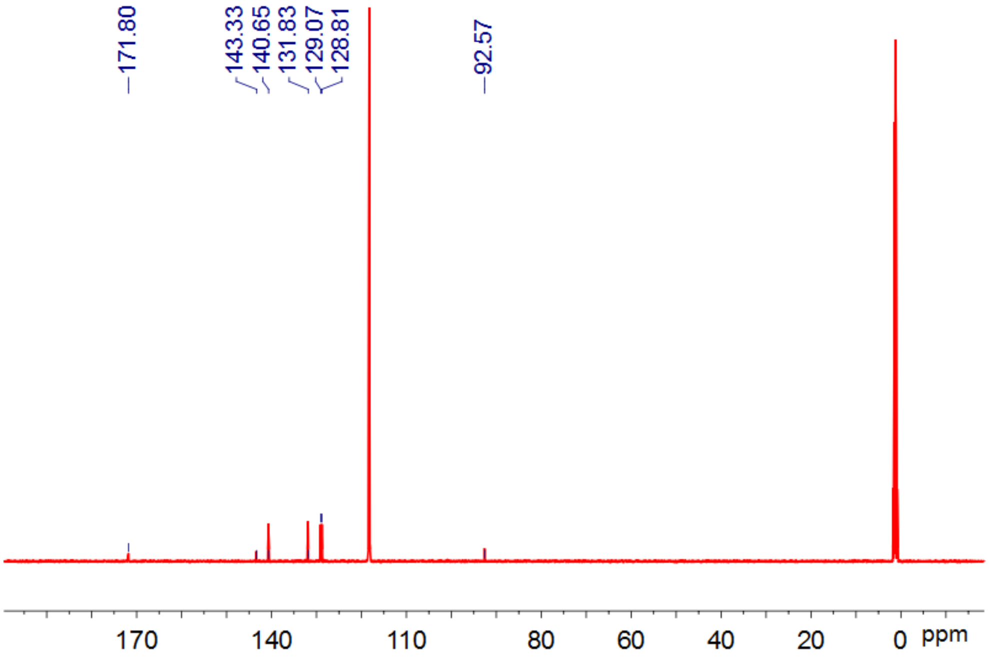

Supplementary Figure 33. ^{1}H NMR and ^{13}C NMR spectrum of 2-iodobenzamide (**11**).

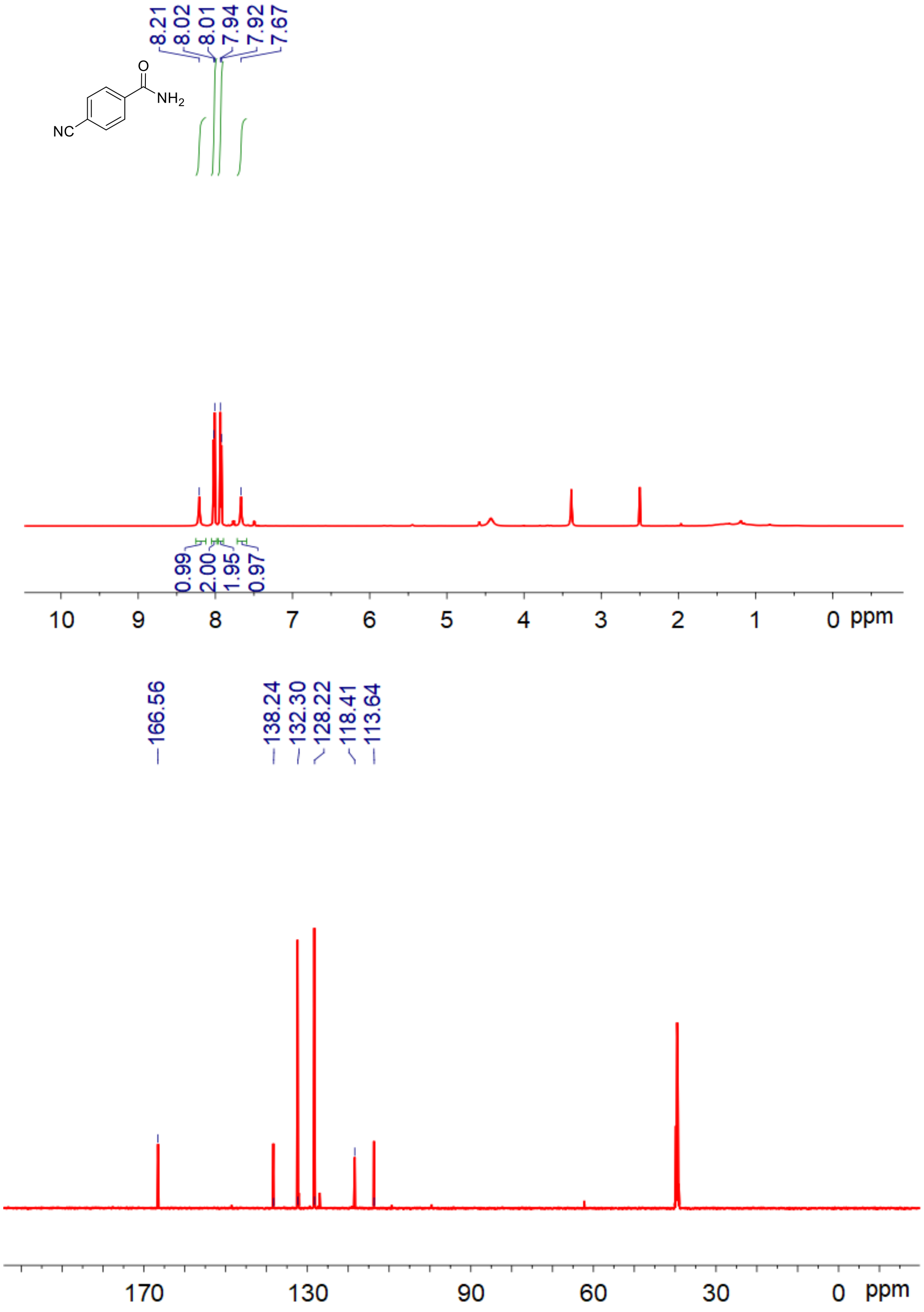

Supplementary Figure 34. ^{1}H NMR and ^{13}C NMR spectrum of 4-cyanobenzamide (**12**).

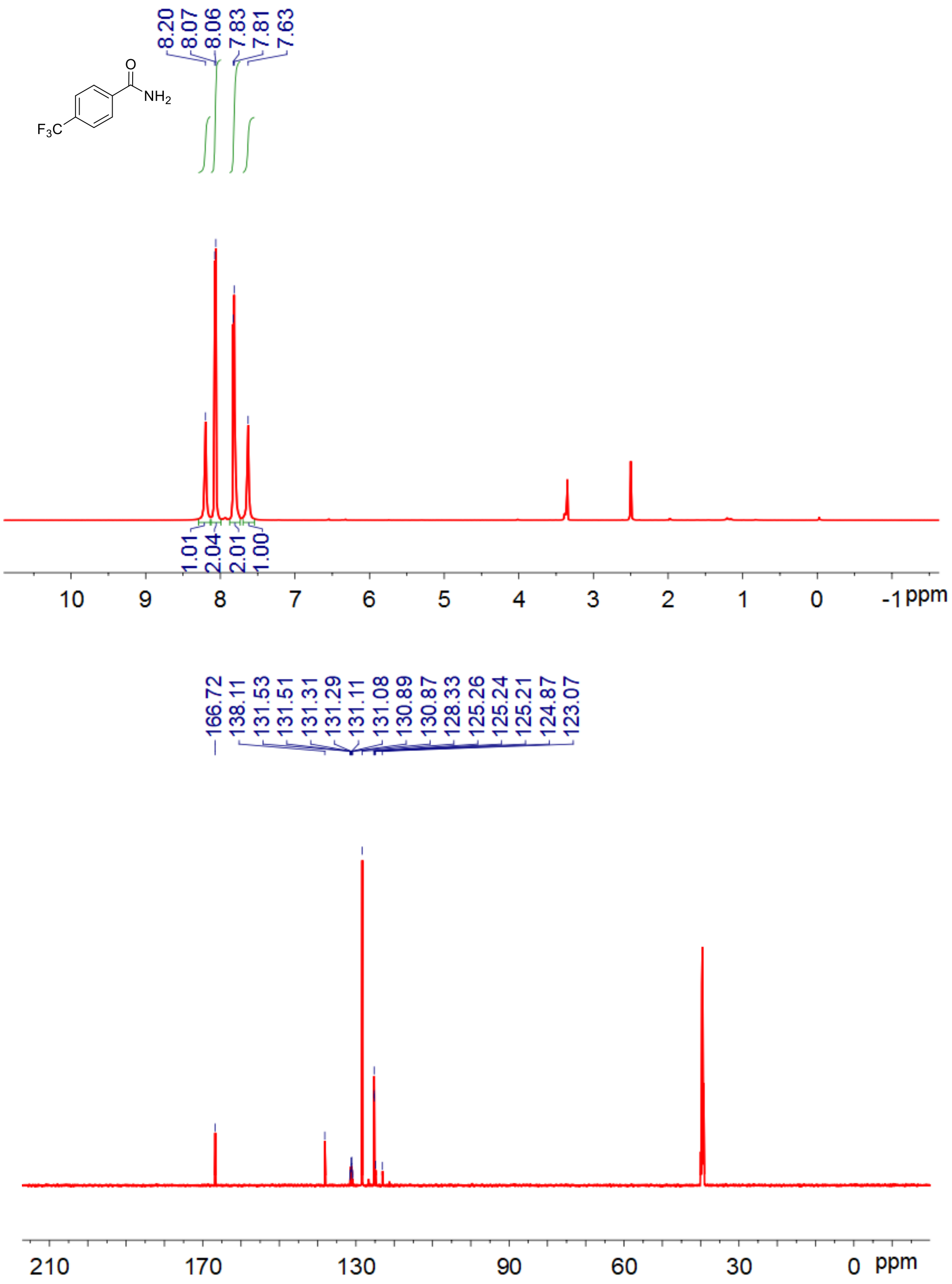

Supplementary Figure 35. ^{1}H NMR and ^{13}C NMR spectrum of 4-trifluoromethylbenzamide (**13**).

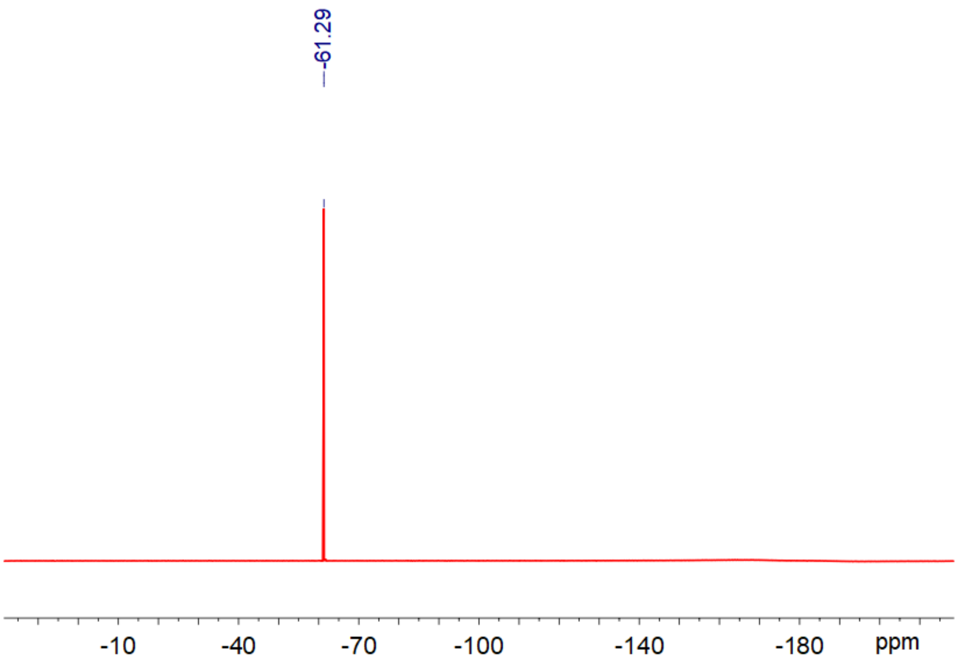

Supplementary Figure 36. ^{19}F NMR spectrum of 4-trifluoromethylbenzamide (**13**).

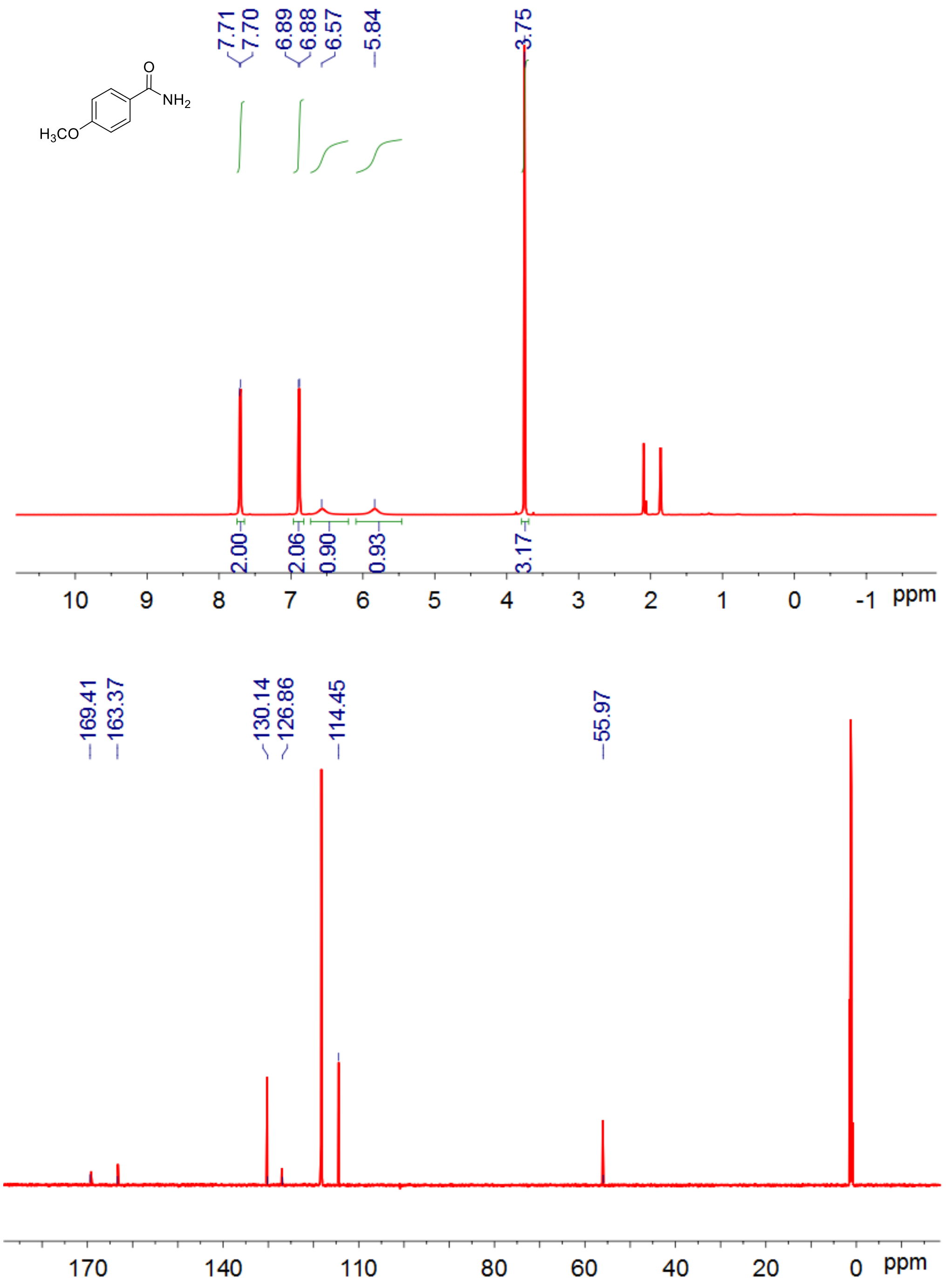

Supplementary Figure 37. ^{1}H NMR and ^{13}C NMR spectrum of 4-methoxybenzamide (**14**).

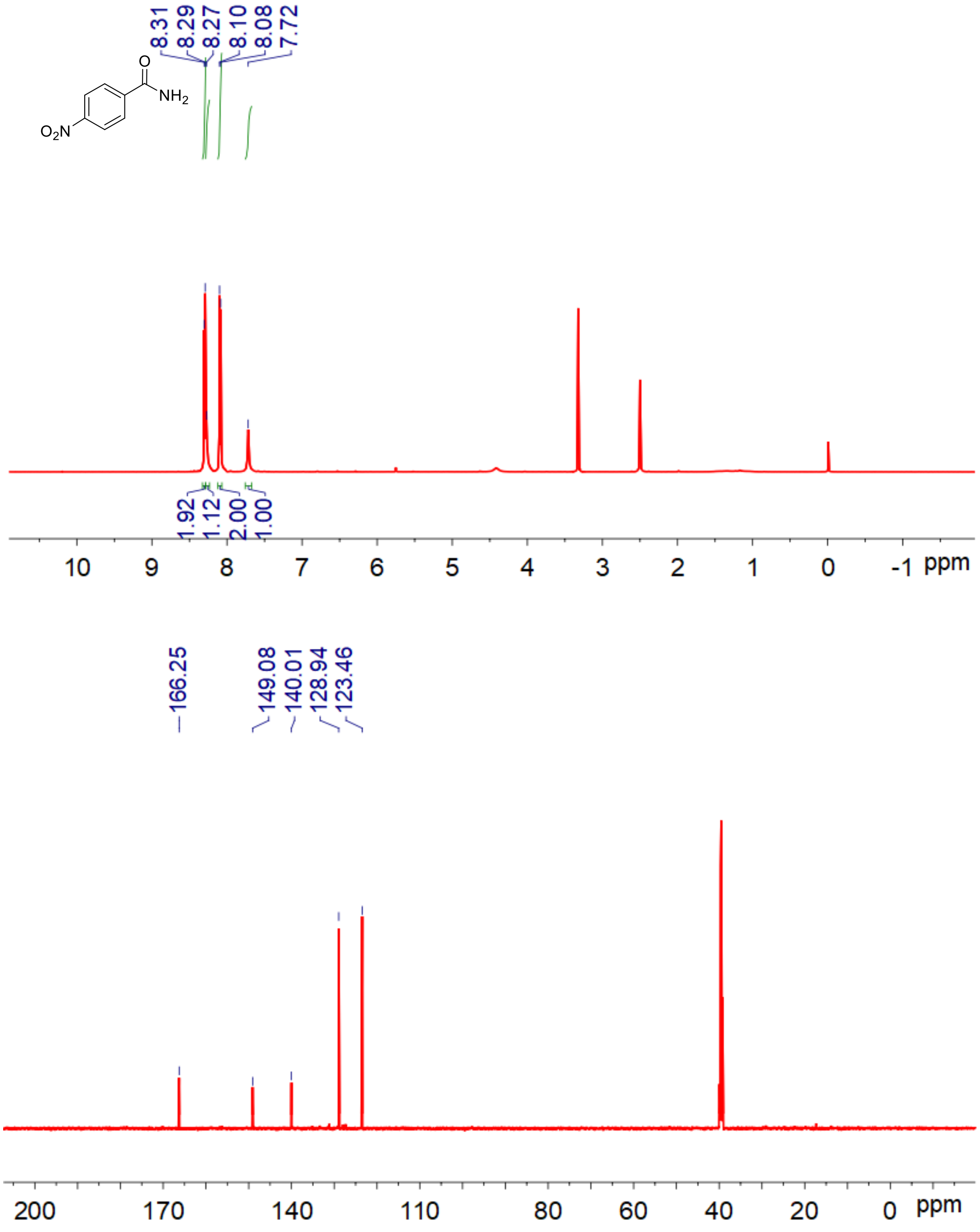

Supplementary Figure 38. ^{1}H NMR and ^{13}C NMR spectrum of 4-nitrobenzamide (**15**).

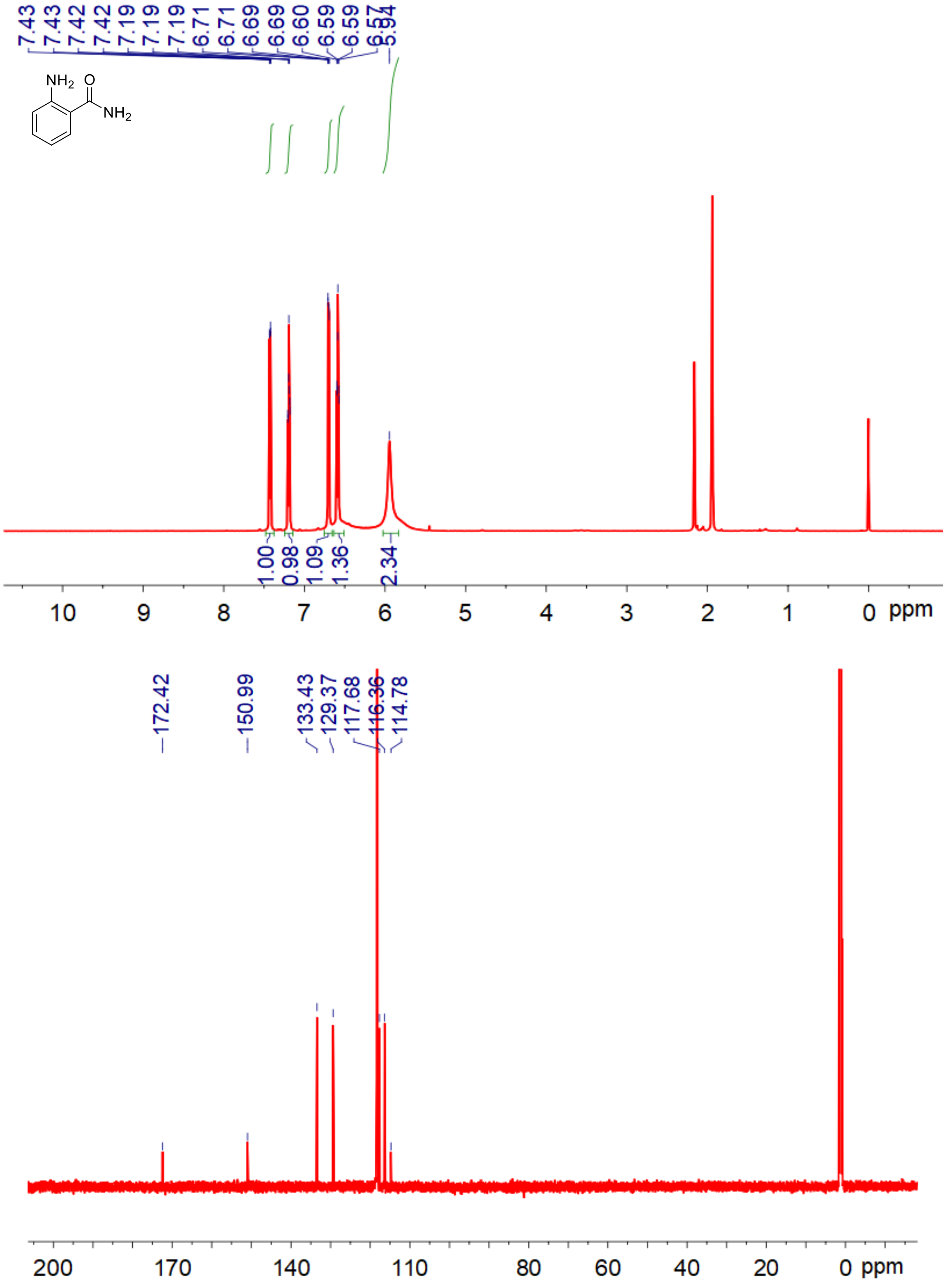

Supplementary Figure 39. ^{1}H NMR and ^{13}C NMR spectrum of 2-aminobenzamide (**16**).

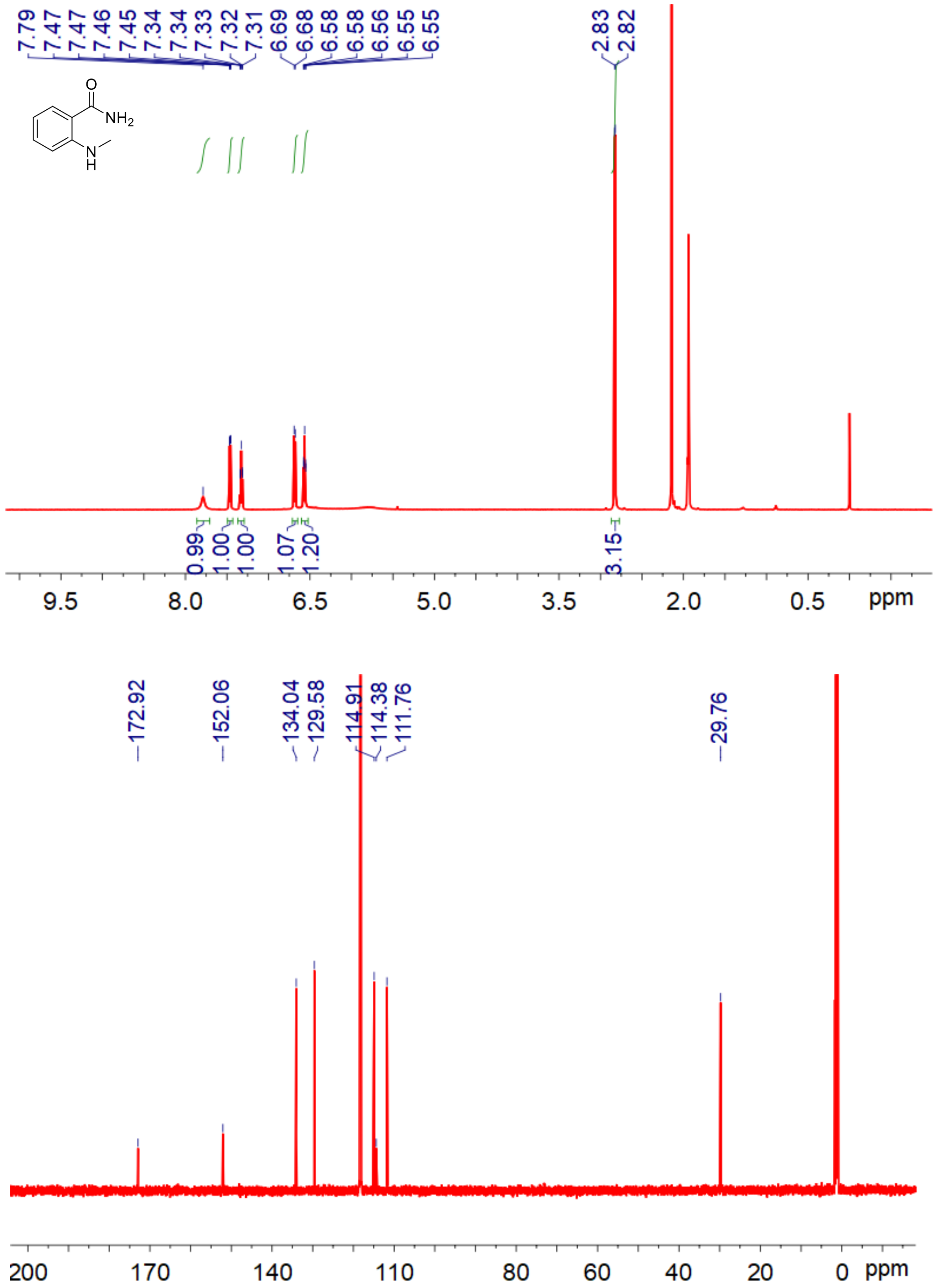

Supplementary Figure 40. ^{1}H NMR and ^{13}C NMR spectrum of 2-(methylamino)benzamide (**17**).

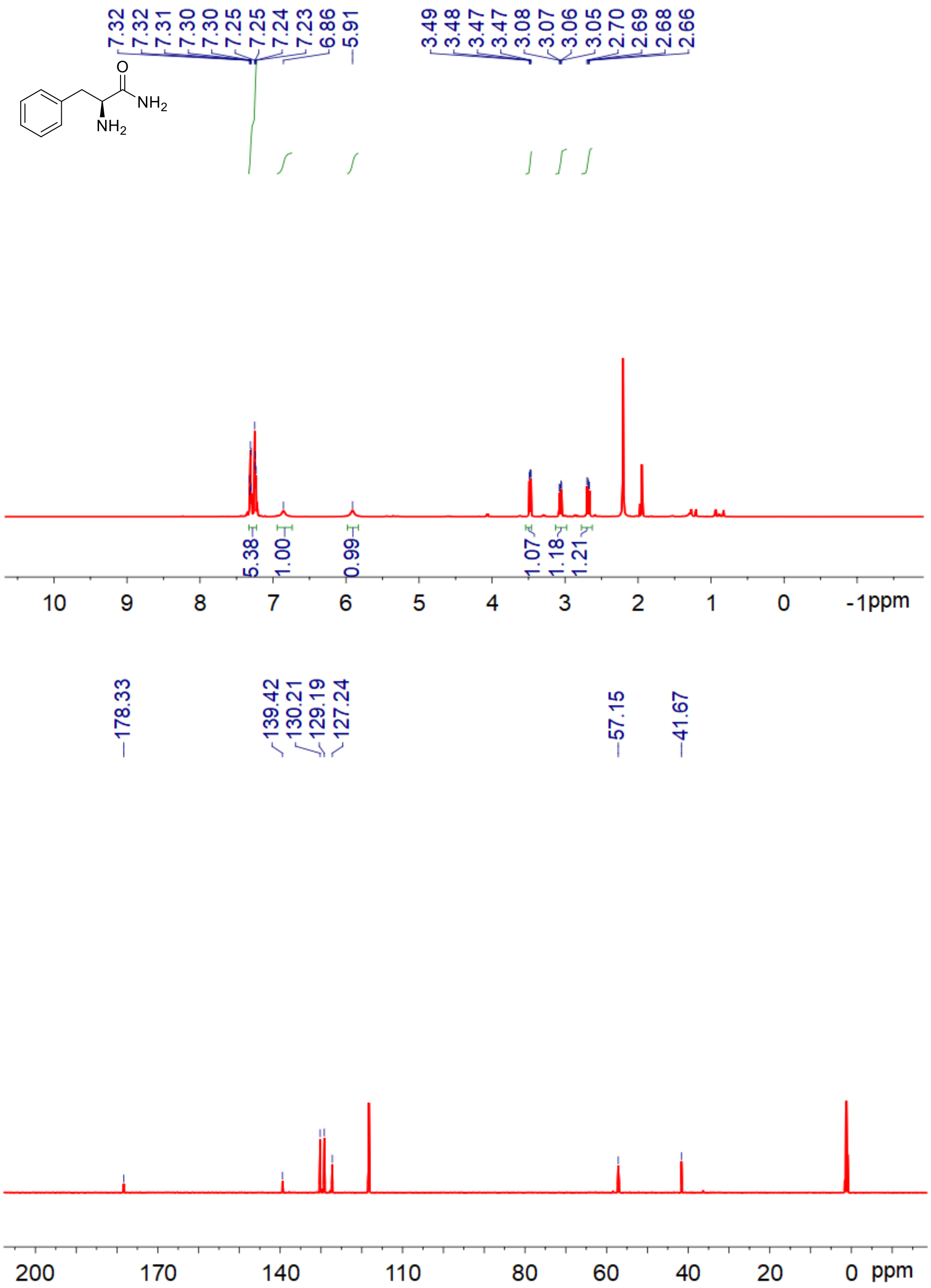

Supplementary Figure 41. ^{1}H NMR and ^{13}C NMR spectrum of L-phenylalaninamide (**18**).

Chiral HPLC Assay:

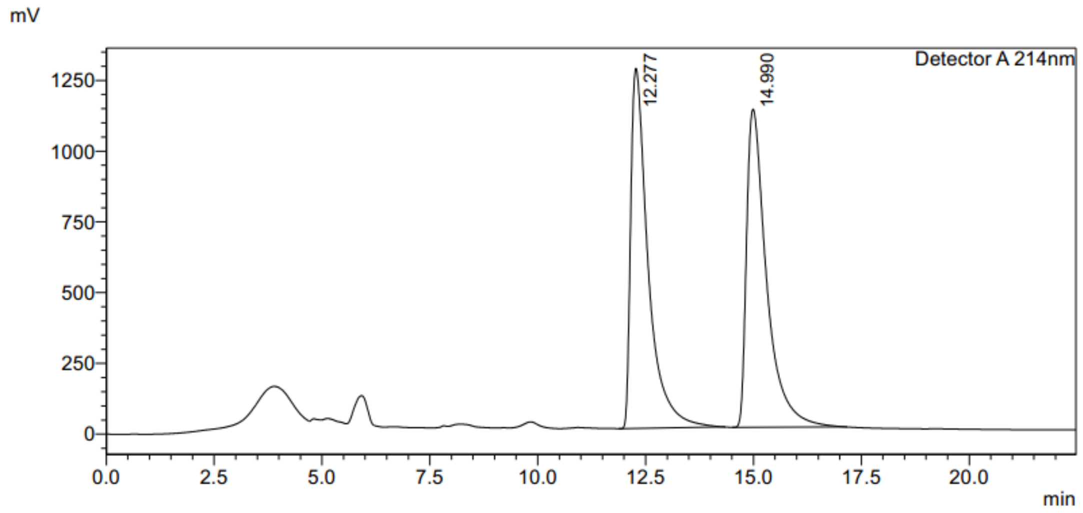

<Peak Table>

Detector A 214nm

Peak#	Ret. Time	Area	Height	Conc.	Unit	Mark	Name
1	12.277	35858522	1271519	49.650		M	
2	14.990	36364182	1123904	50.350		M	
Total		72222704	2395423				

Supplementary Figure 42. HPLC data of D, L-phenylalaninamide.

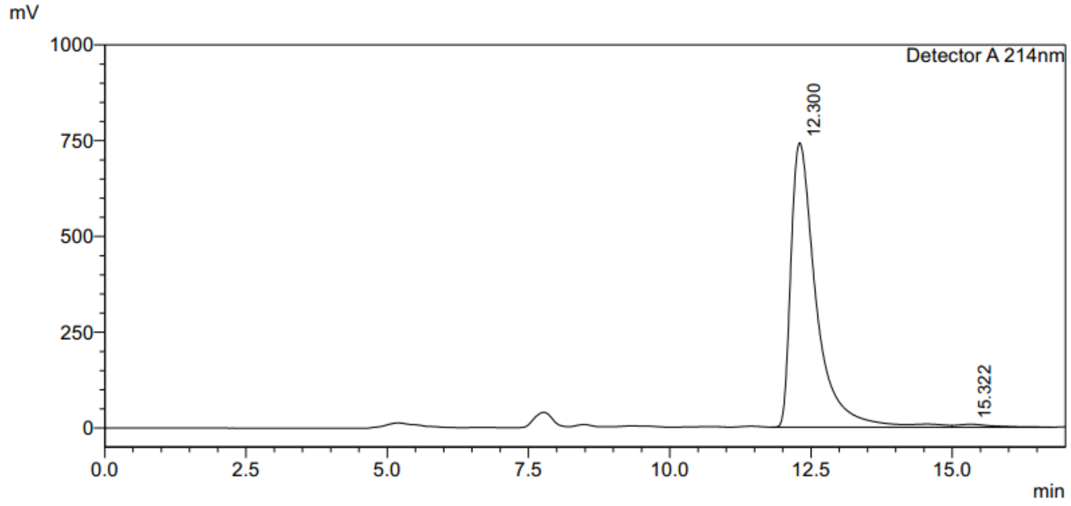

<Peak Table>

Detector A 214nm

Peak#	Ret. Time	Area	Height	Conc.	Unit	Mark	Name
1	12.300	23228548	742292	98.645		M	
2	15.322	319053	7632	1.355		V M	
Total		23547602	749925				

Supplementary Figure 43. HPLC data of L-phenylalaninamide (**18**).

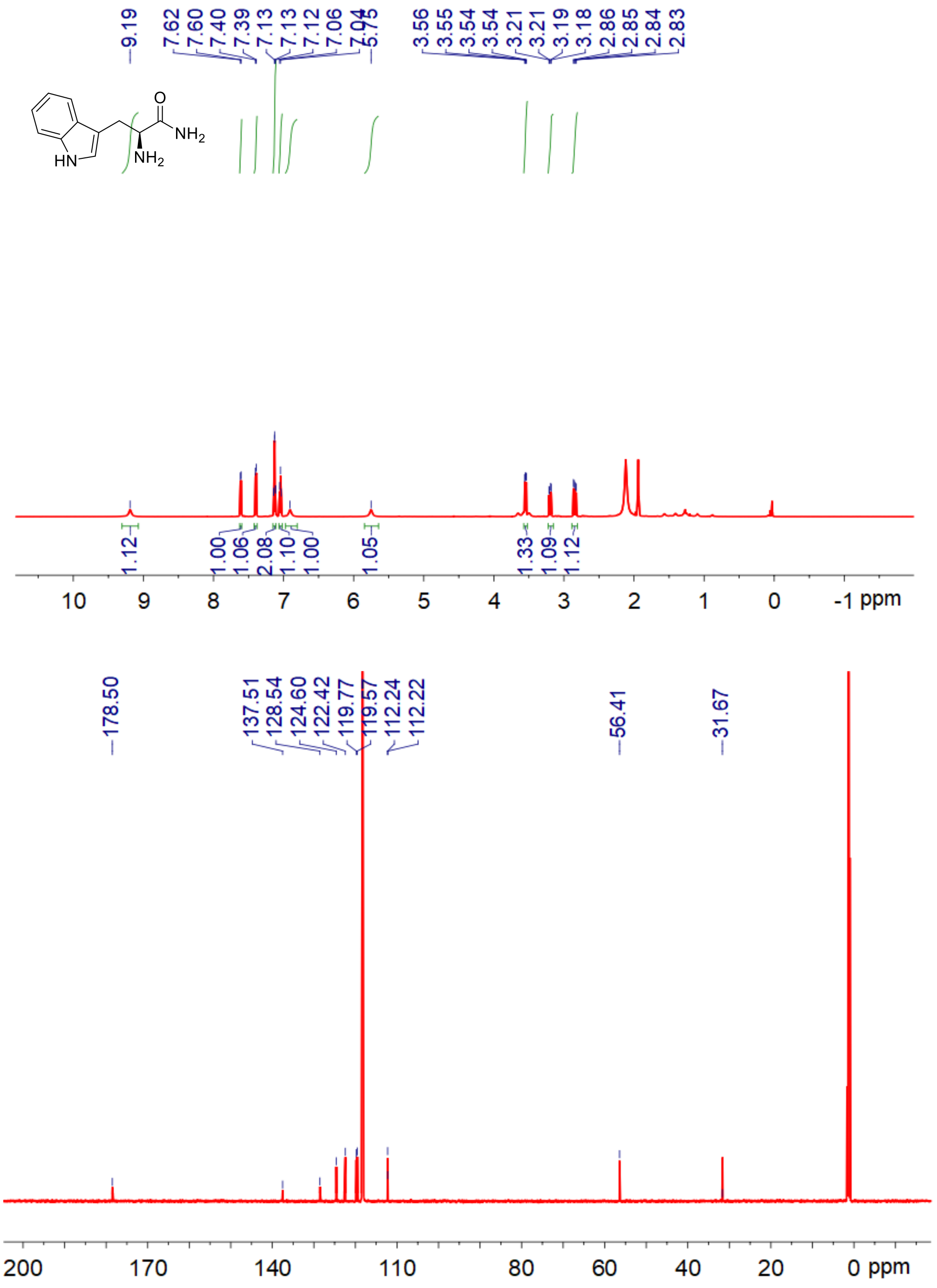

Supplementary Figure 44. ^{1}H NMR and ^{13}C NMR spectrum of L-tryptophanamide (**19**).

Chiral HPLC Assay:

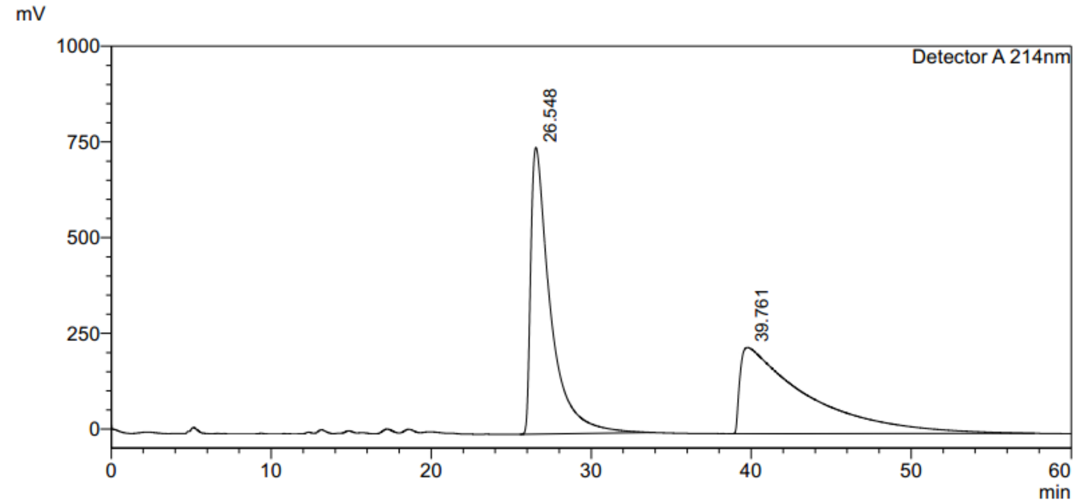

<Peak Table>

Detector A 214nm

Peak#	Ret. Time	Area	Height	Conc.	Unit	Mark	Name
1	26.548	64192561	748972	49.839		M	
2	39.761	64607661	225018	50.161		M	
Total		128800222	973990				

Supplementary Figure 45. HPLC data of D, L-tryptophanamide.

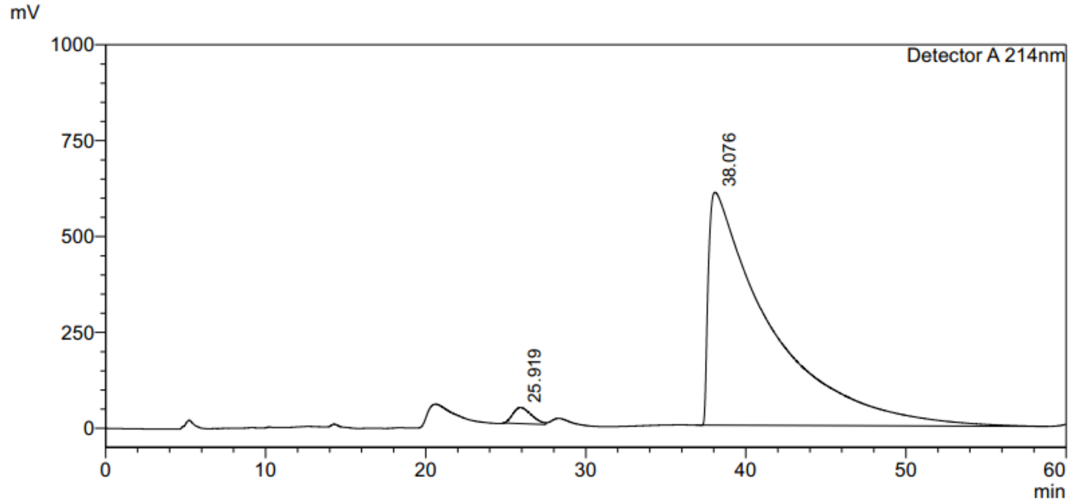

<Peak Table>

Detector A 214nm

Peak#	Ret. Time	Area	Height	Conc.	Unit	Mark	Name
1	25.919	3398882	41996	2.054		M	
2	38.076	162063683	607286	97.946		M	
Total		165462565	649282				

Supplementary Figure 46. HPLC data of L-tryptophanamide (**19**).

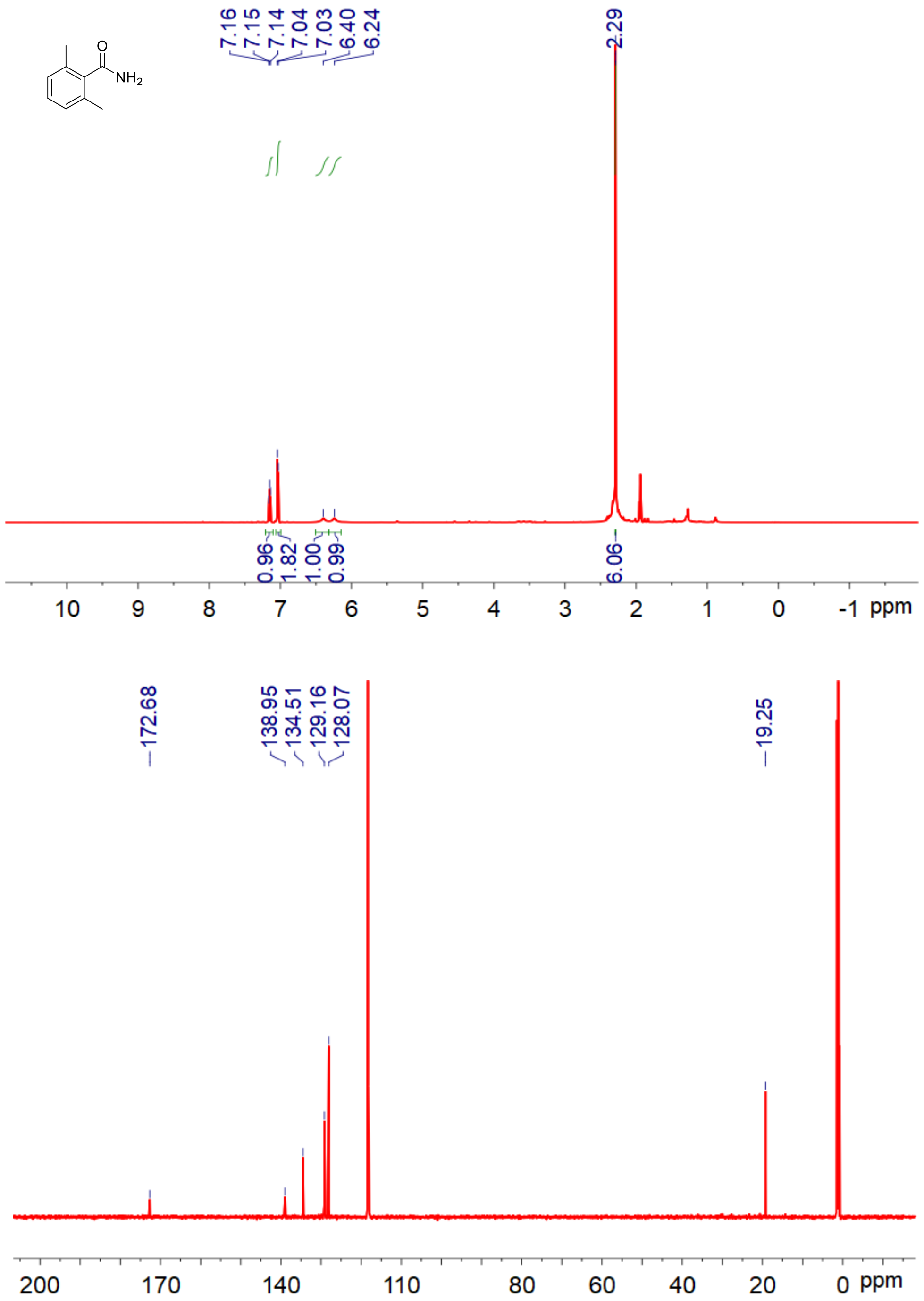

Supplementary Figure 47. ^{1}H NMR and ^{13}C NMR spectrum of 2,6-dimethylbenzamide (**20**).

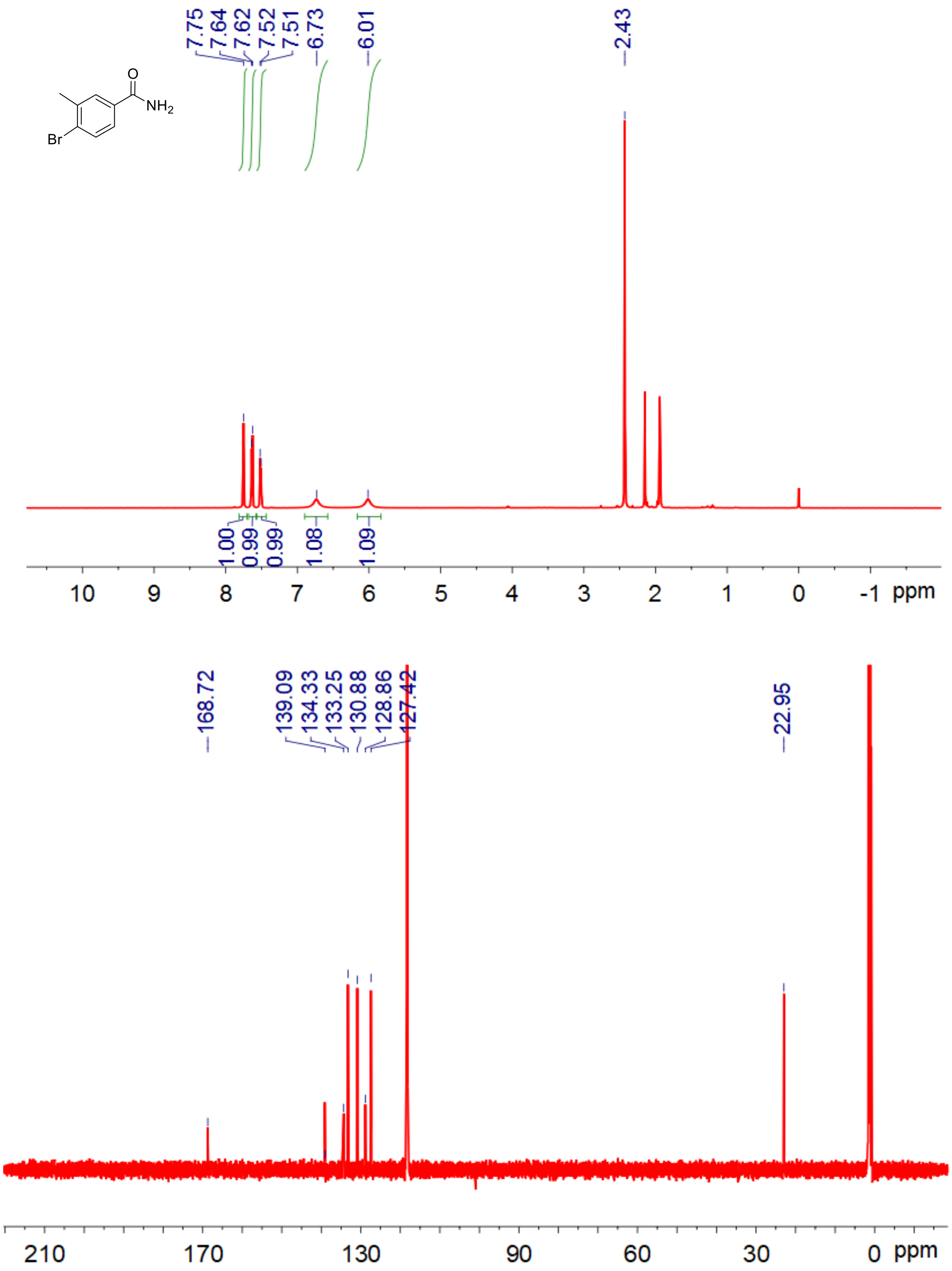

Supplementary Figure 48. ^{1}H NMR and ^{13}C NMR spectrum of 4-bromo-3-methyl-benzamide (**21**).

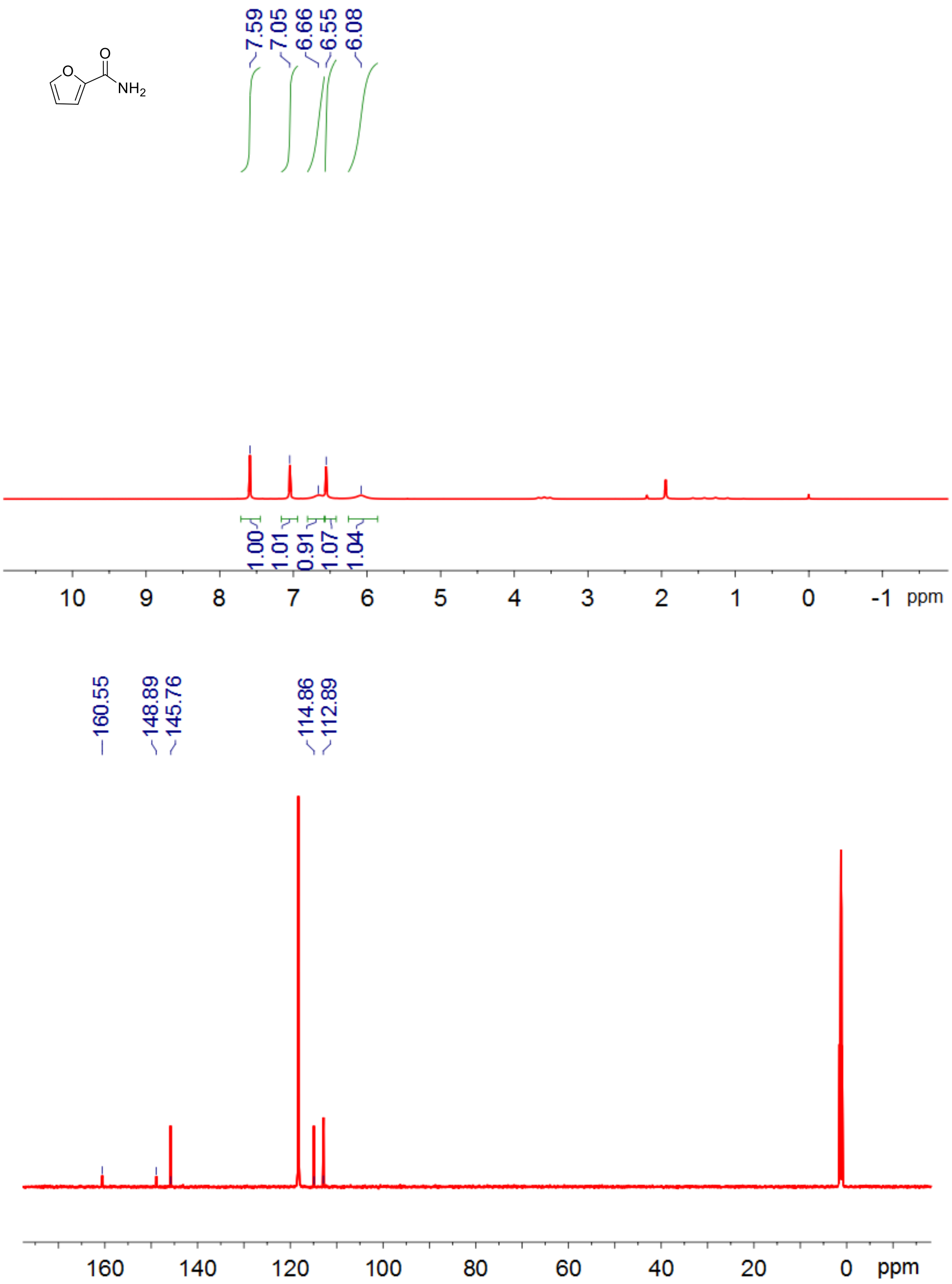

Supplementary Figure 49. ^{1}H NMR and ^{13}C NMR spectrum of 2-furancarboxamide (**22**).

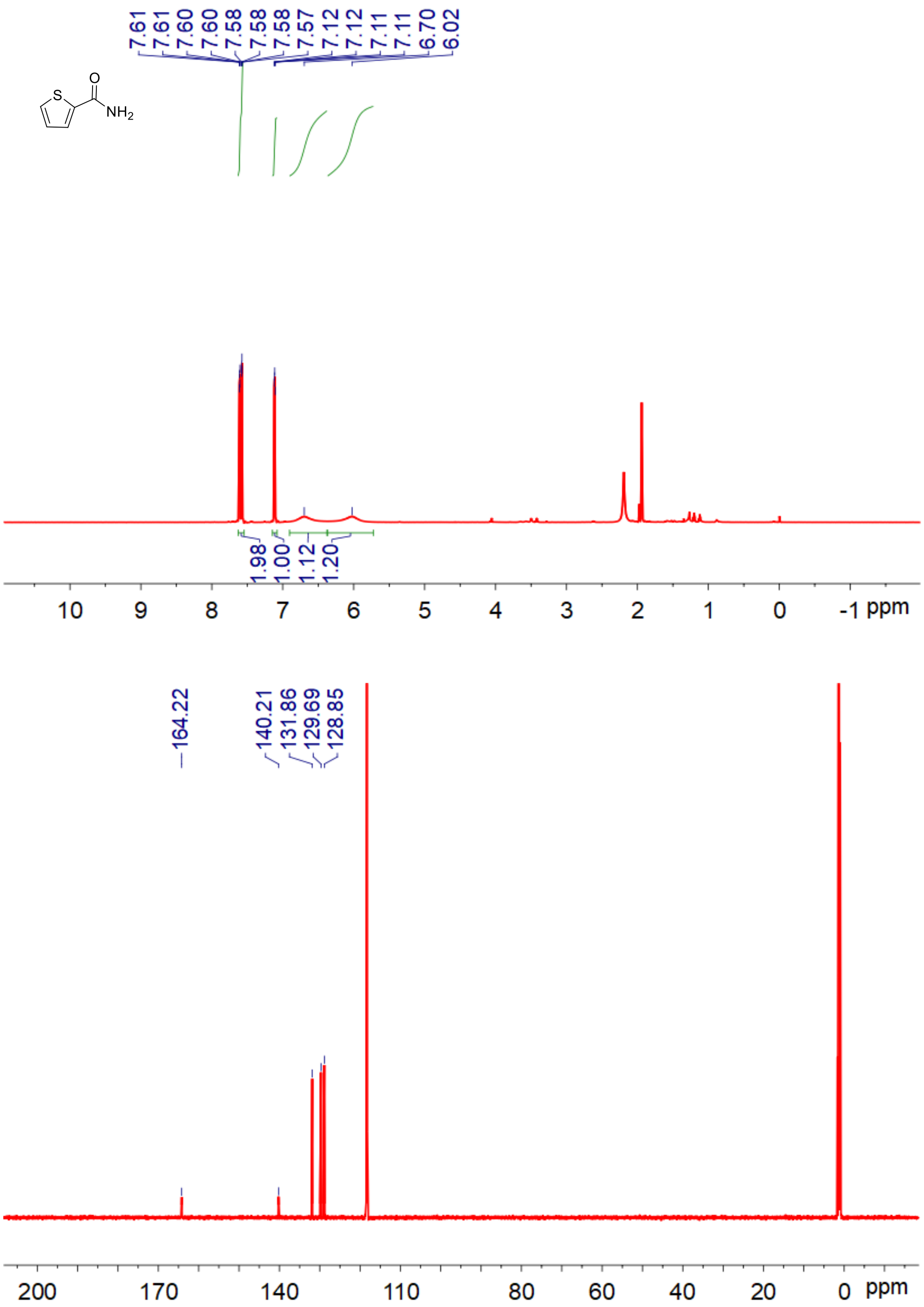

Supplementary Figure 50. ^{1}H NMR and ^{13}C NMR spectrum of 2-thiophenecarboxamide (**23**).

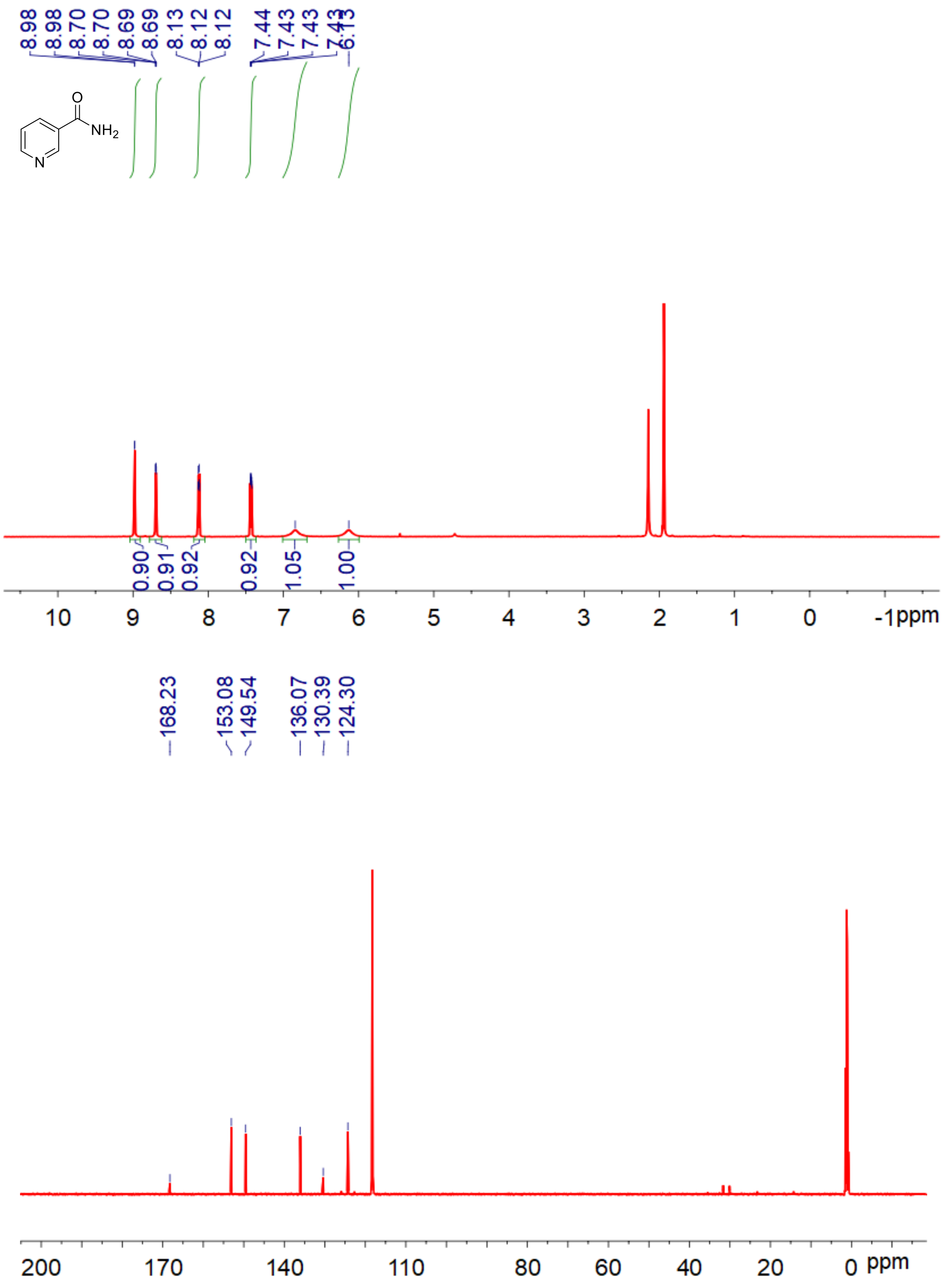

Supplementary Figure 51. ^{1}H NMR and ^{13}C NMR spectrum of nicotinamide (**24**).

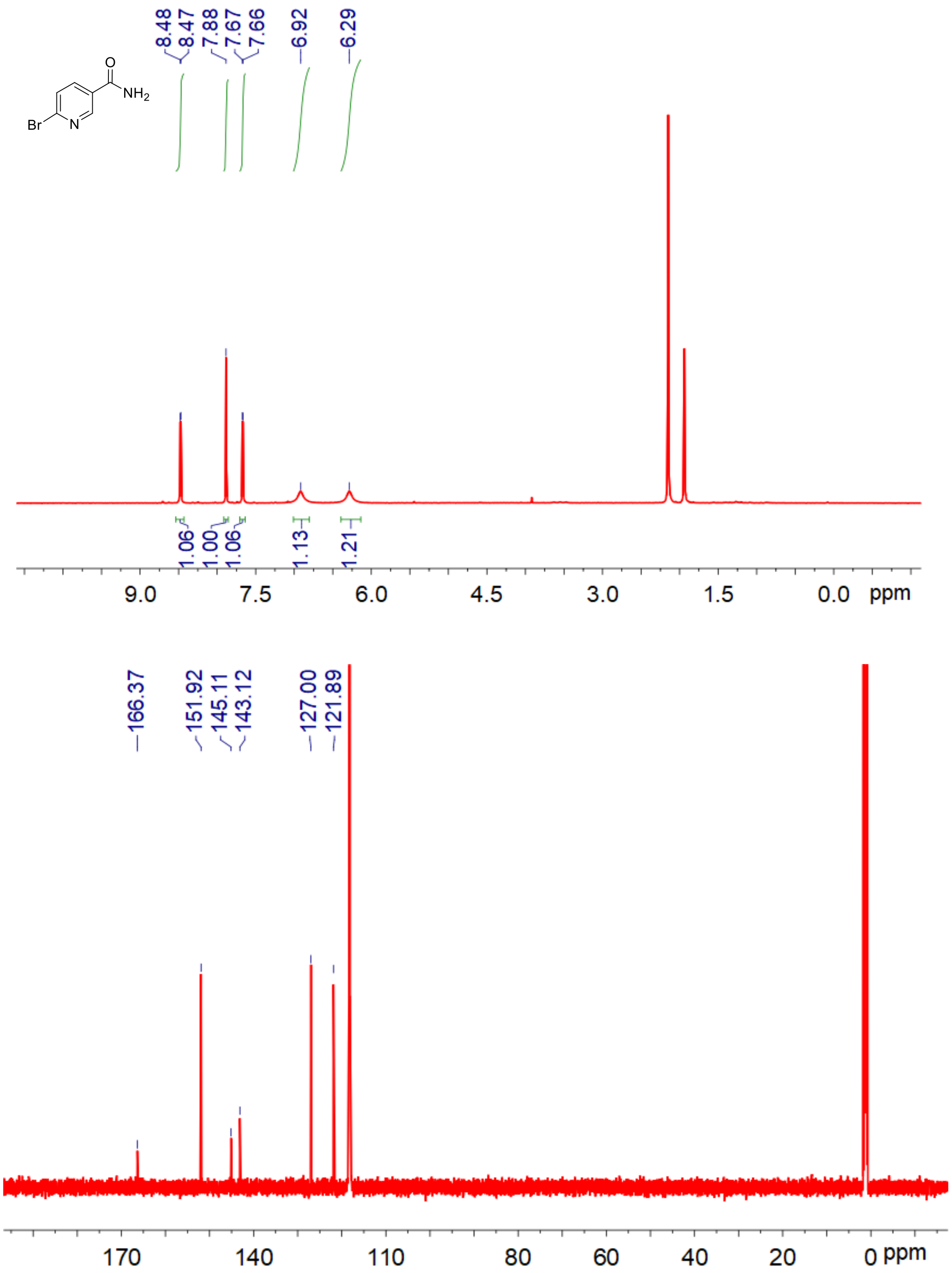

Supplementary Figure 52. ^{1}H NMR and ^{13}C NMR spectrum of 2-bromopyridine-4-formamide (**25**).

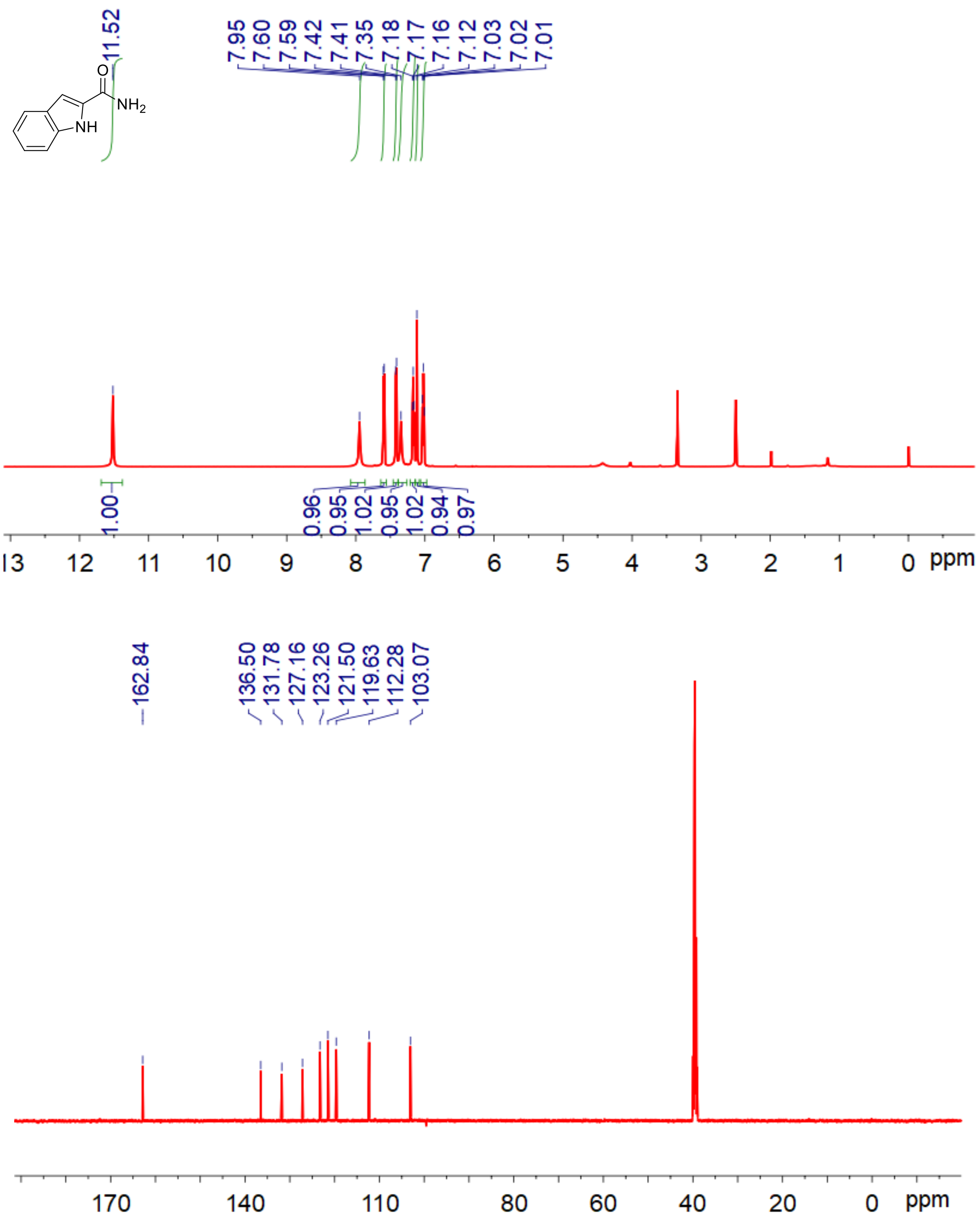

Supplementary Figure 53. ^{1}H NMR and ^{13}C NMR spectrum of indole-2-carboxylic acid formamide (**26**).

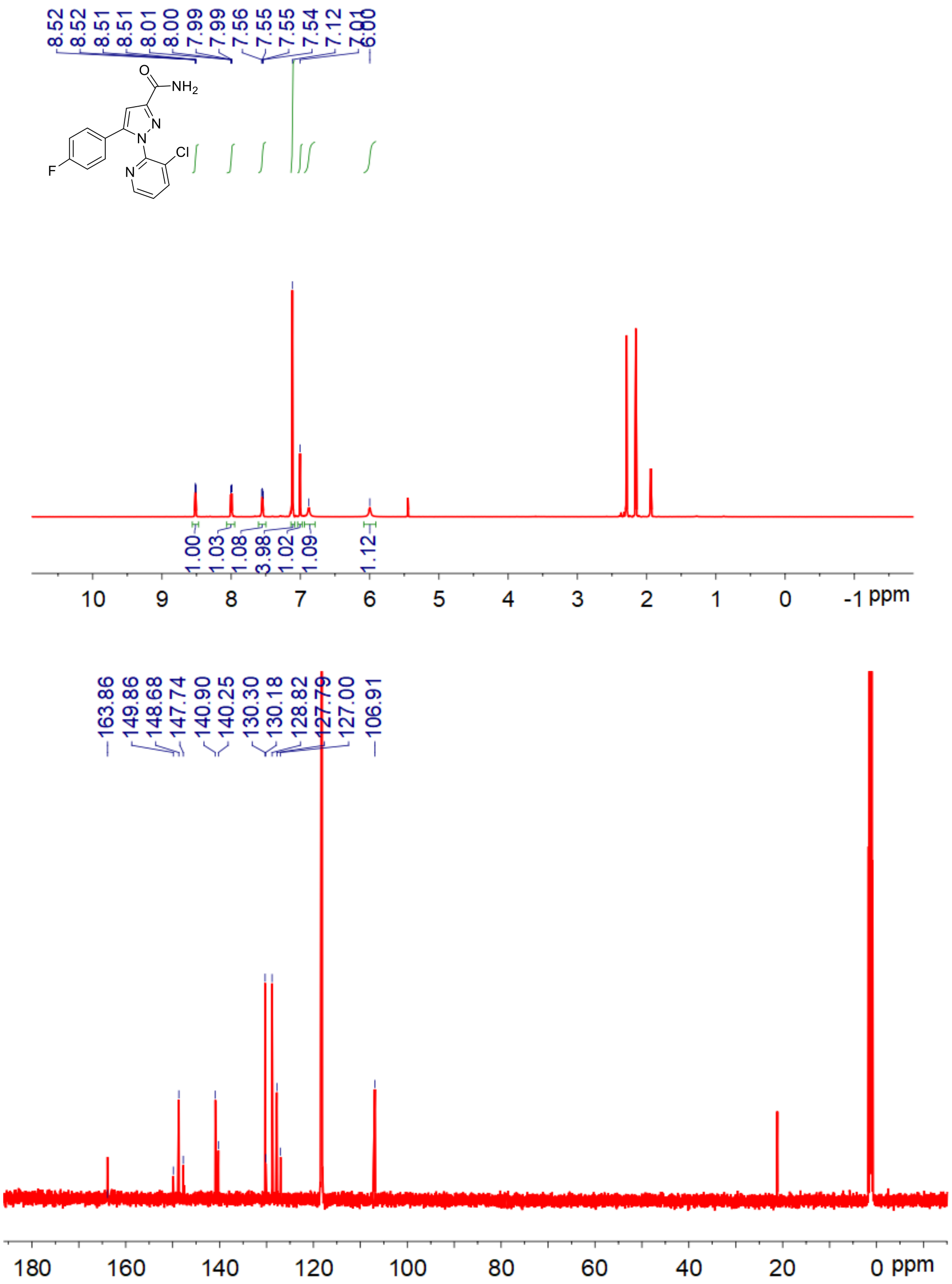

Supplementary Figure 54. ^{1}H NMR and ^{13}C NMR spectrum of 1H-pyrazole-3-carboxylic acid, 5-(4-fluorophenyl)-1-(6-Cl-2-pyridinyl)-acetamide (**27**).

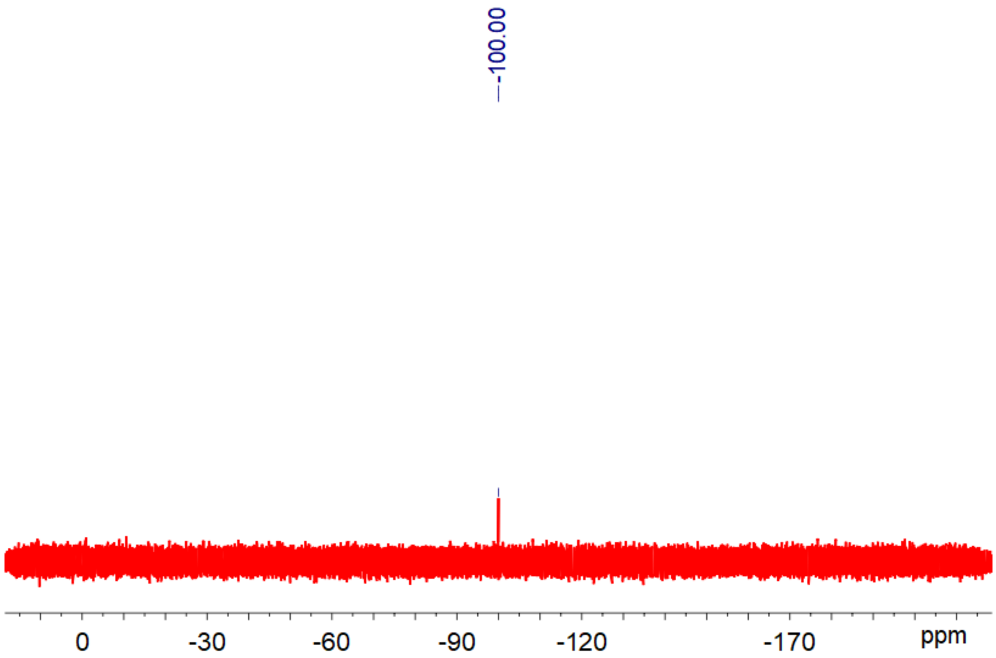

Supplementary Figure 55. ^{19}F NMR spectrum of 1H-pyrazole-3-carboxylic acid, 5-(4-fluorophenyl)-1-(6-Cl-2-pyridinyl)-acetamide (**27**).

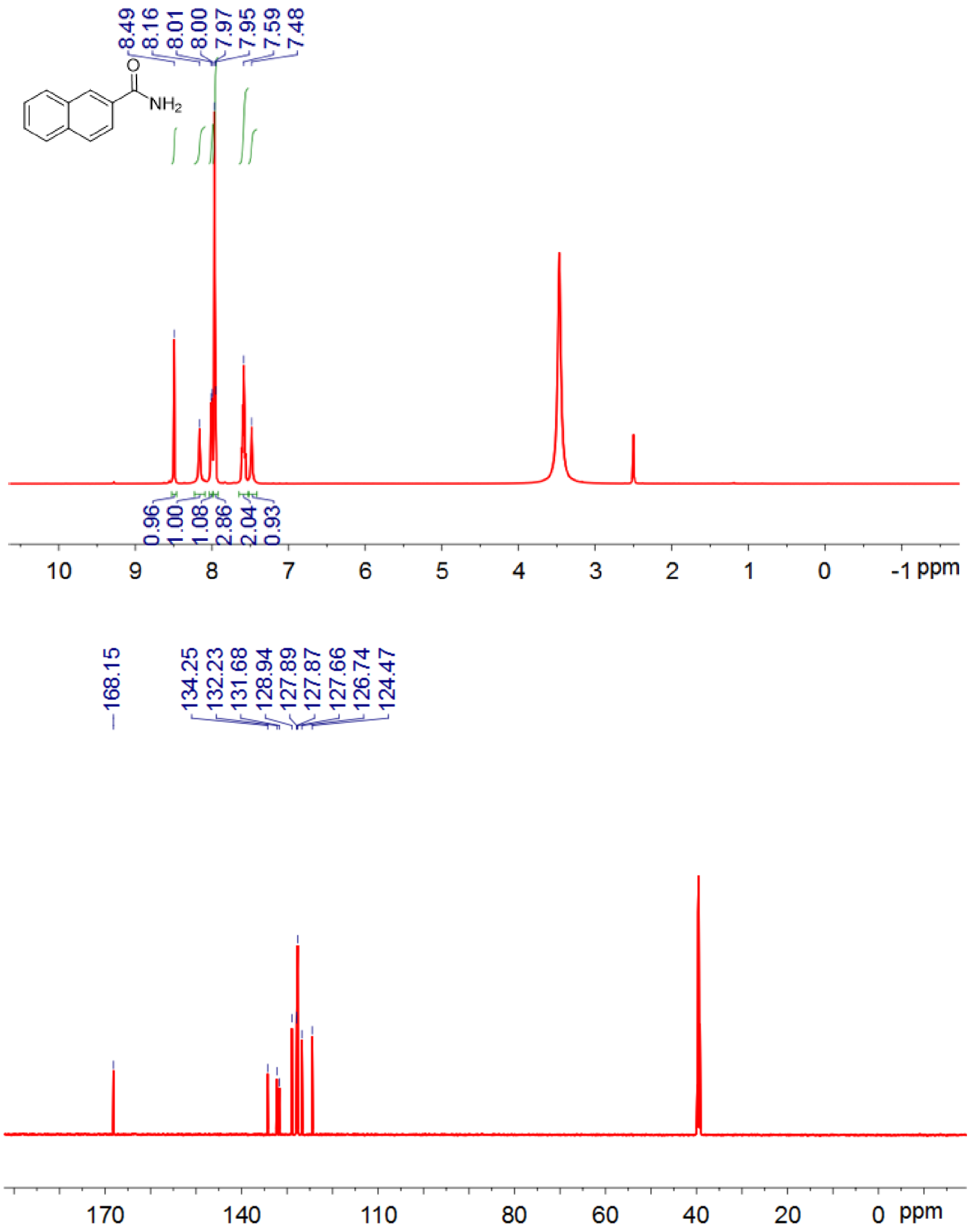

Supplementary Figure 56. ^{1}H NMR and ^{13}C NMR spectrum of 2-naphthalamide (**28**).

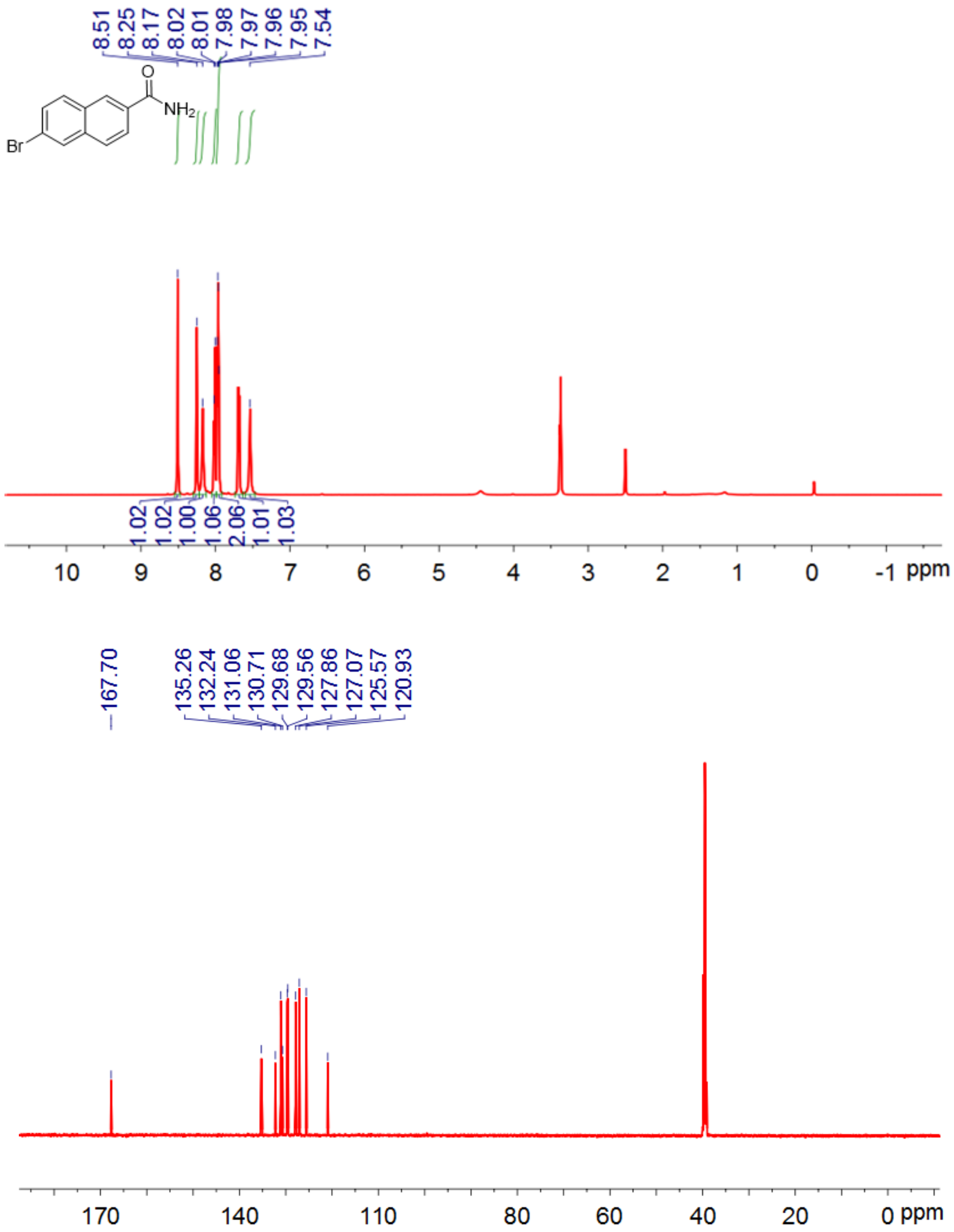

Supplementary Figure 57. ^{1}H NMR and ^{13}C NMR spectrum of 6-bromo-2-naphthamide (**29**).

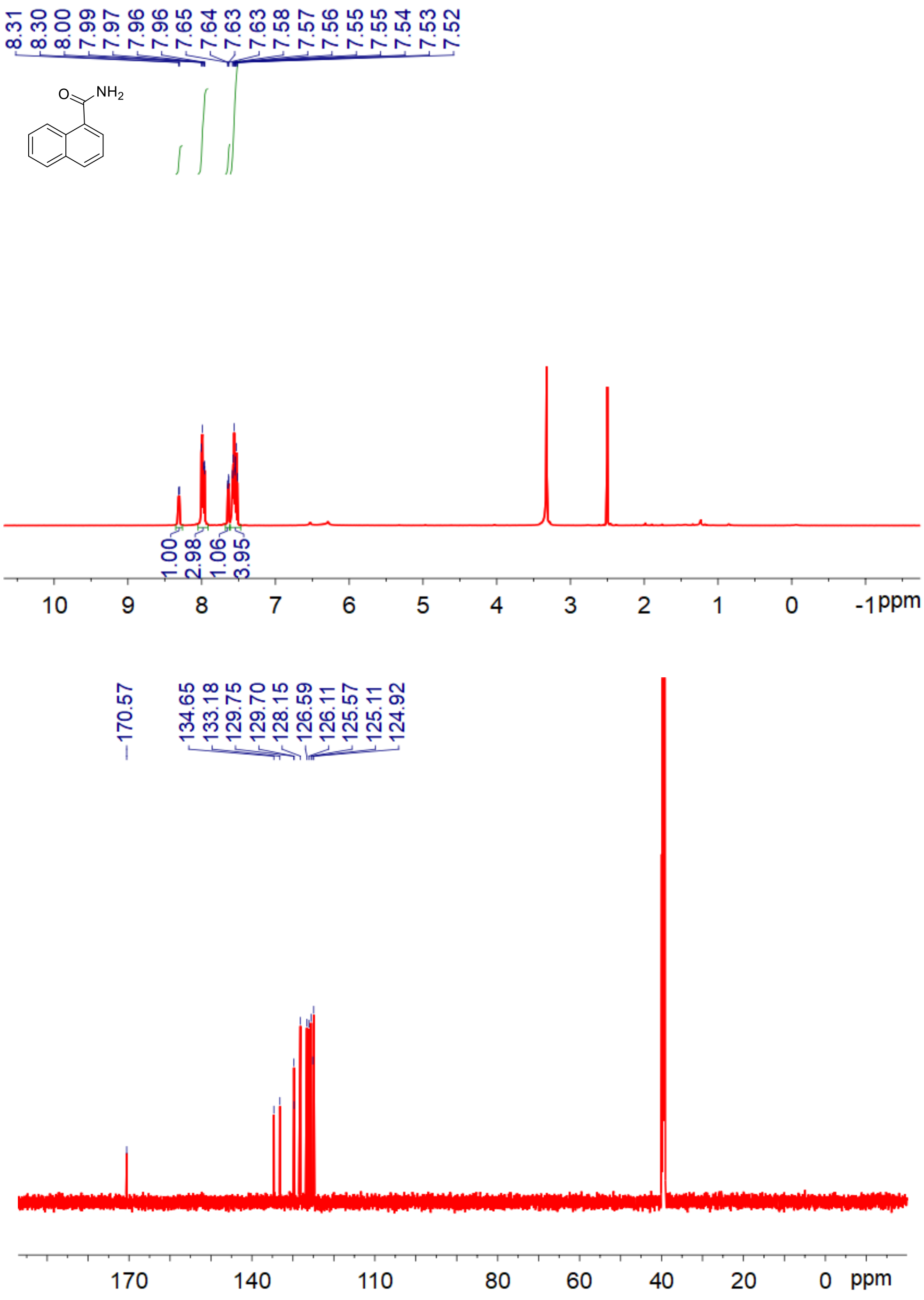

Supplementary Figure 58. ^{1}H NMR and ^{13}C NMR spectrum of 1-naphthalenecarboxamide (**30**).

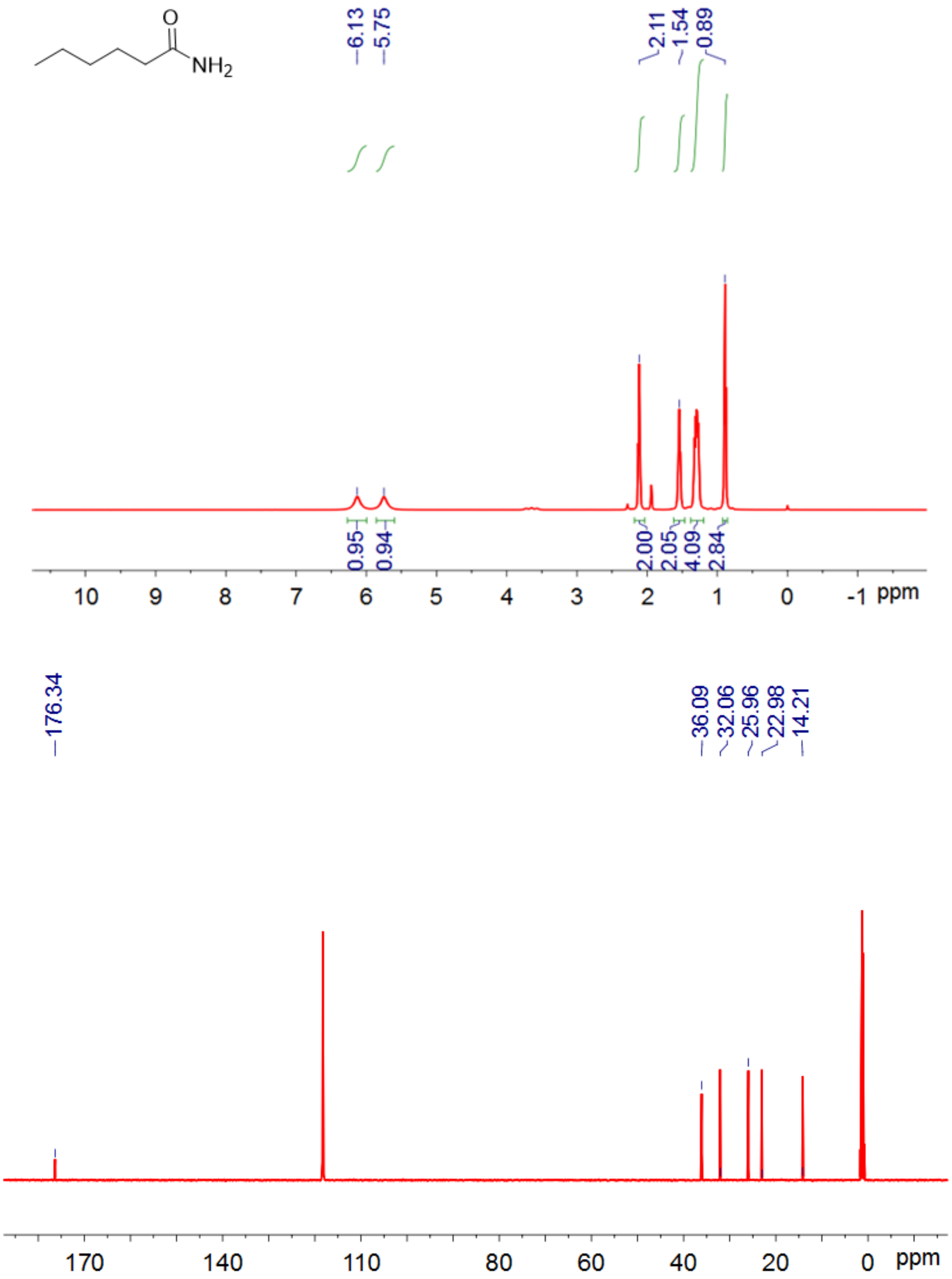

Supplementary Figure 59. ^{1}H NMR and ^{13}C NMR spectrum of formamide caproate (**31**).

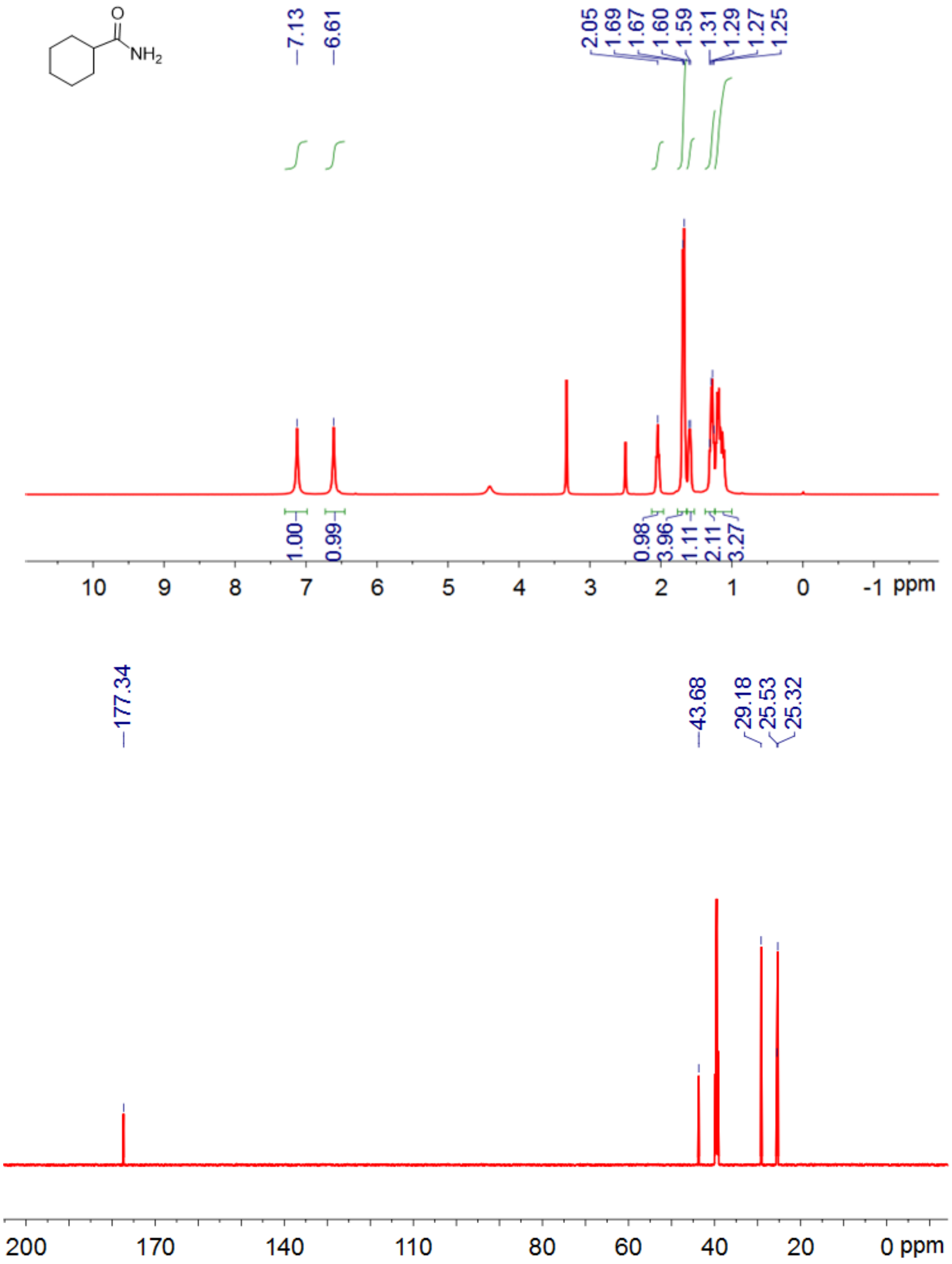

Supplementary Figure 60. ^{1}H NMR and ^{13}C NMR spectrum of 1-cyclohexylformamide (**32**).

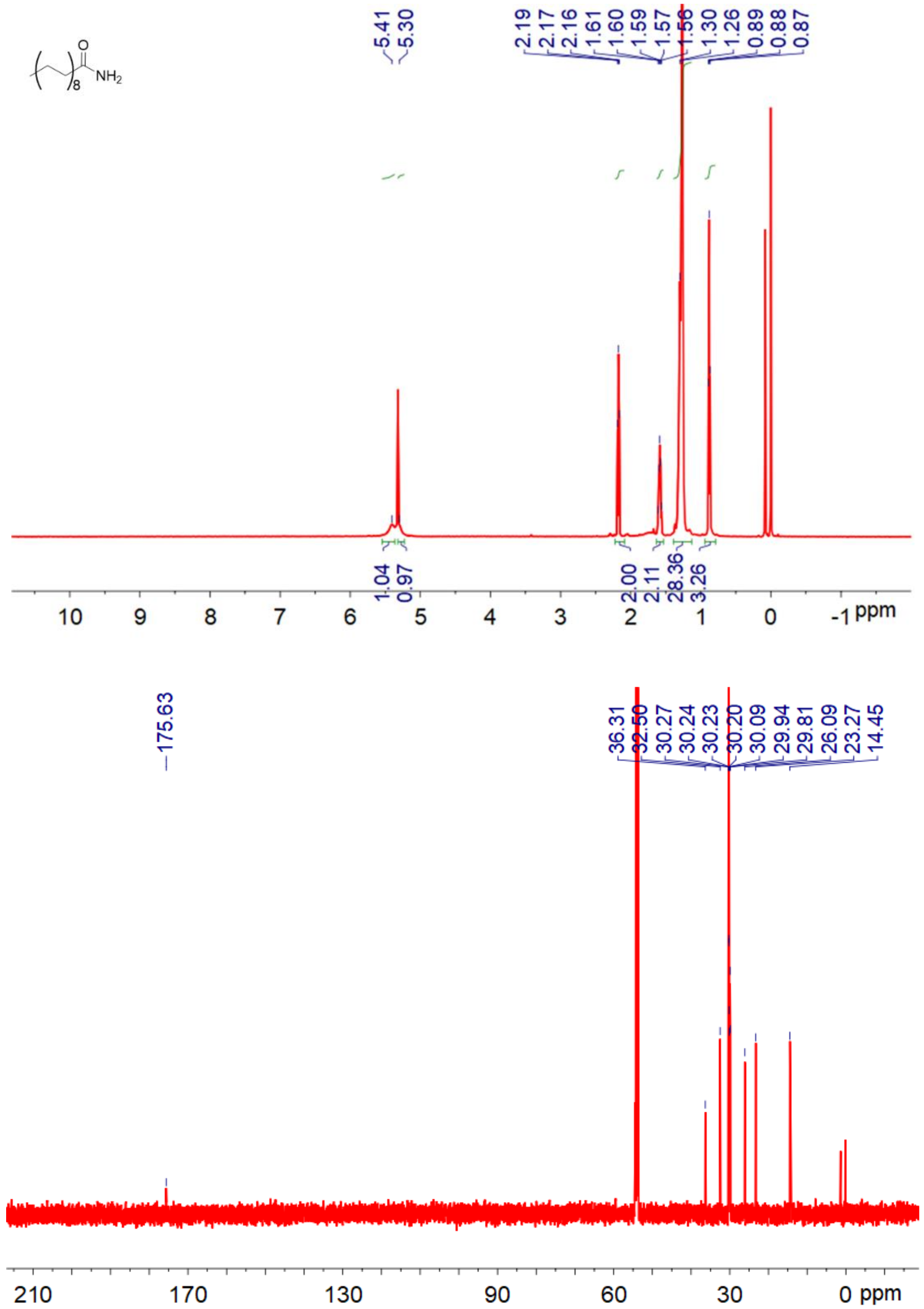

Supplementary Figure 61. ^{1}H NMR and ^{13}C NMR spectrum of octadecanamide (**33**).

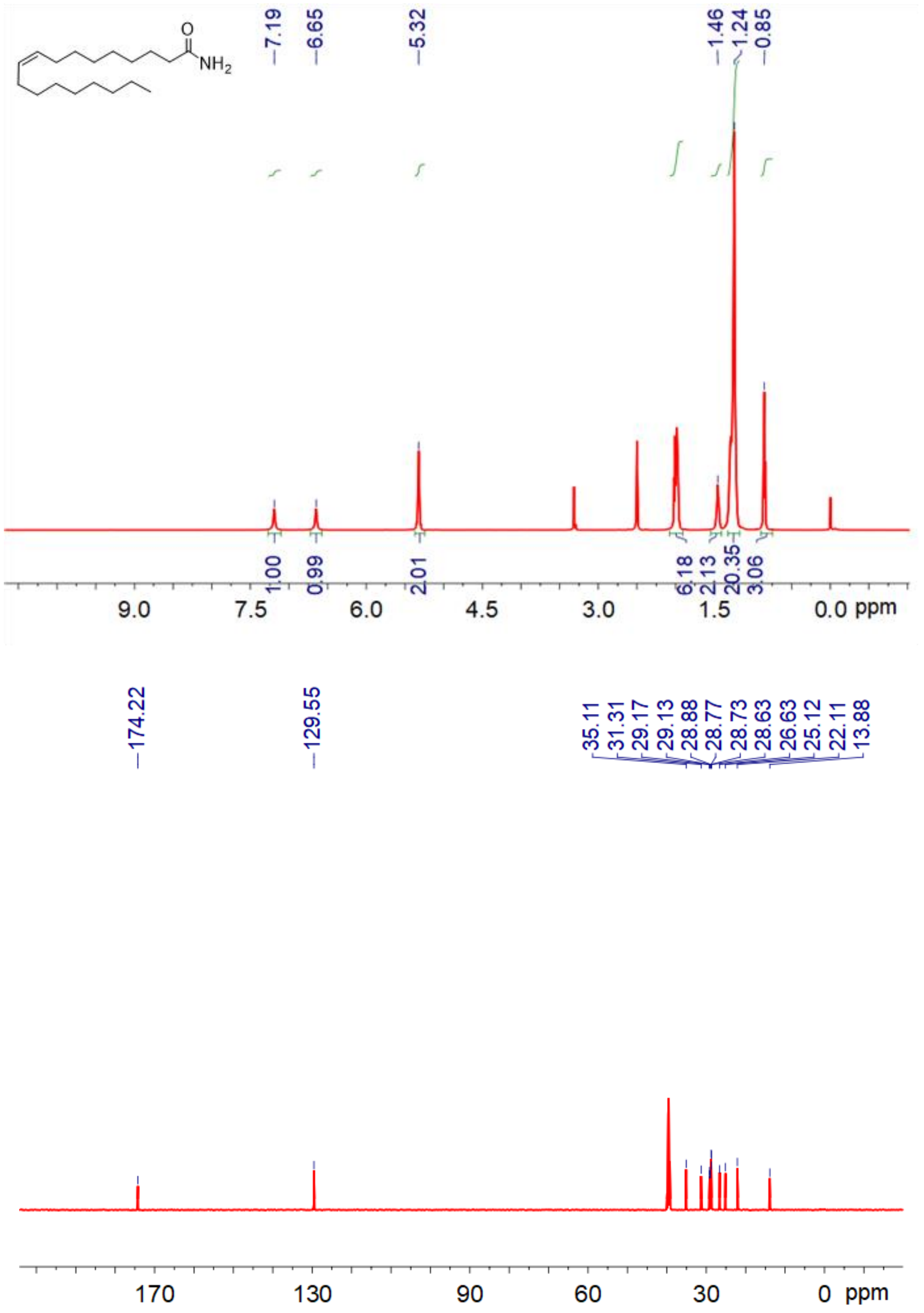

Supplementary Figure 62. ^{1}H NMR and ^{13}C NMR spectrum of oleamide (**34**).

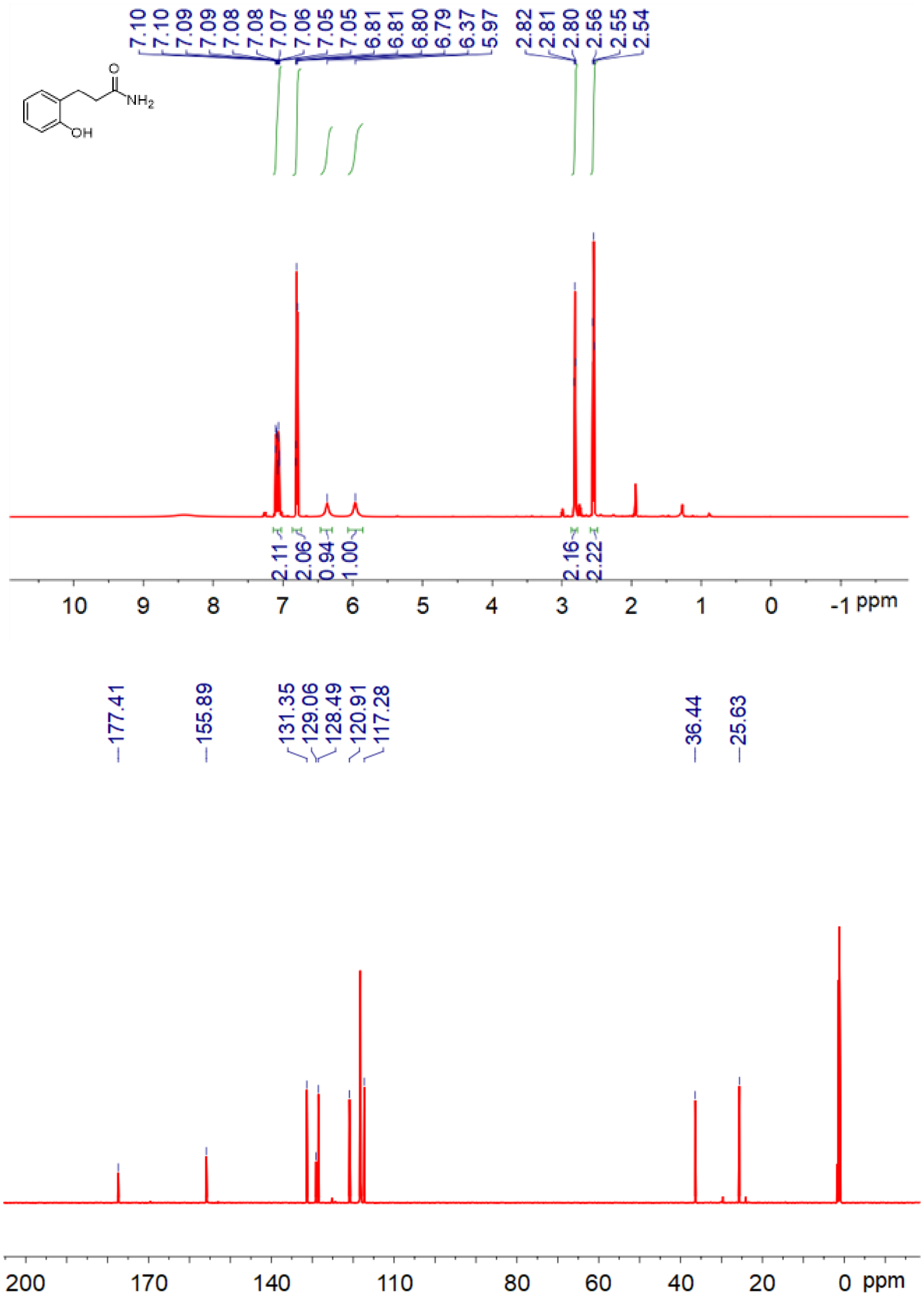

Supplementary Figure 63. ^{1}H NMR and ^{13}C NMR spectrum of 2-hydroxybenzenepropanamide (**35**).

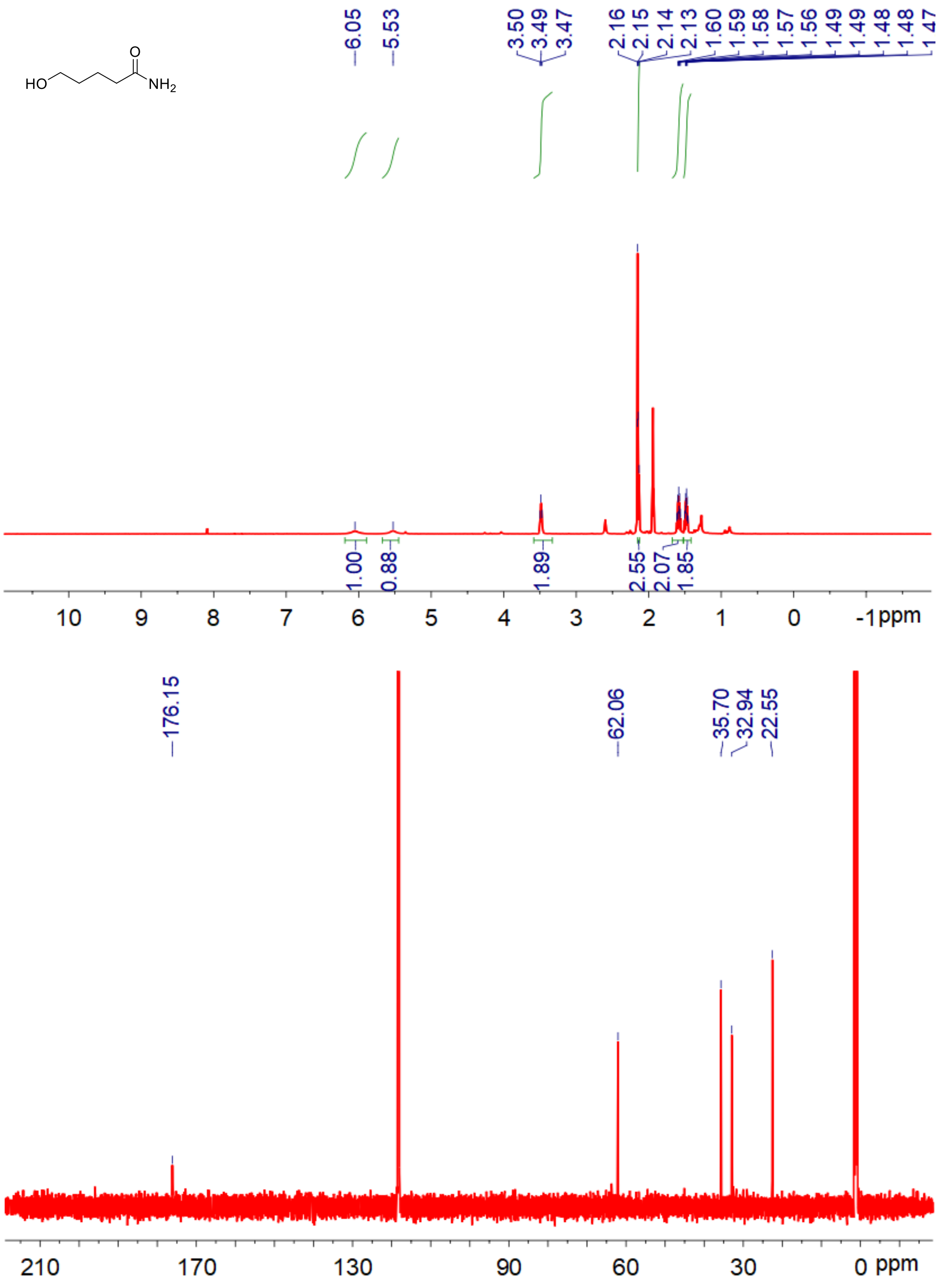

Supplementary Figure 64. ^{1}H NMR and ^{13}C NMR spectrum of 5-hydroxypentanamide (**36**).

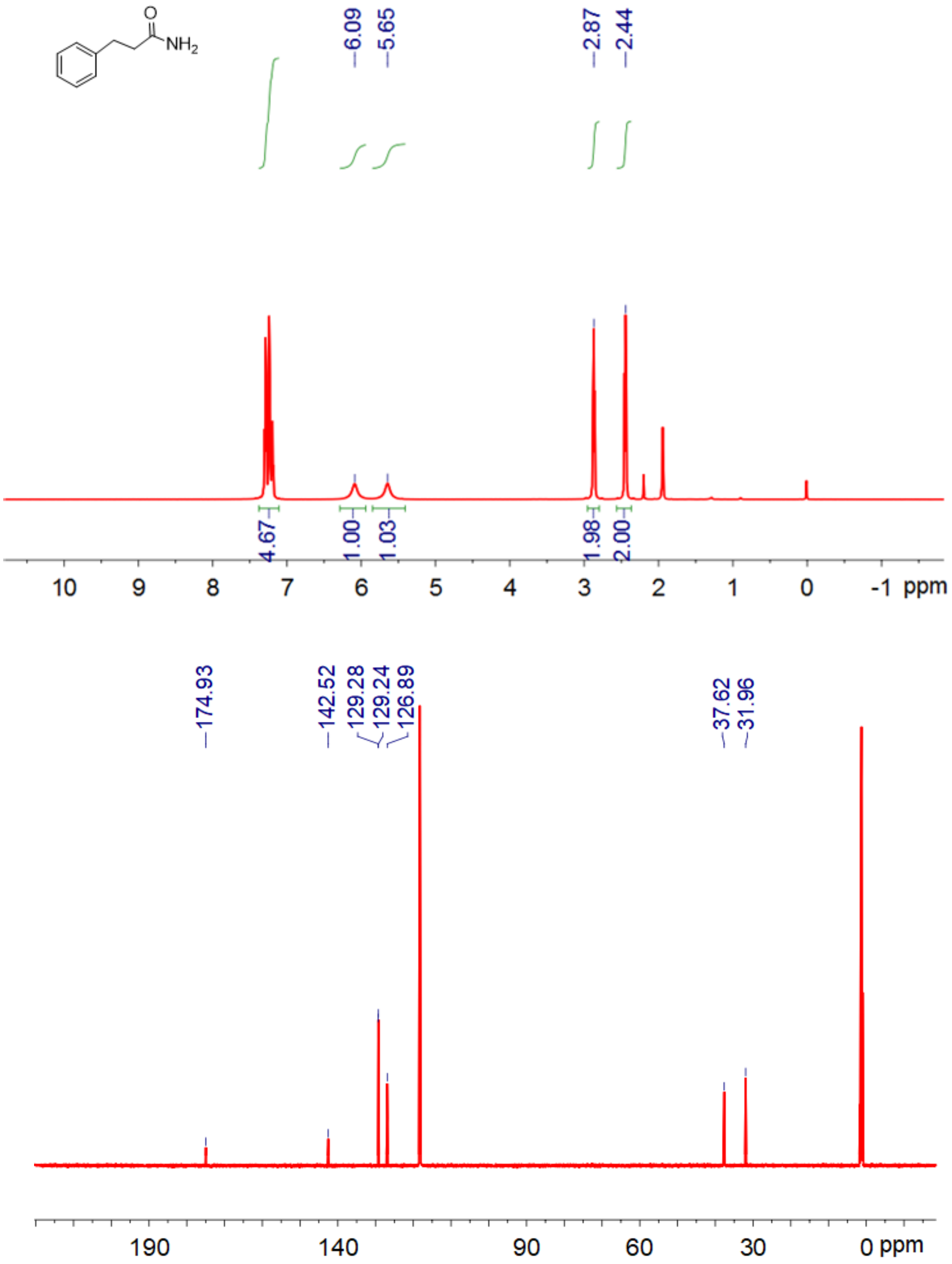

Supplementary Figure 65. ^{1}H NMR and ^{13}C NMR spectrum of phenylpropanamide (**37**).

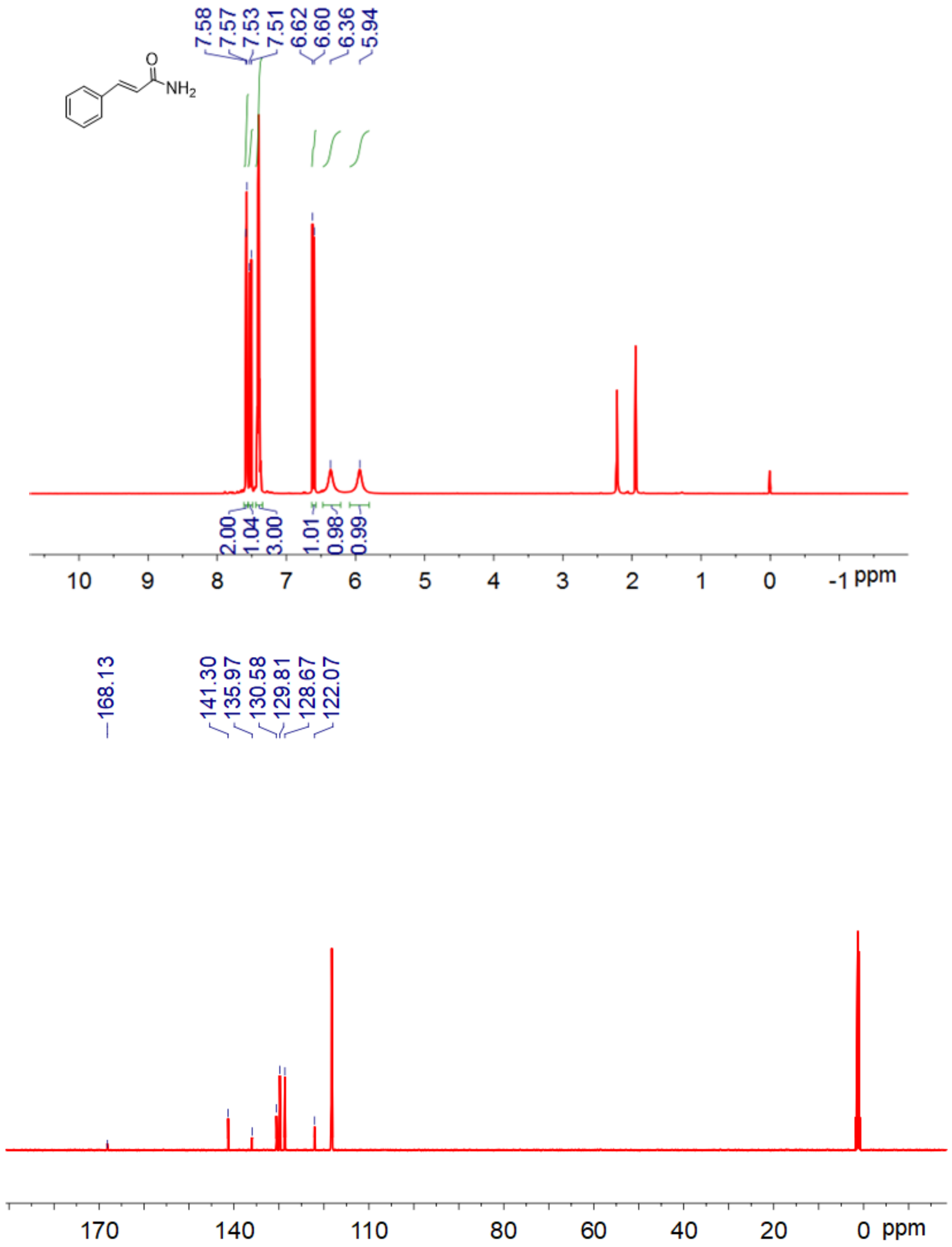

Supplementary Figure 66. ^{1}H NMR and ^{13}C NMR spectrum of cinnamamide (**38, 39**).

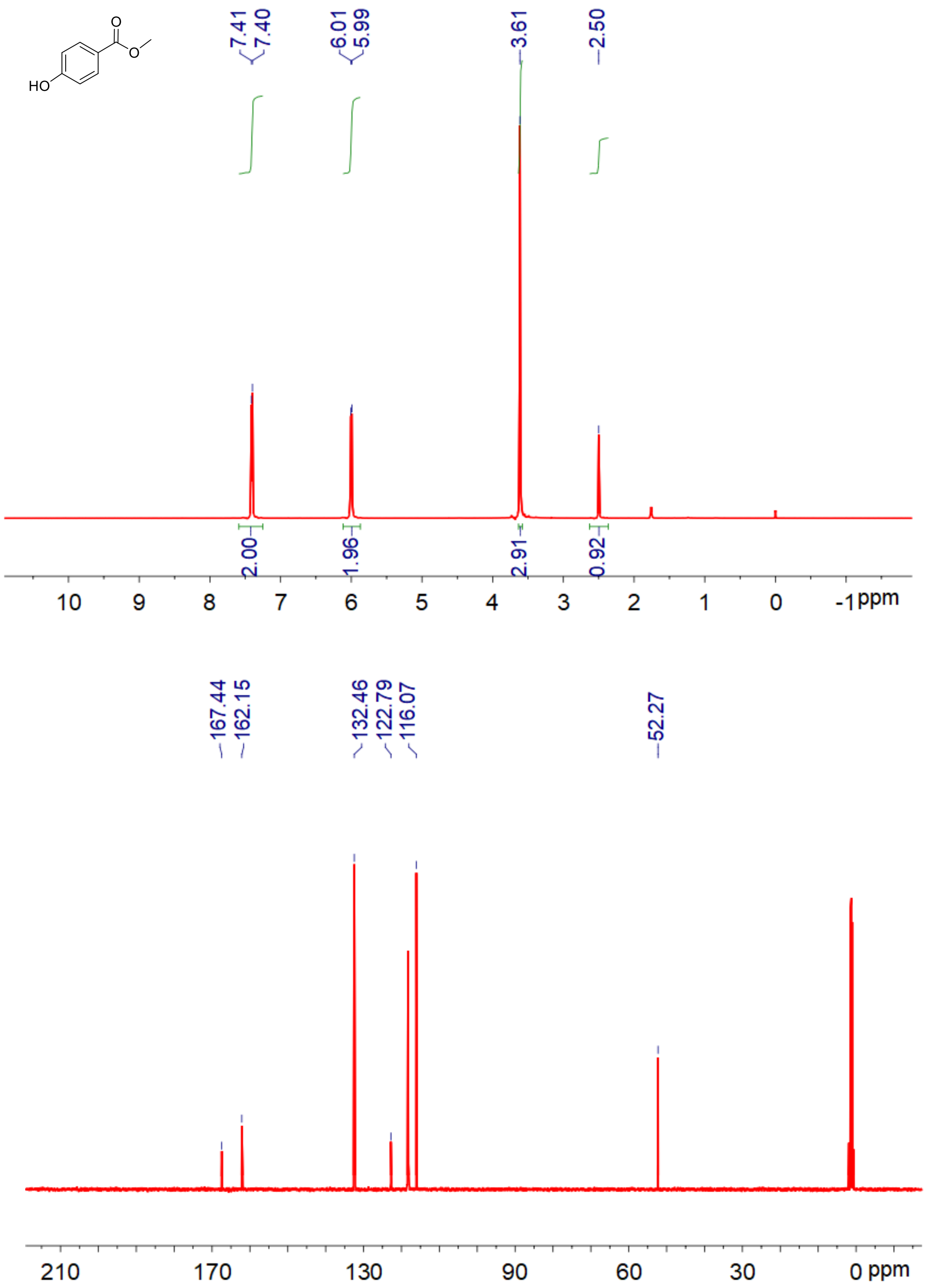

Supplementary Figure 67. ^{1}H NMR and ^{13}C NMR spectrum of 4-hydroxymethyl benzoate (**40**).

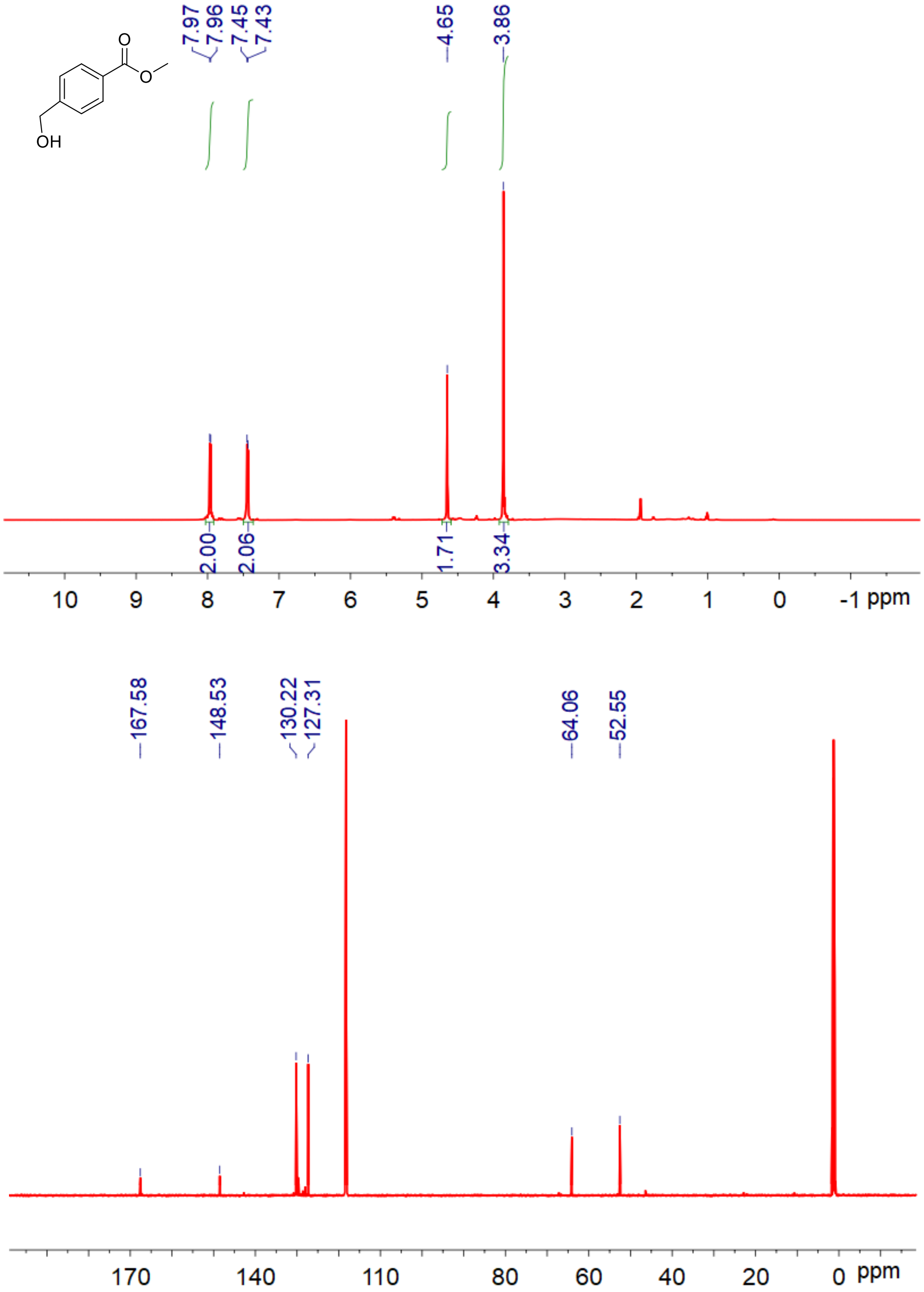

Supplementary Figure 68. ^{1}H NMR and ^{13}C NMR spectrum of methyl 4-(hydroxymethyl)benzoate (**41**).

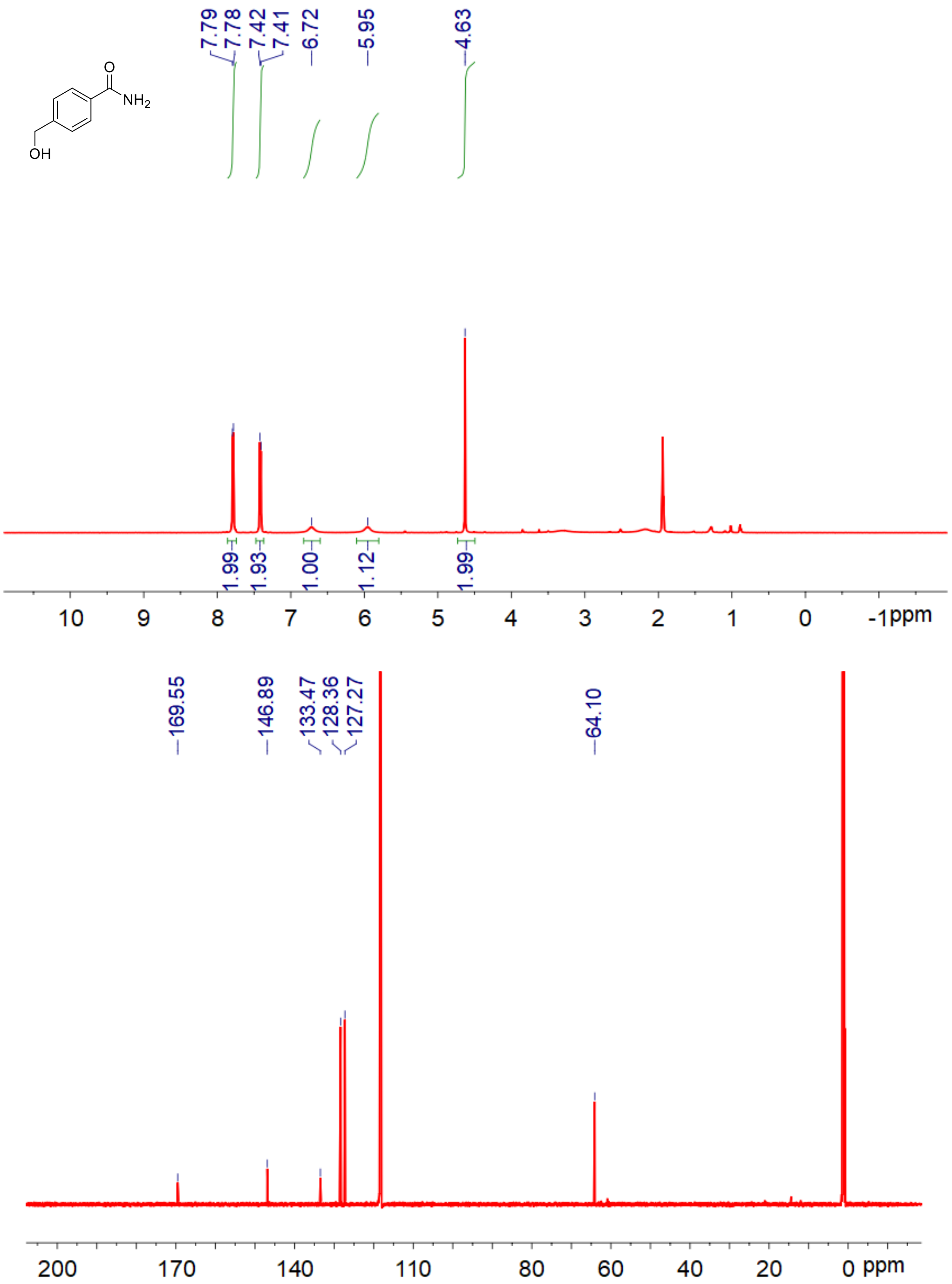

Supplementary Figure 69. ^{1}H NMR and ^{13}C NMR spectrum of 4-(hydroxymethyl)benzamide (**42**).

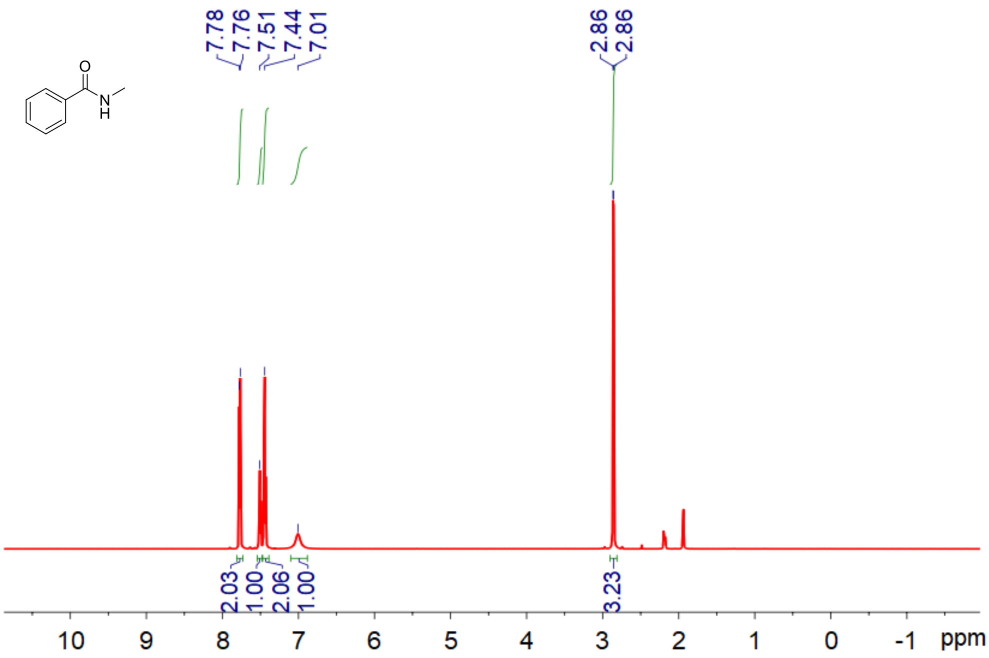

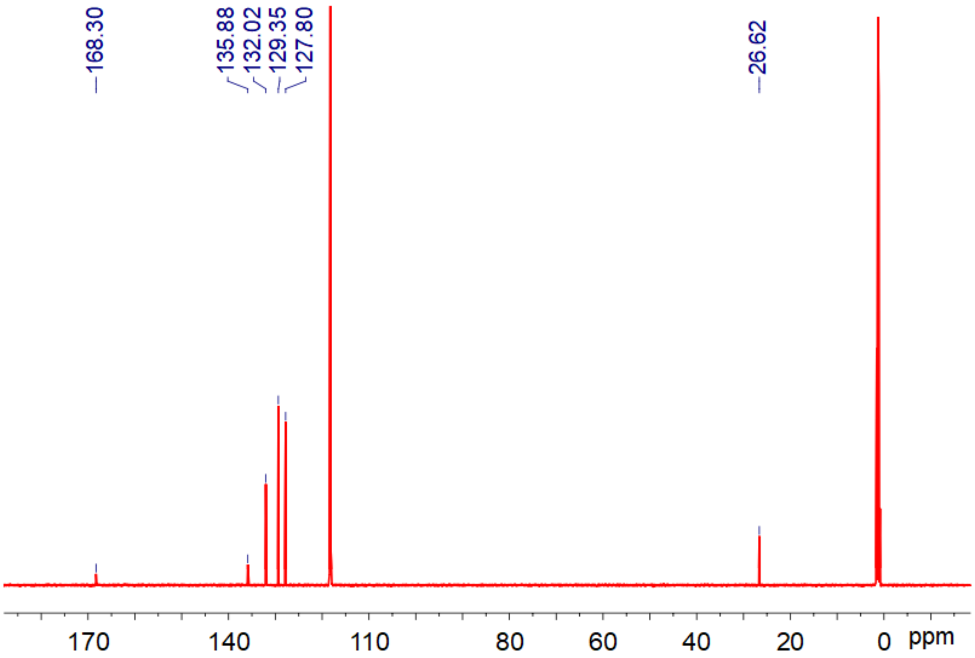

Supplementary Figure 70. ^{1}H NMR and ^{13}C NMR spectrum of *N*-methylbenzamide (**43**).

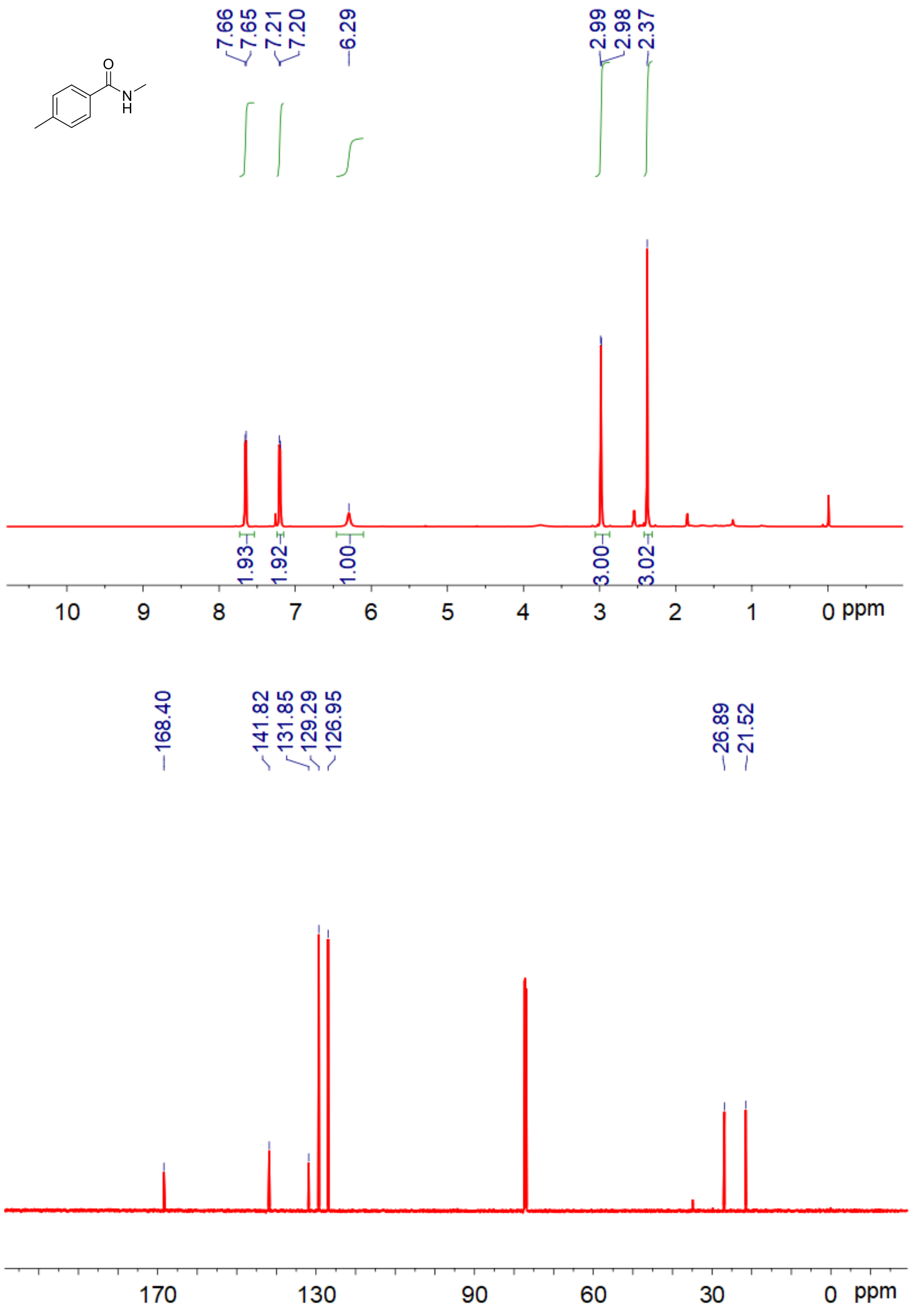

Supplementary Figure 71. ^{1}H NMR and ^{13}C NMR spectrum of *N*-methyl-p-toluamide (**44**).

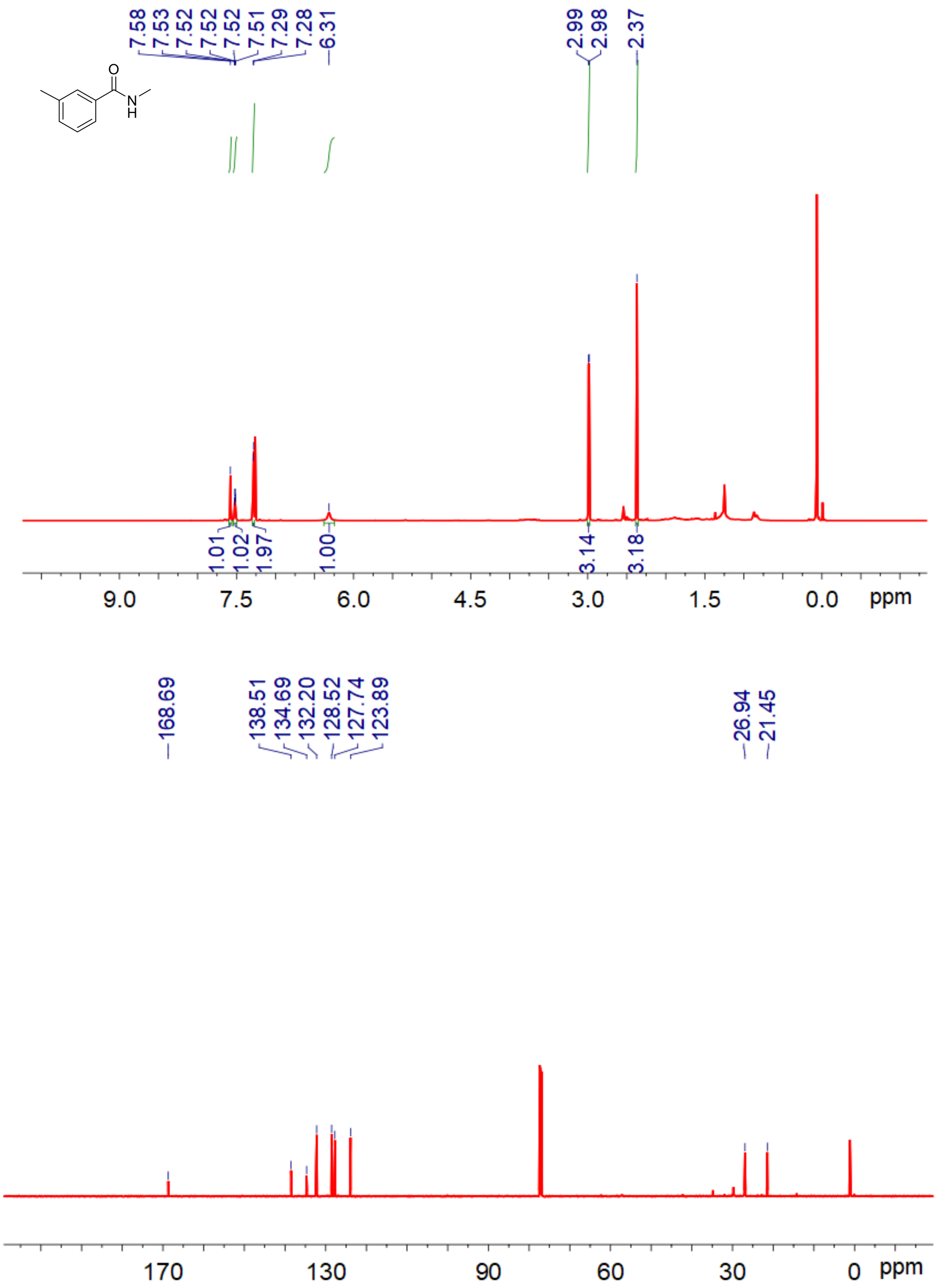

Supplementary Figure 72. ^{1}H NMR and ^{13}C NMR spectrum of *N*-methyl-3-methylbenzamide (**45**).

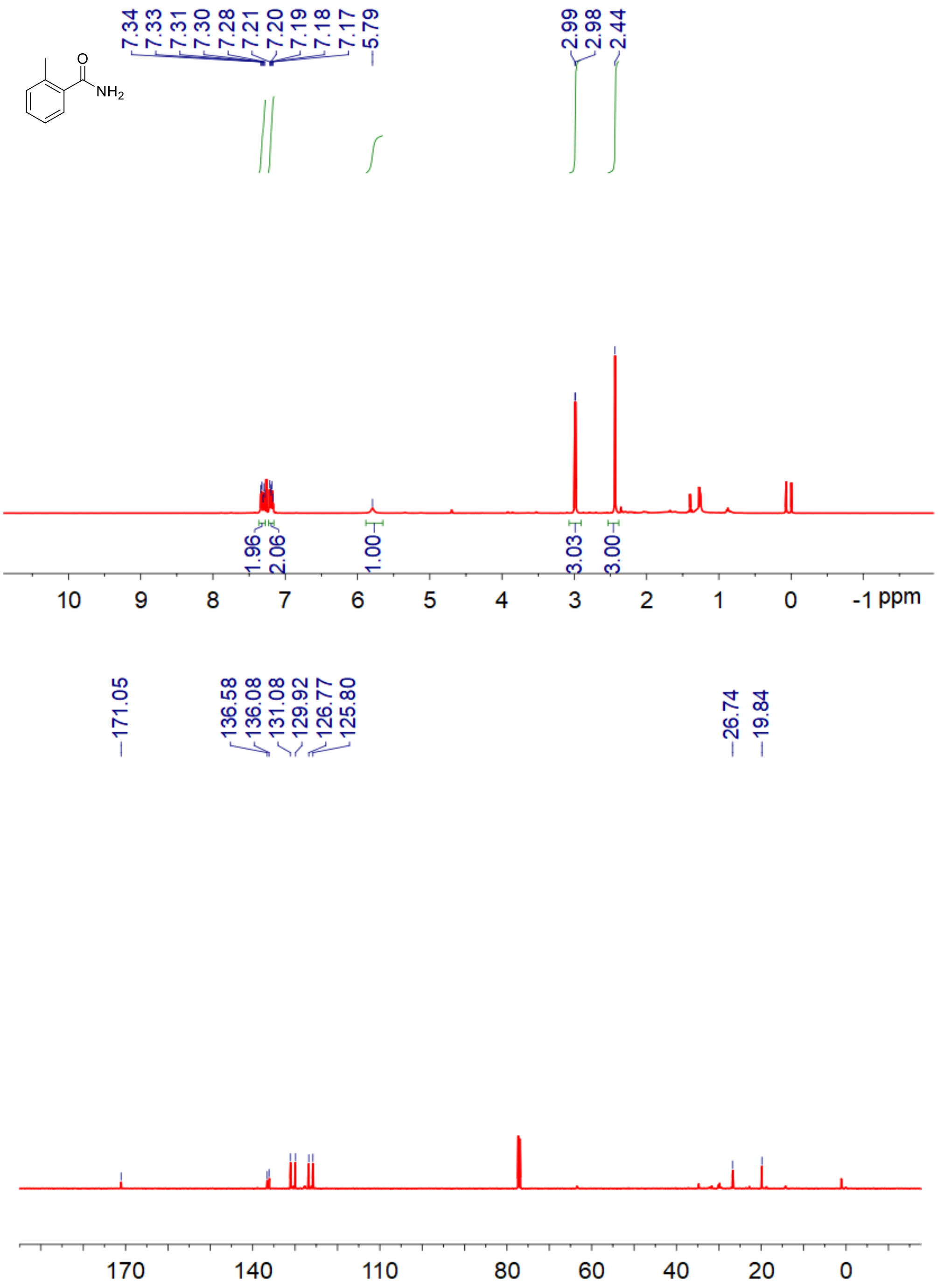

Supplementary Figure 73. ^{1}H NMR and ^{13}C NMR spectrum of *N*-methyl-2-methylbenzamide (**46**).

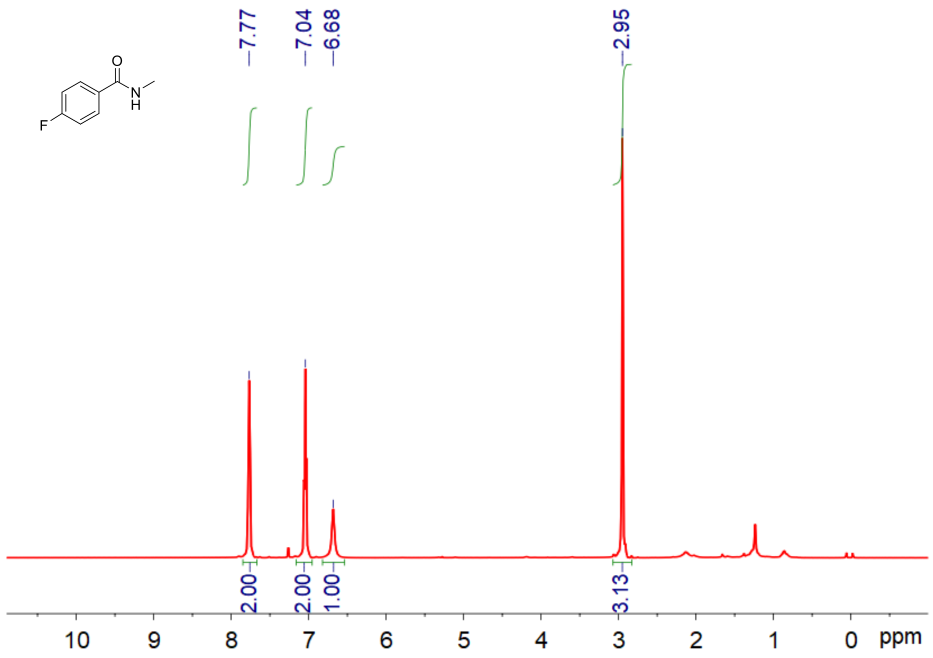

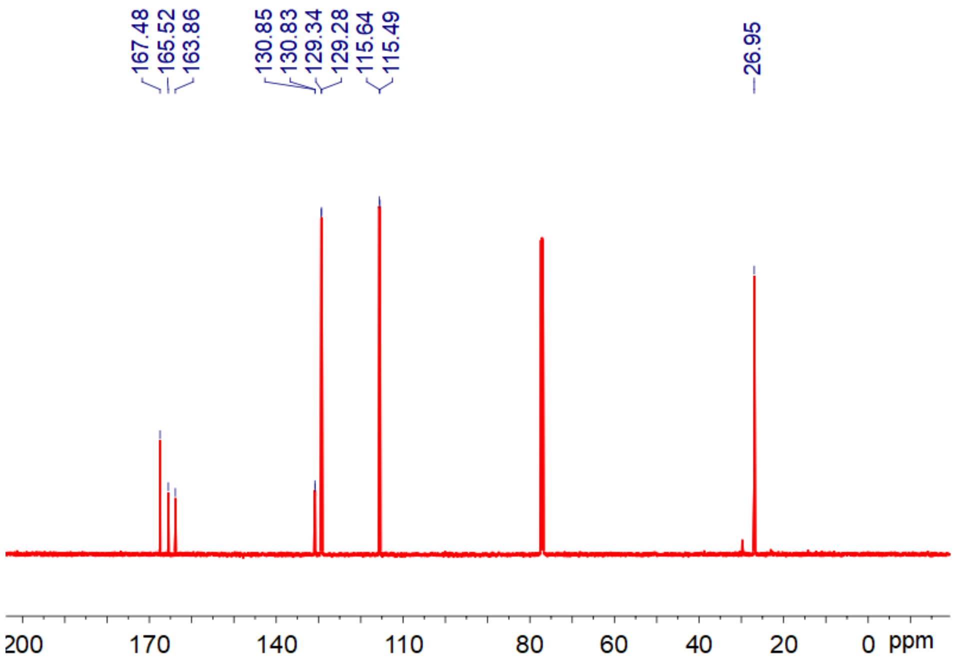

Supplementary Figure 74. ^{1}H NMR and ^{13}C NMR spectrum of *N*-methyl-4-fluorobenzamide (**47**).

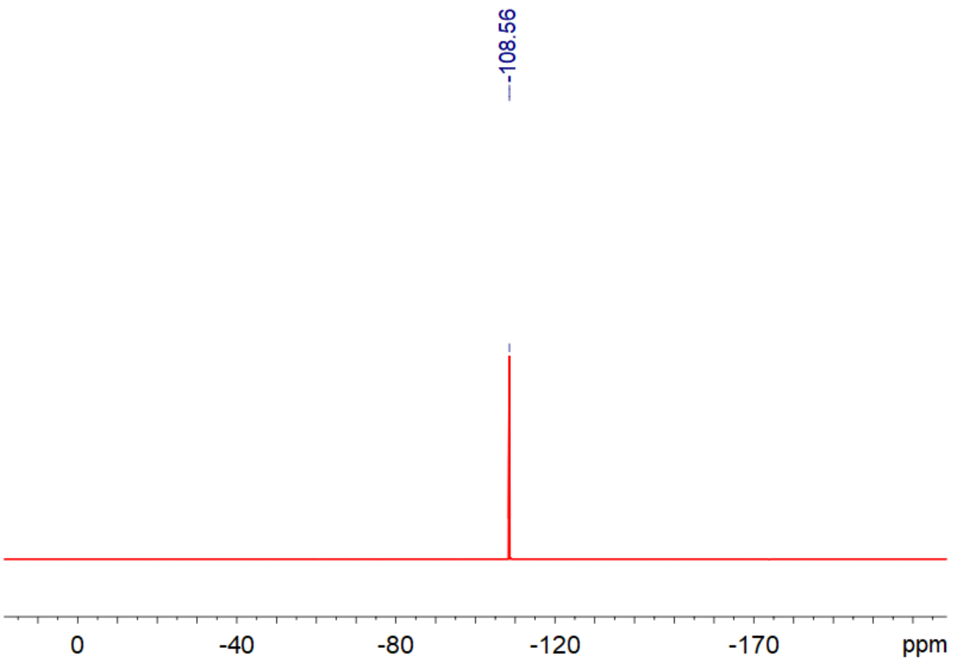

Supplementary Figure 75. ^{19}F NMR spectrum of *N*-methyl-4-fluorobenzamide (**47**).

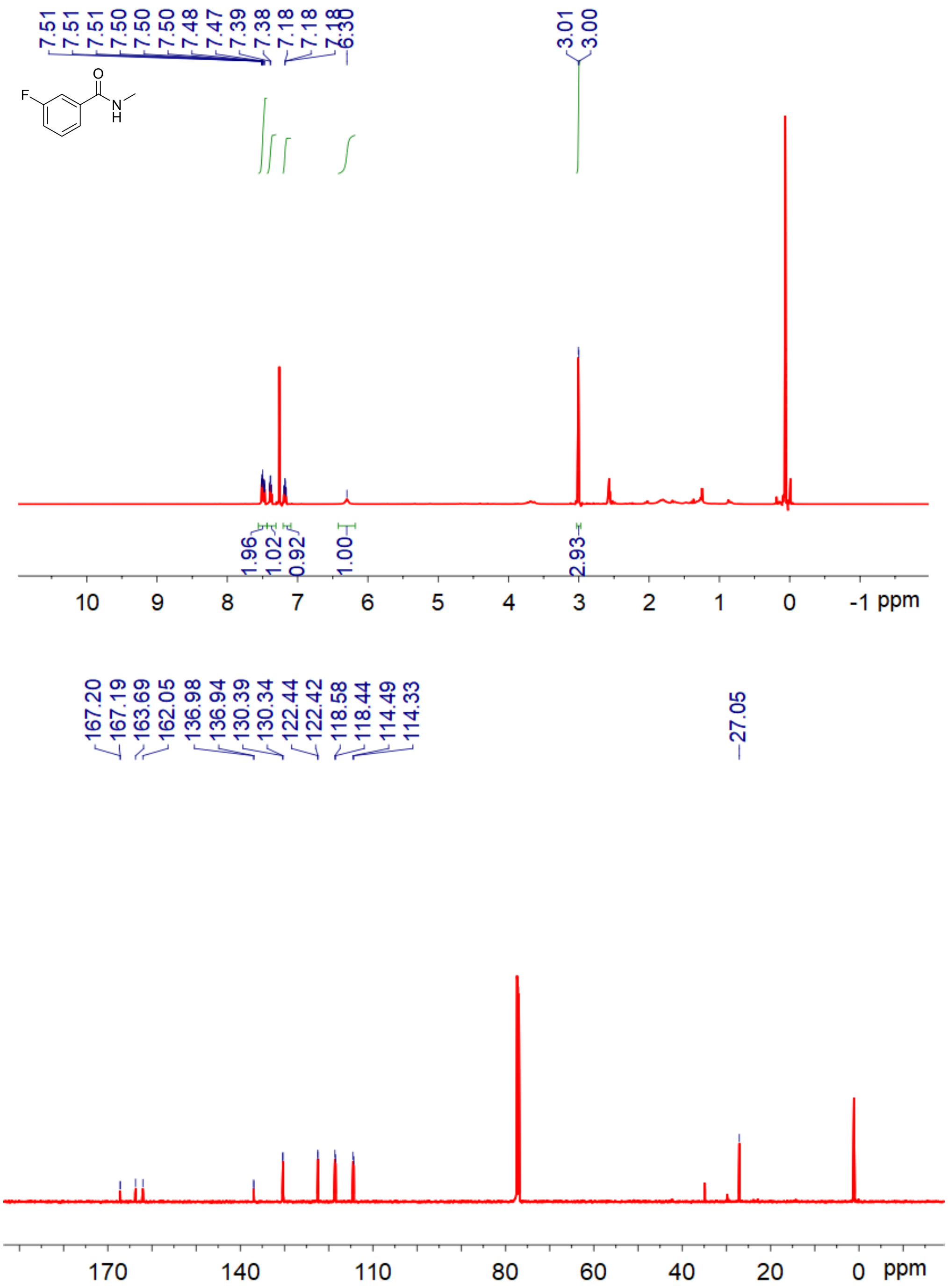

Supplementary Figure 76. ^{1}H NMR and ^{13}C NMR spectrum of 3-fluoro-*N*-methylbenzamide (**48**).

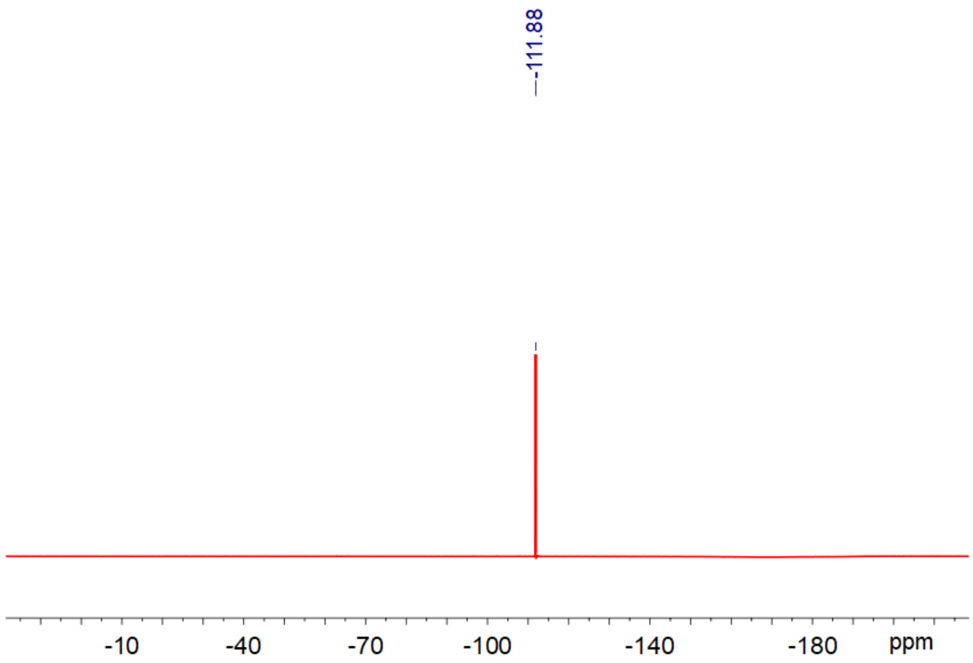

Supplementary Figure 77. ^{19}F NMR spectrum of 3-fluoro-*N*-methylbenzamide (**48**).

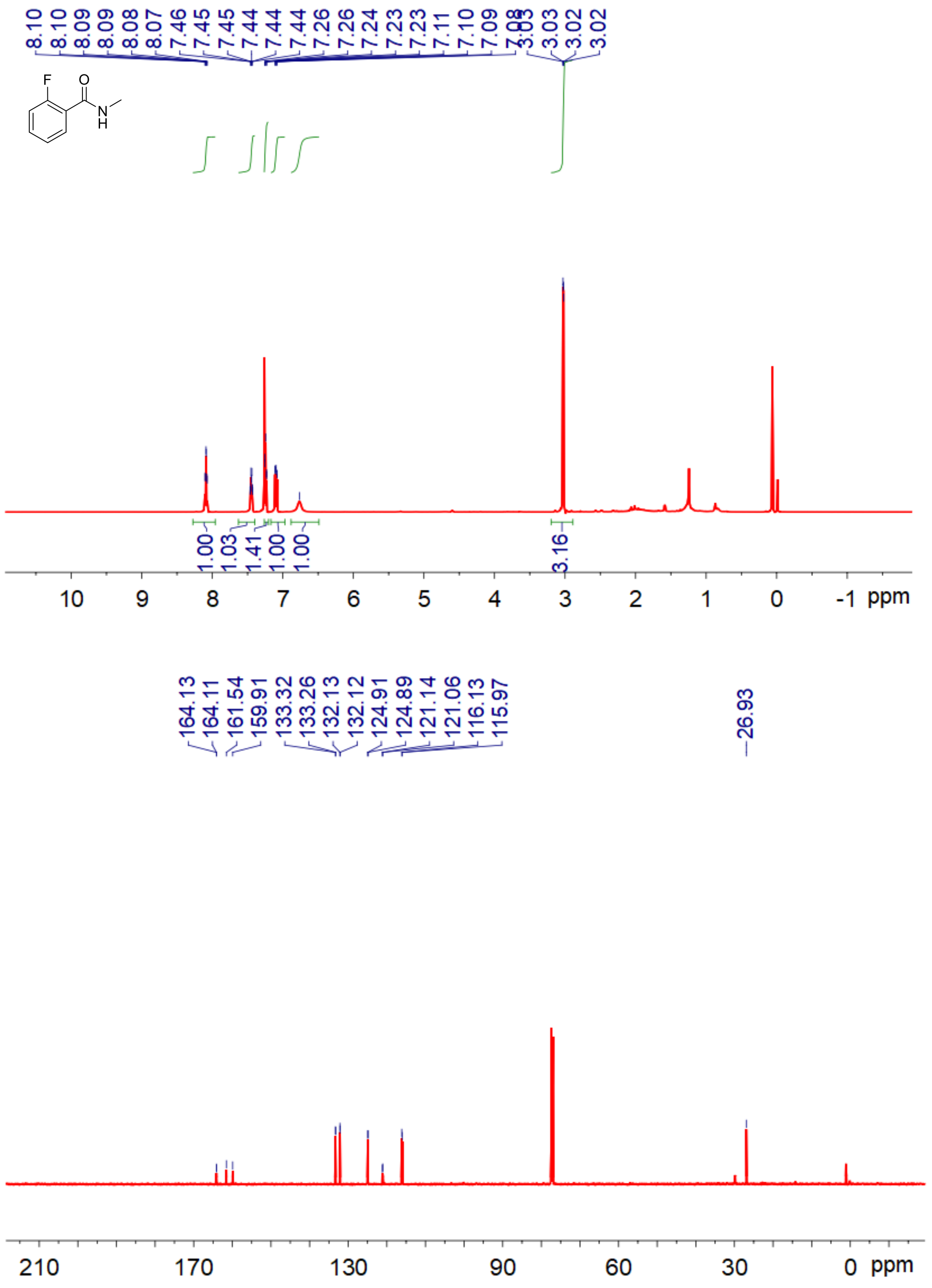

Supplementary Figure 78. ^{1}H NMR and ^{13}C NMR spectrum of 2-fluoro-*N*-methylbenzamide (**49**).

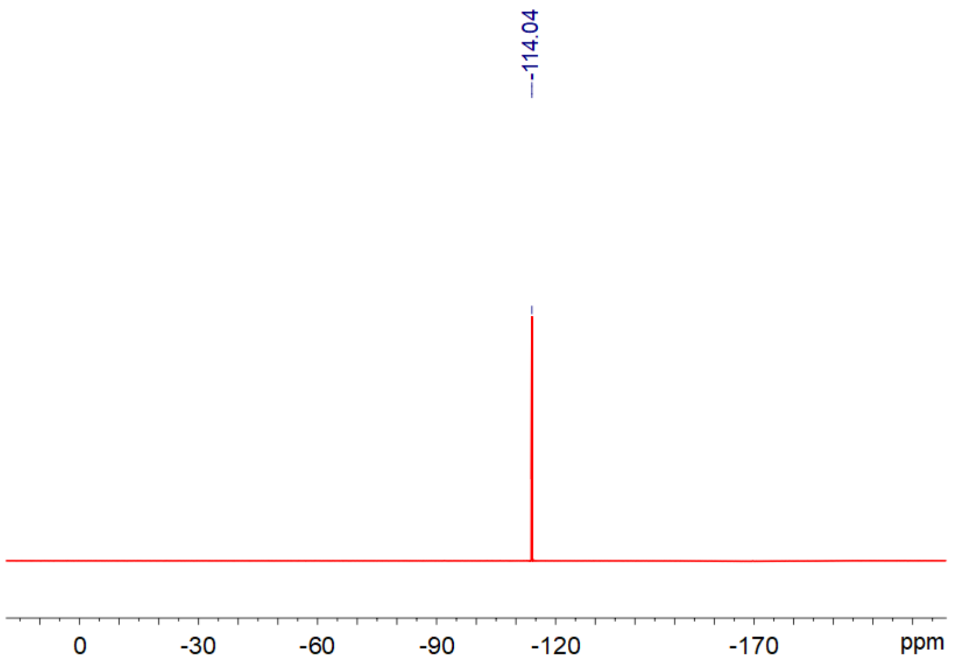

Supplementary Figure 79. ^{19}F NMR spectrum of 2-fluoro-*N*-methylbenzamide (**49**).

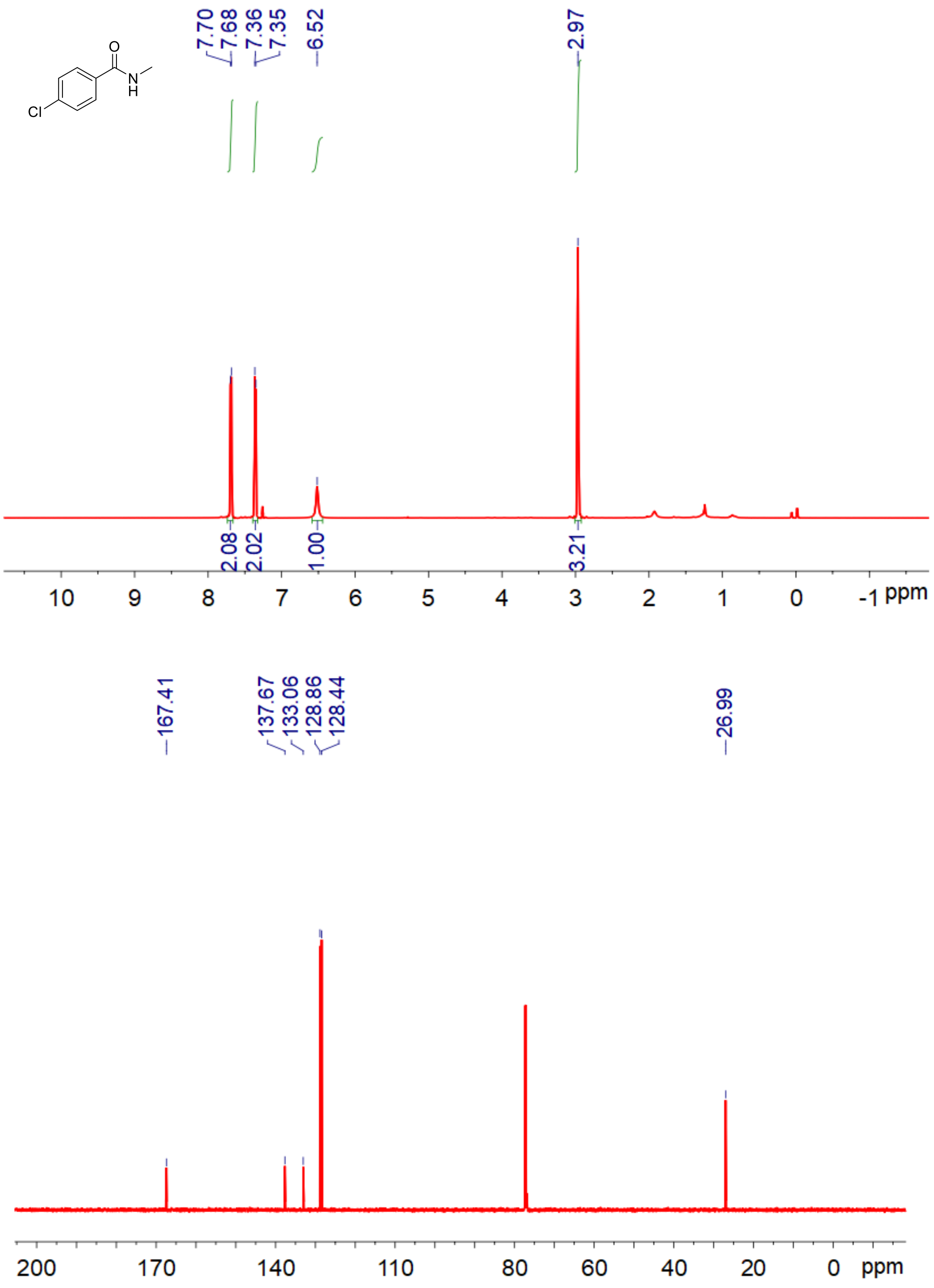

Supplementary Figure 80. ^{1}H NMR and ^{13}C NMR spectrum of 4-chloro-*N*-methylbenzamide (**50**).

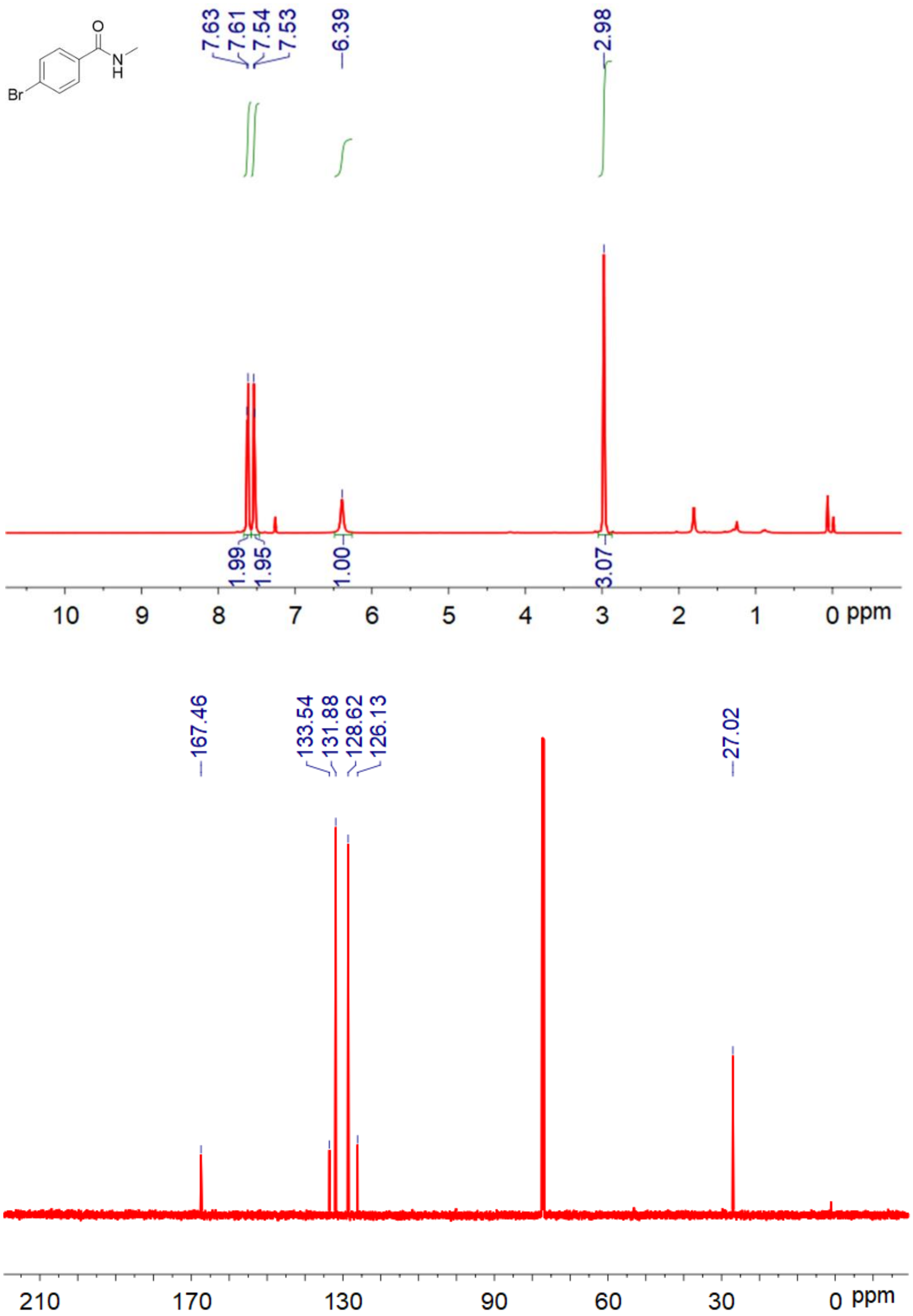

Supplementary Figure 81. ^{1}H NMR and ^{13}C NMR spectrum of 4-bromo-*N*-methylbenzamide (**51**).

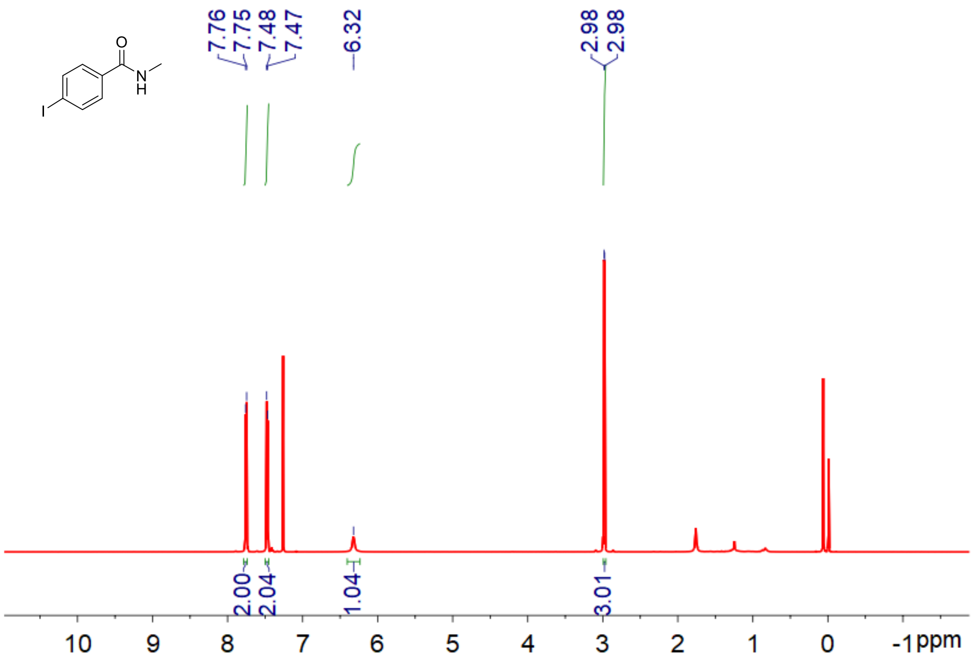

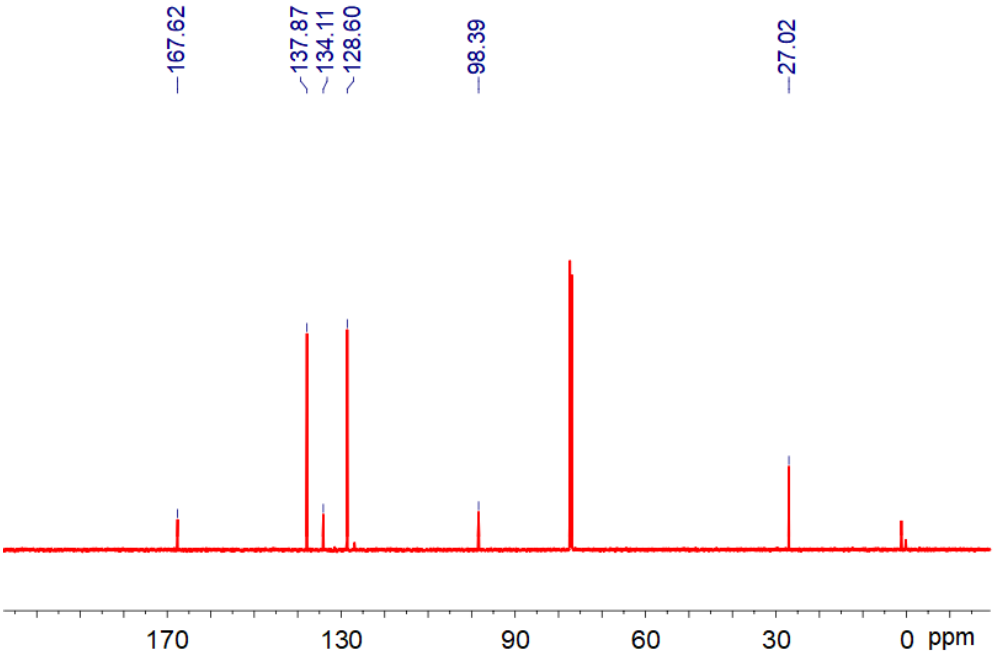

Supplementary Figure 82. ^{1}H NMR and ^{13}C NMR spectrum of 4-iodo-*N*-methylbenzamide (**52**).

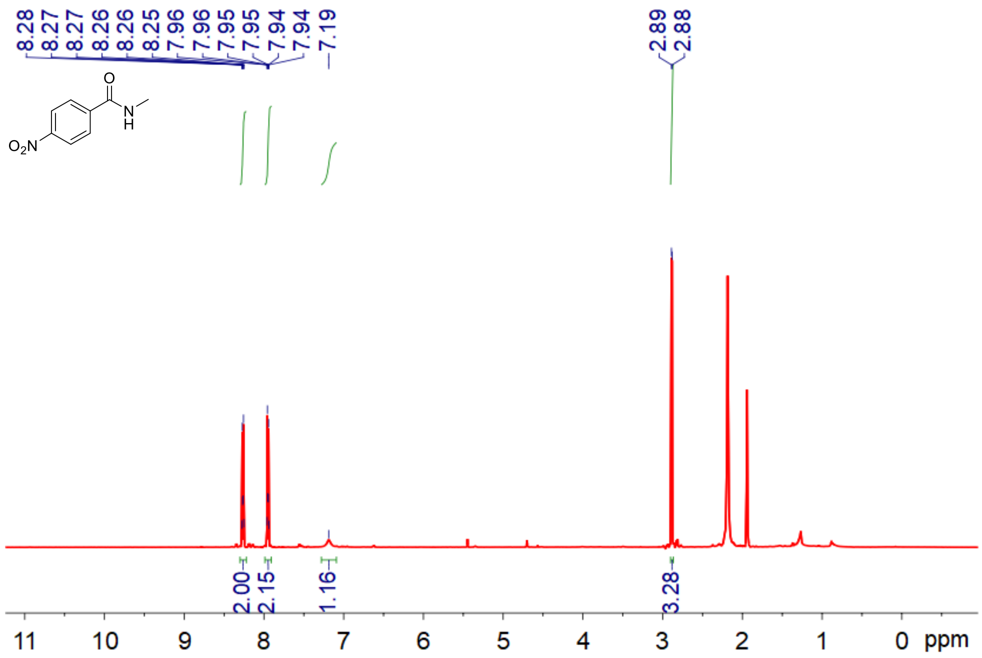

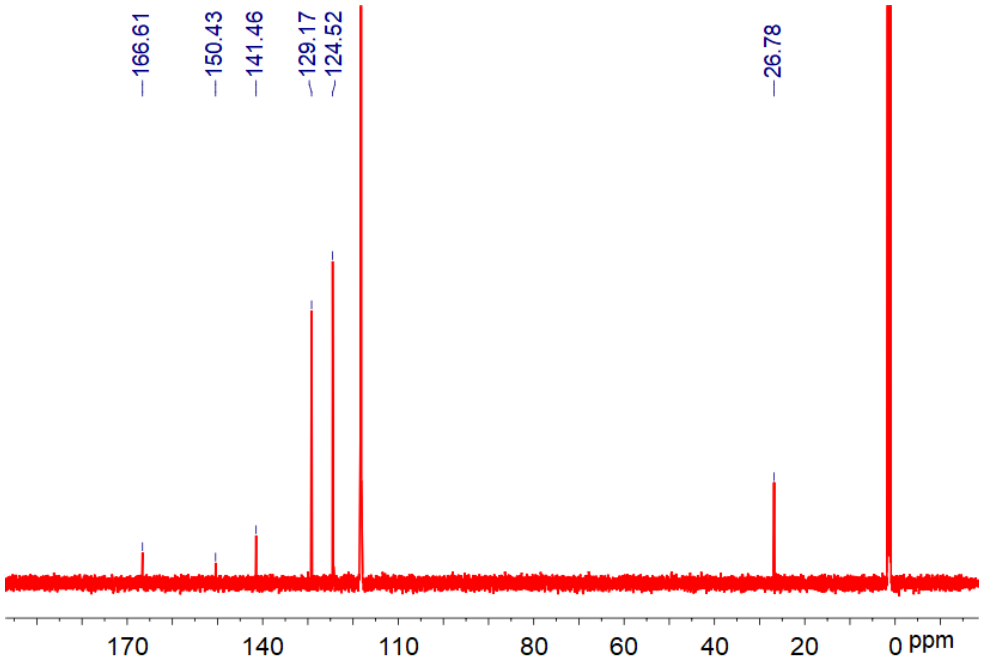

Supplementary Figure 83. ^{1}H NMR and ^{13}C NMR spectrum of *N*-methyl-4-nitrobenzamide (**53**).

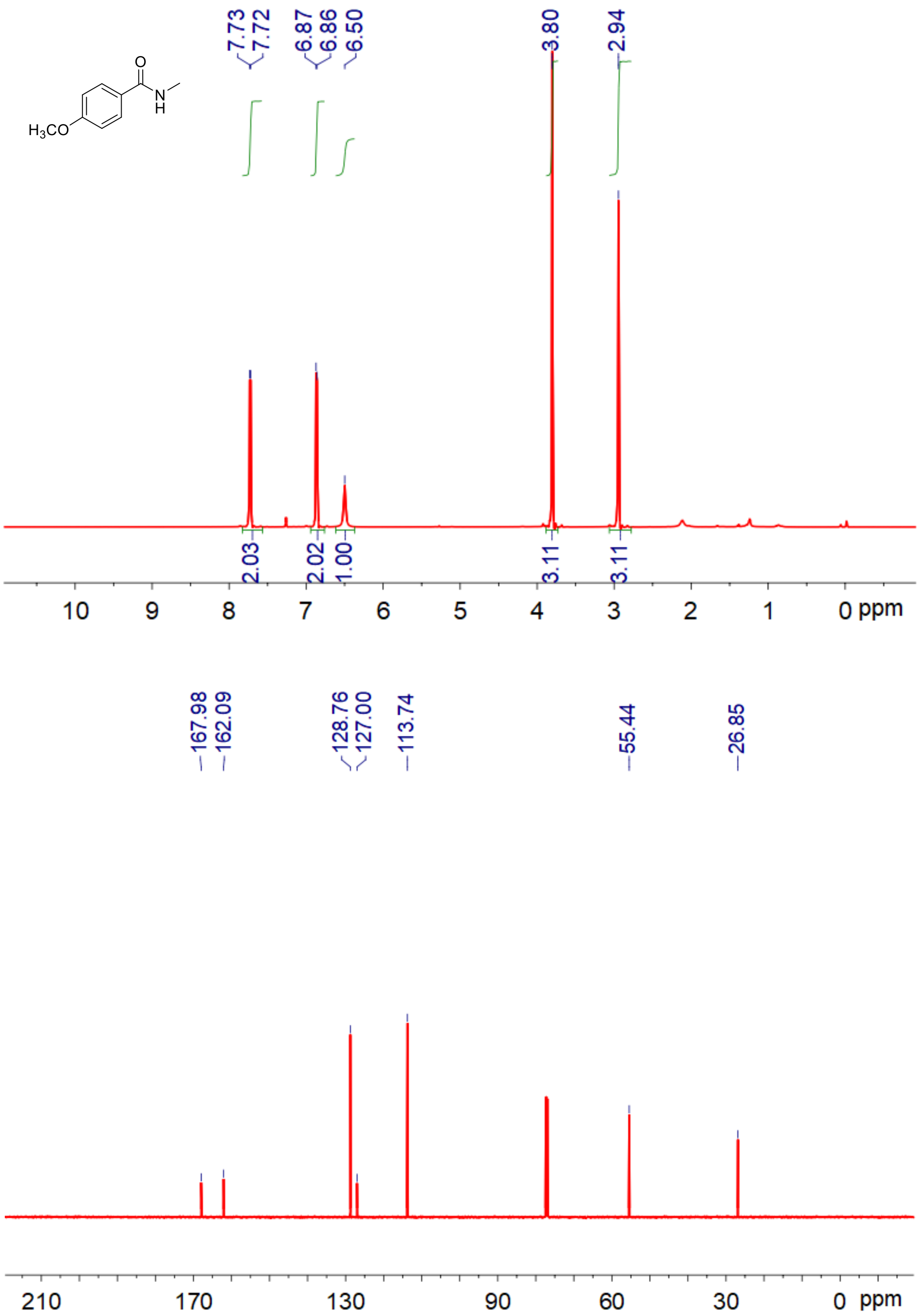

Supplementary Figure 84. ^{1}H NMR and ^{13}C NMR spectrum of 4-methoxy-*N*-methylbenzamide (**54**).

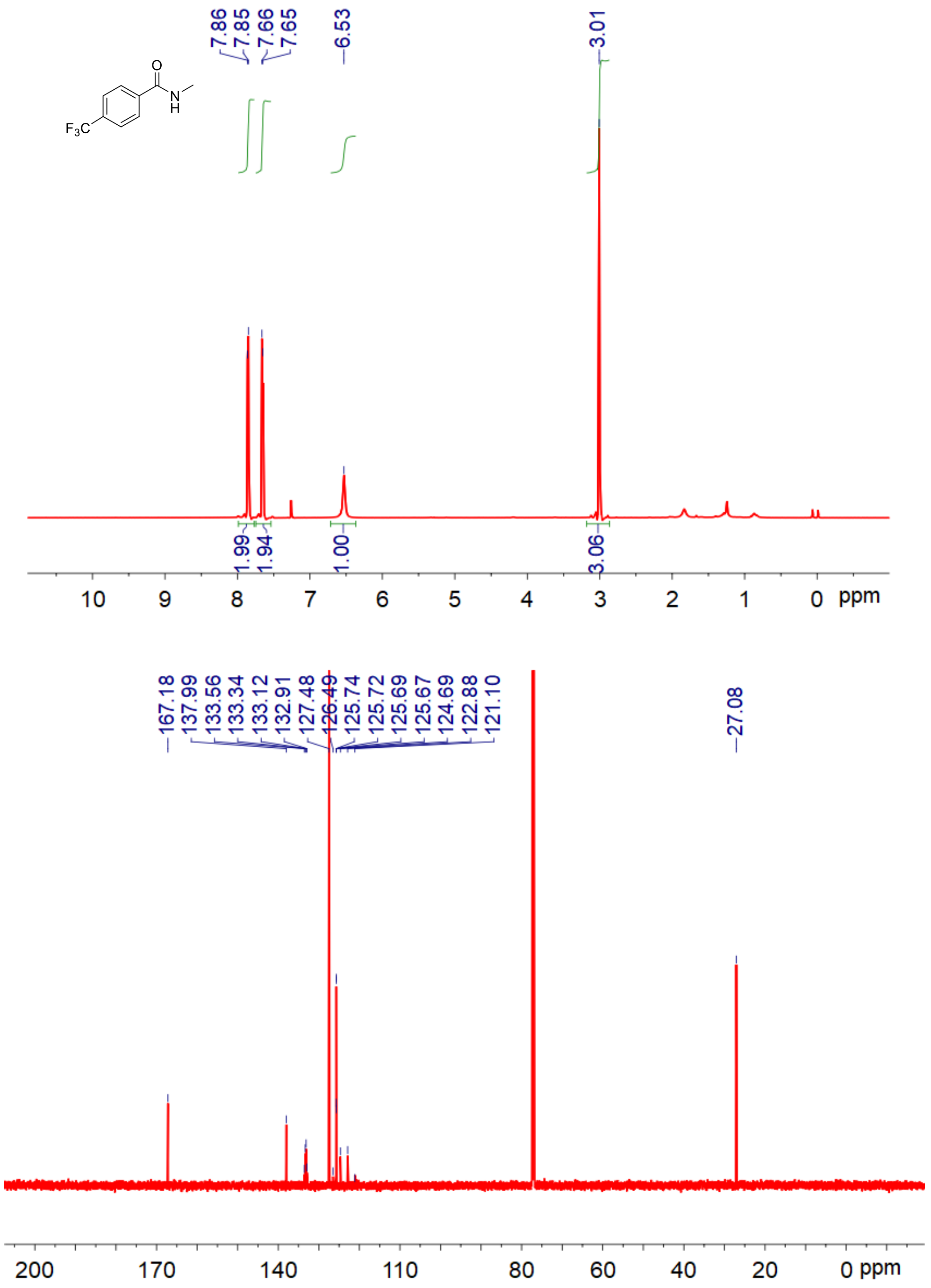

Supplementary Figure 85. ^{1}H NMR and ^{13}C NMR spectrum of *N*-methyl-4-(trifluoromethyl)benzamide (**55**).

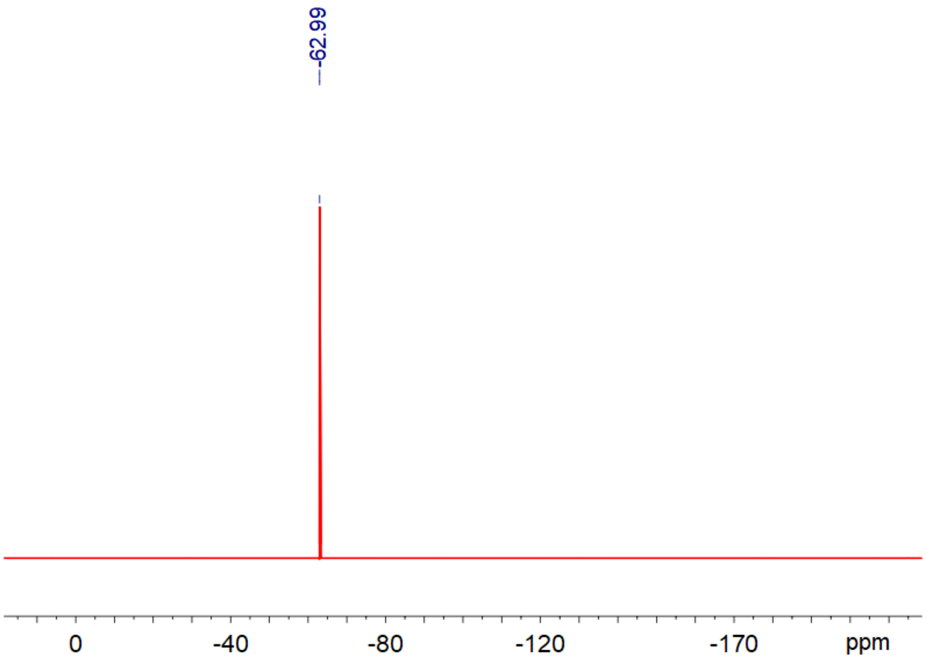

Supplementary Figure 86. ^{19}F NMR spectrum of *N*-methyl-4-(trifluoromethyl)benzamide (**55**).

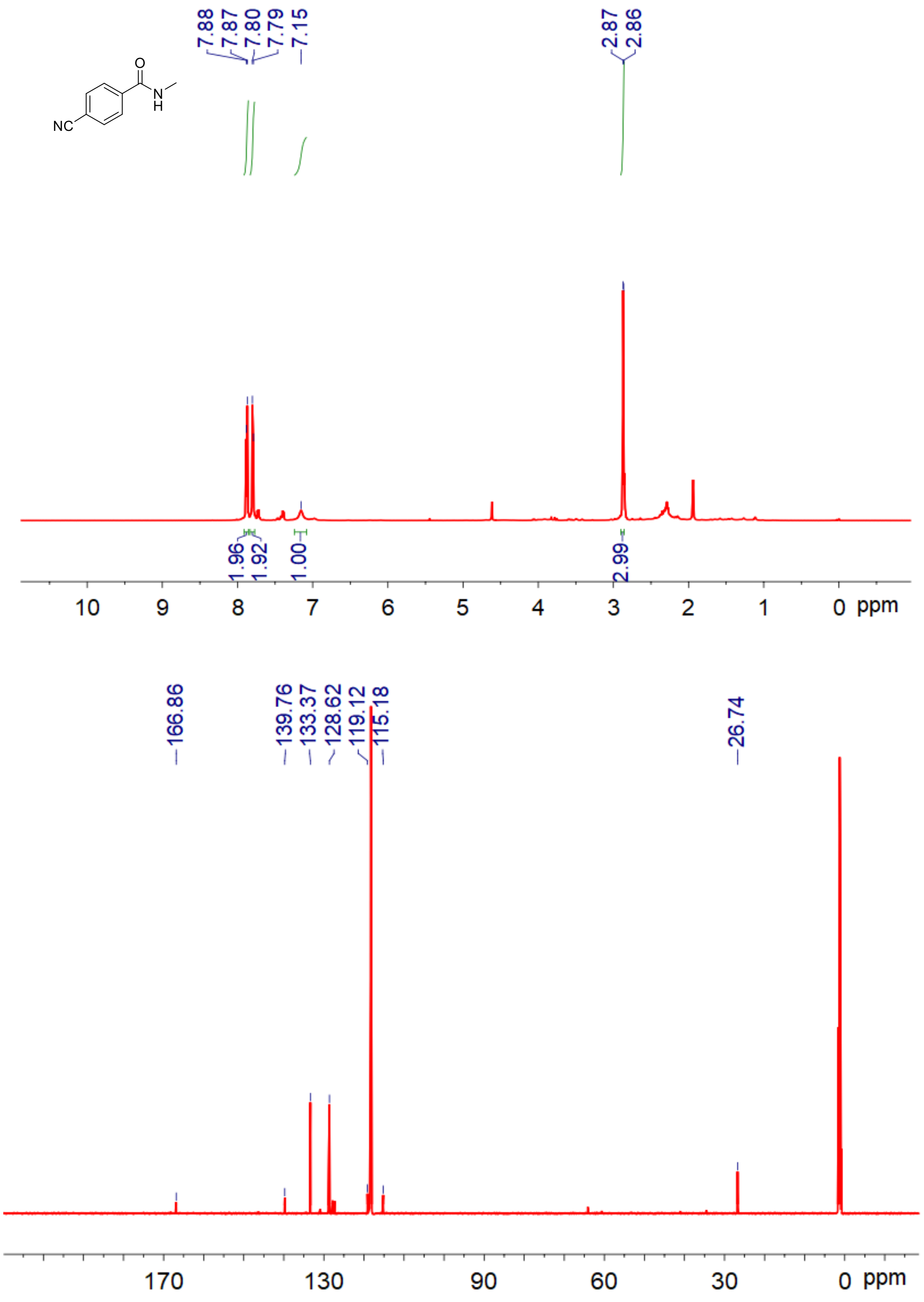

Supplementary Figure 87. ^{1}H NMR and ^{13}C NMR spectrum of 4-cyano-*N*-methylbenzamide (**56**).

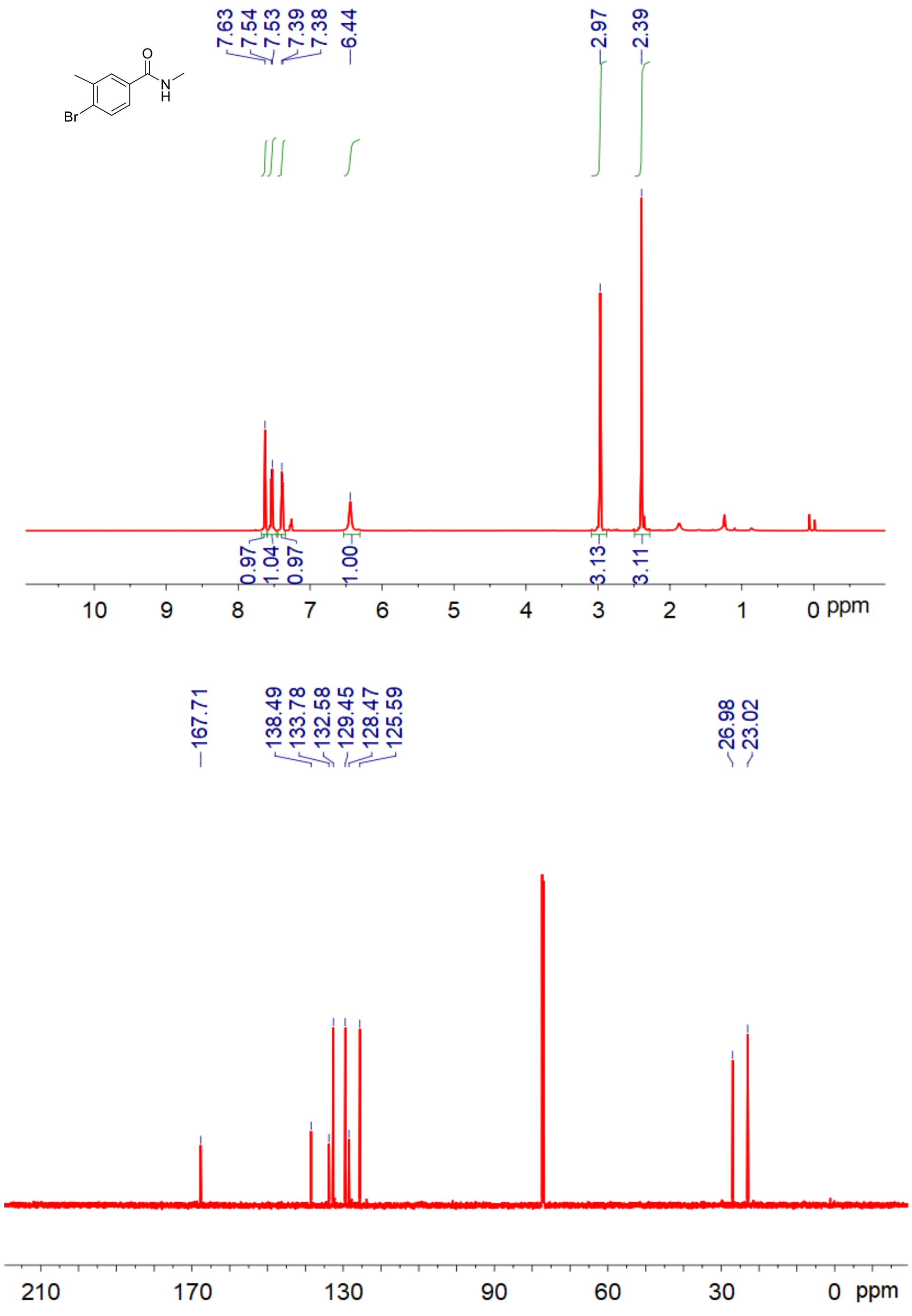

Supplementary Figure 88. ^{1}H NMR and ^{13}C NMR spectrum of 4-bromo-N,3-dimethylbenzamide (**57**).

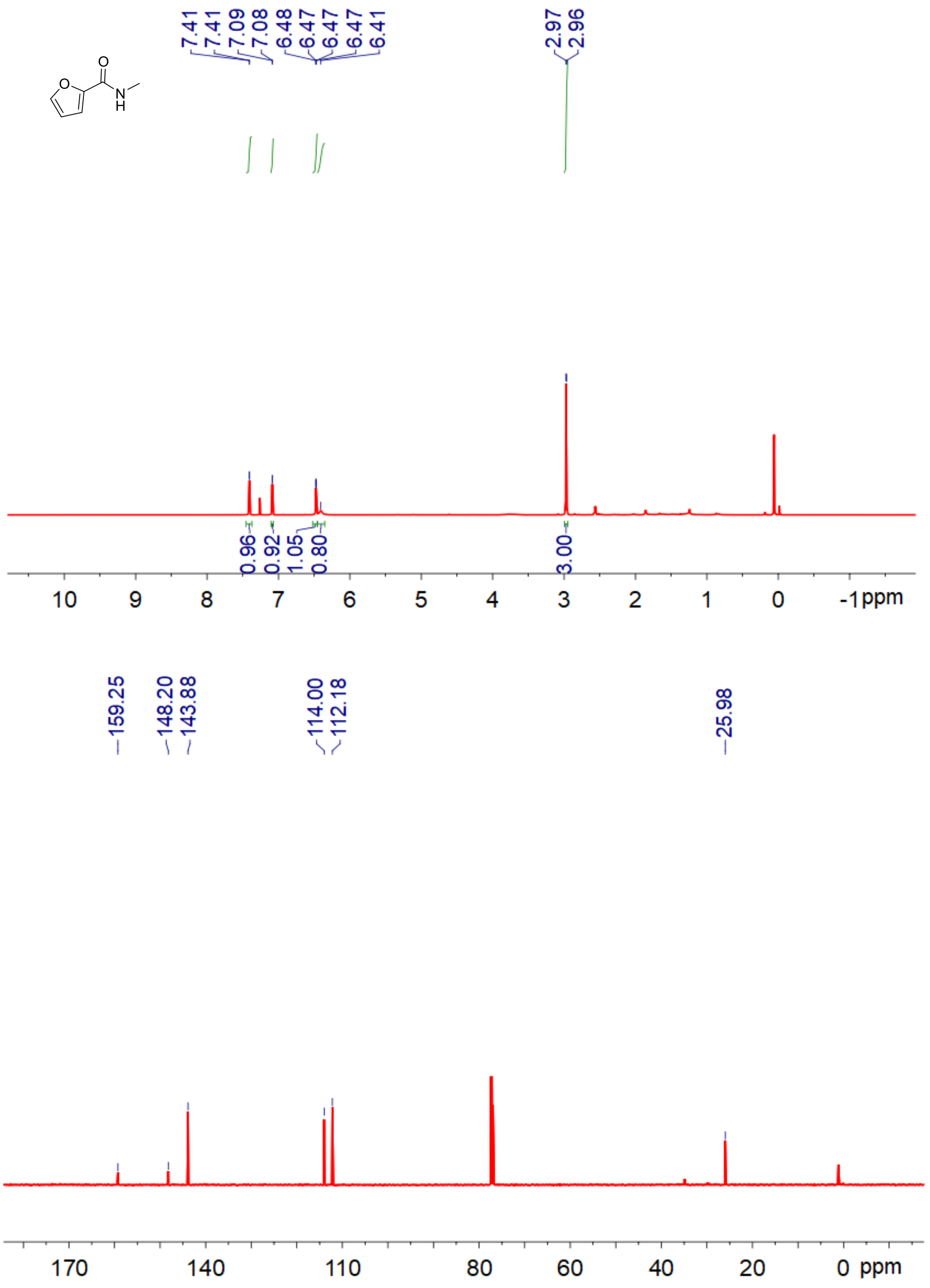

Supplementary Figure 89. ^{1}H NMR and ^{13}C NMR spectrum of *N*-methyl-2-furancarboxamide (**58**).

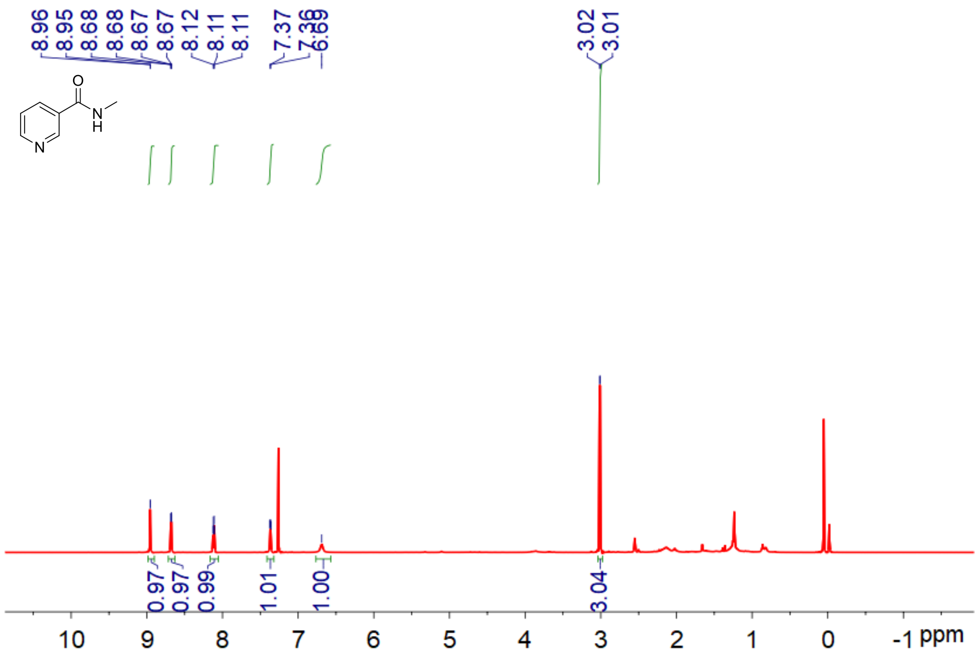

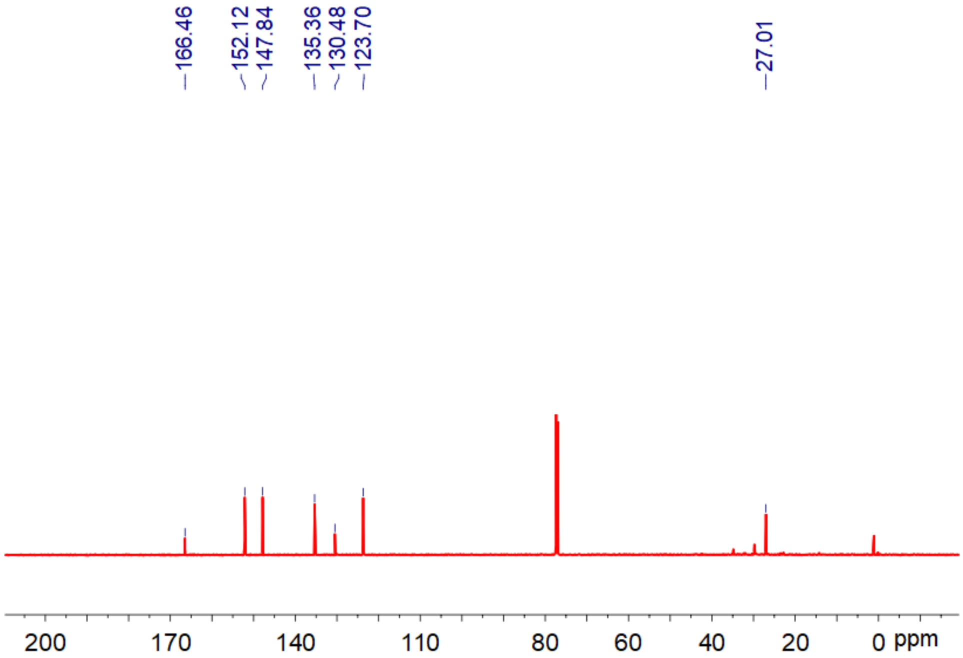

Supplementary Figure 90. ^{1}H NMR and ^{13}C NMR spectrum of *N*-methylnicotinamide (**59**).

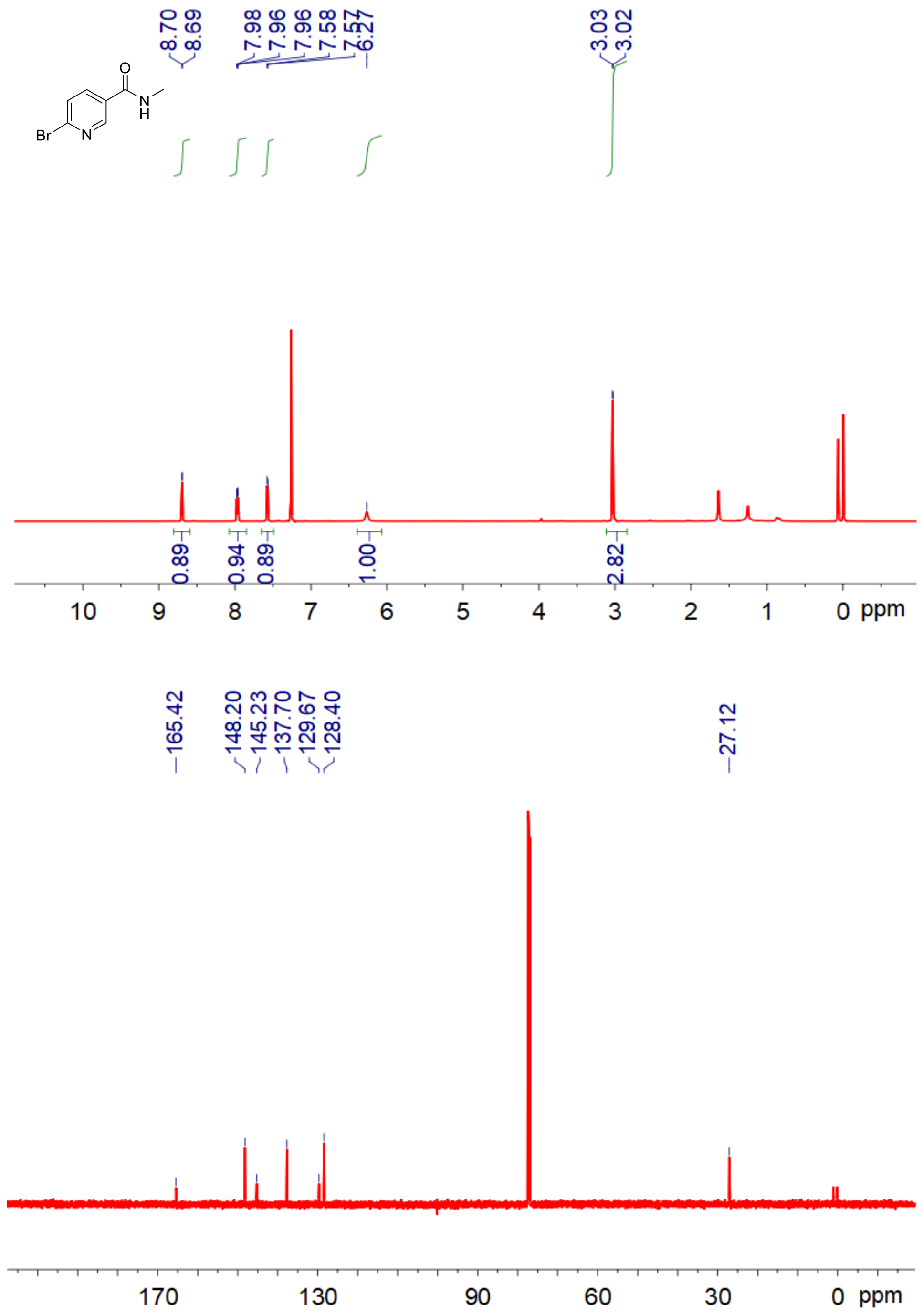

Supplementary Figure 91. ^{1}H NMR and ^{13}C NMR spectrum of 6-bromo-*N*-methylnicotinamide (**60**).

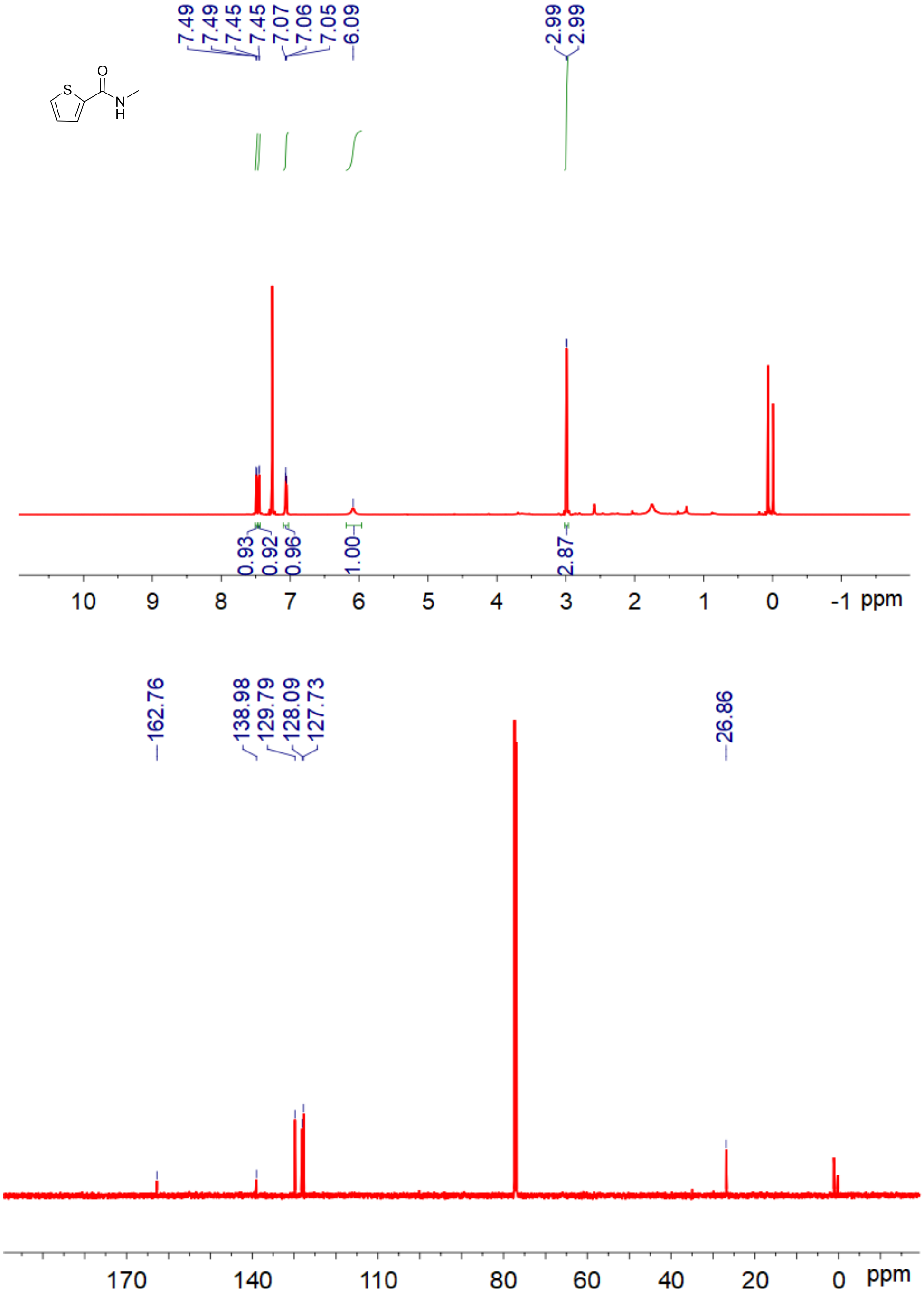

Supplementary Figure 92. ^{1}H NMR and ^{13}C NMR spectrum of *N*-methyl-2-thiophenecarboxamide (**61**).

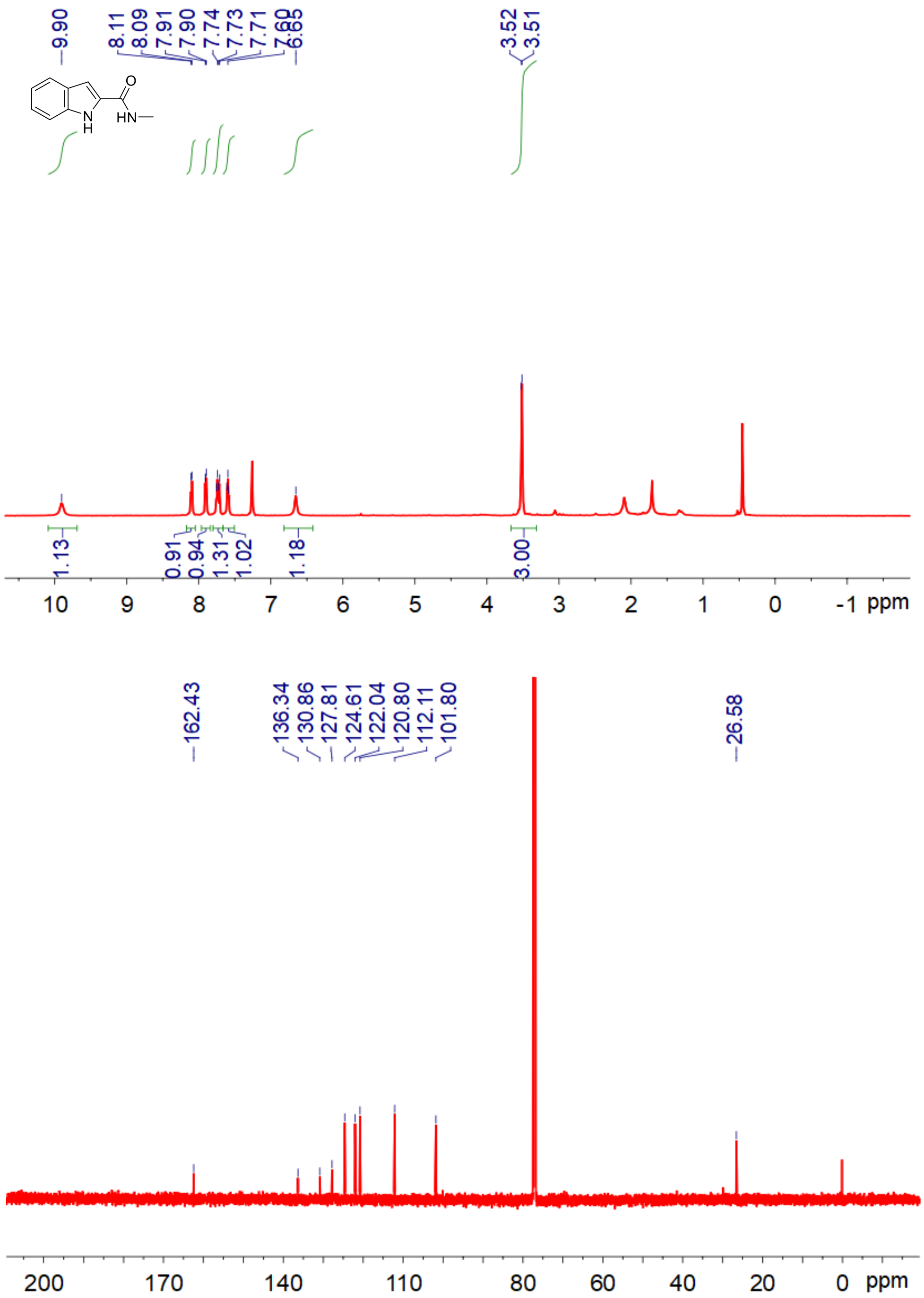

Supplementary Figure 93. ^{1}H NMR and ^{13}C NMR spectrum of *N*-methyl-1H-indole-2-carboxamide (**62**).

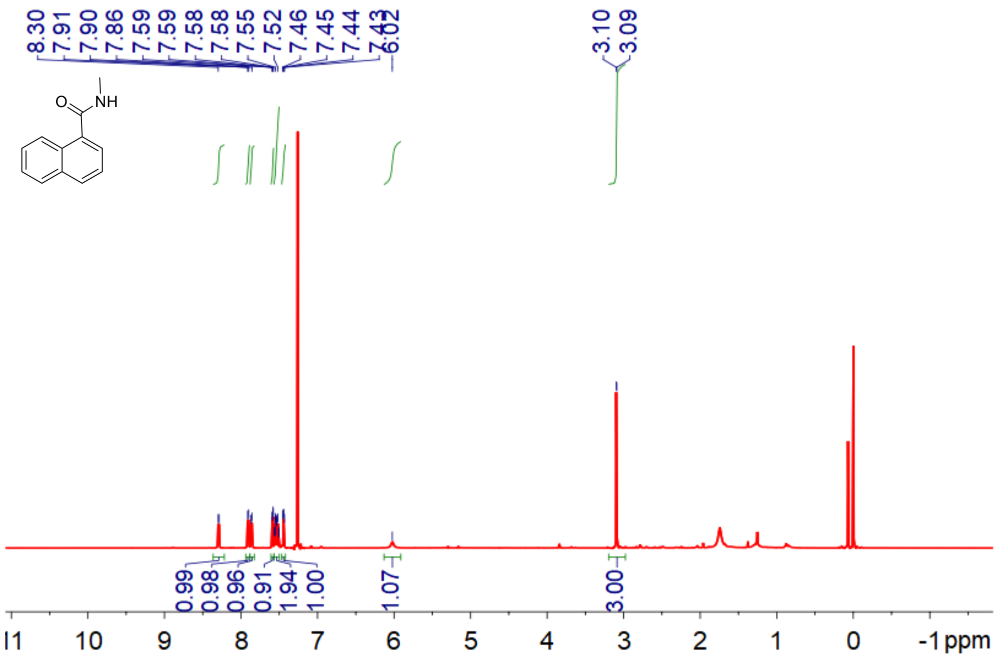

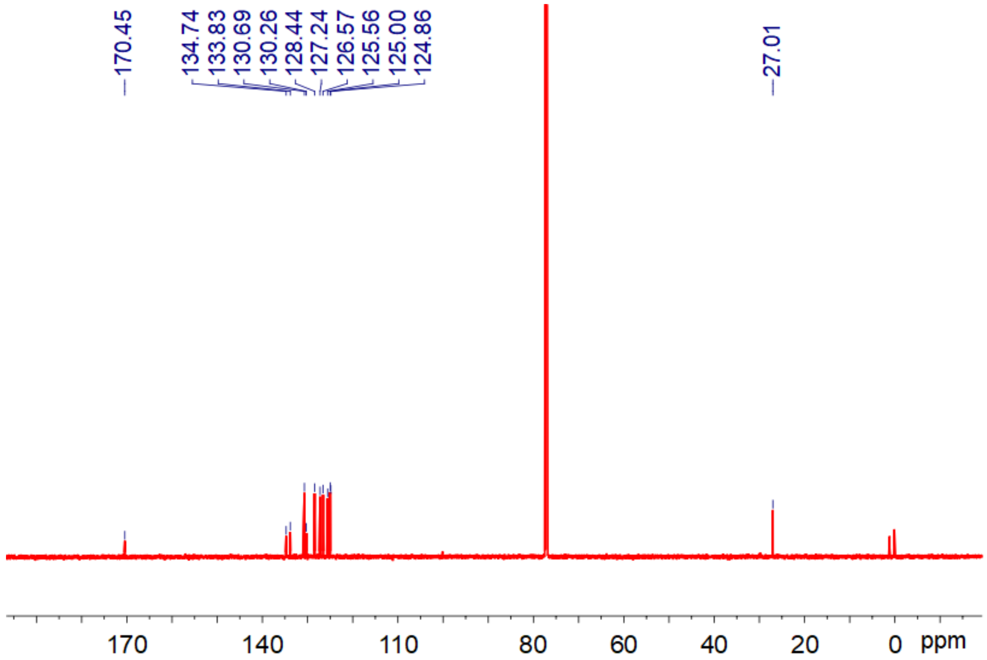

Supplementary Figure 94. ^{1}H NMR and ^{13}C NMR spectrum of *N*-methyl-1-naphthamide (**63**).

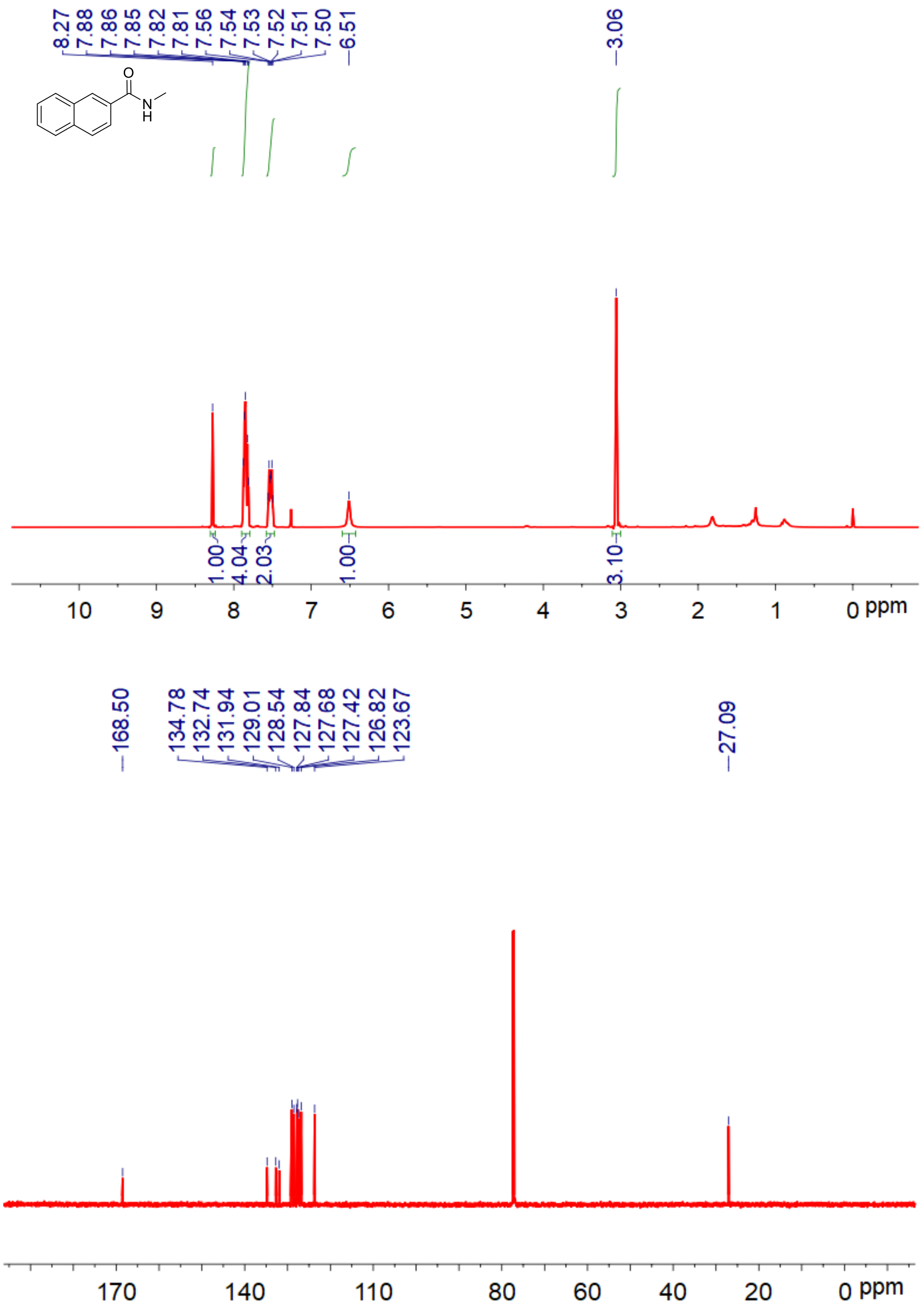

Supplementary Figure 95. ^{1}H NMR and ^{13}C NMR spectrum of *N*-methyl-2-naphthamide (**64**).

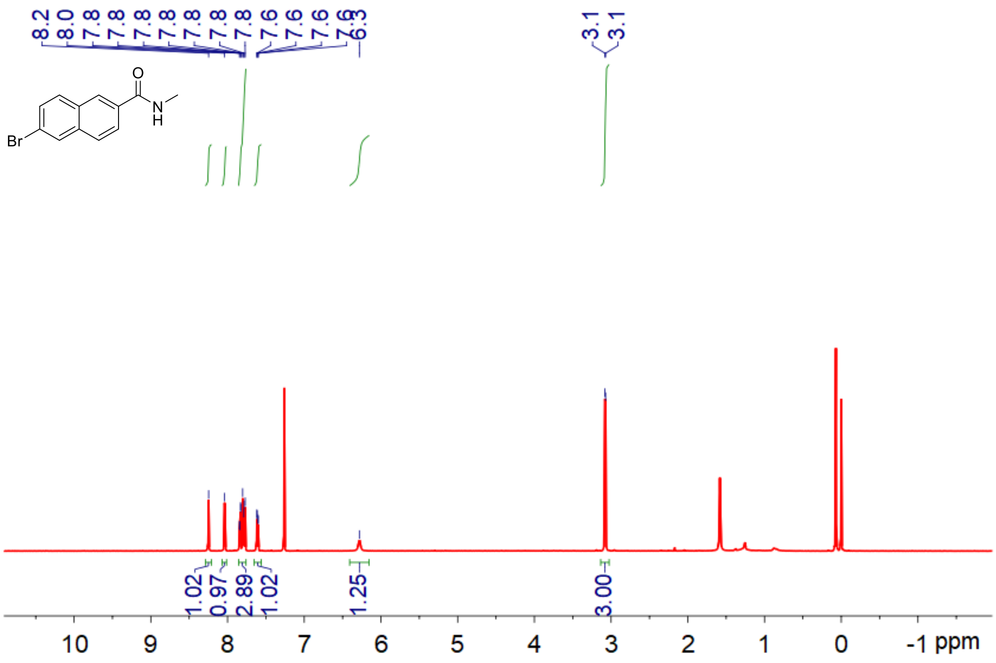

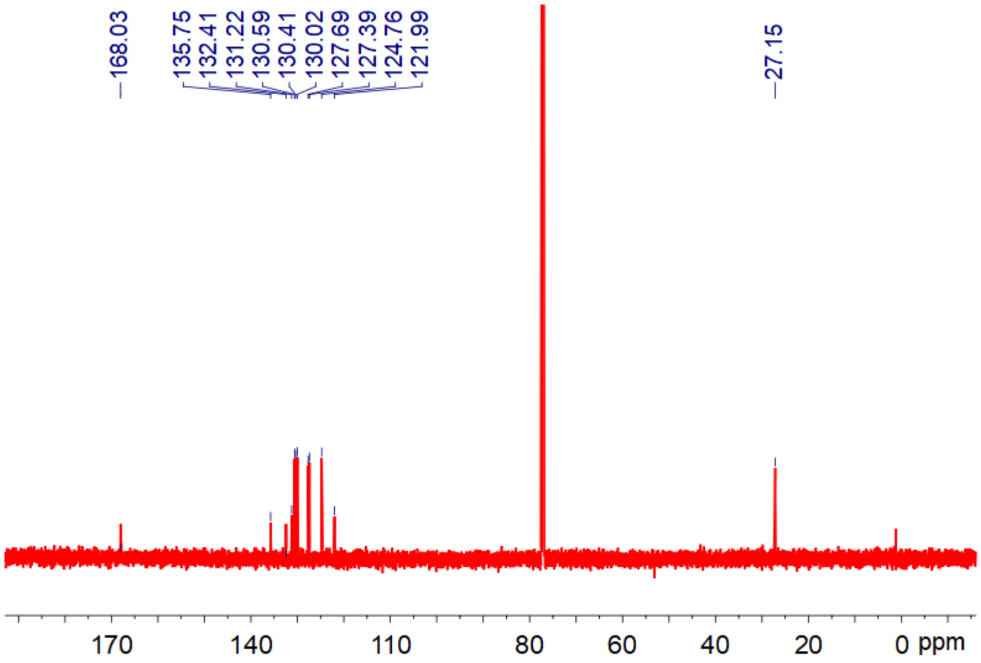

Supplementary Figure 96. ^{1}H NMR and ^{13}C NMR spectrum of 6-bromo-*N*-methyl-2-naphthamide (**65**).

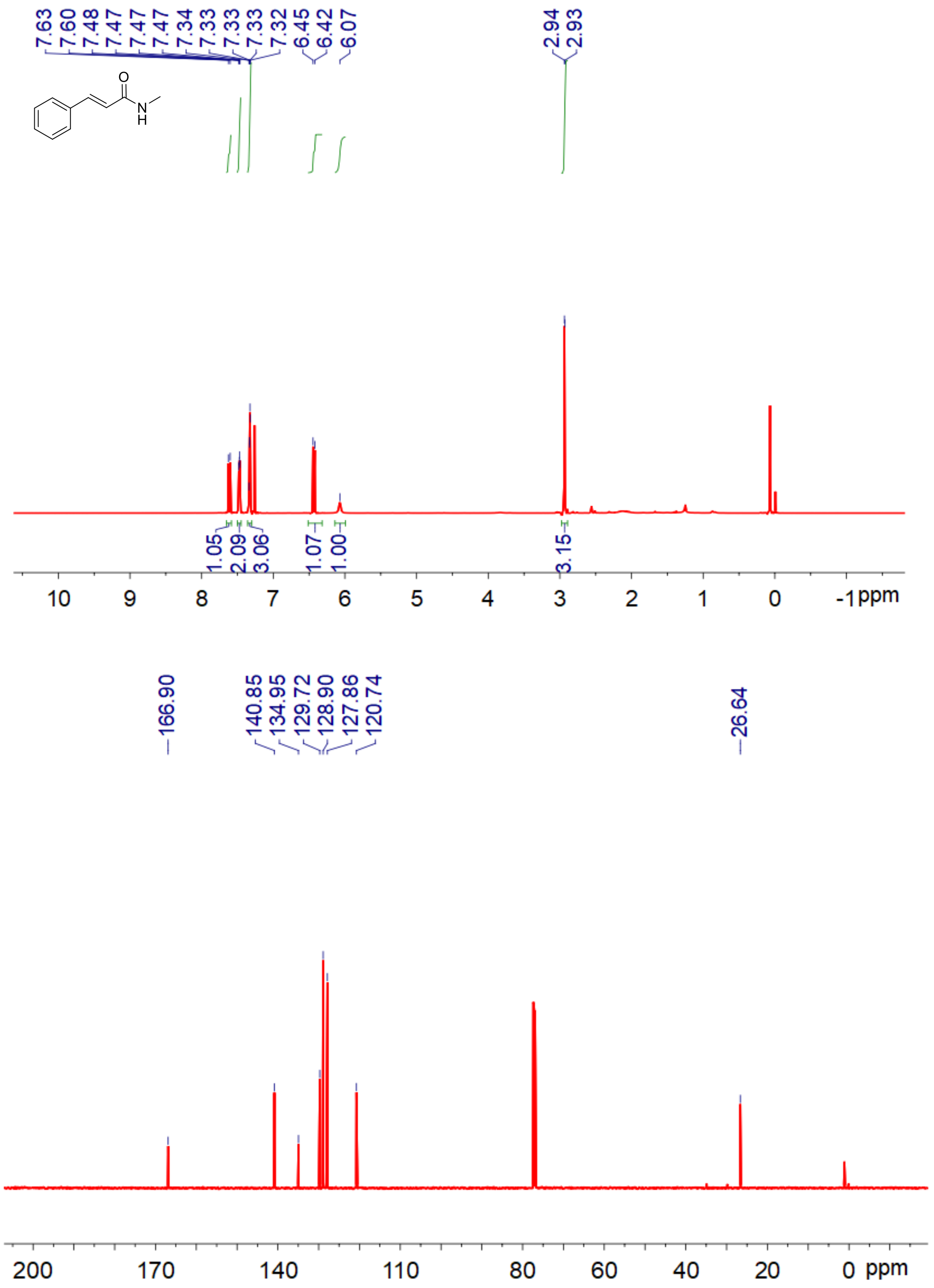

Supplementary Figure 97. ^{1}H NMR and ^{13}C NMR spectrum of *N*-methyl-3-phenylacrylamide (**66**).

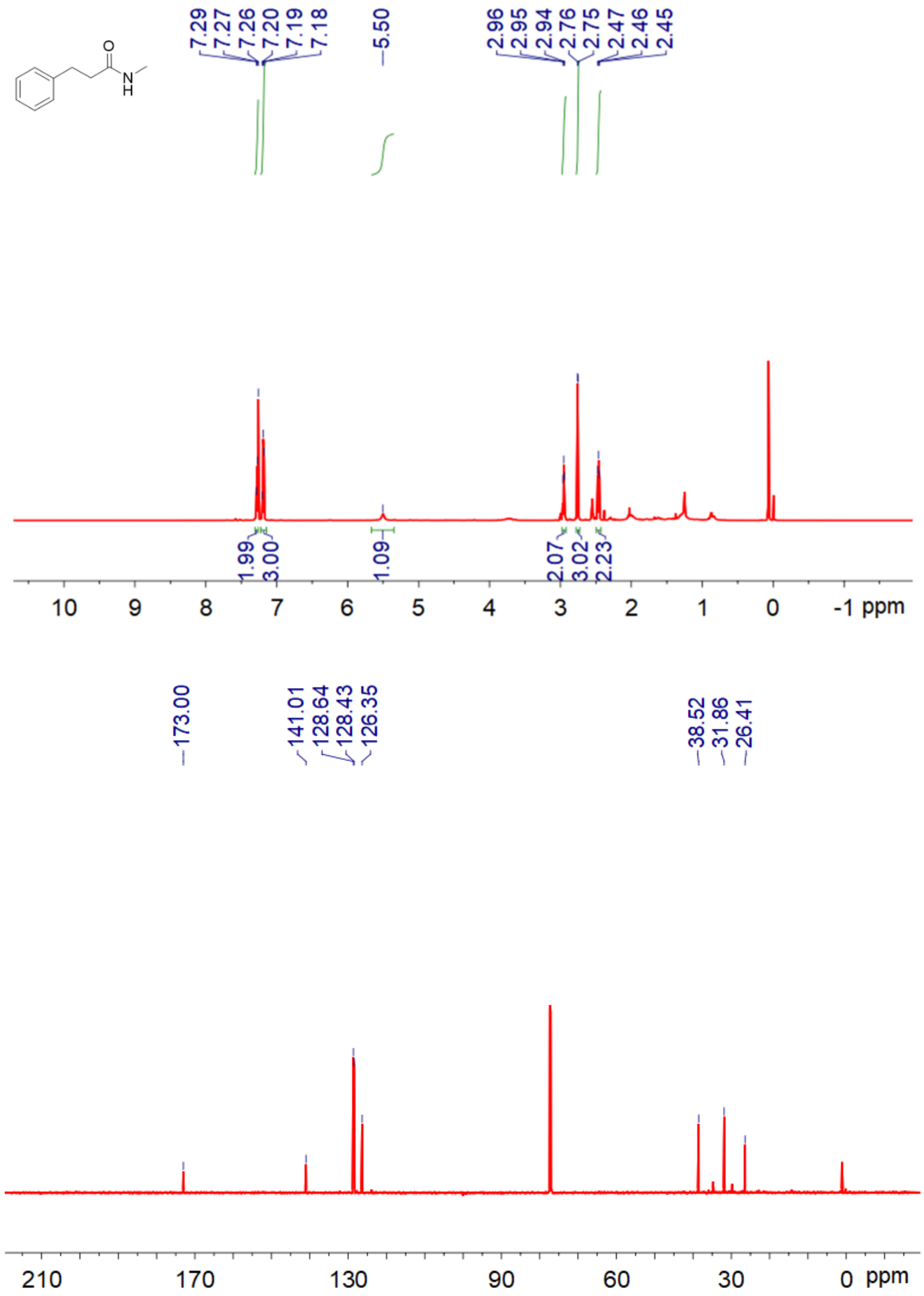

Supplementary Figure 98. ^{1}H NMR and ^{13}C NMR spectrum of *N*-methyl-3-phenylpropanamide (**67**).

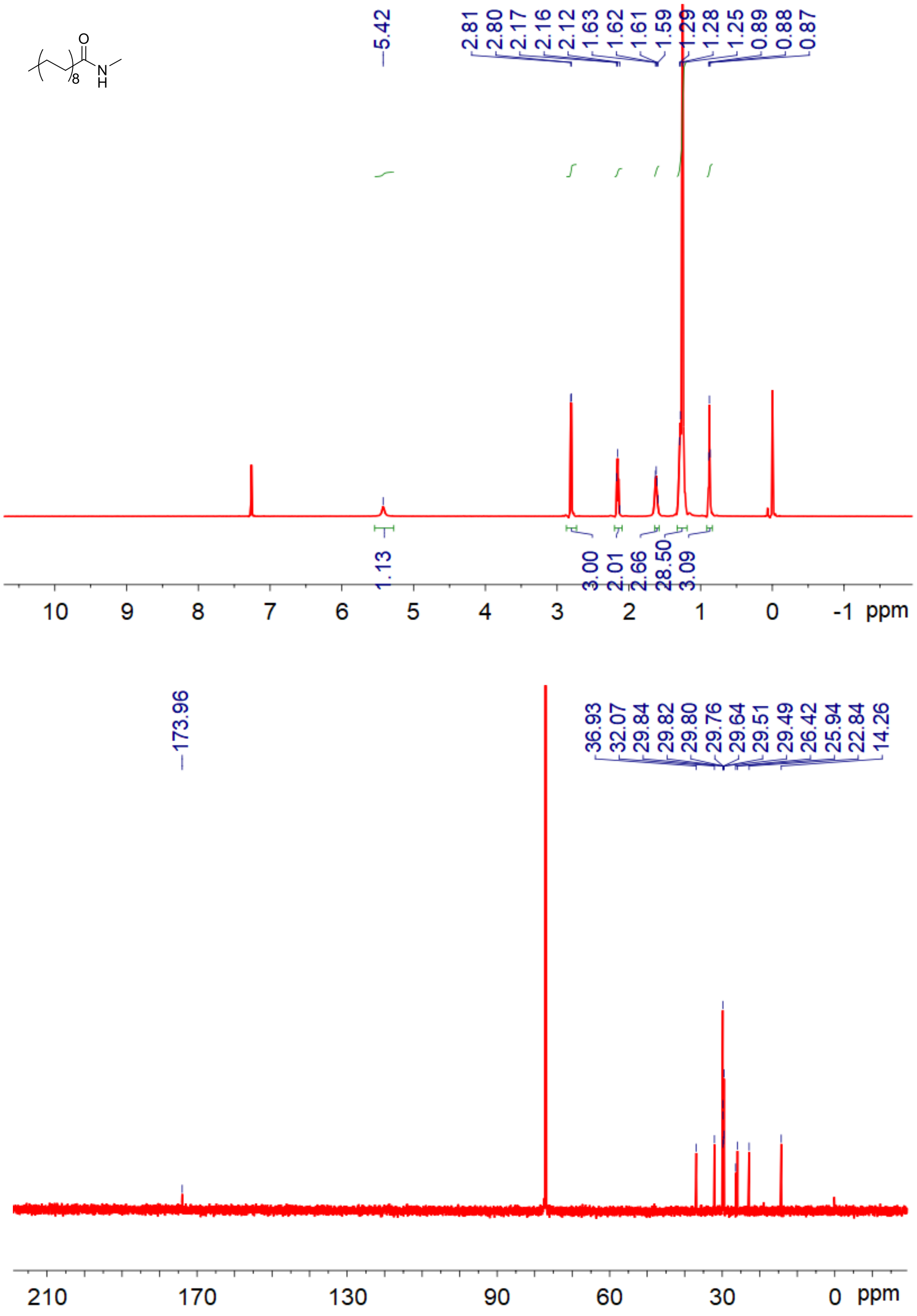

Supplementary Figure 99. ^{1}H NMR and ^{13}C NMR spectrum of *N*-methyloctadecanamide (**68**).

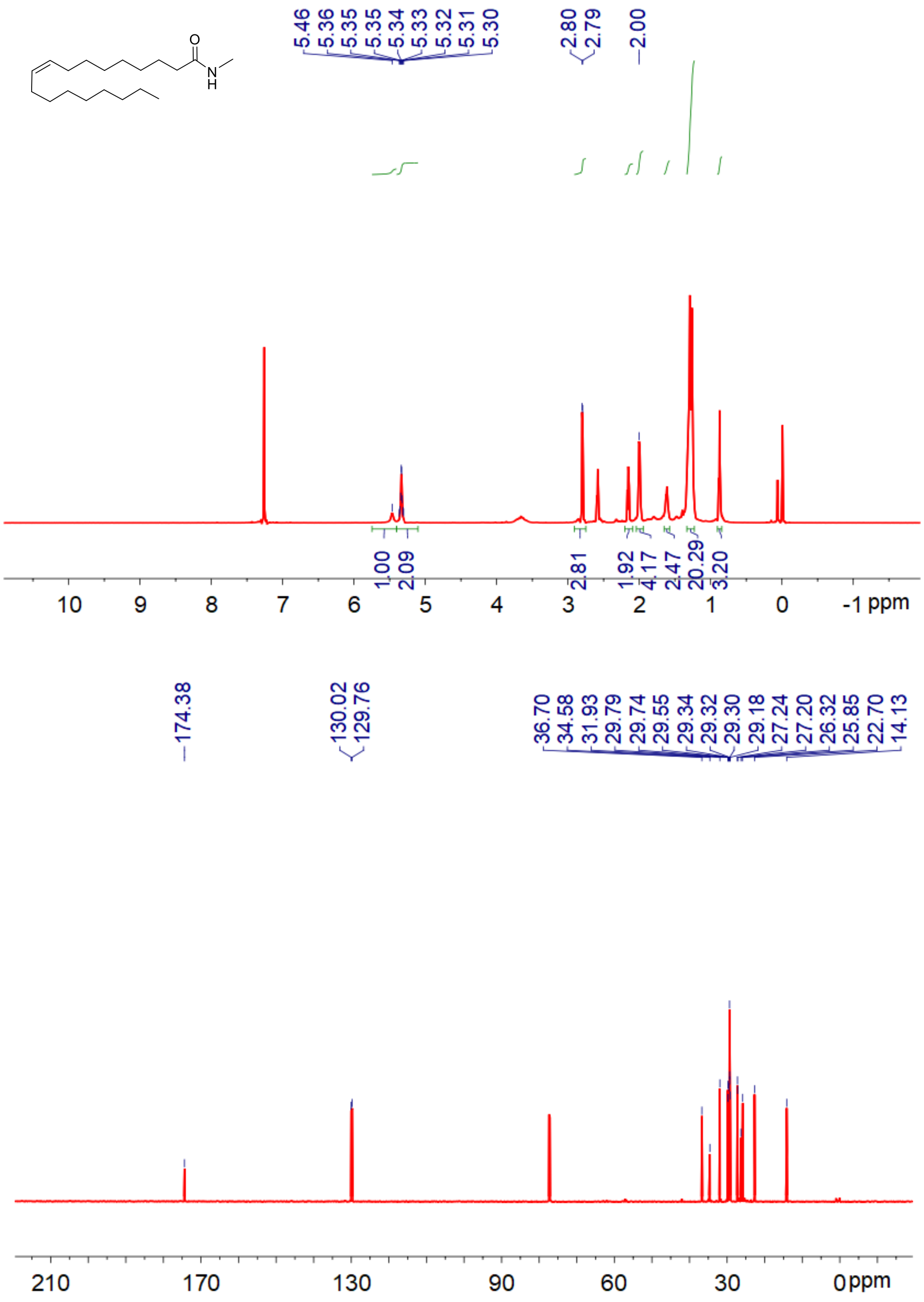

Supplementary Figure 100. ^{1}H NMR and ^{13}C NMR spectrum of *N*-methyloleamide (**69**).

4. Supplementary References

1. Luedtke, A. T. & Autrey, T. Hydrogen release studies of alkali metal amidoboranes. *Inorg. Chem*. **49**, 3905-3910 (2010).
2. Diyabalanage, H. V. K. et al. Potassium(I) amidotrihydroborate: structure and hydrogen release. *J. Am. Chem. Soc.* **132**, 11836-11837 (2010).
3. Theppawong, A. et al. Facile and divergent synthesis of lamellarins and lactam-containing derivatives with improved drug likeness and biological activities. *Chem. Asian. J.* **10**, 2631-2650 (2015).
4. Terentjeva, S., Muceniece, D., Petushkova, J. & Lūsis, V. Synthesis of novel 3-substituted benzamides related to imatinib. *J. Chem. Res.* **40**, 224-227 (2016).
5. Han, C., Lee, J. P., Lobkovsky, E. & Porco, J. A. Catalytic ester-amide exchange using group (IV) metal alkoxide-activator complexes. *J. Am. Chem. Soc.* **127**, 10039-10044 (2005).
6. Morimoto, H., Fujiwara, R., Shimizu, Y., Morisaki, K. & Ohshima, T. Lanthanum (III) triflate catalyzed direct amidation of esters. *Org. Lett.* **16**, 2018-2021 (2014).
7. Zheng, Y.-L. & Newman, S. G. Methyl esters as cross-coupling electrophiles: direct synthesis of amide bonds. *ACS. Catal.* **9**, 4426-4433 (2019).
8. Hie, L. et al. Nickel-catalyzed activation of acyl C-O bonds of methyl esters. *Angew. Chem. Int. Ed.* **55**, 2810-2814 (2016).
9. Ben Halima, T., Masson-Makdissi, J. & Newman, S. G., Nickel-catalyzed amide bond formation from methyl esters. *Angew. Chem. Int. Ed.* **57**, 12925-12929 (2018).
10. Shi, S. & Szostak, M. Pd-PEPPSI: a general Pd-NHC precatalyst for Buchwald-Hartwig cross-coupling of esters and amides (transamidation) under the same reaction conditions. *Chem. Commun.* **53**, 10584-10587 (2017).
11. Halima, T. B., Vandavasi, J. K., Shkoor, M. & Newman, S. G. A cross-coupling approach to amide bond formation from esters. *ACS. Catal.* **7**, 2176 (2017).
12. Karthik, S., Sreedharan, R. & Gandhi, T. A straightforward metal-free and mild base promoted amidation and transesterification via acyl C-O bond cleavage-An expedite synthesis of aromatic amides and esters. *Chemistry. Select*. **4**, 175-180 (2019).
13. Meng, G., Lei, P. & Szostak, M. A general method for two-step transamidation of secondary amides using commercially available, air- and moisture-stable palladium/NHC (N-Heterocyclic Carbene) complexes. *Org. Lett.* **19**, 2158-2161 (2017).
14. Wang, S., Zhao, X., Zhang-Negrerie, D. & Du, Y. Reductive cleavage of the N-O bond: elemental sulfur-mediated conversion of N-alkoxyamides to amides. *Org. Chem. Front.* **6**, 347-351 (2019).
15. Zhou, L., Liu, W., Zhao, Y. & Chen, J. Cu(Ⅱ)-promoted oxidative C-N bond cleavage of N-benzoylamino acids to primary aryl amides. *Arkivoc* **6**, 52-62 (2016).
16. Midya, G. C., Kapat, A., Maiti, S. & Dash, J. Transition-metal-free hydration of nitriles using potassium tert-butoxide under anhydrous conditions. *J. Org. Chem.* **80**, 4148-4151 (2015).
17. Lee, J., Kim. M., Chang. S. & Lee. H.-Y. Anhydrous hydration of nitriles to amides using aldoximes as the water source. *Org. lett.* **11**, 5598-5601 (2009).
18. Black, G. W., Gregson, T., McPake, C. B., Perry, J. J. & Zhang, M. Biotransformation of nitriles using the solvent-Tolerant nitrile hydratase from Rhodopseudomonas Palustris CGA009. *Tetrahedron. Lett.* **51**, 1639-1641 (2010).

19. Khalafi-Nezhad, A., Parhami, A., Soltani Rad, M. N. & Zarea, A. Efficient method for the direct preparation of amides from carboxylic acids using tosyl chloride under solvent-free conditions. *Tetrahedron. Lett.* **46**, 6879-6882 (2005).
20. Ramachandran, P. V., Hamann, H. J. & Choudhary, S. Amine-boranes as dual-purpose reagents for direct amidation of carboxylic acids. *Org. Lett.* **22**, 8593-8597 (2020).
21. Tinnis, F., Lundberg, H. & Adolfsson, H. Direct catalytic formation of primary and tertiary amides from non-activated carboxylic acids, employing carbamates as amine source. *Adv. Synth. Catal.* 354, 2531-2536 (2012).
22. Khalafinezhad, A., Mokhtari, B. & Soltanirad, M. Direct preparation of primary amides from carboxylic acids and urea using imidazole under microwave irradiation. *Tetrahedron. Lett.* **44**, 7325-7328 (2003).
23. Shimada, N. et al. Diboronic acid anhydrides as effective catalysts for the hydroxydirected dehydrative amidation of carboxylic acids. *Org. Lett.* **21**, 4303-4308 (2019).
24. Sawant, D. N., Bagal, D. B., Ogawa, S., Selvam, K. & Saito, S. Diboron-catalyzed dehydrative amidation of aromatic carboxylic acids with amines. *Org. Lett.* **20**, 4397-4400 (2018).
25. Wang, K., Lu, Y. & Ishihara, K. The ortho-substituent on 2,4-bis(trifluoromethyl)phenylboronic acid catalyzed dehydrative condensation between carboxylic acids and amines. *Chem. Commun.* **54**, 5410-5413 (2018).
26. Srinivasan, S. & Manisankar, P. Formamide as an ammonia synthon in amination of acid chlorides. *Synthetic. Commun*, **40**, 3538-3543 (2010).
27. Zarchi, M. A. K. & Bahadoran, A. Convenient synthesis of benzamides mediated by poly(4-vinylpyridine)-supported benzoyl chloride. *J. Appl. Polym. Sci.* **119**, 2345-2349 (2011).
28. Cho, S. H., Yoo, E. J., Bae, I. & Chang. S. Copper-catalyzed hydrative amide synthesis with terminal alkyne, sulfonyl azide, and water. *J. Am. Chem. Soc.* **127**, 16046-16047 (2005).
29. Chan, W.-K., Ho, C.-M., Wong, M.-K. & Che, C.-M. Oxidative amide synthesis and N-terminal α-amino group ligation of peptides in aqueous medium. *J. Am. Chem. Soc*. **128**, 14796-14797 (2006).
30. Gunanathan, C., Ben-David, Y. & Milstein, D. Direct synthesis of amides from alcohols and amines with liberation of H_2. *Science* **317**, 790 (2007).
31. Nordstrøm, L. U., Vogt, H., Madsen, R. Amide synthesis from alcohols and amines by the extrusion of dihydrogen. *J. Am. Chem. Soc.* **130**, 17672-17673 (2008).
32. Yoo, W.-J. & Li, C.-J. Highly efficient oxidative amidation of aldehydes with amine hydrochloride salts. *J. Am. Chem. Soc.* **128**, 13064-13065 (2006).
33. Gao, J. & Wang, G.-W. Direct oxidative amidation of aldehydes with anilines under mechanical milling conditions. *J. Org. Chem.* **73**, 2955-2958 (2008).
34. Chen, J., Xia, Y. & Lee, S. Transamidation for the synthesis of primary amides at room temperature. *Org. Lett*. **22**, 3504-3508 (2020).
35. Ghosh, S. & Jana, C. K. Metal-free thermal activation of molecular oxygen enabled direct α-CH_2-oxygenation of free amines. *J. Org. Chem*. **83**, 260-266 (2018).
36. Zhan, W., Ji, L., Ge, Z. M., Wang, X. & Li, R. T. A continuous-flow synthesis of primary amides from hydrolysis of nitriles using hydrogen peroxide as oxidant. *Tetrahedron* **74,** 1527-1532 (2018).
37. Roy, A. D., Subramanian, A. & Roy, R. Auto-redox reaction: Tin(II) chloride-mediated one-step reductive cyclization leading to the synthesis of novel biheterocyclic 5,6-dihydro-

quinazolino[4,3-*b*]quinazolin-8-ones with three-point diversity. *J. Org. Chem.* **71**, 382-385 (2006).
38. Bertini, V. et al. Alkylamino derivatives of 4-aminomethylpyridine as inhibitors of copper-containing amine oxidases. *J. Med. Chem.* **48**, 664-670 (2005).
39. Wagner, A. J., Zubarev, D. Y., Aspuru-Guzik, A. & Blackmond, D. G. Chiral sugars drive enantioenrichment in prebiotic amino acid synthesis. *ACS Cent. Sci.* **3**, 322-328 (2017).
40. Chakraborty, D. P., Mandal, A. K. & Roy, S. K. Ethyl carbamate as an aminocarbonylating agent: modification of gattermann's amidation reaction. *Synthesis* **12**, 977-979 (1981).
41. Das, H. S. et al. Primary amides to amines or nitriles: a dual role by a single catalyst. *Chem. Commun.* **55**, 11868-11871 (2019).
42. Nielsen, D. U., Taaning, R. H., Lindhardt, A. T., Goegsig, T. M. & Skrydstrup, T. Palladium-catalyzed approach to primary amides using nongaseous precursors. *Org. Lett*. **13**, 4454-4457 (2011).
43. Pu, C. et al. Synthesis and biological evaluation of indole derivatives as vif inhibitors. *Bioorgan. Med. Chem. Lett.* **27**, 4150-4155 (2017).
44. Csomos, P., Fodor, L., Mandity, I. & Bernath, G. An efficient route for the synthesis of 2-arylthiazino[5,6-b]indole derivatives. *Tetrahedron* **63**, 4983-4989 (2007).
45. Zhou, H., Wang, Z. S., Liu, X. H. & Chen, F. H. Novel amidrazone derivatives: design, synthesis and activity evaluation. *Bioorgan. Med. Chem*. **26**, 3158-3165 (2018).
46. Ohmura, R., Takahata, M. & Togo, H. Metal-free one-pot oxidative conversion of benzylic alcohols and benzylic halides into aromatic amides with molecular iodine in aq ammonia, and hydrogen peroxide. *Tetrahedron Lett*. **51**, 4378-4381 (2010).
47. Xu, M.-Z. et al. Acyl-CoA: cholesterol acyltransferase inhibitory activities of fatty acid amides isolated from mylabris phalerate pallas. *Bioorg. Med. Chem. Lett*. **14**, 4277-4280 (2004).
48. Veitch, G. E., Bridgwood, K. L. & Ley, S. V. Magnesium nitride as a convenient source of ammonia: preparation of primary amides. *Org. Lett.* **10**, 3623-3625 (2008).
49. Breuilles, P., Leclerc, R. & Uguen, D. A mild hydration of nitriles into amides. *Tetrahedron Lett*. **35**, 1401-1404 (1994).
50. Koltunov, K. Y., Walspurger, S. & Sommer, J. Superacidic activation of α,β-unsaturated amides and their electrophilic reactions. *Eur. J. Org. Chem.* 4039-4047 (2004).
51. Guguloth, V. C. & Battu, S. Synthesis of some aromatic and aliphatic esters using WO_3/ZrO_2 solid acid catalyst under solvent free conditions. *Asian J. Chem*. **32**, 2153-2157 (2020).
52. Shaikh, N. S., Junge, K. & Beller, M. A convenient and general iron-catalyzed hydrosilylation of aldehydes. *Org. Lett*. **9**, 5429-5432 (2007).
53. Murai, N., Yonaga, M. & Tanaka, K. Palladium-catalyzed direct hydroxymethylation of aryl halides and triflates with potassium acetoxymethyltrifluoroborate. *Org. Lett*. **14**, 1278-1281 (2012).
54. Ackermann, L., Lygin, A. V. & Hofmann, N. Ruthenium-catalyzed oxidative annulation by cleavage of C-H/N-H bonds. *Angew. Chem. Int. Ed*. **50**, 6379-6382 (2011).
55. Jo, Y., Ju, J., Choe, J., Song, K. H. & Lee, S. The scope and limitation of nickel-catalyzed aminocarbonylation of aryl bromides from formamide derivatives. *J. Org. Chem*. **74**, 6358-6361 (2009).
56. Hyster, T. K. & Rovis, T. Rhodium-catalyzed oxidative cycloaddition of benzamides and alkynes via C-H/N-H activation. *J. Am. Chem. Soc*. **132**, 10565-10569 (2010).

57. Nørager, N. G. & Juhl, K. Conjugate addition-S_NAr domino reaction for the synthesis of benzo- or pyridyl-fused lactams and sultams. *Synthesis* 4273-4281 (2010).
58. Gallasch, D. P., Woodhouse, S. L. & Rendina, L. M. Synthesis and redistribution reactions of asymmetric σ-arylplatinum(II) complexes containing 4,7-phenanthroline. *J. Org. Chem.* **689**, 1288-1294 (2004).
59. Kuang, L., Zhou, J., Chen, S. & Ding, K. Room-temperature debenzylation of N-benzylcarboxamides by N-bromosuccinimide. *Synthesis* 3129-3134 (2007).
60. Chen, Z. et al. Acyl cyanides as bifunctional reagent: application in copper-catalyzed cyanoamidation and cyanoesterification reaction. *J. Org. Chem*. **85**, 5691-5701 (2020).
61. Chan, B. et al. Preparation of naphthyridine compounds as inhibitors of HPK1 useful for treatment of cancer. *PCT Int. Appl*. WO 2018183956A1 (2018).
62. Ringeissen, S. et al. Potential urinary and plasma biomarkers of peroxisome proliferation in the rat: identification of N-methylnicotinamide and N-methyl-4-pyridone-3-carboxamide by ^{1}H nuclear magnetic resonance and high performance liquid chromatography. *Biomarkers* **8**, 240-271 (2003).
63. Strang, R. S., Lunn, G. & Mathias, J. P. Preparation of tetrahydronaphthyridines as histamine H3 receptor ligands. *Eur. Pat. Appl.* EP 1595881A1 (2005).
64. An, J. et al. Efficient and general synthesis of oxazino[4,3-a]indoles by cascade addition-cyclization reactions of (1H-indol-2-yl)methanols and vinyl sulfonium salts. *Chem. Commun.* **47**, 1869-1871 (2011).
65. Kaku, T. et al. Discovery of orteronel (TAK-700), a naphthylmethylimidazole derivative, as a highly selective 17, 20-lyase inhibitor with potential utility in the treatment of prostate cancer. *Bioorg. Med. Chem*. **19**, 6383-6399 (2011).
66. Zhu, C.-L. & Wang, X.-X. A novel method for the synthesis of α,β-unsaturated amides mediated by samarium diiodide. *J. Chin. Chem. Soc*. **52**. 953-955 (2005).
67. Asai, S., Ban, K., Monguchi, Y., Sajiki, H. & Sawama, Y. Selective N-monoalkylation of amide derivatives with trialkyl phosphates. *Synlett* **29**, 322-325 (2018).
68. Ito, A. et al. A derivative of oleamide potently inhibits the spontaneous metastasis of mouse melanoma BL6 Cells. *Carcinogenesis* **25**, 2015-2022 (2004).
69. Lai, J.-C. et al. Thermodynamically stable whilst kinetically labile coordination bonds lead to strong and tough self-healing polymers. Nature Commun. 10, 1164 (2019).
70. Tian, L. & Chen, F. Multiwfn: a multifunctional wavefunction analyzer. J. Comput. Chem. 33, 580-592 (2012).
